# Diamond formation due to a pH drop during fluid–rock interactions

**DOI:** 10.1038/ncomms9702

**Published:** 2015-11-03

**Authors:** Dimitri A. Sverjensky, Fang Huang

**Affiliations:** 1 Department of Earth and Planetary Sciences, Johns Hopkins University, Charles and 34th Streets, Baltimore, Maryland 21218, USA

## Abstract

Diamond formation has typically been attributed to redox reactions during precipitation from fluids or magmas. Either the oxidation of methane or the reduction of carbon dioxide has been suggested, based on simplistic models of deep fluids consisting of mixtures of dissolved neutral gas molecules without consideration of aqueous ions. The role of pH changes associated with water–silicate rock interactions during diamond formation is unknown. Here we show that diamonds could form due to a drop in pH during water–rock interactions. We use a recent theoretical model of deep fluids that includes ions, to show that fluid can react irreversibly with eclogite at 900 °C and 5.0 GPa, generating diamond and secondary minerals due to a decrease in pH at almost constant oxygen fugacity. Overall, our results constitute a new quantitative theory of diamond formation as a consequence of the reaction of deep fluids with the rock types that they encounter during migration. Diamond can form in the deep Earth during water–rock interactions without changes in oxidation state.

## Abstract (web summary)

The cause of diamond precipitation has previously been attributed to poorly understood redox changes at depth. Here, the authors propose that a drop in pH during water–rock interactions leads to diamond formation as a consequence of the migration of reactive fluids at elevated temperatures and pressures.

## Graphical abstract

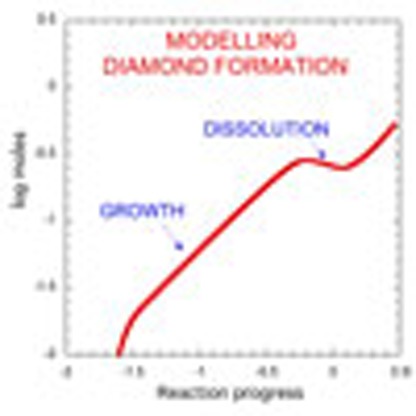

Diamonds record fluid migration events over billions of years in the deep carbon cycle in the Earth's interior^1^,^2^,^3^. Major advances in our understanding of the origin of diamonds in the deep Earth have come from studies of these fluids^4^,^5^,^6^,^7^,^8^,^9^,^10^,^11^,^12^. Aqueous fluids, melts and/or supercritical mixtures of the two are suspected to have been involved in the origin of diamonds in both the cratonic lithospheric mantle^1^,^9^,^11^,^12^,^13^,^14^,^15^ and the ultra-high pressure metamorphic environments^4^,^5^. However, previous quantitative models of the fluids involved in diamond formation have been so simplistic as to severely restrict the scope of theories about the origin of diamonds.

Traditional models of the fluids involved in diamond formation have long treated the fluids as mixtures of neutral gas molecules such as CO_2_, CH_4_, H_2_ and H_2_O (that is, COH fluids) without consideration of aqueous ions or species derived from silicate rock components^4^,^16^. Consequently, the only link in the models between the fluids and their silicate host environments is the fugacity of oxygen 
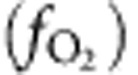
. For this reason, the cause of diamond precipitation has typically been attributed to redox changes^1^,^2^,^3^,^4^,^13^,^17^. Addressing alternate possibilities, such as the possible role of evolving fluid chemistry during fluid–rock interaction as a cause of diamond precipitation, has not been possible. Modelling diamond formation during silicate alteration reactions involving aqueous ions and aqueous species derived from silicate rocks could not be attempted, because COH models at high pressures do not contain any ions or silicate rock components.

Recent advances in theoretical and experimental aqueous geochemistry resulting in the Deep Earth Water (DEW) model enable calculation of equilibrium constants involving minerals and aqueous ions, metal complexes and organics to 6.0 GPa and 1,200 °C (refs 18, 19, 20, 21). In the present study, we use these advances to address the potential role of evolving aqueous fluid chemistry in the origin of diamonds. We first calibrate our model at 5.0 GPa and 900 °C using experimental data (see Calculation Methods and Supplementary Table 1 and Fig. 1), then we model diamond formation during the irreversible chemical mass transfer associated with aqueous fluid–rock interactions^22^,^23^, to investigate the role of pH changes in driving diamond precipitation. The model uses a conceptual scenario in which fluids from the oceanic mantle lithosphere in a subducting plate react with eclogitic mineral assemblages at 900 °C and 5.0 GPa, as a generic scenario of subcratonic lithospheric diamond formation (Calculation Methods). The aim of the present study is not to model the formation of specific diamonds and their fluid and solid inclusions. Instead, we demonstrate how it is now possible to monitor evolving fluid chemistry during diamond formation and the potential importance of pH drop as a new mechanism for diamond formation.

## Results

### Mineral products during fluid–rock interaction

**Figure 1:**
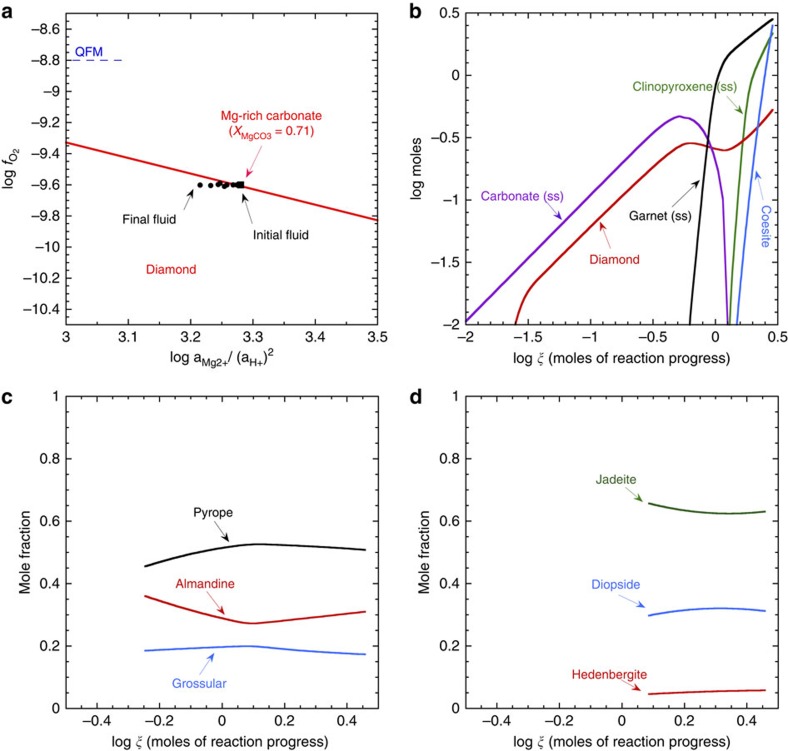
Predicted reaction path and mineral products of the fluid–rock interactions forming diamond. (**a**) Reaction path of the fluid (black dots) relative to the equilibrium boundary between a specific magnesite component composition in a carbonate solid solution, diamond, aqueous species and O_2,*g*_. (**b**) Minerals produced during the reaction of the fluid with eclogite (Supplementary Table 2). (**c**) Composition of the garnet solid solution produced during the reaction of the fluid with eclogite (Supplementary Table 2). (**d**) Composition of the clinopyroxene solid solution produced during the reaction of the fluid with eclogite (Supplementary Table 2). All calculations refer to 900 °C and 5.0 GPa. In **b**,**c**,**d**, the *x* axis represents the logarithm of the reaction progress variable *ξ*, which is equal to the number of moles of each reactant mineral destroyed during the reaction progress.

An initial fluid chemistry (Supplementary Table 2) was set by reaction of water with a carbonated mafic rock^24^ resulting in a fluid very close to equilibrium with a Mg-rich carbonate solid solution and diamond (Fig. 1a). The fluid was then permitted to react with a model metasedimentary eclogite^25^,^26^ consisting of clinopyroxene (mole fractions of jadeite 0.7, diopside 0.2 and hedenbergite 0.1), garnet (mole fractions of pyrope 0.6, almandine 0.3 and grossular 0.1) and coesite. The final fluid chemistry is given in Table 1 (see also Supplementary Note 1 for the full reaction path). The reactions produced abundant diamond, while reactant silicate minerals were destroyed (Fig. 1b). As the initial fluid was in equilibrium with Mg-rich carbonate solid solution (magnesite 0.71, siderite 0.12 and calcite 0.17), secondary Mg-rich carbonate was predicted to precipitate and replace eclogite at small extents of reaction progress (log*ξ* values of −2.0 to about −0.30; Fig. 1b). However, at large extents of reaction progress (log*ξ* values greater than about −0.30, Fig. 1b), carbonate and eclogite are replaced by diamond, secondary garnet, clinopyroxene and coesite. The predicted secondary garnet and clinopyroxene compositions (Fig. 1c,d) could serve as models of the solid inclusions typically preserved in natural diamonds^13^. Future studies could include more detailed comparisons of predicted evolving solid solutions in garnet and pyroxene during diamond formation with specific inclusion compositions in natural diamonds.

It is interesting to note that the first part of the reaction path shown in Fig. 1b (log*ξ*<−0.30), corresponding to the smallest extents of fluid–rock interaction, could correspond to the formation of diamond in a Mg-rich carbonate-metasomatized eclogite. It might be expected that the carbonate phase could disaggregate during transport in a kimberlite magma^1^, which would result in xenocryst diamonds hosted by kimberlite rock. In contrast, the second part of the reaction path in Fig. 1b (log*ξ*>−0.30), corresponding to much larger extents of fluid–rock interaction, could correspond to the formation of diamond, clinopyroxene and garnet-metasomatized eclogite. Under these circumstances, diamonds may well be preserved in their eclogitic host–rock during the kimberlitic eruption process. If such large extents of fluid–rock interaction are uncommon, the overall model in Fig. 1b may help to explain the comparative rarity of diamonds set in a matrix of eclogite^27^ rock.

### Changes in the aqueous phase during fluid–rock interaction

**Figure 2:**
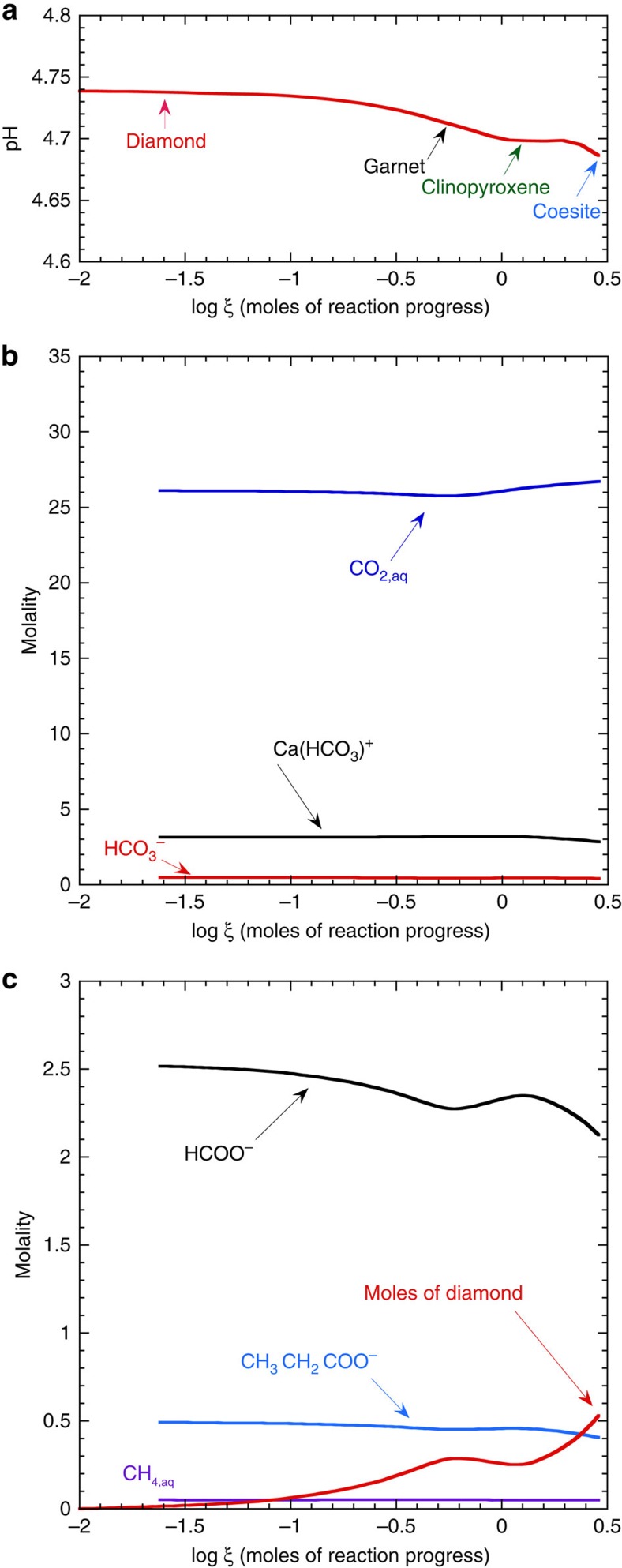
Predicted aqueous phase evolution during fluid–rock interactions forming diamond. (**a**) Evolution of the pH of the fluid during reaction with eclogite. (**b**) Evolution of the concentrations of the major inorganic oxidized carbon species. (**c**) Number of moles of diamond precipitated per kg of water compared with the evolution of the concentrations of the major aqueous organic carbon species. In **a**,**b**,**c**, the *x* axis represents the logarithm of the reaction progress variable *ξ*, which is equal to the number of moles of each reactant mineral destroyed during the reaction progress. All calculations refer to 900 °C and 5.0 GPa.

It can be seen in Fig. 1a that the log *f*_O_2__ is almost unchanged (<0.01 log units) during the entire reaction path. However, in Fig. 2a, it can be seen that the pH decreases during most of the reaction path. It should be noted here that neutral pH under these conditions is about 2.7 because of the drastic increase in the dissociation constant of water at this temperature and pressure. The reason for the pH decrease of the reacting fluids can be seen in the irreversible reaction path traced on the diagram in Fig. 1a. In contrast to traditional COH models, the equilibrium between a given activity of the magnesite component of a carbonate mineral and diamond represented by the solid line in Fig. 1a is univariant at constant temperature and pressure according to the reaction


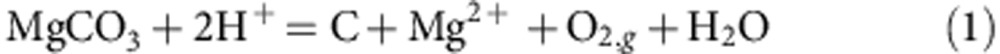


The univariance of the equilibrium in equation (1) arises, because HCl is a chemical component that provides ions^28^.

It can be seen in Fig. 1b that reaction of the fluid with eclogitic minerals in the earliest stages of reaction progress (−2.0<log*ξ*<−0.30) causes the precipitation of diamond and a secondary Mg-rich carbonate mineral according to the reactions


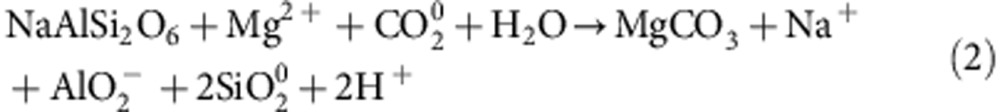



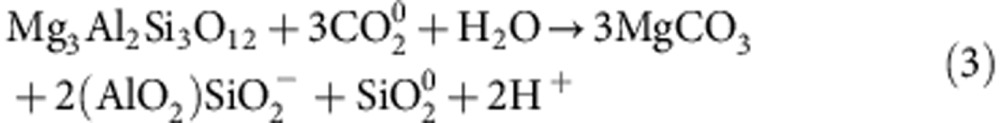


These reactions remove the Mg^2+^ ion and add the (AlO_2_)SiO_2_^−^ complex and H^+^ ions to the fluid, causing the ratio of 
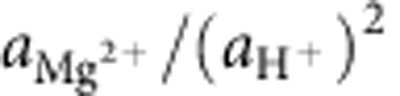
 and the pH to decrease, which results in the fluid chemistry moving into the diamond stability field in Fig. 1a.

In the later stages of reaction progress (−0.30<logξ<+0.1), the fluid is directly altering primary eclogitic clinopyroxene to secondary garnet with an increasing pyrope component (Fig. 1c) according to the reaction


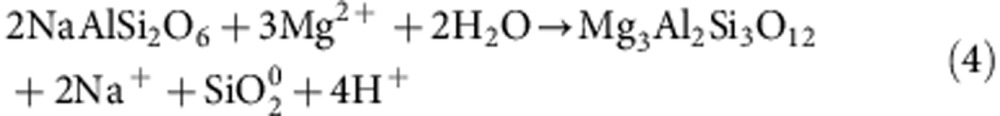


These reactions remove the Mg^2+^ ion and add H^+^ ions to the fluid, again causing the ratio of 
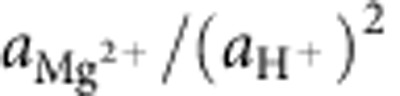
 and the pH to decrease, which results in the fluid chemistry moving further into the diamond stability field in Fig. 1a.

### Aqueous organic carbon species during diamond formation

The overall decrease in the pH of the fluid seen in Fig. 2a also causes changes in the most abundant carbon species in the fluid (Fig. 2b): the concentration of the aqueous species CO_2,aq_ increases by 623 mmolal, which is mainly balanced by decreases in the concentrations of the Ca(HCO_3_)^+^ complex, HCO_3_^−^ and CO_3_^2−^ (−300, −75 and −62 mmolal , respectively) plus a decrease of about −160 mmolal of C from organic carbon species (Supplementary Table S1). However, it can be seen in Fig. 2c that the trend of the number of moles of carbon precipitated as diamond is closely mirrored by the changes in the organic carbon species formate. In fact, a total of 529 mmol of carbon is precipitated as diamond, which comes from the destruction of 405 mmolal formate and 264 mmolal carbon from propionate, according to the reactions:


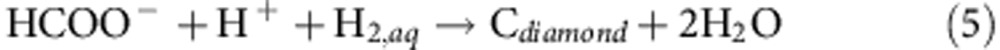



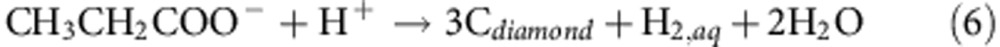


(as noted above, the small excess of organic carbon destroyed compared with diamond precipitated was converted to CO_2_ and Ca(HCO_3_)^+^). Although each of the reactions in equations (5) and (6) are redox reactions, the sum of these reactions is independent of the dissolved H_2,aq_ concentration and therefore of the *f*_O_2__. Consequently, the overall reaction precipitating diamond is independent of *f*_O_2__ and the *f*_O_2__ barely changes during the calculated reaction path in Fig. 1a.

## Discussion

Diamond precipitation in Fig. 1a,b is driven by pH drop and not by changes in redox conditions. The formation of diamond is a direct consequence of changes in aqueous fluid chemistry associated with metasomatic reactions involving silicate minerals under upper mantle conditions. Furthermore, in the same reacting chemical system, diamond dissolution can be driven by changes in fluid chemistry at almost constant redox states. For example, it can be seen in Figs 1b and 2c that diamond is predicted to partially dissolve during the interval of (−0.25<log*ξ*<+0.1) because of small fluctuations in solution chemistry associated with the disappearance of the Mg-rich carbonate solid solution. This predicted behaviour may mirror the often noted precipitation and dissolution features in natural diamonds^1^.

It is also interesting that the precipitation reactions for diamond in Figs 1 and 2 involved aqueous organic carbon species, not just the CO_2_ or CH_4_ previously pictured as the only possible sources of carbon for diamond formation. The involvement of aqueous organic species in our model arises, because it is predicted that at pressures above ∼3.0 GPa it is possible to have a complete equilibrium between all oxidation states of aqueous carbon species^29^. The proposed involvement of these organic carbon species in the formation of diamond has implications for the carbon isotopic composition of diamond^29^,^30^,^31^,^32^,^33^,^34^,^35^. For example, it can be expected that the carbon isotopic compositions of diamonds in equilibrium with an aqueous fluid will be a function of both pH and redox state. A change in either pH or redox state could, in principle, cause changes in the carbon isotopic composition of diamond, depending on the magnitude of the equilibrium fractionation factor between diamond and aqueous C-species. This inference is exactly analogous to that predicted for graphite under crustal hydrothermal conditions^36^. To date, most studies of the carbon isotopic compositions of diamonds invoke redox fluctuations^3^. However, in at least one instance, changes in the carbon isotopic composition of diamond have been suggested to be independent of redox changes^37^. The potential for a dependence on pH changes offers an alternate hypothesis that might be testable with experiments. Similar considerations involving a potential dependence on either redox state or pH variations during metasomatic reactions may apply to the large variability exhibited in the nitrogen isotopic compositions of individual diamonds^38^.

**Figure 3:**
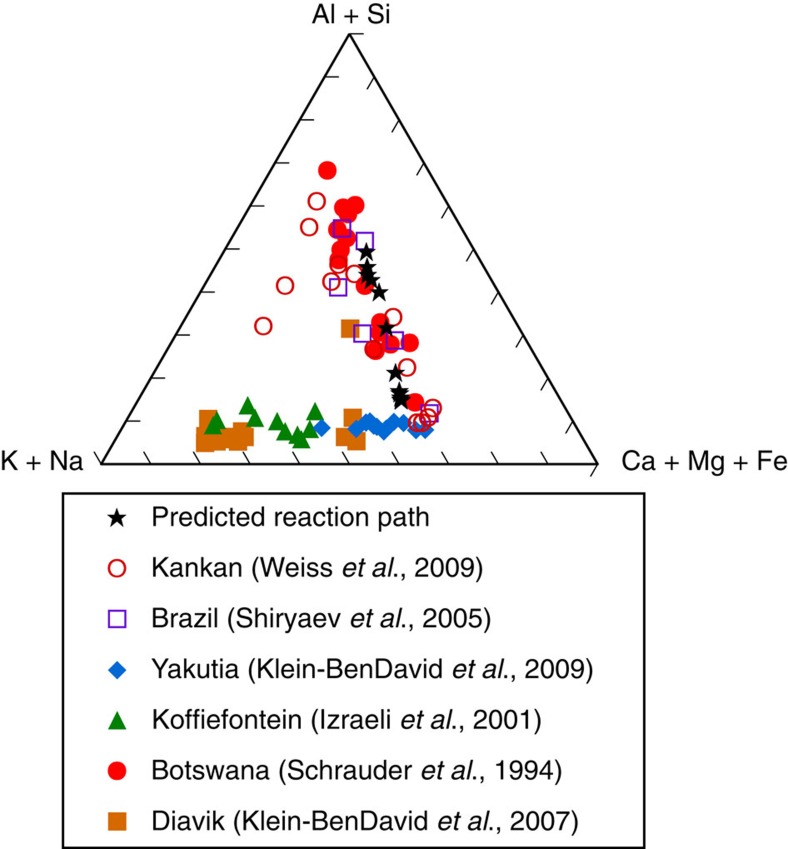
Predicted versus measured fluid chemistry during diamond formation. Theoretical evolution of the fluid chemistry from the model in Figs 1 and 2 (black stars). Measured compositions of fluid inclusions from worldwide diamonds^6^,^39^,^55^,^56^,^57^,^58^ (coloured symbols). The predicted fluid composition evolves from a carbonatitic end-member fluid towards the silicic end member of fluid composition from natural diamonds.

The predicted model evolution of the fluid chemistry during the precipitation of diamond is shown in Fig. 3 for comparison with worldwide trends in the chemistry of fluid inclusions in diamonds. The initial fluid chemistry is Ca-rich and Si-poor but progressively changes towards a silica-rich composition. After equilibration with the reactant eclogitic minerals, the final fluid chemistry (Supplementary Table 2) is extremely enriched in Al and Si, and depleted in Ca relative to the initial fluid. It can be seen in Fig. 3 that these changes are qualitatively consistent with one of the major trends in the chemistry of fluid inclusions in diamonds that extends from a carbonatitic fluid to a silicic fluid^6^. This trend has previously been suggested to be the result of equilibration with eclogites^39^. As a first step, given our current limited information about element solubility and speciation in high-pressure fluids, this approximation shows a reasonable agreement between predicted and actual fluid compositions.

Significant differences, however, are apparent when a more detailed comparison of individual elemental concentrations is made. For example, in Table 1 a more detailed comparison of the final eclogitic fluid composition from the model (Fig. 2) is made with the composition of a silicic fluid inclusion from a diamond from Brazil. To facilitate the comparison of elemental values, the fluid inclusion analysis was normalized to the molality of K in the predicted eclogitic model fluid. Excellent agreement can be seen between the concentrations of Si, Al and Na. However, the predicted model concentrations of Mg, Ca and Fe are either too high or too low by factors of 3.3, 1.9 and 6, respectively. This could be due to inaccurate speciation of these elements in the fluid model, for which we need more experimental data. The most significant difference that can be seen in Table 1 is that the wt.% CO_2_ is much higher in the model fluid (47%) than has been derived for the natural fluid inclusions (11%). A possible cause of this discrepancy might be the simplistic assumption of a unit activity coefficient for the the aqueous CO_2_ species in the model. Finally, it should be emphasized that the calculations plotted in Fig. 3 refer only to 900 °C and 5.0 GPa, whereas the fluid inclusion compositions of the natural diamonds plotted in Fig. 3 probably refer to a very wide range of temperatures, pressures and geologic environments^13^. Nevertheless, the overall comparison is encouraging. The model fluid speciation presented here is only a first step in modelling the chemistry of the evolution of fluids during diamond formation. Much more experimental and theoretical work needs to be done, to characterize the most important fluid species under such extreme conditions.

Our theoretical calculations provide a model clearly demonstrating that diamond can form by pH decreases during water–rock interaction under constant redox conditions in subcratonic lithospheric mantle environments. The theory implies that diamonds and their solid and fluid inclusions could be the natural, and perhaps even the common result of changes in water chemistry rather than redox changes alone. We wish to emphasize that our proposed pH-change mechanism for diamond formation represents a mechanism in addition to potential changes in redox, temperature and pressure^2^. Indeed, combined pH decreases and *f*_O_2__ increases could result in multiple cycles of diamond precipitation and dissolution, which might explain some observed diamond resorption features^1^ as well as the evidence for evolving fluid chemistry during individual diamond formation^40^.

The theoretical model developed here could also be used to investigate subcratonic lithospheric diamond formation in peridotites. Furthermore, the model could be expanded to include more sophisticated models of solid solution in the minerals, the partitioning of trace elements between the minerals and fluids, a much wider range of aqueous organic species, and liquid and crystalline hydrocarbons. It would then be possible to integrate quantitative theoretical models of evolving fluid chemistry with detailed studies of natural diamonds including their solid and fluid inclusions, their trends with time during diamond growth and their relationships to host eclogites and peridotites^7^,^8^,^9^,^27^,^41^,^42^. Ultimately, such integrated studies hold the potential for helping to unravel specific reaction histories of fluids in the deep Earth and in deep time. The theoretical model could also be used to predict the cooling behaviour of individual fluid inclusions to room temperature, including the formation of daughter crystals, enabling independent comparisons to be made with the extensive studies of fluid inclusion and their daughter crystals^5^,^10^.

## Methods

### Conceptual model of diamond formation

Diamond formation occurs particularly in the roots of continents known as mantle keels that extend from about 40 to 250 km depth. In this subcratonic mantle lithosphere environment, diamonds have formed at temperatures in the range of 900–1,400 °C and pressures of about 4.0–8.0 GPa (ref. 13). Diamonds are also described from ultra-high pressure metamorphic rocks referring to temperatures as low as 600 °C and pressures of >3 GPa (ref. 13). In the present study a model was constructed for subcratonic lithospheric mantle diamond formation in eclogite at 900 °C and 5.0 GPa. Although this temperature is at the low end of the range of recorded temperatures for subcratonic mantle diamond formation, the reactions described here should also apply to higher temperatures. It should be emphasized that the model is a generic one for the purpose of illustrating the role of pH in diamond-forming systems in which the fluid chemistry is coupled to the silicate rock mineralogy of eclogites.

The initial fluid was derived by reaction of fluids assumed to be low in chloride with a carbonated peridotitic mineral assemblage envisioned as passing through the carbonated mafic part of a subducting slab^24^. As a first approximation, the fluid was assumed to have moved through the slab at constant temperature and pressure, to infiltrate and react with a model metasedimentary eclogite consisting of clinopyroxene, garnet and coesite also based on previous model calculations^26^. Ideal site mixing was used for the silicate solid solution model.

### Calculation methods

The calculations referred to in Figs 1, 2, 3 were carried out with versions of the Fortran computer codes EQ3NR and EQ6 (refs 22, 23) modified for use at elevated pressures and temperatures. The code EQ3NR is used to set up a speciated initial fluid chemistry in equilibrium with a specified set of minerals that represents a model of a rock as described above. This fluid is then used in the code EQ6, to react with eclogitic minerals as described above. The irreversible mass transfer calculations were carried out assuming relative reaction rates of unity with excess amounts of each reactant mineral. In this mode of calculation, if the fluid reaches equilibrium with a given reactant mineral, it is subsequently assumed that the mineral remains in equilibrium with the fluid, although some of it may dissolve or precipitate during further reaction progress.

The two codes use a database of equilibrium constants calculated in advance using the DEW model^18^. The DEW model is based on an extension of the Helgeson–Kirkham–Flowers aqueous species equation of state, traditionally limited to an upper pressure of 0.5 GPa because of lack of knowledge of the dielectric constant of water at high pressures^43^,^44^. We have taken advantage of recent experimental and theoretical advances^19^,^20^ that enable this limitation to be overcome and we have further shown that the Helgeson–Kirkham–Flowers formalism can be applied to experimental solubility and aqueous speciation data for quartz, forsterite+enstatite, corundum, calcite and aragonite at pressures up to 6.0 GPa, enabling theoretical predictions of equilibrium constants involving aqueous inorganic and organic ions, neutral species and metal complexes up to pressures and temperatures of the upper mantle. Overall uncertainties in the DEW model may be of the order of ±0.3 to 0.5 units in equilibrium constants for aqueous species at high temperatures and pressures. It should be emphasized that all the aqueous species used in the calculations rest on experimental calibrations for as wide a range of temperatures or pressures as is available (see also below). Activity coefficients for neutral aqueous species were set to unity, whereas those for ions were approximated by the Debye–Hückel equation in which the long-range interaction parameters were calculated using dielectric constants and densities of water from the DEW model. Owing to a lack of sufficient data on mineral solubilities at elevated pressures as a function of ionic strength, the short-range interaction parameter in the Debye–Hückel equation was set to zero^21^.

### Comparison of model calculations and experimental data

Remarkably, little experimental data exist for mineral and silicate-rock solubilities in aqueous fluids at elevated temperatures and at pressures >3.0 GPa, in particular as the melting point of the rocks is approached^45^,^46^,^47^,^48^,^49^,^50^,^51^. Considerable uncertainties surround the nature of the fluid phase in equilibrium with silicate rocks under these conditions. In the present study, we calibrated our high temperature–pressure aqueous model using experimental measurements of a synthetic K-free eclogite at 900 °C and 5.0 GPa (ref. 50). The high solubilities of the elements Si, Mg, Fe and Al in these experiments almost certainly reflect complexes between metals and silicate as the temperatures approach the melting point of the eclogite^51^,^52^. Although the exact nature of these complexes remains uncertain and is the topic of intense interest^49^,^53^,^54^, as a first approximation we assumed they were 1:1 metal–bisilicate complexes, for example, Mg(HSiO_3_)^+^, because of the predicted abundance of the bisilicate anion HSiO_3_^−^ at high pressures^29^. Based on equilibrium constants calculated using the DEW model, the experimental eclogite solubility data were used to retrieve new equilibrium constants for the dissociation of the aqueous silica monomer to the bisilicate anion, and the bisilicate complexes of Mg, Fe, Ca and Al (Supplementary Table 1). Comparison between the experimental and calculated fluid composition is shown in Supplementary Fig. 1. Given the overall uncertainties in the experiments and the calculations, the agreement in Supplementary Fig. 1 was deemed to be sufficiently good for the application of the present study to diamond formation. Sensitivity calculations with and without metal–silicate complexes emphasized that the overall conclusion of the present study regarding the importance of a pH drop causing diamond formation did not depend on either the precise values of the equilibrium constants or even the existence of the metal–silicate complexes in the model. This is a consequence of the fundamental importance of reactions such as the one given in Eqn. (4) in controlling the pH of the fluids.

## Additional information

**How to cite this article:** Sverjensky, D. A. & Huang, F. Diamond formation due to a pH drop during fluid–rock interactions. *Nat. Commun.* 6:8702 doi: 10.1038/ncomms9702 (2015).

## Supplementary Material

Supplementary Information Supplementary Figure 1, Supplementary Tables 1-2, Supplementary Note 1 and Supplementary Reference.

## Figures and Tables

**Table 1:** Comparison of the predicted final fluid composition after reaction with eclogite at 900 °C and 5.0 GPa with the measured composition of a highly siliceous fluid inclusion*.

**Final model fluid**	**Parameter value**†	Siliceous fluid inclusion*	**Parameter value**†
Na	0.94	Na	0.84
K	1.69	K	1.69
Mg	0.24	Mg	0.78
Ca	3.07	Ca	1.62
Fe	0.14	Fe	0.87
Al	0.68	Al	1.0
Si	5.18	Si	5.2
Cl	0.10	Cl	0.7
S	0.18		
C	33.7		
pH	4.74		
log *f*_O_2__	−9.60		
Mole ratio of C/(C+H_2_O)	0.4	Mole ratio of C/(C+H_2_O)	0.1‡
wt.% H_2_O in fluid	32	wt.% H_2_O in fluid	Up to 40‡
wt.% CO_2_ in fluid	47	wt.% CO_2_ in fluid	Up to 11§

^*^Ref. 5555, inclusion number Sp.BR-1(inner) normalized to the K content of the model eclogitic fluid given above (1.69 m).

^†^Concentrations given in molality (m) unless otherwise noted.

^‡^Ref. 66.

^§^Calculated from the given mole ratio of C/(C+H_2_O) equal to 0.1 and the given upper limit of the wt.% H_2_O in fluid equal to 40%.

## References

[b1] Shirey S. B. *et al.* Diamonds and the geology of mantle carbon. Rev. Mineral. Geochem. 75, 355–421 (2013).

[b2] Stachel T. & Luth R. Diamond formation: where, when and how? Lithos 220, 200–220 (2015).

[b3] Cartigny P., Palot M., Thomassot E. & Harris J. W. Diamond formation: a stable isotope perspective. Annu. Rev. Earth Planet. Sci. 42, 699–732 (2014).

[b4] Frezzotti M.-L., Huizenga J.-M., Compagnoni R. & Selverstone J. Diamond formation by carbon saturation in C-O-H fluids during cold subduction of oceanic lithosphere. Geochim. Cosmochim. Acta 143, 68–86 (2014).

[b5] Frezzotti M. L., Selverstone J., Sharp Z. D. & Compagnoni R. Carbonate dissolution during subduction revealed by diamond-bearing rocks from the Alps. Nat. Geosci. 4, 703–706 (2011).

[b6] Klein-BenDavid O., Izraeli E. S., Hauri E. & Navon O. Fluid inclusions in diamonds from the Diavik mine, Canada, and the evolution of diamond-forming fluids. Geochim. Cosmochim. Acta 71, 723–744 (2007).

[b7] Tomlinson E. & Müller W. A snapshot of mantle metasomatism: trace element analysis of coexisting fluid (LA-ICP-MS) and silicate (SIMS) inclusions in fibrous diamonds. Earth Planet. Sci. Lett. 279, 362–372 (2009).

[b8] Tomlinson E. L., Jones A. P. & Harris J. W. Co-existing fluid and silicate inclusions in mantle diamond. Earth Planet. Sci. Lett. 250, 581–595 (2006).

[b9] Miller C. E., Kopylova M. & Smith E. Mineral inclusions in fibrous diamonds: constraints on cratonic mantle refertilization and diamond formation. Miner. Petrol. 108, 317–331 (2014).

[b10] Weiss Y., Kiflawi I. & Navon O. IR spectroscopy: quantitative determination of the mineralogy and bulk composition of fluid microinclusions in diamonds. Chem. Geol. 275, 26–34 (2010).

[b11] Bureau H. *et al.* The growth of fibrous, cloudy and polycrystalline diamonds. Geochim. Cosmochim. Acta 77, 202–214 (2012).

[b12] Weiss Y., McNeill J., Pearson D. G., Nowell G. M. & Ottley C. J. Highly saline fluids from a subducting slab as the source for fluid-rich diamonds. Nature 524, 339–342 (2015). 26289205 10.1038/nature14857

[b13] Stachel T. & Harris J. The origin of cratonic diamonds- constraints from mineral inclusions. Ore Geol. Rev. 34, 5–32 (2008).

[b14] Palyanov Y. N. *et al.* Mantle-slab interaction and redox mechanism of diamond formation. Proc. Natl Acad. Sci. 110, 20408–20413 (2013). 24297876 10.1073/pnas.1313340110 PMC3870714

[b15] Sokol A. G., Palyanova G. A., Palyanov Y. N., Tomilenko A. A. & Melenevsky V. N. Fluid regime and diamond formation in the reduced mantle: Experimental constraints. Geochim. Cosmochim. Acta 73, 5820–5834 (2009).

[b16] Zhang C. & Duan Z. A model for C-O-H fluid in the Earth's mantle. Geochim. Cosmochim. Acta 73, 2089–2102 (2009).

[b17] Luth R. W. Diamonds, eclogites, and the oxidation state of the Earth's mantle. Science 261, 66–68 (1993). 17750546 10.1126/science.261.5117.66

[b18] Sverjensky D. A., Harrison B. & Azzolini D. Water in the deep Earth: the dielectric constant and the solubilities of quartz and corundum to 60 kb and 1,200 °C. Geochim. Cosmochim. Acta 129, 125–145 (2014).

[b19] Pan D., Spanu L., Harrison B., Sverjensky D. A. & Galli G. The dielectric constant of water under extreme conditions and transport of carbonates in the deep Earth. Proc. Natl Acad. Sci. 110, 6646–6650 (2013). 23513225 10.1073/pnas.1221581110 PMC3637742

[b20] Facq S., Daniel I. & Sverjensky D. A. *In situ* Raman study and thermodynamic model of aqueous carbonate speciation in equilibrium with aragonite under subduction zone conditions. Geochim. Cosmochim. Acta 132, 375–390 (2014).

[b21] Manning C. E. Thermodynamic modeling of fluid-rock interaction at mid-crustal to upper-mantle conditions. Rev. Mineral. Geochem. 76, 135–164 (2013).

[b22] Wolery T. J. EQ3NR: A Computer Program for Geochemical Aqueous Speciation-Solubility Calculations, User's Guide and Documentation: UCRL-53414 Lawrence Livermore Lab., Univ. Calif. (1983).

[b23] Wolery T. J. EQ6---A computer Program For Reaction-Path Modelling of Aqueous Geochemical Systems: User's Guide and Documentation: UCRL-51 Lawrence Livermore Nat. Lab., Univ. Calif. (1984).

[b24] Kerrick D. M. & Connolly J. A. D. Metamorphic devolatilization of subducted oceanic metabasalts: implications for seismicity, arc magmatism and volatile recycling. Earth Planet. Sci. Lett. 189, 19–29 (2001).

[b25] Hacker B. R. H2O subduction beyond arcs. Geochem. Geophys. Geosyst. 9, Q03001 (2008).

[b26] Kerrick D. M. & Connolly J. A. D. Metamorphic devolatilization of subducted marine sediments and the transport of volatiles into the Earth's mantle. Nature 411, 293–296 (2001). 11357128 10.1038/35077056 PMC8127824

[b27] Howarth G. H. *et al.* The secondary origin of diamonds: multi-modal radiation tomography of diamondiferous mantle eclogites. Int. Geol. Rev. 56, 1172–1180 (2014).

[b28] Bowers T. S., Jackson K. J. & Helgeson H. C. Equilibrium Activity Diagrams Springer-Verlag (1984).

[b29] Sverjensky D. A., Stagno V. & Huang F. Important role for organic carbon in subduction-zone fluids in the deep carbon cycle. Nat. Geosci. 7, 909–913 (2014).

[b30] Navon O. in Proceedings of the 7th International Kimberlite Conference 2, 584–604Cape Town: Red Roof Design.

[b31] Kirkley M. B., Gurney J. J. & Levinson A. A. Age, origin, and emplacement of diamonds; scientific advances in the last decade. Gems Gemol. 27, 2–25 (1991).

[b32] Nisbet E. G., Mattey D. P. & Lowry D. Can diamonds be dead bacteria? Nature 367, 694 (1994).

[b33] Cartigny P. Stable isotopes and the origin of diamond. Elements 1, 79–84 (2005).

[b34] Mikhail S. *et al.* Empirical evidence for the fractionation of carbon isotopes between diamond and iron carbide from the Earth's mantle. Geochem. Geophys. Geosyst. 15, 855–866 (2014).

[b35] Thomassot E., Cartigny P., Harris J. & Viljoen K. F. Methane-related diamond crystallization in the Earth's mantle: stable isotope evidences from a single diamond-bearing xenolith. Earth Planet. Sci. Lett. 257, 362–371 (2007).

[b36] Ohmoto H. Systematics of sulfur and carbon isotopes in hydrothermal ore deposits. Econ. Geol. 67, 551–578 (1972).

[b37] Shiryaev A. A., Zubavichus Y. V., Veligzhanin A. A. & McCammon C. Local environment and valence state of iron in microinclusions in fibrous diamonds: X-ray absorption and Mössbauer data. Russ. Geol. Geophys. 51, 1262–1266 (2010).

[b38] Mikhail S. *et al.* Constraining the internal variability of the stable isotopes of carbon and nitrogen within mantle diamonds. Chem. Geol. 366, 14–23 (2014).

[b39] Weiss Y. *et al.* A new model for the evolution of diamond-forming fluids: Evidence from microinclusion-bearing diamonds from Kankan, Guinea. Lithos 112, 660–674 (2009).

[b40] Palot M., Pearson D., Stern R., Stachel T. & Harris J. Multiple growth events, processes and fluid sources involved in diamond genesis: a micro-analytical study of sulphide-bearing diamonds from Finsch mine, RSA. Geochim. Cosmochim. Acta 106, 51–70 (2013).

[b41] Weiss Y., Kiflawi I., Davies N. & Navon O. High-density fluids and th growth of monocrystalline diamonds. Geochim. Cosmochim. Acta 141, 145–159 (2014).

[b42] Klein-BenDavid O. *et al.* The sources and time-integrated evolution of diamond-forming fluids: trace elements and isotopic evidence. Geochim. Cosmochim. Acta 125, 146–169 (2014).

[b43] Shock E. L., Oelkers E. H., Johnson J. W., Sverjensky D. A. & Helgeson H. C. Calculation of the thermodynamic and transport properties of aqueous species at high pressures and temperatures: effective electrostatic radii to 1000 °C and 5 kb. Faraday Soc. Trans. 88, 803–826 (1992).

[b44] Shock E. L., Sassani D. C., Willis M. & Sverjensky D. A. Inorganic species in geologic fluids: correlations among standard molal thermodynamic properties of aqueous cations, oxyanions, acid oxyanions, oxyacids and hydroxide complexes. Geochim. Cosmochim. Acta 61, 907–950 (1997). 11541225 10.1016/s0016-7037(96)00339-0

[b45] Kessel R., Pettke T. & Fumagalli P. Melting of metasomatized peridotite at 4–6 GPa and up to 1200 °C: an experimental approach. Contr. Mineral. Petrol. 169, 1–19 (2015).

[b46] Kessel R., Schmidt M. W., Ulmer P. & Pettke T. Trace element signature of subduction-zone fluids, melts and supercritical liquids at 120–180 km depth. Nature 437, 724–727 (2005). 16193050 10.1038/nature03971

[b47] Kessel R., Ulmer P., Pettke T., Schmidt M. W. & Thompson A. B. A novel approach to determine high-pressure high-temperature fluid and melt compositions using diamond-trap experiments. Am. Miner 89, 1078–1086 (2004).

[b48] Hermann J. & Spandler C. J. Sediment melts at sub-arc depths: an experimental study. J. Petrol. 49, 717–740 (2008).

[b49] Zheng Y.-F. & Hermann J. Geochemistry of continental subduction-zone fluids. Earth Planets Space 66, 1–16 (2014).

[b50] Kessel R., Ulmer P., Pettke T., Schmidt M. W. & Thompson A. B. The water-basalt system at 4 to 6 GPa: Phase relations and second critical endpoint in a K-free eclogite at 700 to 1400 °C. Earth Planet. Sci. Lett. 237, 873–892 (2005).

[b51] Wohlers A., Manning C. E. & Thompson A. B. Experimental investigation of the solubility of albite and jadeite in H2O, with paragonite+quartz at 500 and 600 °C, and 1–2.25 GPa. Geochim. Cosmochim. Acta 75, 2924–2939 (2011).

[b52] Manning C. E., Antignano A. & Lin H. A. Premelting polymerization of crustal and mantle fluids, as indicated by the solubility of albite+paragonite+quartz in H2O at 1 GPa and 350–620 °C. Earth Planet. Sci. Lett. 292, 325–336 (2010).

[b53] Hermann J., Zheng Y.-F. & Rubatto D. Deep fluids in subducted continental crust. Elements 9, 281–287 (2013).

[b54] Newton R. C. & Manning C. E. Evidence for SiO2-NaCl complexing in H2O-NaCl solutions at high pressure and temperature. Geofluids doi: 10.1111/gfl.12153 (2015).

[b55] Shiryaev A., Izraeli E., Hauri E., Zakharchenko O. & Navon O. Chemical, optical and isotopic investigation of fibrous diamonds from Brazil. Russ. Geol. Geophys. 46, 1185–1201 (2005).

[b56] Klein-BenDavid O. *et al.* High-Mg carbonatitic microinclusions in some Yakutian diamonds‘ a new type of diamond-forming fluid. Lithos 112, 648–659 (2009).

[b57] Izraeli E. S., Harris J. W. & Navon O. Brine inclusions in diamonds: a new upper mantle fluid. Earth Planet. Sci. Lett. 187, 323–332 (2001).

[b58] Schrauder M. & Navon O. Hydrous and carbonatitic mantle fluids in fibrous diamonds from Jwaneng, Botswana. Geochim. Cosmochim. Acta 58, 761–771 (1994).

