# Supplementary material for: Diamond formation due to a pH drop during fluid–rock interactions
Source: Nat Commun. 2015 Nov 3;6:8702. doi: 10.1038/ncomms9702 (PMC4667645; doi:10.1038/ncomms9702)
Supplement: Supplementary Information — Supplementary Figure 1, Supplementary Tables 1-2, Supplementary Note 1 and Supplementary Reference. [file ncomms9702-s1.pdf]

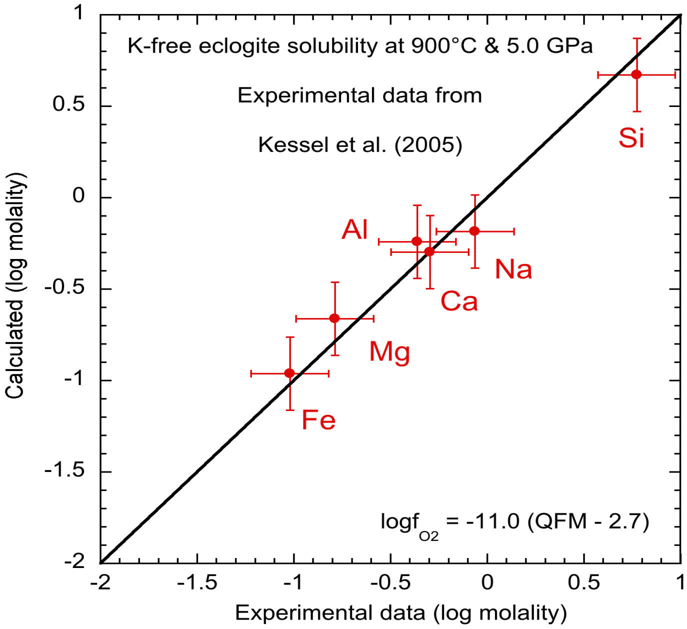

Supplementary Figure 1. Graphical comparison of the experimentally determined fluid composition for a synthetic K-free eclogite at 900°C and 5.0 GPa[1] with model calculations based on the results given in Supplementary Table 1.

Supplementary Table 1. Comparison of the experimentally determined fluid and mineral compositions for a synthetic K-free eclogite at 900°C and 5.0 GPa[1] with a calculated model fluid chemistry based on the given mineral compositions. There was also no Cl or C in the experimental fluid. The experimental data were used to retrieve values of the equilibrium constants for the following silicate and metal-silicate complexes:

$$HSiO_3^- + H^+ = SiO_2^0 + H_2O; \qquad \log K = +5.10$$
$$MgHSiO_3^+ + H^+ = Mg^{2+} + SiO_2^0 + H_2O; \qquad \log K = -0.27$$
$$FeHSiO_3^+ + H^+ = Fe^{2+} + SiO_2^0 + H_2O; \qquad \log K = -1.87$$
$$CaHSiO_3^+ + H^+ = Ca^{2+} + SiO_2^0 + H_2O; \qquad \log K = +0.83$$
$$AlHSiO_3^+ + H^+ = Al^{3+} + SiO_2^0 + H_2O; \qquad \log K = +1.70$$

EXPERIMENTAL DATA	Parameter value[a.]		MODEL FLUID	Parameter value[a.]
Na	0.86		Na	0.66
Mg	0.16		Mg	0.22
Ca	0.50		Ca	0.51
Fe	0.10		Fe	0.11
Al	0.43		Al	0.58
Si	5.9		Si	4.7
pH	not determined		pH	4.44
$\log f_{O2}$	not determined		$\log f_{O2}$	-11.0
MINERALS	COMPONENTS	log (MOLE ACTIVITY)		
CLINOPYROXENE	Diopside	-0.49		
	Jadeite	-0.26		
GARNET	Pyrope	-1.01		
	Almandine	-1.80		
	Grossular	-1.22		
COESITE	SiO_2	0.0		

[a.] Concentrations given in molality (m).

Supplementary Table 2. Summary of the initial and final fluid compositions and mineral assemblages at 900°C and 5.0 GPa. Full details of the aqueous speciation of all the elements at each step of the reaction progress are given in the reaction path output file (see below).

INITIAL FLUID	Parameter value[a.]		FINAL FLUID	Parameter value[a.]
Na	0.30		Na	0.94
K	1.69		K	1.69
Mg	0.054		Mg	0.24
Ca	3.21		Ca	3.07
Fe	0.016		Fe	0.14
Al	0.00009		Al	0.68
Si	0.89		Si	5.18
Cl	0.10		Cl	0.10
S	0.18		S	0.18
C	34.2		C	33.7
pH	4.74		pH	4.65
$\log f_{O2}$	-9.60		$\log f_{O2}$	-9.59
CO_2	26.1		CO_2	26.7
HCO_3^-	0.501		HCO_3^-	0.426
$Ca(HCO_3)^+$	3.146		$Ca(HCO_3)^+$	2.846
CO_3^{2-}	0.222		CO_3^{2-}	0.160
$CaCO_{3,aq}$	0.0057		$CaCO_{3,aq}$	0.004
$HCOO^-$	2.53		$HCOO^-$	2.12
CH_3COO^-	0.030		CH_3COO^-	0.025
$CH_3CH_2COO^-$	0.494		$CH_3CH_2COO^-$	0.406
CH_4	0.051		CH_4	0.049
REACTANT MINERALS	COMPONENTS	REACTANT MOLE FRACTION	FINAL PRODUCT MOLE FRACTION	
CLINOPYROXENE	Diopside	0.20	0.31	
	Hedenbergite	0.10	0.06	
	Jadeite	0.70	0.63	
GARNET	Pyrope	0.60	0.51	
	Almandine	0.30	0.32	
	Grossular	0.10	0.17	
COESITE	SiO_2	1.0	1.0	

[a.] Concentrations given in molality (m).

Supplementary Note 1

FULL REACTION PATH OUTPUT FILE PRODUCED BY EQ6

Model of the reaction of a fluid at 900°C and 5.0 GPa with an eclogite

EQ6, version 3245R100

Copyright (c) 1987 The Regents of the University of California,
Lawrence Livermore National Laboratory. All rights reserved.

Supported by EQLIB, version 3245R136

Copyright (c) 1987 The Regents of the University of California,
Lawrence Livermore National Laboratory. All rights reserved.

Run

```
--- reading the input file ---
Reaction of mantle fluid with metabasalt


Reaction with

Cpx (solid solution)
Coesite
Garnet (solid solution)

initially pptd minerals are removed before reaction path commences
(i.e. initial diamond and carbonate solid solution)


Closed SYSTEM CALCULATION



SOLID SOLUTIONS ACTIVATED;

HEXANE, OCTANE SUPPRESSED




  The bottom half of this input file is the pickup file produced by
eq3nr using the input file produced in April, 2007

endit.
    nmodl1=  2              nmodl2=  0
    tempc0=  9.00000E+02     jtemp=  0
       tk1=  0.00000E+00       tk2=  0.00000E+00       tk3=  0.00000E+00
    zistrt=  0.00000E+00     zimax=  2.00000E+01
     tstrt=  0.00000E+00    timemx=  0.00000E+00
    kstpmx=        1000      cplim=  0.00000E+00
    dzprnt=  1.00000E+38    dzprlg=  5.00000E-01    ksppmx= 10000
    dzplot=  1.00000E+38    dzpllg=  1.00000E+04    ksplmx= 10000
     ifile= 16
               1    2    3    4    5    6    7    8    9   10
  iopt1-10=    0    0    0    1    2    1    0    0    0    0
     11-20=    0    0    0    0    0    0    0    0    0    0
  iopr1-10=    0    0    1    0    1    0    0    1    0    0
 iopr11-20=    0    0    0    0    0    0    0    0    0    0
  iodb1-10=    0    0    0    0    0    0    0    0    0    0
     11-20=    0    0    0    0    0    0    0    0    0    0
     nxopt=  0
     nffg =  0
      nrct=     3
  reactant= CLINOPYROXENE(SS)
     jcode=  1               jreac=  0
      morr=  2.00000E+01      modr=  0.00000E+00
   JADEITE              7.00000E-01
   DIOPSIDE             2.00000E-01
   HEDENBERGITE         1.00000E-01
  endit.
       nsk=  0                  sk=  0.00000E+00        fk=  0.00000E+00
       nrk=  1                nrpk=  0
       rk1=  1.00000E+00       rk2=  0.00000E+00       rk3=  0.00000E+00
  reactant= GARNET(SS)
     jcode=  1               jreac=  0
```

```
      morr=  2.00000E+01      modr=  0.00000E+00
   PYROPE                6.00000E-01
   ALMANDINE             3.00000E-01
   GROSSULAR             1.00000E-01
  endit.
       nsk=  0                  sk=  0.00000E+00        fk=  0.00000E+00
       nrk=  1                nrpk=  0
       rk1=  1.00000E+00       rk2=  0.00000E+00       rk3=  0.00000E+00
  reactant= COESITE
     jcode=  0               jreac=  0
      morr=  2.00000E+01      modr=  0.00000E+00
       nsk=  0                  sk=  0.00000E+00        fk=  0.00000E+00
       nrk=  1                nrpk=  0
       rk1=  1.00000E+00       rk2=  0.00000E+00       rk3=  0.00000E+00
    dlzidp=  0.00000E+00
     tolbt=  1.00000E-10     toldl=  1.00000E-10      tolx=  0.00000E+00
    tolsat=  0.00000E+00    tolsst=  0.00000E+00
    screw1=  0.00000E+00    screw2=  0.00000E+00    screw3=  0.00000E+00
    screw4=  0.00000E+00    screw5=  0.00000E+00    screw6=  0.00000E+00
    zklogu=        0.000    zklogl=        0.000     zkfac=        0.000
    dlzmx1=  0.00000E+00    dlzmx2=  0.00000E+00    nordlm=  0
    itermx=  0              ntrymx=  0
    npslmx=  0              nsslmx=  0              ioscan=  0
Mantle fluid in slab based on Kerrick & Connolly (1998) at 900 C & 50.0 kbar

Charge balance for Ca++

Solid solutions



Cl- at 0.1 molal
logfO2 at QFM - 1.8 = -10.3

endit.
    uacion= CL-
    tempci=  9.00000E+02
     nxmod= 15
   species= MG(OH)+
      type=  0              option= -1              xlkmod=  0.00000E+00
   species= CASO4(AQ)
      type=  0              option= -1              xlkmod=  0.00000E+00
   species= BRUCITE
      type=  1              option= -1              xlkmod=  0.00000E+00
   species= FERROUS_OXIDE
      type=  1              option= -1              xlkmod=  0.00000E+00
   species= ENSTATITE-CL
      type=  1              option= -1              xlkmod=  0.00000E+00
   species= ENSTATITE-PR
      type=  1              option= -1              xlkmod=  0.00000E+00
   species= SEPIOLITE
      type=  1              option= -1              xlkmod=  0.00000E+00
   species= DIASPORE
      type=  1              option= -1              xlkmod=  0.00000E+00
   species= OCTANE(AQ)
      type=  0              option= -1              xlkmod=  0.00000E+00
   species= DECANE(AQ)
      type=  0              option= -1              xlkmod=  0.00000E+00
   species= ALO2(SIO2)-
      type=  0              option=  1              xlkmod= -1.50400E+00
   species= MG(HSIO3)+
      type=  0              option=  1              xlkmod= -3.12290E+00
   species= FE(HSIO3)+
      type=  0              option=  1              xlkmod= -4.72290E+00
   species= CA(HSIO3)+
      type=  0              option=  1              xlkmod= -2.02290E+00
   species= HSIO3-
      type=  0              option=  1              xlkmod=  6.81950E-01
     iopg1=  0               iopg2= -1               iopg3=  0
     iopg4=  0               iopg5=  1               iopg6=  0
     iopg7=  0               iopg8=  0               iopg9=  0
    iopg10=  0
       kct= 12                 ksq= 13                 kmt= 13
       kxt= 13                kdim= 13                kprs=  0
component            moles            moles aqueous
   O                  1.304946520890770E+02       0.000000000000000E+00
```

```
   NA                3.000000014091010E-01       0.000000000000000E+00
   K                 1.700000000832560E+00       0.000000000000000E+00
   CA                3.286801580213060E+00       0.000000000000000E+00
   MG                3.000000180787070E-01       0.000000000000000E+00
   AL                9.482192408130389E-05       0.000000000000000E+00
   SI                9.000000087014421E-01       0.000000000000000E+00
   H                 1.223732579198800E+02       0.000000000000000E+00
   C                 3.500000642997010E+01       0.000000000000000E+00
   CL                1.000000009737470E-01       0.000000000000000E+00
   S                 1.856289652534230E-01       0.000000000000000E+00
   FE                5.588966078488220E-02       0.000000000000000E+00
   electr            2.131708884967050E-07
   H2O               H2O                1.744360912091830E+00
   NA+               NA+               -5.884424373671960E-01
   K+                K+                 1.634932713998970E-01
   CA++              CA++              -3.825389630303470E+00
   MG++              MG++              -4.017433709988790E+00
   AL+++             AL+++             -1.828471453116840E+01
   SIO2(AQ)          SIO2(AQ)          -5.838005476294899E-01
   H+                H+                -4.448269293707500E+00
   CO3--             CO3--             -5.724925771302690E-01
   CL-               CL-               -1.075699968166110E+00
   SO4--             SO4--             -3.454142850945010E+00
   FE++              FE++              -6.340321982570260E+00
   O2(G)             O2(G)             -9.600000000000000E+00

--- the input file has been successfully read ---

--- reading the data1 file ---


                    --- list of solid solutions ---

 1  (NA,K)-SANIDINE          no.components=   2
         model type=   0  (ideal solution                )
 2  PLAGIOCLASE              no.components=   2
         model type=   0  (ideal solution                )
 3  ORTHOPYROXENE(SS)        no.components=   2
         model type=   0  (ideal solution                )
 4  OLIVINE                  no.components=   2
         model type=   0  (ideal solution                )
 5  BIOTITE                  no.components=   4
         model type=   0  (ideal solution                )
 6  CA-SMECTITE              no.components=   3
         model type=   0  (ideal solution                )
 7  NA-SMECTITE              no.components=   3
         model type=   0  (ideal solution                )
 8  TALC(SS)                 no.components=   3
         model type=   0  (ideal solution                )
 9  CLINOPYROXENE(SS)        no.components=   3
         model type=   0  (ideal solution                )
10  GARNET(SS)               no.components=   3
         model type=   0  (ideal solution                )
11  CA-AMPHIBOLE(SS)         no.components=   3
         model type=   0  (ideal solution                )
12  CHLORITE                 no.components=   3
         model type=   0  (ideal solution                )
13  MAGNETITE(SS)            no.components=   2
         model type=   0  (ideal solution                )
14  SPHALERITE(SS)           no.components=   2
         model type=   0  (ideal solution                )
15  CALCITE(SS)              no.components=   4
         model type=   0  (ideal solution                )
16  APATITE(SS)              no.components=   3
         model type=   0  (ideal solution                )

--- the data1 file has been successfully read ---

                      1.000  ALO2(SIO2)-
                   +  4.000  H+
                          ==
                      2.000  H2O
                   +  1.000  AL+++
                   +  1.000  SIO2(AQ)

      log k of the above reaction at    900.000
```

```
       deg celsius and  50000.000 bars was changed from
          3.2040 to     1.7000


                      1.000  MG(HSIO3)+
                   +  1.000  H+
                          ==
                      1.000  H2O
                   +  1.000  MG++
                   +  1.000  SIO2(AQ)

      log k of the above reaction at    900.000
       deg celsius and  50000.000 bars was changed from
          2.8529 to    -0.2700


                      1.000  FE(HSIO3)+
                   +  1.000  H+
                          ==
                      1.000  H2O
                   +  1.000  SIO2(AQ)
                   +  1.000  FE++

      log k of the above reaction at    900.000
       deg celsius and  50000.000 bars was changed from
          2.8529 to    -1.8700


                      1.000  CA(HSIO3)+
                   +  1.000  H+
                          ==
                      1.000  H2O
                   +  1.000  CA++
                   +  1.000  SIO2(AQ)

      log k of the above reaction at    900.000
       deg celsius and  50000.000 bars was changed from
          2.8529 to     0.8300


                      1.000  HSIO3-
                   +  1.000  H+
                          ==
                      1.000  H2O
                   +  1.000  SIO2(AQ)

      log k of the above reaction at    900.000
       deg celsius and  50000.000 bars was changed from
          4.4581 to     5.1400

the species MG(OH)+                  has been user-suppressed

the species CASO4(AQ)                has been user-suppressed

the species BRUCITE                  has been user-suppressed

the species FERROUS_OXIDE            has been user-suppressed

the species ENSTATITE-CL             has been user-suppressed

the species ENSTATITE-PR             has been user-suppressed

the species SEPIOLITE                has been user-suppressed

the species DIASPORE                 has been user-suppressed

* note- suppress species OCTANE(AQ)               was not among
      the loaded aqueous species          (eqlib/supprs)

* note- suppress species DECANE(AQ)               was not among
      the loaded aqueous species          (eqlib/supprs)

  mineral OLIVINE              was assumed ideal - inconsistent with data in apx array - fix this
  mineral CHLORITE             was assumed ideal - inconsistent with data in apx array - fix this
```

```
eeeee  qqq   666
e     q   q 6
eeee  q   q 6666
e     q q q 6   6
eeeee  qqq   666
          q
```

eq6.3245R100
 supported by eqlib.3245R136

Reaction of mantle fluid with metabasalt

Reaction with

Cpx (solid solution)
Coesite
Garnet (solid solution)

initially pptd minerals are removed before reaction path commences
(i.e. initial diamond and carbonate solid solution)

Closed SYSTEM CALCULATION

SOLID SOLUTIONS ACTIVATED;

HEXANE, OCTANE SUPPRESSED

The bottom half of this input file is the pickup file produced by
eq3nr using the input file produced in April, 2007

Mantle fluid in slab based on Kerrick & Connolly (1998) at 900 C & 50.0 kbar

Charge balance for Ca++

Solid solutions

Cl- at 0.1 molal
logfO2 at QFM - 1.8 = -10.3

DATA FILE DATA0.3230U03
  HIGH PRESSURE DATA FILE
  LAST REVISED OCTOBER, 2013

the activity coefficients of aqueous solute species
and the activity of water are calculated according to
b-dot equation plus others

no. of elements in the data base = 28
no. of elements dimensioned for = 70
no. of active elements = 12

no. of aqueous species dimensioned for = 750
no. of aqueous species loaded = 90
no. of active aqueous species = 67

no. of aqueous reactions dimensioned for = 679

```
         no. of aqueous reactions loaded =  61
         no. of active aqueous reactions =  55


            no. of pure minerals dimensioned for = 750
            no. of pure minerals loaded =  81
            no. of active pure minerals =  60

            no. of gases dimensioned for =  15
            no. of gases loaded =   7
            no. of active gases =   7

               no. of solid solutions in the data base =  16
               no. of solid solutions dimensioned for =  20
               no. of active solid solutions =   6



---listing of species and reactions ---

      temperature=    900.000 degrees celsius
      pressure=   50000.0     bars

       --- strict basis species ---

                             H2O
                             NA+
                             K+
                             CA++
                             MG++
                             AL+++
                             SIO2(AQ)
                             H+
                             CO3--
                             CL-
                             SO4--
                             FE++


       --- aqueous species dissociation reactions ---

--------------------------------------------------

                      1.000  O2(AQ)
                          ==
                      1.000  O2(G)

         log k=       6.1252

--------------------------------------------------

                      2.000  H2(AQ)
                   +  1.000  O2(G)
                          ==
                      2.000  H2O

         log k=      13.3705

--------------------------------------------------

                      1.000  CH4(AQ)
                   +  2.000  O2(G)
                          ==
                      1.000  H2O
                   +  2.000  H+
                   +  1.000  CO3--

         log k=       9.0893

--------------------------------------------------

                      1.000  HS-
                   +  2.000  O2(G)
                          ==
                      1.000  H+
                   +  1.000  SO4--
```

```
        log k=      11.3742

--------------------------------------------------

                 1.000  FE+++
              +  0.500  H2O
                     ==
                 1.000  H+
              +  1.000  FE++
              +  0.250  O2(G)

        log k=       1.2474

--------------------------------------------------

                 1.000  HCO3-
                     ==
                 1.000  H+
              +  1.000  CO3--

        log k=      -6.0544

--------------------------------------------------

                 1.000  CLO4-
                     ==
                 1.000  CL-
              +  2.000  O2(G)

        log k=      21.5009

--------------------------------------------------

                 1.000  OH-
              +  1.000  H+
                     ==
                 1.000  H2O

        log k=       5.3306

--------------------------------------------------

                 1.000  HCOO-
              +  0.500  O2(G)
                     ==
                 1.000  H+
              +  1.000  CO3--

        log k=      -1.9553

--------------------------------------------------

                 1.000  CH3COO-
              +  2.000  O2(G)
                     ==
                 3.000  H+
              +  2.000  CO3--

        log k=       2.9698

--------------------------------------------------

                 1.000  CH3CH2COO-
              +  3.500  O2(G)
                     ==
                 5.000  H+
              +  3.000  CO3--

        log k=       4.7380

--------------------------------------------------

                 1.000  CO(AQ)
              +  1.000  H2O
              +  0.500  O2(G)
```

```
                      ==
                  2.000  H+
               +  1.000  CO3--

         log k=      -5.4160

--------------------------------------------------

                  1.000  ETHANE(AQ)
               +  3.500  O2(G)
                      ==
                  1.000  H2O
               +  4.000  H+
               +  2.000  CO3--

         log k=      14.4526

--------------------------------------------------

                  1.000  ETHYLENE(AQ)
               +  3.000  O2(G)
                      ==
                  4.000  H+
               +  2.000  CO3--

         log k=      12.3155

--------------------------------------------------

                  1.000  PROPANE(AQ)
               +  5.000  O2(G)
                      ==
                  1.000  H2O
               +  6.000  H+
               +  3.000  CO3--

         log k=      19.6880

--------------------------------------------------

                  0.500  HEXANE(AQ)
               +  4.750  O2(G)
                      ==
                  0.500  H2O
               +  6.000  H+
               +  3.000  CO3--

         log k=      17.6402

--------------------------------------------------

                  1.000  BENZENE(AQ)
               +  3.000  H2O
               +  7.500  O2(G)
                      ==
                 12.000  H+
               +  6.000  CO3--

         log k=      15.5549

--------------------------------------------------

                  1.000  TOLUENE(AQ)
               +  3.000  H2O
               +  9.000  O2(G)
                      ==
                 14.000  H+
               +  7.000  CO3--

         log k=      20.9416

--------------------------------------------------

                  1.000  SI2O4(AQ)
                      ==
                  2.000  SIO2(AQ)
```

```
        log k=       0.8686

--------------------------------------------------

                     1.000  AL(OH)++
                  +  1.000  H+
                         ==
                     1.000  H2O
                  +  1.000  AL+++

        log k=      26.1262

--------------------------------------------------

                     1.000  AL(OH)2+
                  +  2.000  H+
                         ==
                     2.000  H2O
                  +  1.000  AL+++

        log k=      37.6748

--------------------------------------------------

                     1.000  HALO2(AQ)
                  +  3.000  H+
                         ==
                     2.000  H2O
                  +  1.000  AL+++

        log k=      37.6748

--------------------------------------------------

                     1.000  ALO2-
                  +  4.000  H+
                         ==
                     2.000  H2O
                  +  1.000  AL+++

        log k=       3.2040

--------------------------------------------------

                     1.000  ALO2(SIO2)-
                  +  4.000  H+
                         ==
                     2.000  H2O
                  +  1.000  AL+++
                  +  1.000  SIO2(AQ)

        log k=       1.7000

--------------------------------------------------

                     1.000  CACL+
                         ==
                     1.000  CA++
                  +  1.000  CL-

        log k=      -2.5351

--------------------------------------------------

                     1.000  CACL2(AQ)
                         ==
                     1.000  CA++
                  +  2.000  CL-

        log k=      -1.4848

--------------------------------------------------

                     1.000  CACO3(AQ)
                         ==
```

```
                 1.000  CA++
              +  1.000  CO3--

        log k=      -4.7841

--------------------------------------------------

                 1.000  CA(HCO3)+
                     ==
                 1.000  CA++
              +  1.000  H+
              +  1.000  CO3--

        log k=     -12.0220

--------------------------------------------------

                 1.000  CA(OH)+
              +  1.000  H+
                     ==
                 1.000  H2O
              +  1.000  CA++

        log k=       3.3483

--------------------------------------------------

                 1.000  CA(HSIO3)+
              +  1.000  H+
                     ==
                 1.000  H2O
              +  1.000  CA++
              +  1.000  SIO2(AQ)

        log k=       0.8300

--------------------------------------------------

                 1.000  FECL+
                     ==
                 1.000  CL-
              +  1.000  FE++

        log k=      -2.1242

--------------------------------------------------

                 1.000  FECL2(AQ)
                     ==
                 2.000  CL-
              +  1.000  FE++

        log k=      -6.7578

--------------------------------------------------

                 1.000  FECL++
              +  0.500  H2O
                     ==
                 1.000  H+
              +  1.000  CL-
              +  1.000  FE++
              +  0.250  O2(G)

        log k=      -4.3927

--------------------------------------------------

                 1.000  FE(HSIO3)+
              +  1.000  H+
                     ==
                 1.000  H2O
              +  1.000  SIO2(AQ)
              +  1.000  FE++

        log k=      -1.8700
```

```
-------------------------------------------------

                    1.000  KCL(AQ)
                        ==
                    1.000  K+
                 +  1.000  CL-

        log k=      0.2866

-------------------------------------------------

                    1.000  KOH
                 +  1.000  H+
                        ==
                    1.000  H2O
                 +  1.000  K+

        log k=      5.2512

-------------------------------------------------

                    1.000  KSO4-
                        ==
                    1.000  K+
                 +  1.000  SO4--

        log k=      0.2475

-------------------------------------------------

                    1.000  MGCL+
                        ==
                    1.000  MG++
                 +  1.000  CL-

        log k=     -1.9870

-------------------------------------------------

                    1.000  MGCO3(AQ)
                        ==
                    1.000  MG++
                 +  1.000  CO3--

        log k=      3.0303

-------------------------------------------------

                    1.000  MG(HCO3)+
                        ==
                    1.000  MG++
                 +  1.000  H+
                 +  1.000  CO3--

        log k=     -9.3832

-------------------------------------------------

                    1.000  MG(HSIO3)+
                 +  1.000  H+
                        ==
                    1.000  H2O
                 +  1.000  MG++
                 +  1.000  SIO2(AQ)

        log k=     -0.2700

-------------------------------------------------

                    1.000  MGSO4(AQ)
                        ==
                    1.000  MG++
                 +  1.000  SO4--

        log k=     -2.9989
```

```
--------------------------------------------------

                     1.000  NACL(AQ)
                         ==
                     1.000  NA+
                  +  1.000  CL-

        log k=       0.7838

--------------------------------------------------

                     1.000  NACO3-
                         ==
                     1.000  NA+
                  +  1.000  CO3--

        log k=       0.7450

--------------------------------------------------

                     1.000  NAHCO3(AQ)
                         ==
                     1.000  NA+
                  +  1.000  H+
                  +  1.000  CO3--

        log k=      -5.4279

--------------------------------------------------

                     1.000  NAHSIO3(AQ)
                  +  1.000  H+
                         ==
                     1.000  H2O
                  +  1.000  NA+
                  +  1.000  SIO2(AQ)

        log k=       7.0716

--------------------------------------------------

                     1.000  NAOH(AQ)
                  +  1.000  H+
                         ==
                     1.000  H2O
                  +  1.000  NA+

        log k=       5.3412

--------------------------------------------------

                     1.000  HSIO3-
                  +  1.000  H+
                         ==
                     1.000  H2O
                  +  1.000  SIO2(AQ)

        log k=       5.1400

--------------------------------------------------

                     1.000  HSO4-
                         ==
                     1.000  H+
                  +  1.000  SO4--

        log k=      -6.5088

--------------------------------------------------

                     1.000  H2S(AQ)
                  +  2.000  O2(G)
                         ==
                     2.000  H+
                  +  1.000  SO4--
```

```
         log k=       5.7603

--------------------------------------------------

                      1.000  CO2(AQ)
                   +  1.000  H2O
                          ==
                      2.000  H+
                   +  1.000  CO3--

         log k=     -12.8298

--------------------------------------------------

                      1.000  HCL(AQ)
                          ==
                      1.000  H+
                   +  1.000  CL-

         log k=      -2.5664

--------------------------------------------------

                      1.000  CH3CH2COOH
                   +  3.500  O2(G)
                          ==
                      6.000  H+
                   +  3.000  CO3--

         log k=       3.6584

--------------------------------------------------

                      1.000  CH3COOH
                   +  2.000  O2(G)
                          ==
                      4.000  H+
                   +  2.000  CO3--

         log k=      -0.8476

--------------------------------------------------

                      1.000  HCOOH
                   +  0.500  O2(G)
                          ==
                      2.000  H+
                   +  1.000  CO3--

         log k=      -4.1088

--------------------------------------------------

       --- mineral dissolution reactions ----

--------------------------------------------------

                      1.000  ALUNITE
                   +  6.000  H+
                          ==
                      6.000  H2O
                   +  1.000  K+
                   +  3.000  AL+++
                   +  2.000  SO4--

         log k=       7.9764

--------------------------------------------------

                      1.000  DIAMOND
                   +  1.000  H2O
                   +  1.000  O2(G)
                          ==
                      2.000  H+
                   +  1.000  CO3--
```

```
        log k=      -1.8086

-------------------------------------------------

                   1.000  MAGNETITE
                +  6.000  H+
                       ==
                   3.000  H2O
                +  3.000  FE++
                +  0.500  O2(G)

        log k=       2.6833

-------------------------------------------------

                   1.000  CORUNDUM
                +  6.000  H+
                       ==
                   3.000  H2O
                +  2.000  AL+++

        log k=      -6.2606

-------------------------------------------------

                   1.000  HEMATITE
                +  4.000  H+
                       ==
                   2.000  H2O
                +  2.000  FE++
                +  0.500  O2(G)

        log k=       0.7175

-------------------------------------------------

                   1.000  PERICLASE
                +  2.000  H+
                       ==
                   1.000  H2O
                +  1.000  MG++

        log k=       5.1789

-------------------------------------------------

                   1.000  LIME
                +  2.000  H+
                       ==
                   1.000  H2O
                +  1.000  CA++

        log k=       9.8317

-------------------------------------------------

                   1.000  SPINEL
                +  8.000  H+
                       ==
                   4.000  H2O
                +  1.000  MG++
                +  2.000  AL+++

        log k=      -1.8251

-------------------------------------------------

                   1.000  ARAGONITE
                       ==
                   1.000  CA++
                +  1.000  CO3--

        log k=      -6.5865

-------------------------------------------------
```

```
                  1.000  DOLOMITE
                      ==
                  1.000  CA++
               +  1.000  MG++
               +  2.000  CO3--

        log k=     -14.4866

--------------------------------------------------

                  1.000  ANDALUSITE
               +  6.000  H+
                      ==
                  3.000  H2O
               +  2.000  AL+++
               +  1.000  SIO2(AQ)

        log k=      -5.1557

--------------------------------------------------

                  1.000  KYANITE
               +  6.000  H+
                      ==
                  3.000  H2O
               +  2.000  AL+++
               +  1.000  SIO2(AQ)

        log k=      -6.5126

--------------------------------------------------

                  1.000  SILLIMANITE
               +  6.000  H+
                      ==
                  3.000  H2O
               +  2.000  AL+++
               +  1.000  SIO2(AQ)

        log k=      -5.6552

--------------------------------------------------

                  1.000  GLAUCOPHANE
               + 14.000  H+
                      ==
                  8.000  H2O
               +  2.000  NA+
               +  3.000  MG++
               +  2.000  AL+++
               +  8.000  SIO2(AQ)

        log k=      15.4088

--------------------------------------------------

                  1.000  LAWSONITE
               +  8.000  H+
                      ==
                  6.000  H2O
               +  1.000  CA++
               +  2.000  AL+++
               +  2.000  SIO2(AQ)

        log k=      -1.8597

--------------------------------------------------

                  1.000  PUMPELLYITE
               + 25.000  H+
                      ==
                 16.000  H2O
               +  4.000  CA++
               +  1.000  MG++
               +  5.000  AL+++
```

```
                   +  6.000  SIO2(AQ)

         log k=       9.5615

--------------------------------------------------

                      1.000  ZOISITE
                   + 13.000  H+
                          ==
                      7.000  H2O
                   +  2.000  CA++
                   +  3.000  AL+++
                   +  3.000  SIO2(AQ)

         log k=       0.2795

--------------------------------------------------

                      1.000  CLINOZOISITE
                   + 13.000  H+
                          ==
                      7.000  H2O
                   +  2.000  CA++
                   +  3.000  AL+++
                   +  3.000  SIO2(AQ)

         log k=       0.7770

--------------------------------------------------

                      1.000  MONTICELLITE
                   +  4.000  H+
                          ==
                      2.000  H2O
                   +  2.000  CA++
                   +  1.000  SIO2(AQ)

         log k=      16.5497

--------------------------------------------------

                      1.000  MERWINITE
                   +  8.000  H+
                          ==
                      4.000  H2O
                   +  3.000  CA++
                   +  1.000  MG++
                   +  2.000  SIO2(AQ)

         log k=      23.3934

--------------------------------------------------

                      1.000  CHRYSOTILE
                   +  6.000  H+
                          ==
                      5.000  H2O
                   +  3.000  MG++
                   +  2.000  SIO2(AQ)

         log k=      12.6499

--------------------------------------------------

                      1.000  CA-AL-PYROXENE
                   +  8.000  H+
                          ==
                      4.000  H2O
                   +  1.000  CA++
                   +  2.000  AL+++
                   +  1.000  SIO2(AQ)

         log k=      -0.3043

--------------------------------------------------
```

```
                  1.000  WOLLASTONITE
               +  2.000  H+
                      ==
                  1.000  H2O
               +  1.000  CA++
               +  1.000  SIO2(AQ)

         log k=       6.4274

--------------------------------------------------

                  1.000  PSEUDOWOLLASTONITE
               +  2.000  H+
                      ==
                  1.000  H2O
               +  1.000  CA++
               +  1.000  SIO2(AQ)

         log k=       6.5238

--------------------------------------------------

                  1.000  TREMOLITE
               + 14.000  H+
                      ==
                  8.000  H2O
               +  2.000  CA++
               +  5.000  MG++
               +  8.000  SIO2(AQ)

         log k=      29.0710

--------------------------------------------------

                  1.000  ANTHOPHYLLITE
               + 14.000  H+
                      ==
                  8.000  H2O
               +  7.000  MG++
               +  8.000  SIO2(AQ)

         log k=      27.2555

--------------------------------------------------

                  1.000  CORDIERITE
               + 16.000  H+
                      ==
                  8.000  H2O
               +  2.000  MG++
               +  4.000  AL+++
               +  5.000  SIO2(AQ)

         log k=       3.1104

--------------------------------------------------

                  1.000  K-FELDSPAR
               +  4.000  H+
                      ==
                  2.000  H2O
               +  1.000  K+
               +  1.000  AL+++
               +  3.000  SIO2(AQ)

         log k=       2.8349

--------------------------------------------------

                  1.000  ALBITE
               +  4.000  H+
                      ==
                  2.000  H2O
               +  1.000  NA+
               +  1.000  AL+++
               +  3.000  SIO2(AQ)
```

```
        log k=       3.3787

--------------------------------------------------

                    1.000  ANORTHITE
                 +  8.000  H+
                       ==
                    4.000  H2O
                 +  1.000  CA++
                 +  2.000  AL+++
                 +  2.000  SIO2(AQ)

        log k=       1.2932

--------------------------------------------------

                    1.000  GEHLENITE
                 + 10.000  H+
                       ==
                    5.000  H2O
                 +  2.000  CA++
                 +  2.000  AL+++
                 +  1.000  SIO2(AQ)

        log k=       8.3343

--------------------------------------------------

                    1.000  KAOLINITE
                 +  6.000  H+
                       ==
                    5.000  H2O
                 +  2.000  AL+++
                 +  2.000  SIO2(AQ)

        log k=      -4.1176

--------------------------------------------------

                    1.000  ANTIGORITE
                 + 96.000  H+
                       ==
                   79.000  H2O
                 + 48.000  MG++
                 + 34.000  SIO2(AQ)

        log k=     191.9396

--------------------------------------------------

                    1.000  PYROPHYLLITE
                 +  6.000  H+
                       ==
                    4.000  H2O
                 +  2.000  AL+++
                 +  4.000  SIO2(AQ)

        log k=      -3.9521

--------------------------------------------------

                    1.000  TALC
                 +  6.000  H+
                       ==
                    4.000  H2O
                 +  3.000  MG++
                 +  4.000  SIO2(AQ)

        log k=      11.9853

--------------------------------------------------

                    1.000  MUSCOVITE
                 + 10.000  H+
                       ==
```

```
             6.000  H2O
          +  1.000  K+
          +  3.000  AL+++
          +  3.000  SIO2(AQ)

 log k=      -4.9944

--------------------------------------------------

             1.000  PARAGONITE
          + 10.000  H+
                ==
             6.000  H2O
          +  1.000  NA+
          +  3.000  AL+++
          +  3.000  SIO2(AQ)

 log k=      -4.0653

--------------------------------------------------

             1.000  MARGARITE
          + 14.000  H+
                ==
             8.000  H2O
          +  1.000  CA++
          +  4.000  AL+++
          +  2.000  SIO2(AQ)

 log k=      -6.4908

--------------------------------------------------

             1.000  PREHNITE
          + 10.000  H+
                ==
             6.000  H2O
          +  2.000  CA++
          +  2.000  AL+++
          +  3.000  SIO2(AQ)

 log k=       7.3796

--------------------------------------------------

             1.000  CLINOCHLORE
          + 16.000  H+
                ==
            12.000  H2O
          +  5.000  MG++
          +  2.000  AL+++
          +  3.000  SIO2(AQ)

 log k=      12.9158

--------------------------------------------------

             1.000  MEIONITE
          + 24.000  H+
                ==
            12.000  H2O
          +  4.000  CA++
          +  6.000  AL+++
          +  6.000  SIO2(AQ)
          +  1.000  CO3--

 log k=      -2.4318

--------------------------------------------------

             1.000  COESITE
                ==
             1.000  SIO2(AQ)

 log k=       0.2551
```

```
--------------------------------------------------

                    1.000  IRON
                 +  2.000  H+
                 +  0.500  O2(G)
                        ==
                    1.000  H2O
                 +  1.000  FE++

         log k=      10.0605

--------------------------------------------------

                    1.000  GRAPHITE
                 +  1.000  H2O
                 +  1.000  O2(G)
                        ==
                    2.000  H+
                 +  1.000  CO3--

         log k=      -1.7447

--------------------------------------------------

                    1.000  PYRRHOTITE
                 +  2.000  O2(G)
                        ==
                    1.000  SO4--
                 +  1.000  FE++

         log k=       6.8175

--------------------------------------------------

                    1.000  PYRITE
                 +  1.000  H2O
                 +  3.500  O2(G)
                        ==
                    2.000  H+
                 +  2.000  SO4--
                 +  1.000  FE++

         log k=       7.0871

--------------------------------------------------

                    1.000  GIBBSITE
                 +  3.000  H+
                        ==
                    3.000  H2O
                 +  1.000  AL+++

         log k=      -2.3055

--------------------------------------------------

                    1.000  PORTLANDITE
                 +  2.000  H+
                        ==
                    2.000  H2O
                 +  1.000  CA++

         log k=       8.2773

--------------------------------------------------

                    1.000  HALITE
                        ==
                    1.000  NA+
                 +  1.000  CL-

         log k=       2.5348

--------------------------------------------------

                    1.000  SYLVITE
```

```
                    ==
                1.000  K+
             +  1.000  CL-

        log k=       2.8317

--------------------------------------------------

                1.000  HYDROMAGNESITE
             +  2.000  H+
                    ==
                6.000  H2O
             +  5.000  MG++
             +  4.000  CO3--

        log k=     -20.8259

--------------------------------------------------

                1.000  NESQUEHONITE
                    ==
                3.000  H2O
             +  1.000  MG++
             +  1.000  CO3--

        log k=      -1.5307

--------------------------------------------------

                1.000  ANHYDRITE
                    ==
                1.000  CA++
             +  1.000  SO4--

        log k=      -5.9190

--------------------------------------------------

                1.000  QUARTZ-ALPHA
                    ==
                1.000  SIO2(AQ)

        log k=       0.3740

--------------------------------------------------

                1.000  QUARTZ-BETA
                    ==
                1.000  SIO2(AQ)

        log k=       0.5748

--------------------------------------------------

                1.000  CRISTOBALITE-ALPHA
                    ==
                1.000  SIO2(AQ)

        log k=       1.2686

--------------------------------------------------

                1.000  CRISTOBALITE-BETA
                    ==
                1.000  SIO2(AQ)

        log k=       1.6541

--------------------------------------------------

                1.000  CHALCEDONY
                    ==
                1.000  SIO2(AQ)

        log k=       0.5518
```

```
--------------------------------------------------

                      1.000  AMORPHOUS_SILICA
                          ==
                      1.000  SIO2(AQ)

         log k=       0.8012

--------------------------------------------------

                      0.100  DECANE(L)
                   +  1.550  O2(G)
                          ==
                      0.100  H2O
                   +  2.000  H+
                   +  1.000  CO3--

         log k=       6.7454

--------------------------------------------------

       --- gas dissolution reactions ---

--------------------------------------------------

                      1.000  CO2(G)
                   +  1.000  H2O
                          ==
                      2.000  H+
                   +  1.000  CO3--

         log k=     -20.5184

--------------------------------------------------

                      1.000  O2(G)
                          ==
                      1.000  O2(G)

         log k=       0.0000

--------------------------------------------------

                      1.000  S2(G)
                   +  2.000  H2O
                   +  3.000  O2(G)
                          ==
                      4.000  H+
                   +  2.000  SO4--

         log k=      -3.7706

--------------------------------------------------

                      1.000  CH4(G)
                   +  2.000  O2(G)
                          ==
                      1.000  H2O
                   +  2.000  H+
                   +  1.000  CO3--

         log k=       0.8193

--------------------------------------------------

                      1.000  H2(G)
                   +  0.500  O2(G)
                          ==
                      1.000  H2O

         log k=       0.9912

--------------------------------------------------

                      1.000  H2S(G)
                   +  2.000  O2(G)
```

```
                   ==
               2.000  H+
            +  1.000  SO4--

        log k=      -2.3578

--------------------------------------------------

               1.000  H2O(G)
                   ==
               1.000  H2O

        log k=      -6.9018

--------------------------------------------------
   zistrt =  0.000000E+00 (initial value of zi)
   zimax  =  2.000000E+01 (maximum value of zi)
   timemx =  1.000000E+38 (maximum value of time, sec)
   kstpmx =          1000 (maximum number of steps this run)


      dzprnt =  1.000000E+38 (linear print interval)
      dzprlg =  5.000000E-01 (logarithmic print interval)
      dlzidp =  1.000000E+38 (p.r.s. transfer interval)

      maximum permitted step sizes.....
         dlzmx1 =  1.000000E-08 (nord=0)
         dlzmx2 =  1.000000E+38 (nord.ge.1)
      nordlm =  6 (maximum permitted order)


   temperature =    900.000 c


   nmodl1 =  2 (physical system switch)
     1 = titration, 2 = closed, 3 = flow-through)
   nmodl2 =  0 (economy mode permission switch)
     0 = normal, 1 = economy, 3 = super economy)

      iopt1  =  0 (kinetic mode switch)
      iopt2  =  0 (suppress phase boundary location)
      iopt3  =  0 (interfacing output switch)
      iopt4  =  1 (permit solid solutions switch)
      iopt5  =  2 (remove initial solids switch)
      iopt6  =  1 (clear p.r.s. at start switch)
      iopt7  =  0 (auto basis switch mode switch)
      iopt8  =  0 (linear vs. log taylor-s series)
      iopt9  =  0 (not used)
      iopt10 =  0 (not used)
      iopt11 =  0 (suppress all redox reactions switch)
      iopt12 =  0 (not used)
      iopt13 =  0 (tab file output switch)
      iopt14 =  0 (ahv input file access)
      iopt15 =  0 (not used)
      iopt16-20   (not used)
      ifile  = 16 (supplementary input file)

         iopg1  =  0 (choice of act. coeff. equations)
         iopg2  = -1 (choice of ph scale)
         iopg3  =  0 (e-lambda switch for hydration theory)
         iopg4  =  0 (not used)
         iopg5  =  1 (use bdot term instead of co2 polynomial)
         iopg6  =  0 (choice of pitzer j(x) approximation)
         iopg7  =  0 (not used)
         iopg8  =  0 (not used)
         iopg9  =  0 (not used)
         iopg10 =  0 (not used)

      iopr1  =  0 (print loading of species from data1)
      iopr2  =  0 (print derivatives of basis elements)
      iopr3  =  1 (print loaded species and log k values)
      iopr4  =  0 (print aqueous species distribution)
      iopr5  =  1 (print cation/h+ activity ratios)
      iopr6  =  0 (print element/oxide comp. of mineral assemblage)
      iopr7  =  0 (print mineral affinity summary)
      iopr8  =  1 (print gas fugacity summary)
```

```
    iopr9  =  0 (print mean molal activity coefficient)
    iopr10 =  0 (print tabulation of pitzer coefficients)
    iopr11 =  0 (print major species for each element)
    iopr12-20   (not used)

       iodb1  =  0 (enable comp. messages)
       iodb2  =  0 (print pre-newton-raphson optimization)
       iodb3  =  0 (print order/scaling info.)
       iodb4  =  0 (print newton iteration info.)
       iodb5  =  0 (print search iterations)
       iodb6  =  0 (print hpsatz iterations)
       iodb7  =  0 (print f.d. and t.s. calculations)
       iodb8  =  0 (turns iodb3 on and off)
       iodb9  =  0 (print kinetics info.)
       iodb10 =  0 (check basis var. f.d. and t.s.)
       iodb11 =  0 (check reac. rate f.d. and t.s.)
       iodb12 =  0 (iteration variable killer option)
       iodb13 =  0 (not used)
       iodb14 =  0 (not used)
       iodb15 =  0 (not used)
       iodb16 =  0 (turn on akmatr prints)
       iodb17-20   (not used)

    tolbt  =  1.000000E-10 (residual function convergence tolerance)
    toldl  =  1.000000E-10 (correction term convergence tolerance)
    tolx   =  1.000000E-08 (sol-sol reactant/product identity tolerance)
    tolsat =  5.000000E-03 (lower supersaturation tolerance)
    tolsst =  1.000000E-02 (upper supersaturation tolerance)


screw1 =  1.000E-04 (primary step-size parameter for basis variables)
screw2 =    0.00000 (not used)
screw3 =  1.000E-04 (step size parameter for rate functions)
screw4 =  1.000E-04 (corrector parameter for rate functions)
screw5 =    4.00000 (under-relaxation control for n-r iteration)
screw6 =    4.00000 (step size parameter for economy mode)

    zklogu =     -6.000 (threshhold log mass for solids)
    zklogl =      2.000 (log mass decrement for p.r.s shift)
     zkfac =      0.980 (shift adjustment factor)
    zklgmn =     -6.009 (minimum log mass after a shift)


        itermx= 30 (newton-raphson iteration limit)
        ntrymx= 25 (phase assemblage try limit)
        npslmx=  8 (critical phase instability slide limit)
        nsslmx=  3 (critical redox instability slide limit)


   iacion = 16, CL-          (defines xisteq)



--- inactive loaded aqueous species ---


  SR++                   ZN++                   PB++
  AG+                    BA++                   NH4+
  F-                     HG++                   MN++
  CU+                    AU+                    CS+
  CD++                   U++++                  HPO4--
  BO(OH)(AQ)             N2(AQ)                 CU++
  NO3-                   CN-                    CASO4(AQ)
  MG(OH)+


--- inactive loaded minerals ---


  BRUCITE                DIASPORE               CALCITE
  MAGNESITE              GROSSULAR              FORSTERITE
  FAYALITE               ENSTATITE-CL           ENSTATITE-OR
  ENSTATITE-PR           DIOPSIDE               HEDENBERGITE
  JADEITE                FERROSILITE            ANNITE
  PHLOGOPITE             PYROPE                 ALMANDINE
  FERROUS_OXIDE          SEPIOLITE              SIDERITE
```

```
                   --- reactants and rate coefficients ---

      forward/dissolution=

   CLINOPYROXENE(SS)        arbitrary relative rate
                    rk1=  1.00000E+00   rk2=  0.00000E+00   rk3=  0.00000E+00
   GARNET(SS)               arbitrary relative rate
                    rk1=  1.00000E+00   rk2=  0.00000E+00   rk3=  0.00000E+00
   COESITE                  arbitrary relative rate
                    rk1=  1.00000E+00   rk2=  0.00000E+00   rk3=  0.00000E+00

   reverse/precipitation=

   CLINOPYROXENE(SS)        no precipitation rate
   GARNET(SS)               no precipitation rate
   COESITE                  no precipitation rate


          --- solid solution reactants ---


          CLINOPYROXENE(SS)
            mol. wt. =      209.617g/mol
            molar volume =       62.098 cc

                           composition

                                          mole fraction

                      DIOPSIDE              2.00000E-01
                      HEDENBERGITE          1.00000E-01
                      JADEITE               7.00000E-01

          GARNET(SS)
            mol. wt. =      145.433g/mol
            molar volume =      114.382 cc

                           composition

                                          mole fraction

                      PYROPE                6.00000E-01
                      ALMANDINE             3.00000E-01
                      GROSSULAR             1.00000E-01

- - - - - - - - - - - - - - - - - - - - - - - - - - - - - - - - - - - -


stepping to zi=  0.0000E+00, delzi=  0.0000E+00, nord= 0

  attempted species assemblage no.   1

             1      1  H2O
             2      2  NA+
             3      3  K+
             4      4  CA++
             5      5  MG++
             6      6  AL+++
             7      7  SIO2(AQ)
             8     13  H+
             9     14  CO3--
            10     16  CL-
            11     17  SO4--
            12     21  FE++
            13     29  O2(G)

               ncycle=  0
iter =   23
      1 supersaturated pure minerals
      1 supersaturated solid solutions

                         the most supersaturated phases     affinity, kcal

                           1   51500   CALCITE(SS)             4.64876715
```

```
                         2      14   DOLOMITE                1.73249514


  attempted species assemblage no.   2

             1      1  H2O
             2      2  NA+
             3      3  K+
             4      4  CA++
             5      5  MG++
             6      6  AL+++
             7      7  SIO2(AQ)
             8     13  H+
             9     14  CO3--
            10     16  CL-
            11     17  SO4--
            12     21  FE++
            13     29  O2(G)
            14      1  CALCITE(CALCITE
            15      2  CALCITE(MAGNESITE
            16      3  CALCITE(SIDERITE

iter =   24
      1 supersaturated pure minerals
      0 supersaturated solid solutions

                       the most supersaturated phases     affinity, kcal

                         1       2   DIAMOND                 0.09942597


  attempted species assemblage no.   3

             1      1  H2O
             2      2  NA+
             3      3  K+
             4      4  CA++
             5      5  MG++
             6      6  AL+++
             7      7  SIO2(AQ)
             8     13  H+
             9     14  CO3--
            10     16  CL-
            11     17  SO4--
            12     21  FE++
            13     29  O2(G)
            14      2  DIAMOND
            15      1  CALCITE(CALCITE
            16      2  CALCITE(MAGNESITE
            17      3  CALCITE(SIDERITE

iter =   20

- - - - - - - - - - - - - - - - - - - - - - - - - - - - - - - -



                    reaction progress        =  0.000000000000000E+00
                    log of reaction progress =    -999.0000000

                    temperature    =    900.000 degrees c
                    total pressure =  50000.000 bars

                    computing units remaining =      0.000


change in the product phase assemblage

start or re-start of run


                              --- reactant summary ---


    definitions and conventions
```

delta x = x now - x at start
affinity is + for forward direction (dissolution),
- for reverse direction (precipitation)
rates are + for forward direction (dissolution),
- for reverse direction (precipitation)

reactant	moles	delta moles	grams	delta grams
CLINOPYROXENE(SS)	2.00000E+01	0.00000E+00	4.19234E+03	0.00000E+00
GARNET(SS)	2.00000E+01	0.00000E+00	2.90867E+03	0.00000E+00
COESITE	2.00000E+01	0.00000E+00	1.20169E+03	0.00000E+00

current total mass = 8.30269E+03 grams
delta total mass = 0.00000E+00 grams
delta total volume = 0.00000 cc

reactant	affinity	rel. rate
CLINOPYROXENE(SS)	20.9045	1.00000E+00
GARNET(SS)	15.3537	1.00000E+00
COESITE	3.5190	1.00000E+00

affinity of the overall irreversible reaction= 39.777 kcal
contributions from irreversible reactions
with no thermodynamic data are not included

--- element totals for the aqueous phase ---

element	mg/kg soln.	molal conc.	moles
O	7.273301E+05	1.286050E+02	1.294610E+02
NA	2.421836E+03	2.980164E-01	3.000000E-01
K	2.333972E+04	1.688759E+00	1.700000E+00
CA	4.542477E+04	3.206233E+00	3.227574E+00
MG	4.630786E+02	5.390005E-02	5.425881E-02
AL	8.983888E-01	9.419495E-05	9.482192E-05
SI	8.875923E+03	8.940491E-01	9.000000E-01
H	4.331046E+04	1.215641E+02	1.223733E+02
C	1.451787E+05	3.419429E+01	3.442190E+01
CL	1.244921E+03	9.933879E-02	1.000000E-01
S	2.089768E+03	1.844016E-01	1.856290E-01
FE	3.199028E+02	1.620497E-02	1.631283E-02
co3--		0.000000E+00	0.000000E+00
so4--		0.000000E+00	0.000000E+00
s--		0.000000E+00	0.000000E+00

warning-- co3--, so4--, and s-- totals require that routine comp1
have the names of non-carbonate carbon, sulfide sulfur,
and non-sulfate sulfur aqueous species

single ion activities and activity coefficients are here defined
with respect to the internal ph scale

	ph	eh	pe
internal ph scale	4.7393	1.3458	5.7816E+00
modified nbs ph scale	4.4186	1.4204	6.1023E+00
rational ph scale	4.4186	1.4204	6.1023E+00

phcl = 6.1381

oxygen fugacity = 2.48730E-10
log oxygen fugacity = -9.60427

```
               activity of water =   0.99667
log activity of water =  -0.00145
alkalinity =  0.000000E+00 equiv/kg solvent
                           (not def. for t.gt.50 c)

     ionic strength =  5.212520E+00 molal
  sum of molalities =   36.8425151002908
osmotic coefficient =    0.00502
     equiv. stoich. ionic strength =  9.933879E-02 molal

          mass of solution =   2.847811 kg
          mass of solvent  =   1.006656 kg
          mass of solutes  =   1.841155 kg
          conc of solutes  =  64.651584 per cent (w/w)
```

species	moles	grams	conc	log conc	log g	log act
H2O	5.58782E+01	1.00666E+03				
NA+	2.60353E-01	5.98545E+00	2.58631E-01	-0.58732	-0.32065	-0.90797
K+	1.47005E+00	5.74765E+01	1.46033E+00	0.16445	-0.32065	-0.15620
CA++	1.55306E-04	6.22467E-03	1.54279E-04	-3.81169	-1.28261	-5.09430
MG++	1.21183E-05	2.94536E-04	1.20382E-05	-4.91944	-1.28261	-6.20205
AL+++	4.52241E-19	1.22022E-17	4.49251E-19	-18.34751	-2.88588	-21.23339
SIO2(AQ)	4.00361E-01	2.40554E+01	3.97713E-01	-0.40043	0.00000	-0.40043
H+	3.83914E-05	3.86947E-05	3.81375E-05	-4.41865	-0.32065	-4.73930
CO3--	2.23756E-01	1.34274E+01	2.22276E-01	-0.65311	-1.28261	-1.93572
CL-	8.40901E-02	2.98125E+00	8.35341E-02	-1.07814	-0.32065	-1.39879
SO4--	2.94742E-04	2.83122E-02	2.92793E-04	-3.53344	-1.28261	-4.81605
FE++	9.24498E-08	5.16304E-06	9.18385E-08	-7.03698	-1.28261	-8.31959
O2(AQ)	1.87676E-16	6.00542E-15	1.86435E-16	-15.72947	0.00000	-15.72947
H2(AQ)	1.31316E-02	2.64708E-02	1.30448E-02	-1.88456	0.00000	-1.88456
CH4(AQ)	5.08577E-02	8.15890E-01	5.05214E-02	-1.29652	0.00000	-1.29652
HS-	4.00429E-02	1.32414E+00	3.97782E-02	-1.40036	-0.32065	-1.72101
FE+++	1.52099E-14	8.49427E-13	1.51093E-14	-13.82075	-2.88588	-16.70663
HCO3-	5.04562E-01	3.07869E+01	5.01226E-01	-0.29997	-0.32065	-0.62062
CLO4-	1.64173E-42	1.63271E-40	1.63087E-42	-41.78758	-0.32065	-42.10823
OH-	5.38005E-01	9.15002E+00	5.34448E-01	-0.27209	-0.32065	-0.59275
HCOO-	2.54654E+00	1.14639E+02	2.52970E+00	0.40307	-0.32065	0.08242
CH3COO-	2.97125E-02	1.75436E+00	2.95160E-02	-1.52994	-0.32065	-1.85060
CH3CH2COO-	4.97538E-01	3.63558E+01	4.94248E-01	-0.30605	-0.32065	-0.62671
CO(AQ)	6.42903E-02	1.80080E+00	6.38652E-02	-1.19474	0.00000	-1.19474
ETHANE(AQ)	2.16344E-04	6.50533E-03	2.14913E-04	-3.66774	0.00000	-3.66774
ETHYLENE(AQ)	4.69415E-07	1.31688E-05	4.66311E-07	-6.33132	0.00000	-6.33132
PROPANE(AQ)	1.23547E-06	5.44796E-05	1.22730E-06	-5.91105	0.00000	-5.91105
HEXANE(AQ)	2.99017E-13	2.57683E-11	2.97040E-13	-12.52719	0.00000	-12.52719
BENZENE(AQ)	9.96425E-13	7.78341E-11	9.89836E-13	-12.00444	0.00000	-12.00444
TOLUENE(AQ)	4.01636E-15	3.70068E-13	3.98980E-15	-14.39905	0.00000	-14.39905
SI2O4(AQ)	2.15487E-02	2.58948E+00	2.14062E-02	-1.66946	0.00000	-1.66946
ALO2-	6.92476E-06	4.08425E-04	6.87897E-06	-5.16248	-0.32065	-5.48313
ALO2(SIO2)-	8.78972E-05	1.04654E-02	8.73160E-05	-4.05891	-0.32065	-4.37956
CACL+	2.32037E-04	1.75265E-02	2.30503E-04	-3.63732	-0.32065	-3.95798
CACL2(AQ)	3.94251E-07	4.37563E-05	3.91644E-07	-6.40711	0.00000	-6.40711
CACO3(AQ)	5.71481E-03	5.71990E-01	5.67702E-03	-2.24588	0.00000	-2.24588
CA(HCO3)+	3.16653E+00	3.20127E+02	3.14559E+00	0.49770	-0.24503	0.25267
CA(OH)+	4.15710E-04	2.37318E-02	4.12962E-04	-3.38409	-0.32065	-3.70474
CA(HSIO3)+	5.45299E-02	6.38936E+00	5.41694E-02	-1.26625	-0.32065	-1.58690
FECL+	5.36297E-08	4.89640E-06	5.32751E-08	-7.27348	-0.32065	-7.59413
FECL2(AQ)	4.40015E-05	5.57733E-03	4.37106E-05	-4.35941	0.00000	-4.35941
FE(HSIO3)+	1.62687E-02	2.16274E+00	1.61611E-02	-1.79153	-0.32065	-2.11218
KCL(AQ)	1.44968E-02	1.08076E+00	1.44010E-02	-1.84161	0.00000	-1.84161
KOH	2.15439E-01	1.20873E+01	2.14014E-01	-0.66956	0.00000	-0.66956
KSO4-	1.26999E-05	1.71646E-03	1.26159E-05	-4.89908	-0.32065	-5.21973
MGCL+	5.12470E-06	3.06242E-04	5.09082E-06	-5.29321	-0.32065	-5.61387
MG(HCO3)+	6.75534E-04	5.76380E-02	6.71068E-04	-3.17323	-0.32065	-3.49389
MG(HSIO3)+	5.35660E-02	5.43141E+00	5.32118E-02	-1.27399	-0.32065	-1.59464
MGSO4(AQ)	9.63119E-09	1.15924E-06	9.56751E-09	-8.01920	0.00000	-8.01920
NACL(AQ)	8.17152E-04	4.77566E-02	8.11749E-04	-3.09058	0.00000	-3.09058
NACO3-	5.43077E-04	4.50748E-02	5.39486E-04	-3.26802	-0.32065	-3.58867
NAHCO3(AQ)	7.04311E-03	5.91669E-01	6.99654E-03	-2.15512	0.00000	-2.15512
NAHSIO3(AQ)	2.29464E-04	2.29651E-02	2.27947E-04	-3.64217	0.00000	-3.64217
NAOH(AQ)	3.10143E-02	1.24048E+00	3.08092E-02	-1.51132	0.00000	-1.51132
HSIO3-	3.31860E-01	2.55836E+01	3.29666E-01	-0.48193	-0.32065	-0.80258
HSO4-	1.89242E-03	1.83689E-01	1.87991E-03	-2.72586	-0.32065	-3.04652
H2S(AQ)	1.43386E-01	4.88600E+00	1.42438E-01	-0.84637	0.00000	-0.84637

CO2(AQ)	2.62922E+01	1.15711E+03	2.61183E+01	1.41695	0.00000	1.41695
HCL(AQ)	2.69904E-04	9.84093E-03	2.68119E-04	-3.57167	0.00000	-3.57167
CH3CH2COOH	5.20604E-05	3.85659E-03	5.17162E-05	-4.28637	0.00000	-4.28637
CH3COOH	1.69982E-03	1.02078E-01	1.68858E-03	-2.77248	0.00000	-2.77248
HCOOH	3.15827E-03	1.45361E-01	3.13739E-03	-2.50343	0.00000	-2.50343

--- activity ratios of cations ---

log (NA+ /h+**0)	3.8313289
log (K+ /h+**0)	4.5830991
log (CA++ /h+**0)	4.3842967
log (MG++ /h+**0)	3.2765510
log (AL+++ /h+**0)	-7.0154861
log (FE++ /h+**0)	1.1590140
log (FE+++ /h+**0)	-2.4887299

--- summary of solid product phases---

product	log moles	moles	grams	volume, cc
DIAMOND	-0.6315913	2.33566E-01	2.80536E+00	7.98794E-01
CALCITE(SS)	-0.4627533	3.44546E-01	3.12327E+01	1.02363E+01
CALCITE	-1.2274764	5.92275E-02	5.92804E+00	2.18550E+00
MAGNESITE	-0.6095220	2.45741E-01	2.07195E+01	6.88813E+00
SIDERITE	-1.4025590	3.95768E-02	4.58522E+00	1.16269E+00

--- grand summary of solid phases (e.s.+p.r.s.+reactants) ---

phase/end-member	log moles	moles	grams	volume, cc
DIAMOND	-0.6315913	2.33566E-01	2.80536E+00	7.98794E-01
COESITE	1.3010300	2.00000E+01	1.20169E+03	4.53760E+02
CLINOPYROXENE(SS)	1.3010300	2.00000E+01		
DIOPSIDE	0.6020600	4.00000E+00	8.66210E+02	2.64800E+02
HEDENBERGITE	0.3010300	2.00000E+00	4.96189E+02	1.32400E+02
JADEITE	1.1461280	1.40000E+01	2.82994E+03	8.44760E+02
GARNET(SS)	1.3010300	2.00000E+01		
PYROPE	1.0791812	1.20000E+01	1.61259E+03	1.35792E+03
ALMANDINE	0.7781513	6.00000E+00	9.95734E+02	6.78960E+02
GROSSULAR	0.3010300	2.00000E+00	3.00346E+02	2.50760E+02
CALCITE(SS)	-0.4627533	3.44546E-01		
CALCITE	-1.2274764	5.92275E-02	5.92804E+00	2.18550E+00
MAGNESITE	-0.6095220	2.45741E-01	2.07195E+01	6.88813E+00
SIDERITE	-1.4025590	3.95768E-02	4.58522E+00	1.16269E+00

	mass, grams	volume, cc
created	3.403809E+01	1.103510E+01
destroyed	0.000000E+00	0.000000E+00
net	3.403809E+01	1.103510E+01

warning-- these volume totals may be incomplete because
of missing partial molar volume data in the data base

--- mineral saturation state summary ---

mineral	affinity, kcal	state	mineral	affinity, kcal	state

```
DIAMOND                 0.0000  satd         BRUCITE                 -7.2209
CALCITE                -4.1051               ARAGONITE               -2.3809
MAGNESITE              -0.7879               DOLOMITE                -3.6567
FORSTERITE             -9.8292               ENSTATITE-CL            -4.6060
ENSTATITE-OR           -4.5110               ENSTATITE-PR            -5.7108
DIOPSIDE               -8.5220               FERROSILITE             -9.5954
COESITE                -3.5190               GRAPHITE                -0.3430
PYRRHOTITE             -3.9971               PYRITE                  -4.8369
FERROUS_OXIDE          -6.7195               SEPIOLITE             1034.8824  ssatd
SIDERITE               -5.0450               QUARTZ-ALPHA            -4.1572
QUARTZ-BETA            -5.2352               CRISTOBALITE-ALPHA      -8.9596
CHALCEDONY             -5.1117               AMORPHOUS_SILICA        -6.4505


                    --- summary of solid solutions  ---

mineral               aff. kcal/mol  mole frac.    lambda      state

ORTHOPYROXENE(SS)        -4.2615
    FERROSILITE                -4.26152    0.1014822     1.00000
    ENSTATITE-OR               -4.26152    0.8985178     1.00000

OLIVINE                  -9.7264
    FAYALITE                   -9.72639    0.0431395     1.00000
    FORSTERITE                 -9.72639    0.9568605     1.00000

BIOTITE                 -29.4596
    PHLOGOPITE                -29.45965    0.9992091     1.00000
    ANNITE                    -29.45965    0.0007909     1.00000

CLINOPYROXENE(SS)        -8.3055
    DIOPSIDE                   -8.30552    0.9113361     1.00000
    HEDENBERGITE               -8.30552    0.0884589     1.00000
    JADEITE                    -8.30552    0.0002050     1.00000

GARNET(SS)              -15.1897
    PYROPE                    -15.18966    0.5831516     1.00000
    ALMANDINE                 -15.18966    0.1916139     1.00000
    GROSSULAR                 -15.18966    0.2252345     1.00000

CALCITE(SS)               0.0000                            saturated
    CALCITE                     0.00000    0.1719004     1.00000
    MAGNESITE                   0.00000    0.7132328     1.00000
    SIDERITE                    0.00000    0.1148668     1.00000


solid solution product phases

           xbar     lambda   activity  log xbar  log lambda log activity

  CALCITE(SS)
ideal solution

    CALCITE
            0.1719    1.0000    0.1719   -0.7647    0.0000   -0.7647
    MAGNESITE
            0.7132    1.0000    0.7132   -0.1468    0.0000   -0.1468
    SIDERITE
            0.1149    1.0000    0.1149   -0.9398    0.0000   -0.9398


          --- summary of gas species ---

          gas               log fugacity         fugacity         partial pressure

  CO2(G)                        9.10553      1.27505E+09
  O2(G)                        -9.60427      2.48730E-10
  S2(G)                         3.99701      9.93132E+03
  CH4(G)                        6.97348      9.40753E+06
  H2(G)                         3.80949      6.44893E+03
  H2S(G)                        7.27169      1.86935E+07
  H2O(G)                        6.90035      7.94972E+06



- - - - - - - - - - - - - - - - - - - - - - - - - - - - - - - -
```

```
--- shifting e.s. solid(s) to the p.r.s. (shftz) ---

      shifted -- DIAMOND
              - old mass=  2.33566E-01, new mass=  0.00000E+00
      shifted -- CALCITE(SS)
              - old mass=  3.44546E-01, new mass=  0.00000E+00
* note-- clearing the p.r.s. (eq6)

stepping to zi=  1.0000E-08, delzi=  1.0000E-08, nord= 0
              ncycle=  0
steps completed =     1, iter =  5, ncorr = 0
most rapidly changing is zvclg1(AL+++            ) =    -18.3446

stepping to zi=  1.0000E-08, delzi=  6.0775E-17, nord= 0
steps completed =     2, iter =  1, ncorr = 0
most rapidly changing is zvclg1(AL+++            ) =    -18.3446
- - - - - - - - - - - - - - - - - - - - - - - - - - - - - - - -



                    reaction progress        =  9.99999999999998E-09
                    log of reaction progress =      -8.0000000

                    temperature    =    900.000 degrees c
                    total pressure =  50000.000 bars

                    computing units remaining =      0.000


step size is limited by the print requirement


                              --- reactant summary ---



        reactant            moles       delta moles      grams        delta grams

  CLINOPYROXENE(SS)       2.00000E+01    1.00000E-08    4.19234E+03    2.09617E-06
  GARNET(SS)              2.00000E+01    1.00000E-08    2.90867E+03    1.45433E-06
  COESITE                 2.00000E+01    1.00000E-08    1.20169E+03    6.00843E-07


               current total mass =  8.30269E+03 grams
               delta total mass   =  4.15135E-06 grams
               delta total volume =      0.00000 cc


        reactant           affinity     rel. rate

  CLINOPYROXENE(SS)         20.9043    1.00000E+00
  GARNET(SS)                15.3535    1.00000E+00
  COESITE                    3.5190    1.00000E+00

         affinity of the overall irreversible reaction=    39.777 kcal
         contributions from irreversible reactions
         with no thermodynamic data are not included



     --- element totals for the aqueous phase ---

  element         mg/kg soln.         molal conc.          moles

     O         7.273301E+05       1.286050E+02       1.294610E+02
     NA        2.421836E+03       2.980164E-01       3.000000E-01
     K         2.333972E+04       1.688759E+00       1.700000E+00
     CA        4.542477E+04       3.206233E+00       3.227574E+00
     MG        4.630787E+02       5.390006E-02       5.425882E-02
     AL        8.985182E-01       9.420852E-05       9.483558E-05
     SI        8.875923E+03       8.940492E-01       9.000000E-01
     H         4.331046E+04       1.215641E+02       1.223733E+02
     C         1.451787E+05       3.419429E+01       3.442190E+01
     CL        1.244921E+03       9.933879E-02       1.000000E-01
```

S	2.089768E+03	1.844016E-01	1.856290E-01
FE	3.199029E+02	1.620497E-02	1.631284E-02
co3--		0.000000E+00	0.000000E+00
so4--		0.000000E+00	0.000000E+00
s--		0.000000E+00	0.000000E+00

warning-- co3--, so4--, and s-- totals require that routine comp1
have the names of non-carbonate carbon, sulfide sulfur,
and non-sulfate sulfur aqueous species

single ion activities and activity coefficients are here defined
with respect to the internal ph scale

	ph	eh	pe
internal ph scale	4.7393	1.3458	5.7816E+00
modified nbs ph scale	4.4186	1.4204	6.1023E+00
rational ph scale	4.4186	1.4204	6.1023E+00

phcl = 6.1381

oxygen fugacity = 2.48730E-10
log oxygen fugacity = -9.60427

activity of water = 0.99667
log activity of water = -0.00145
alkalinity = 0.000000E+00 equiv/kg solvent
(not def. for t.gt.50 c)

ionic strength = 5.212520E+00 molal
sum of molalities = 36.8425151550886
osmotic coefficient = 0.00502
equiv. stoich. ionic strength = 9.933879E-02 molal

mass of solution = 2.847811 kg
mass of solvent = 1.006656 kg
mass of solutes = 1.841155 kg
conc of solutes = 64.651584 per cent (w/w)

species	moles	grams	conc	log conc	log g	log act
H2O	5.58782E+01	1.00666E+03				
NA+	2.60353E-01	5.98545E+00	2.58631E-01	-0.58732	-0.32065	-0.90797
K+	1.47005E+00	5.74765E+01	1.46033E+00	0.16445	-0.32065	-0.15620
CA++	1.55306E-04	6.22467E-03	1.54279E-04	-3.81169	-1.28261	-5.09430
MG++	1.21183E-05	2.94536E-04	1.20382E-05	-4.91944	-1.28261	-6.20205
AL+++	4.52306E-19	1.22039E-17	4.49315E-19	-18.34745	-2.88588	-21.23333
SIO2(AQ)	4.00361E-01	2.40554E+01	3.97713E-01	-0.40043	0.00000	-0.40043
H+	3.83914E-05	3.86947E-05	3.81375E-05	-4.41865	-0.32065	-4.73930
CO3--	2.23756E-01	1.34274E+01	2.22276E-01	-0.65311	-1.28261	-1.93572
CL-	8.40901E-02	2.98125E+00	8.35341E-02	-1.07814	-0.32065	-1.39879
SO4--	2.94742E-04	2.83122E-02	2.92793E-04	-3.53344	-1.28261	-4.81605
FE++	9.24498E-08	5.16304E-06	9.18385E-08	-7.03698	-1.28261	-8.31959
O2(AQ)	1.87676E-16	6.00542E-15	1.86435E-16	-15.72947	0.00000	-15.72947
H2(AQ)	1.31316E-02	2.64708E-02	1.30448E-02	-1.88456	0.00000	-1.88456
CH4(AQ)	5.08577E-02	8.15890E-01	5.05214E-02	-1.29652	0.00000	-1.29652
HS-	4.00429E-02	1.32414E+00	3.97782E-02	-1.40036	-0.32065	-1.72101
FE+++	1.52099E-14	8.49427E-13	1.51093E-14	-13.82075	-2.88588	-16.70663
HCO3-	5.04562E-01	3.07869E+01	5.01226E-01	-0.29997	-0.32065	-0.62062
CLO4-	1.64173E-42	1.63271E-40	1.63087E-42	-41.78758	-0.32065	-42.10823
OH-	5.38005E-01	9.15002E+00	5.34448E-01	-0.27209	-0.32065	-0.59275
HCOO-	2.54654E+00	1.14639E+02	2.52970E+00	0.40307	-0.32065	0.08242
CH3COO-	2.97125E-02	1.75436E+00	2.95160E-02	-1.52994	-0.32065	-1.85060
CH3CH2COO-	4.97538E-01	3.63558E+01	4.94248E-01	-0.30605	-0.32065	-0.62671
CO(AQ)	6.42903E-02	1.80080E+00	6.38652E-02	-1.19474	0.00000	-1.19474
ETHANE(AQ)	2.16344E-04	6.50533E-03	2.14913E-04	-3.66774	0.00000	-3.66774
ETHYLENE(AQ)	4.69415E-07	1.31688E-05	4.66311E-07	-6.33132	0.00000	-6.33132

PROPANE(AQ)	1.23547E-06	5.44796E-05	1.22730E-06	-5.91105	0.00000	-5.91105
HEXANE(AQ)	2.99017E-13	2.57683E-11	2.97040E-13	-12.52719	0.00000	-12.52719
BENZENE(AQ)	9.96425E-13	7.78341E-11	9.89836E-13	-12.00444	0.00000	-12.00444
TOLUENE(AQ)	4.01636E-15	3.70068E-13	3.98980E-15	-14.39905	0.00000	-14.39905
SI2O4(AQ)	2.15487E-02	2.58948E+00	2.14062E-02	-1.66946	0.00000	-1.66946
ALO2-	6.92576E-06	4.08483E-04	6.87996E-06	-5.16241	-0.32065	-5.48307
ALO2(SIO2)-	8.79098E-05	1.04670E-02	8.73286E-05	-4.05884	-0.32065	-4.37950
CACL+	2.32037E-04	1.75265E-02	2.30503E-04	-3.63732	-0.32065	-3.95798
CACL2(AQ)	3.94251E-07	4.37563E-05	3.91644E-07	-6.40711	0.00000	-6.40711
CACO3(AQ)	5.71481E-03	5.71990E-01	5.67702E-03	-2.24588	0.00000	-2.24588
CA(HCO3)+	3.16653E+00	3.20127E+02	3.14559E+00	0.49770	-0.24503	0.25267
CA(OH)+	4.15710E-04	2.37318E-02	4.12962E-04	-3.38409	-0.32065	-3.70474
CA(HSIO3)+	5.45300E-02	6.38936E+00	5.41694E-02	-1.26625	-0.32065	-1.58690
FECL+	5.36298E-08	4.89640E-06	5.32752E-08	-7.27348	-0.32065	-7.59413
FECL2(AQ)	4.40015E-05	5.57733E-03	4.37106E-05	-4.35941	0.00000	-4.35941
FE(HSIO3)+	1.62687E-02	2.16274E+00	1.61611E-02	-1.79153	-0.32065	-2.11218
KCL(AQ)	1.44968E-02	1.08076E+00	1.44010E-02	-1.84161	0.00000	-1.84161
KOH	2.15439E-01	1.20873E+01	2.14014E-01	-0.66956	0.00000	-0.66956
KSO4-	1.26999E-05	1.71646E-03	1.26159E-05	-4.89908	-0.32065	-5.21973
MGCL+	5.12470E-06	3.06242E-04	5.09082E-06	-5.29321	-0.32065	-5.61387
MG(HCO3)+	6.75535E-04	5.76380E-02	6.71068E-04	-3.17323	-0.32065	-3.49389
MG(HSIO3)+	5.35660E-02	5.43141E+00	5.32118E-02	-1.27399	-0.32065	-1.59464
MGSO4(AQ)	9.63120E-09	1.15924E-06	9.56751E-09	-8.01920	0.00000	-8.01920
NACL(AQ)	8.17152E-04	4.77566E-02	8.11749E-04	-3.09058	0.00000	-3.09058
NACO3-	5.43077E-04	4.50748E-02	5.39486E-04	-3.26802	-0.32065	-3.58867
NAHCO3(AQ)	7.04311E-03	5.91669E-01	6.99654E-03	-2.15512	0.00000	-2.15512
NAHSIO3(AQ)	2.29464E-04	2.29651E-02	2.27947E-04	-3.64217	0.00000	-3.64217
NAOH(AQ)	3.10143E-02	1.24048E+00	3.08092E-02	-1.51132	0.00000	-1.51132
HSIO3-	3.31860E-01	2.55836E+01	3.29666E-01	-0.48193	-0.32065	-0.80258
HSO4-	1.89242E-03	1.83689E-01	1.87991E-03	-2.72586	-0.32065	-3.04652
H2S(AQ)	1.43386E-01	4.88600E+00	1.42438E-01	-0.84637	0.00000	-0.84637
CO2(AQ)	2.62922E+01	1.15711E+03	2.61183E+01	1.41695	0.00000	1.41695
HCL(AQ)	2.69904E-04	9.84093E-03	2.68119E-04	-3.57167	0.00000	-3.57167
CH3CH2COOH	5.20604E-05	3.85659E-03	5.17162E-05	-4.28637	0.00000	-4.28637
CH3COOH	1.69982E-03	1.02078E-01	1.68858E-03	-2.77248	0.00000	-2.77248
HCOOH	3.15827E-03	1.45361E-01	3.13739E-03	-2.50343	0.00000	-2.50343

--- activity ratios of cations ---

log (NA+ /h+**0)	3.8313289
log (K+ /h+**0)	4.5830991
log (CA++ /h+**0)	4.3842967
log (MG++ /h+**0)	3.2765511
log (AL+++ /h+**0)	-7.0154235
log (FE++ /h+**0)	1.1590141
log (FE+++ /h+**0)	-2.4887298

--- grand summary of solid phases (e.s.+p.r.s.+reactants) ---

phase/end-member	log moles	moles	grams	volume, cc
COESITE	1.3010300	2.00000E+01	1.20169E+03	4.53760E+02
CLINOPYROXENE(SS)	1.3010300	2.00000E+01		
DIOPSIDE	0.6020600	4.00000E+00	8.66210E+02	2.64800E+02
HEDENBERGITE	0.3010300	2.00000E+00	4.96189E+02	1.32400E+02
JADEITE	1.1461280	1.40000E+01	2.82994E+03	8.44760E+02
GARNET(SS)	1.3010300	2.00000E+01		
PYROPE	1.0791812	1.20000E+01	1.61259E+03	1.35792E+03
ALMANDINE	0.7781513	6.00000E+00	9.95734E+02	6.78960E+02
GROSSULAR	0.3010300	2.00000E+00	3.00346E+02	2.50760E+02

	mass, grams	volume, cc
created	0.000000E+00	0.000000E+00
destroyed	4.151346E-06	1.991680E-06
net	-4.151346E-06	-1.991680E-06

warning-- these volume totals may be incomplete because
of missing partial molar volume data in the data base

--- mineral saturation state summary ---

mineral	affinity, kcal	state	mineral	affinity, kcal	state
DIAMOND	0.0000		BRUCITE	-7.2209	
CALCITE	-4.1051		ARAGONITE	-2.3809	
MAGNESITE	-0.7879		DOLOMITE	-3.6567	
FORSTERITE	-9.8292		ENSTATITE-CL	-4.6060	
ENSTATITE-OR	-4.5110		ENSTATITE-PR	-5.7108	
DIOPSIDE	-8.5220		FERROSILITE	-9.5954	
COESITE	-3.5190		GRAPHITE	-0.3430	
PYRRHOTITE	-3.9971		PYRITE	-4.8369	
FERROUS_OXIDE	-6.7195		SEPIOLITE	1034.8824	ssatd
SIDERITE	-5.0450		QUARTZ-ALPHA	-4.1572	
QUARTZ-BETA	-5.2352		CRISTOBALITE-ALPHA	-8.9596	
CHALCEDONY	-5.1117		AMORPHOUS_SILICA	-6.4505	

--- summary of solid solutions ---

mineral	aff. kcal/mol		mole frac.	lambda	state
ORTHOPYROXENE(SS)	-4.2615				
FERROSILITE		-4.26152	0.1014822	1.00000	
ENSTATITE-OR		-4.26152	0.8985178	1.00000	
OLIVINE	-9.7264				
FAYALITE		-9.72639	0.0431395	1.00000	
FORSTERITE		-9.72639	0.9568605	1.00000	
BIOTITE	-29.4593				
PHLOGOPITE		-29.45931	0.9992091	1.00000	
ANNITE		-29.45931	0.0007909	1.00000	
CLINOPYROXENE(SS)	-8.3055				
DIOPSIDE		-8.30551	0.9113361	1.00000	
HEDENBERGITE		-8.30551	0.0884589	1.00000	
JADEITE		-8.30551	0.0002050	1.00000	
GARNET(SS)	-15.1894				
PYROPE		-15.18943	0.5831516	1.00000	
ALMANDINE		-15.18943	0.1916139	1.00000	
GROSSULAR		-15.18943	0.2252345	1.00000	
CALCITE(SS)	0.0000				
CALCITE		0.00000	0.1719004	1.00000	
MAGNESITE		0.00000	0.7132328	1.00000	
SIDERITE		0.00000	0.1148668	1.00000	

solid solution product phases

	xbar	lambda	activity	log xbar	log lambda	log activity

--- summary of gas species ---

gas	log fugacity	fugacity	partial pressure
CO2(G)	9.10553	1.27505E+09	
O2(G)	-9.60427	2.48730E-10	
S2(G)	3.99701	9.93132E+03	
CH4(G)	6.97348	9.40753E+06	
H2(G)	3.80949	6.44893E+03	
H2S(G)	7.27169	1.86935E+07	
H2O(G)	6.90035	7.94972E+06	

- -

```
stepping to zi=  2.0000E-08, delzi=  1.0000E-08, nord= 0
              ncycle=  0
steps completed =     3, iter =  5, ncorr = 0
most rapidly changing is zvclg1(AL+++            ) =    -18.3445

stepping to zi=  3.0000E-08, delzi=  1.0000E-08, nord= 0
              ncycle=  0
steps completed =     4, iter =  5, ncorr = 0
most rapidly changing is zvclg1(AL+++            ) =    -18.3444

stepping to zi=  3.1623E-08, delzi=  1.6228E-09, nord= 2
              ncycle=  0
steps completed =     5, iter =  1, ncorr = 0
most rapidly changing is zvclg1(AL+++            ) =    -18.3444
- - - - - - - - - - - - - - - - - - - - - - - - - - - - - - - -



                    reaction progress        =  3.16227766016837E-08
                    log of reaction progress =      -7.5000000

                    temperature    =    900.000 degrees c
                    total pressure =  50000.000 bars

                    computing units remaining =      0.000


step size is limited by the print requirement


                              --- reactant summary ---



        reactant            moles       delta moles      grams        delta grams

  CLINOPYROXENE(SS)       2.00000E+01    3.16228E-08    4.19234E+03    6.62867E-06
  GARNET(SS)              2.00000E+01    3.16228E-08    2.90867E+03    4.59900E-06
  COESITE                 2.00000E+01    3.16228E-08    1.20169E+03    1.90003E-06


               current total mass =  8.30269E+03 grams
               delta total mass   =  1.31277E-05 grams
               delta total volume =      0.00001 cc


        reactant           affinity     rel. rate

  CLINOPYROXENE(SS)         20.9038    1.00000E+00
  GARNET(SS)                15.3530    1.00000E+00
  COESITE                    3.5190    1.00000E+00

         affinity of the overall irreversible reaction=    39.776 kcal
         contributions from irreversible reactions
         with no thermodynamic data are not included



     --- element totals for the aqueous phase ---

  element         mg/kg soln.         molal conc.          moles

     O         7.273301E+05       1.286050E+02       1.294610E+02
     NA        2.421836E+03       2.980164E-01       3.000000E-01
     K         2.333972E+04       1.688759E+00       1.700000E+00
     CA        4.542477E+04       3.206233E+00       3.227574E+00
     MG        4.630788E+02       5.390007E-02       5.425884E-02
     AL        8.987981E-01       9.423786E-05       9.486512E-05
     SI        8.875924E+03       8.940493E-01       9.000001E-01
     H         4.331046E+04       1.215641E+02       1.223733E+02
     C         1.451787E+05       3.419429E+01       3.442190E+01
     CL        1.244921E+03       9.933879E-02       1.000000E-01
     S         2.089768E+03       1.844016E-01       1.856290E-01
```

```
   FE        3.199031E+02       1.620498E-02       1.631285E-02

   co3--                        0.000000E+00       0.000000E+00
   so4--                        0.000000E+00       0.000000E+00
   s--                          0.000000E+00       0.000000E+00

 warning-- co3--, so4--, and s-- totals require that routine comp1
 have the names of non-carbonate carbon, sulfide sulfur,
 and non-sulfate sulfur aqueous species


single ion activities and activity coefficients are here defined
with respect to the internal ph scale



                                   ph           eh           pe

  internal ph scale              4.7393       1.3458    5.7816E+00
  modified nbs ph scale          4.4186       1.4204    6.1023E+00
  rational ph scale              4.4186       1.4204    6.1023E+00

phcl =      6.1381



       oxygen fugacity =  2.48730E-10
       log oxygen fugacity =  -9.60427



                          activity of water =   0.99667
            log activity of water =  -0.00145
            alkalinity =  0.000000E+00 equiv/kg solvent
                                    (not def. for t.gt.50 c)

                 ionic strength =  5.212520E+00 molal
              sum of molalities =   36.8425152729370
            osmotic coefficient =    0.00502
                 equiv. stoich. ionic strength =  9.933879E-02 molal

                      mass of solution =   2.847811 kg
                      mass of solvent  =   1.006656 kg
                      mass of solutes  =   1.841155 kg
                      conc of solutes  =  64.651584 per cent (w/w)
```

species	moles	grams	conc	log conc	log g	log act
H2O	5.58782E+01	1.00666E+03				
NA+	2.60353E-01	5.98545E+00	2.58631E-01	-0.58732	-0.32065	-0.90797
K+	1.47005E+00	5.74765E+01	1.46033E+00	0.16445	-0.32065	-0.15620
CA++	1.55306E-04	6.22467E-03	1.54279E-04	-3.81169	-1.28261	-5.09430
MG++	1.21183E-05	2.94536E-04	1.20382E-05	-4.91944	-1.28261	-6.20205
AL+++	4.52447E-19	1.22077E-17	4.49455E-19	-18.34731	-2.88588	-21.23319
SIO2(AQ)	4.00361E-01	2.40554E+01	3.97713E-01	-0.40043	0.00000	-0.40043
H+	3.83914E-05	3.86947E-05	3.81375E-05	-4.41865	-0.32065	-4.73930
CO3--	2.23756E-01	1.34274E+01	2.22276E-01	-0.65311	-1.28261	-1.93572
CL-	8.40901E-02	2.98125E+00	8.35341E-02	-1.07814	-0.32065	-1.39879
SO4--	2.94742E-04	2.83122E-02	2.92793E-04	-3.53344	-1.28261	-4.81605
FE++	9.24498E-08	5.16305E-06	9.18386E-08	-7.03697	-1.28261	-8.31959
O2(AQ)	1.87676E-16	6.00542E-15	1.86435E-16	-15.72947	0.00000	-15.72947
H2(AQ)	1.31316E-02	2.64708E-02	1.30448E-02	-1.88456	0.00000	-1.88456
CH4(AQ)	5.08577E-02	8.15890E-01	5.05214E-02	-1.29652	0.00000	-1.29652
HS-	4.00429E-02	1.32414E+00	3.97782E-02	-1.40036	-0.32065	-1.72101
FE+++	1.52099E-14	8.49427E-13	1.51093E-14	-13.82075	-2.88588	-16.70663
HCO3-	5.04562E-01	3.07869E+01	5.01226E-01	-0.29997	-0.32065	-0.62062
CLO4-	1.64173E-42	1.63271E-40	1.63087E-42	-41.78758	-0.32065	-42.10823
OH-	5.38005E-01	9.15002E+00	5.34448E-01	-0.27209	-0.32065	-0.59275
HCOO-	2.54654E+00	1.14639E+02	2.52970E+00	0.40307	-0.32065	0.08242
CH3COO-	2.97125E-02	1.75436E+00	2.95160E-02	-1.52994	-0.32065	-1.85060
CH3CH2COO-	4.97538E-01	3.63558E+01	4.94248E-01	-0.30605	-0.32065	-0.62671
CO(AQ)	6.42903E-02	1.80080E+00	6.38652E-02	-1.19474	0.00000	-1.19474
ETHANE(AQ)	2.16344E-04	6.50533E-03	2.14913E-04	-3.66774	0.00000	-3.66774
ETHYLENE(AQ)	4.69415E-07	1.31688E-05	4.66311E-07	-6.33132	0.00000	-6.33132
PROPANE(AQ)	1.23547E-06	5.44796E-05	1.22730E-06	-5.91105	0.00000	-5.91105

HEXANE(AQ)	2.99017E-13	2.57683E-11	2.97040E-13	-12.52719	0.00000	-12.52719
BENZENE(AQ)	9.96425E-13	7.78341E-11	9.89836E-13	-12.00444	0.00000	-12.00444
TOLUENE(AQ)	4.01636E-15	3.70068E-13	3.98980E-15	-14.39905	0.00000	-14.39905
SI2O4(AQ)	2.15487E-02	2.58948E+00	2.14062E-02	-1.66946	0.00000	-1.66946
ALO2-	6.92791E-06	4.08611E-04	6.88210E-06	-5.16228	-0.32065	-5.48293
ALO2(SIO2)-	8.79372E-05	1.04702E-02	8.73558E-05	-4.05871	-0.32065	-4.37936
CACL+	2.32037E-04	1.75265E-02	2.30503E-04	-3.63732	-0.32065	-3.95798
CACL2(AQ)	3.94251E-07	4.37563E-05	3.91644E-07	-6.40711	0.00000	-6.40711
CACO3(AQ)	5.71481E-03	5.71990E-01	5.67702E-03	-2.24588	0.00000	-2.24588
CA(HCO3)+	3.16653E+00	3.20127E+02	3.14559E+00	0.49770	-0.24503	0.25267
CA(OH)+	4.15710E-04	2.37318E-02	4.12962E-04	-3.38409	-0.32065	-3.70474
CA(HSIO3)+	5.45300E-02	6.38936E+00	5.41694E-02	-1.26625	-0.32065	-1.58690
FECL+	5.36298E-08	4.89640E-06	5.32752E-08	-7.27348	-0.32065	-7.59413
FECL2(AQ)	4.40016E-05	5.57733E-03	4.37106E-05	-4.35941	0.00000	-4.35941
FE(HSIO3)+	1.62687E-02	2.16274E+00	1.61611E-02	-1.79153	-0.32065	-2.11218
KCL(AQ)	1.44968E-02	1.08076E+00	1.44010E-02	-1.84161	0.00000	-1.84161
KOH	2.15439E-01	1.20873E+01	2.14014E-01	-0.66956	0.00000	-0.66956
KSO4-	1.26999E-05	1.71646E-03	1.26159E-05	-4.89908	-0.32065	-5.21973
MGCL+	5.12471E-06	3.06242E-04	5.09082E-06	-5.29321	-0.32065	-5.61387
MG(HCO3)+	6.75535E-04	5.76380E-02	6.71068E-04	-3.17323	-0.32065	-3.49389
MG(HSIO3)+	5.35660E-02	5.43142E+00	5.32119E-02	-1.27399	-0.32065	-1.59464
MGSO4(AQ)	9.63120E-09	1.15924E-06	9.56752E-09	-8.01920	0.00000	-8.01920
NACL(AQ)	8.17152E-04	4.77566E-02	8.11749E-04	-3.09058	0.00000	-3.09058
NACO3-	5.43077E-04	4.50748E-02	5.39486E-04	-3.26802	-0.32065	-3.58867
NAHCO3(AQ)	7.04311E-03	5.91669E-01	6.99654E-03	-2.15512	0.00000	-2.15512
NAHSIO3(AQ)	2.29464E-04	2.29651E-02	2.27947E-04	-3.64217	0.00000	-3.64217
NAOH(AQ)	3.10143E-02	1.24048E+00	3.08092E-02	-1.51132	0.00000	-1.51132
HSIO3-	3.31860E-01	2.55836E+01	3.29666E-01	-0.48193	-0.32065	-0.80258
HSO4-	1.89242E-03	1.83689E-01	1.87991E-03	-2.72586	-0.32065	-3.04652
H2S(AQ)	1.43386E-01	4.88600E+00	1.42438E-01	-0.84637	0.00000	-0.84637
CO2(AQ)	2.62922E+01	1.15711E+03	2.61183E+01	1.41695	0.00000	1.41695
HCL(AQ)	2.69904E-04	9.84093E-03	2.68119E-04	-3.57167	0.00000	-3.57167
CH3CH2COOH	5.20604E-05	3.85659E-03	5.17162E-05	-4.28637	0.00000	-4.28637
CH3COOH	1.69982E-03	1.02078E-01	1.68858E-03	-2.77248	0.00000	-2.77248
HCOOH	3.15827E-03	1.45361E-01	3.13739E-03	-2.50343	0.00000	-2.50343

--- activity ratios of cations ---

log (NA+ /h+**0)	3.8313289
log (K+ /h+**0)	4.5830991
log (CA++ /h+**0)	4.3842967
log (MG++ /h+**0)	3.2765512
log (AL+++ /h+**0)	-7.0152883
log (FE++ /h+**0)	1.1590144
log (FE+++ /h+**0)	-2.4887296

--- grand summary of solid phases (e.s.+p.r.s.+reactants) ---

phase/end-member	log moles	moles	grams	volume, cc
COESITE	1.3010300	2.00000E+01	1.20169E+03	4.53760E+02
CLINOPYROXENE(SS)	1.3010300	2.00000E+01		
DIOPSIDE	0.6020600	4.00000E+00	8.66210E+02	2.64800E+02
HEDENBERGITE	0.3010300	2.00000E+00	4.96189E+02	1.32400E+02
JADEITE	1.1461280	1.40000E+01	2.82994E+03	8.44760E+02
GARNET(SS)	1.3010300	2.00000E+01		
PYROPE	1.0791812	1.20000E+01	1.61259E+03	1.35792E+03
ALMANDINE	0.7781512	6.00000E+00	9.95734E+02	6.78960E+02
GROSSULAR	0.3010300	2.00000E+00	3.00346E+02	2.50760E+02

	mass, grams	volume, cc
created	0.000000E+00	0.000000E+00
destroyed	1.312771E-05	6.298245E-06
net	-1.312771E-05	-6.298245E-06

warning-- these volume totals may be incomplete because

of missing partial molar volume data in the data base

--- mineral saturation state summary ---

mineral	affinity, kcal	state	mineral	affinity, kcal	state
DIAMOND	0.0000		BRUCITE	-7.2209	
CALCITE	-4.1051		ARAGONITE	-2.3809	
MAGNESITE	-0.7879		DOLOMITE	-3.6567	
FORSTERITE	-9.8292		ENSTATITE-CL	-4.6060	
ENSTATITE-OR	-4.5110		ENSTATITE-PR	-5.7108	
DIOPSIDE	-8.5220		FERROSILITE	-9.5954	
COESITE	-3.5190		GRAPHITE	-0.3430	
PYRRHOTITE	-3.9971		PYRITE	-4.8369	
FERROUS_OXIDE	-6.7195		SEPIOLITE	1034.8824	ssatd
SIDERITE	-5.0450		QUARTZ-ALPHA	-4.1572	
QUARTZ-BETA	-5.2352		CRISTOBALITE-ALPHA	-8.9596	
CHALCEDONY	-5.1117		AMORPHOUS_SILICA	-6.4505	

--- summary of solid solutions ---

mineral	aff. kcal/mol		mole frac.	lambda	state
ORTHOPYROXENE(SS)	-4.2615				
FERROSILITE		-4.26152	0.1014822	1.00000	
ENSTATITE-OR		-4.26152	0.8985178	1.00000	
OLIVINE	-9.7264				
FAYALITE		-9.72639	0.0431395	1.00000	
FORSTERITE		-9.72639	0.9568605	1.00000	
BIOTITE	-29.4586				
PHLOGOPITE		-29.45858	0.9992091	1.00000	
ANNITE		-29.45858	0.0007909	1.00000	
CLINOPYROXENE(SS)	-8.3055				
DIOPSIDE		-8.30551	0.9113360	1.00000	
HEDENBERGITE		-8.30551	0.0884589	1.00000	
JADEITE		-8.30551	0.0002051	1.00000	
GARNET(SS)	-15.1889				
PYROPE		-15.18895	0.5831516	1.00000	
ALMANDINE		-15.18895	0.1916140	1.00000	
GROSSULAR		-15.18895	0.2252344	1.00000	
CALCITE(SS)	0.0000				
CALCITE		0.00000	0.1719004	1.00000	
MAGNESITE		0.00000	0.7132328	1.00000	
SIDERITE		0.00000	0.1148668	1.00000	

solid solution product phases

xbar	lambda	activity	log xbar	log lambda	log activity

--- summary of gas species ---

gas	log fugacity	fugacity	partial pressure
CO2(G)	9.10553	1.27505E+09	
O2(G)	-9.60427	2.48730E-10	
S2(G)	3.99701	9.93132E+03	
CH4(G)	6.97348	9.40753E+06	
H2(G)	3.80949	6.44893E+03	
H2S(G)	7.27169	1.86935E+07	
H2O(G)	6.90035	7.94972E+06	

- -

```
stepping to zi=  4.7851E-08, delzi=  1.6228E-08, nord= 2
              ncycle=  0
steps completed =     6, iter =  4, ncorr = 0
most rapidly changing is zvclg1(AL+++            ) =    -18.3443

stepping to zi=  1.0000E-07, delzi=  5.2149E-08, nord= 2
              ncycle=  0
steps completed =     7, iter =  5, ncorr = 0
most rapidly changing is zvclg1(AL+++            ) =    -18.3440
- - - - - - - - - - - - - - - - - - - - - - - - - - - - - - - - -


                    reaction progress        =  9.99999999999996E-08
                    log of reaction progress =      -7.0000000

                    temperature    =    900.000 degrees c
                    total pressure =  50000.000 bars

                    computing units remaining =      0.000


step size is limited by the print requirement


                              --- reactant summary ---
```

reactant	moles	delta moles	grams	delta grams
CLINOPYROXENE(SS)	2.00000E+01	1.00000E-07	4.19234E+03	2.09617E-05
GARNET(SS)	2.00000E+01	1.00000E-07	2.90867E+03	1.45433E-05
COESITE	2.00000E+01	1.00000E-07	1.20169E+03	6.00843E-06

```
               current total mass =  8.30269E+03 grams
               delta total mass   =  4.15135E-05 grams
               delta total volume =      0.00002 cc
```

reactant	affinity	rel. rate
CLINOPYROXENE(SS)	20.9022	1.00000E+00
GARNET(SS)	15.3515	1.00000E+00
COESITE	3.5190	1.00000E+00

```
         affinity of the overall irreversible reaction=    39.773 kcal
         contributions from irreversible reactions
         with no thermodynamic data are not included



     --- element totals for the aqueous phase ---
```

element	mg/kg soln.	molal conc.	moles
O	7.273301E+05	1.286050E+02	1.294610E+02
NA	2.421836E+03	2.980164E-01	3.000001E-01
K	2.333972E+04	1.688759E+00	1.700000E+00
CA	4.542477E+04	3.206233E+00	3.227574E+00
MG	4.630793E+02	5.390013E-02	5.425889E-02
AL	8.996830E-01	9.433065E-05	9.495852E-05
SI	8.875926E+03	8.940495E-01	9.000004E-01
H	4.331046E+04	1.215641E+02	1.223733E+02
C	1.451787E+05	3.419429E+01	3.442190E+01
CL	1.244921E+03	9.933879E-02	1.000000E-01
S	2.089768E+03	1.844016E-01	1.856290E-01
FE	3.199036E+02	1.620501E-02	1.631287E-02
co3--		0.000000E+00	0.000000E+00
so4--		0.000000E+00	0.000000E+00
s--		0.000000E+00	0.000000E+00

warning-- co3--, so4--, and s-- totals require that routine comp1
have the names of non-carbonate carbon, sulfide sulfur,
and non-sulfate sulfur aqueous species

single ion activities and activity coefficients are here defined
with respect to the internal ph scale

	ph	eh	pe
internal ph scale	4.7393	1.3458	5.7816E+00
modified nbs ph scale	4.4186	1.4204	6.1023E+00
rational ph scale	4.4186	1.4204	6.1023E+00

phcl = 6.1381

oxygen fugacity = 2.48730E-10
log oxygen fugacity = -9.60427

activity of water = 0.99667
log activity of water = -0.00145
alkalinity = 0.000000E+00 equiv/kg solvent
(not def. for t.gt.50 c)

ionic strength = 5.212520E+00 molal
sum of molalities = 36.8425156503874
osmotic coefficient = 0.00502
equiv. stoich. ionic strength = 9.933879E-02 molal

mass of solution = 2.847811 kg
mass of solvent = 1.006656 kg
mass of solutes = 1.841155 kg
conc of solutes = 64.651584 per cent (w/w)

species	moles	grams	conc	log conc	log g	log act
H2O	5.58782E+01	1.00666E+03				
NA+	2.60353E-01	5.98545E+00	2.58631E-01	-0.58732	-0.32065	-0.90797
K+	1.47005E+00	5.74765E+01	1.46033E+00	0.16445	-0.32065	-0.15620
CA++	1.55306E-04	6.22467E-03	1.54279E-04	-3.81169	-1.28261	-5.09430
MG++	1.21183E-05	2.94536E-04	1.20382E-05	-4.91944	-1.28261	-6.20205
AL+++	4.52892E-19	1.22197E-17	4.49898E-19	-18.34689	-2.88588	-21.23276
SIO2(AQ)	4.00361E-01	2.40554E+01	3.97714E-01	-0.40043	0.00000	-0.40043
H+	3.83914E-05	3.86947E-05	3.81375E-05	-4.41865	-0.32065	-4.73930
CO3--	2.23756E-01	1.34274E+01	2.22276E-01	-0.65311	-1.28261	-1.93572
CL-	8.40901E-02	2.98125E+00	8.35341E-02	-1.07814	-0.32065	-1.39879
SO4--	2.94742E-04	2.83122E-02	2.92793E-04	-3.53344	-1.28261	-4.81605
FE++	9.24500E-08	5.16305E-06	9.18387E-08	-7.03697	-1.28261	-8.31959
O2(AQ)	1.87676E-16	6.00542E-15	1.86435E-16	-15.72947	0.00000	-15.72947
H2(AQ)	1.31316E-02	2.64708E-02	1.30448E-02	-1.88456	0.00000	-1.88456
CH4(AQ)	5.08577E-02	8.15890E-01	5.05214E-02	-1.29652	0.00000	-1.29652
HS-	4.00429E-02	1.32414E+00	3.97782E-02	-1.40036	-0.32065	-1.72101
FE+++	1.52099E-14	8.49429E-13	1.51094E-14	-13.82075	-2.88588	-16.70663
HCO3-	5.04562E-01	3.07869E+01	5.01226E-01	-0.29997	-0.32065	-0.62062
CLO4-	1.64173E-42	1.63271E-40	1.63087E-42	-41.78758	-0.32065	-42.10823
OH-	5.38005E-01	9.15002E+00	5.34448E-01	-0.27209	-0.32065	-0.59275
HCOO-	2.54654E+00	1.14639E+02	2.52970E+00	0.40307	-0.32065	0.08242
CH3COO-	2.97125E-02	1.75436E+00	2.95160E-02	-1.52994	-0.32065	-1.85060
CH3CH2COO-	4.97538E-01	3.63558E+01	4.94248E-01	-0.30605	-0.32065	-0.62671
CO(AQ)	6.42903E-02	1.80080E+00	6.38652E-02	-1.19474	0.00000	-1.19474
ETHANE(AQ)	2.16344E-04	6.50533E-03	2.14913E-04	-3.66774	0.00000	-3.66774
ETHYLENE(AQ)	4.69415E-07	1.31688E-05	4.66311E-07	-6.33132	0.00000	-6.33132
PROPANE(AQ)	1.23547E-06	5.44796E-05	1.22730E-06	-5.91105	0.00000	-5.91105
HEXANE(AQ)	2.99017E-13	2.57683E-11	2.97040E-13	-12.52719	0.00000	-12.52719
BENZENE(AQ)	9.96425E-13	7.78341E-11	9.89836E-13	-12.00444	0.00000	-12.00444
TOLUENE(AQ)	4.01636E-15	3.70068E-13	3.98980E-15	-14.39905	0.00000	-14.39905
SI2O4(AQ)	2.15487E-02	2.58948E+00	2.14063E-02	-1.66946	0.00000	-1.66946
ALO2-	6.93473E-06	4.09013E-04	6.88888E-06	-5.16185	-0.32065	-5.48250
ALO2(SIO2)-	8.80238E-05	1.04805E-02	8.74418E-05	-4.05828	-0.32065	-4.37893

CACL+	2.32037E-04	1.75265E-02	2.30503E-04	-3.63732	-0.32065	-3.95798
CACL2(AQ)	3.94251E-07	4.37563E-05	3.91644E-07	-6.40711	0.00000	-6.40711
CACO3(AQ)	5.71481E-03	5.71990E-01	5.67702E-03	-2.24588	0.00000	-2.24588
CA(HCO3)+	3.16653E+00	3.20127E+02	3.14559E+00	0.49770	-0.24503	0.25267
CA(OH)+	4.15710E-04	2.37318E-02	4.12962E-04	-3.38409	-0.32065	-3.70474
CA(HSIO3)+	5.45300E-02	6.38936E+00	5.41694E-02	-1.26625	-0.32065	-1.58690
FECL+	5.36299E-08	4.89641E-06	5.32753E-08	-7.27347	-0.32065	-7.59413
FECL2(AQ)	4.40016E-05	5.57734E-03	4.37107E-05	-4.35941	0.00000	-4.35941
FE(HSIO3)+	1.62687E-02	2.16274E+00	1.61612E-02	-1.79153	-0.32065	-2.11218
KCL(AQ)	1.44968E-02	1.08076E+00	1.44010E-02	-1.84161	0.00000	-1.84161
KOH	2.15439E-01	1.20873E+01	2.14014E-01	-0.66956	0.00000	-0.66956
KSO4-	1.26999E-05	1.71646E-03	1.26159E-05	-4.89908	-0.32065	-5.21973
MGCL+	5.12471E-06	3.06242E-04	5.09083E-06	-5.29321	-0.32065	-5.61386
MG(HCO3)+	6.75535E-04	5.76381E-02	6.71069E-04	-3.17323	-0.32065	-3.49389
MG(HSIO3)+	5.35661E-02	5.43142E+00	5.32119E-02	-1.27399	-0.32065	-1.59464
MGSO4(AQ)	9.63121E-09	1.15924E-06	9.56753E-09	-8.01920	0.00000	-8.01920
NACL(AQ)	8.17152E-04	4.77566E-02	8.11749E-04	-3.09058	0.00000	-3.09058
NACO3-	5.43077E-04	4.50748E-02	5.39486E-04	-3.26802	-0.32065	-3.58867
NAHCO3(AQ)	7.04311E-03	5.91670E-01	6.99654E-03	-2.15512	0.00000	-2.15512
NAHSIO3(AQ)	2.29464E-04	2.29651E-02	2.27947E-04	-3.64217	0.00000	-3.64217
NAOH(AQ)	3.10143E-02	1.24048E+00	3.08092E-02	-1.51132	0.00000	-1.51132
HSIO3-	3.31860E-01	2.55836E+01	3.29666E-01	-0.48193	-0.32065	-0.80258
HSO4-	1.89242E-03	1.83689E-01	1.87991E-03	-2.72586	-0.32065	-3.04652
H2S(AQ)	1.43386E-01	4.88600E+00	1.42438E-01	-0.84637	0.00000	-0.84637
CO2(AQ)	2.62922E+01	1.15711E+03	2.61183E+01	1.41695	0.00000	1.41695
HCL(AQ)	2.69904E-04	9.84093E-03	2.68119E-04	-3.57167	0.00000	-3.57167
CH3CH2COOH	5.20604E-05	3.85659E-03	5.17162E-05	-4.28637	0.00000	-4.28637
CH3COOH	1.69982E-03	1.02078E-01	1.68858E-03	-2.77248	0.00000	-2.77248
HCOOH	3.15827E-03	1.45361E-01	3.13739E-03	-2.50343	0.00000	-2.50343

--- activity ratios of cations ---

log (NA+ /h+**0)	3.8313290
log (K+ /h+**0)	4.5830991
log (CA++ /h+**0)	4.3842967
log (MG++ /h+**0)	3.2765516
log (AL+++ /h+**0)	-7.0148609
log (FE++ /h+**0)	1.1590150
log (FE+++ /h+**0)	-2.4887289

--- grand summary of solid phases (e.s.+p.r.s.+reactants) ---

phase/end-member	log moles	moles	grams	volume, cc
COESITE	1.3010300	2.00000E+01	1.20169E+03	4.53760E+02
CLINOPYROXENE(SS)	1.3010300	2.00000E+01		
DIOPSIDE	0.6020600	4.00000E+00	8.66210E+02	2.64800E+02
HEDENBERGITE	0.3010300	2.00000E+00	4.96189E+02	1.32400E+02
JADEITE	1.1461280	1.40000E+01	2.82994E+03	8.44760E+02
GARNET(SS)	1.3010300	2.00000E+01		
PYROPE	1.0791812	1.20000E+01	1.61259E+03	1.35792E+03
ALMANDINE	0.7781512	6.00000E+00	9.95734E+02	6.78960E+02
GROSSULAR	0.3010300	2.00000E+00	3.00346E+02	2.50760E+02

	mass, grams	volume, cc
created	0.000000E+00	0.000000E+00
destroyed	4.151346E-05	1.991680E-05
net	-4.151346E-05	-1.991680E-05

warning-- these volume totals may be incomplete because
of missing partial molar volume data in the data base

--- mineral saturation state summary ---

mineral	affinity, kcal	state	mineral	affinity, kcal	state
DIAMOND	0.0000		BRUCITE	-7.2209	
CALCITE	-4.1051		ARAGONITE	-2.3809	
MAGNESITE	-0.7879		DOLOMITE	-3.6567	
FORSTERITE	-9.8292		ENSTATITE-CL	-4.6060	
ENSTATITE-OR	-4.5110		ENSTATITE-PR	-5.7108	
DIOPSIDE	-8.5220		FERROSILITE	-9.5954	
COESITE	-3.5190		GRAPHITE	-0.3430	
PYRRHOTITE	-3.9971		PYRITE	-4.8369	
FERROUS_OXIDE	-6.7195		SEPIOLITE	1034.8824	ssatd
SIDERITE	-5.0450		QUARTZ-ALPHA	-4.1572	
QUARTZ-BETA	-5.2352		CRISTOBALITE-ALPHA	-8.9596	
CHALCEDONY	-5.1117		AMORPHOUS_SILICA	-6.4505	

--- summary of solid solutions ---

mineral	aff. kcal/mol	mole frac.	lambda	state
ORTHOPYROXENE(SS)	-4.2615			
FERROSILITE	-4.26151	0.1014823	1.00000	
ENSTATITE-OR	-4.26151	0.8985177	1.00000	
OLIVINE	-9.7264			
FAYALITE	-9.72638	0.0431395	1.00000	
FORSTERITE	-9.72638	0.9568605	1.00000	
BIOTITE	-29.4563			
PHLOGOPITE	-29.45628	0.9992091	1.00000	
ANNITE	-29.45628	0.0007909	1.00000	
CLINOPYROXENE(SS)	-8.3055			
DIOPSIDE	-8.30551	0.9113358	1.00000	
HEDENBERGITE	-8.30551	0.0884590	1.00000	
JADEITE	-8.30551	0.0002053	1.00000	
GARNET(SS)	-15.1874			
PYROPE	-15.18742	0.5831517	1.00000	
ALMANDINE	-15.18742	0.1916141	1.00000	
GROSSULAR	-15.18742	0.2252342	1.00000	
CALCITE(SS)	0.0000			
CALCITE	0.00000	0.1719002	1.00000	
MAGNESITE	0.00000	0.7132329	1.00000	
SIDERITE	0.00000	0.1148669	1.00000	

solid solution product phases

xbar	lambda	activity	log xbar	log lambda	log activity

--- summary of gas species ---

gas	log fugacity	fugacity	partial pressure
CO2(G)	9.10553	1.27505E+09	
O2(G)	-9.60427	2.48730E-10	
S2(G)	3.99701	9.93132E+03	
CH4(G)	6.97348	9.40753E+06	
H2(G)	3.80949	6.44893E+03	
H2S(G)	7.27169	1.86935E+07	
H2O(G)	6.90035	7.94972E+06	

- -

```
stepping to zi=  3.1623E-07, delzi=  2.1623E-07, nord= 2
              ncycle=  0
steps completed =     8, iter =  7, ncorr = 0
most rapidly changing is zvclg1(AL+++             ) =    -18.3427
```

- -

reaction progress   = 3.16227766016836E-07
log of reaction progress =   -6.5000000

temperature   =   900.000 degrees c
total pressure = 50000.000 bars

computing units remaining =   0.000

step size is limited by the print requirement

--- reactant summary ---

reactant	moles	delta moles	grams	delta grams
CLINOPYROXENE(SS)	2.00000E+01	3.16228E-07	4.19234E+03	6.62867E-05
GARNET(SS)	2.00000E+01	3.16228E-07	2.90866E+03	4.59900E-05
COESITE	2.00000E+01	3.16228E-07	1.20169E+03	1.90003E-05

current total mass = 8.30269E+03 grams
delta total mass   = 1.31277E-04 grams
delta total volume =   0.00006 cc

reactant	affinity	rel. rate
CLINOPYROXENE(SS)	20.8971	1.00000E+00
GARNET(SS)	15.3467	1.00000E+00
COESITE	3.5190	1.00000E+00

affinity of the overall irreversible reaction=   39.763 kcal
contributions from irreversible reactions
with no thermodynamic data are not included

--- element totals for the aqueous phase ---

element	mg/kg soln.	molal conc.	moles
O	7.273300E+05	1.286050E+02	1.294610E+02
NA	2.421838E+03	2.980166E-01	3.000002E-01
K	2.333972E+04	1.688759E+00	1.700000E+00
CA	4.542477E+04	3.206233E+00	3.227574E+00
MG	4.630808E+02	5.390030E-02	5.425906E-02
AL	9.024814E-01	9.462406E-05	9.525389E-05
SI	8.875935E+03	8.940504E-01	9.000013E-01
H	4.331046E+04	1.215641E+02	1.223733E+02
C	1.451787E+05	3.419430E+01	3.442190E+01
CL	1.244921E+03	9.933879E-02	1.000000E-01
S	2.089768E+03	1.844016E-01	1.856290E-01
FE	3.199053E+02	1.620510E-02	1.631296E-02
co3--		0.000000E+00	0.000000E+00
so4--		0.000000E+00	0.000000E+00
s--		0.000000E+00	0.000000E+00

warning-- co3--, so4--, and s-- totals require that routine comp1
have the names of non-carbonate carbon, sulfide sulfur,
and non-sulfate sulfur aqueous species

single ion activities and activity coefficients are here defined
with respect to the internal ph scale

ph   eh   pe

```
  internal ph scale              4.7393       1.3458    5.7816E+00
  modified nbs ph scale          4.4186       1.4204    6.1023E+00
  rational ph scale              4.4186       1.4204    6.1023E+00

phcl =      6.1381



       oxygen fugacity =  2.48730E-10
       log oxygen fugacity =  -9.60427



                         activity of water =   0.99667
            log activity of water =  -0.00145
            alkalinity =  0.000000E+00 equiv/kg solvent
                                    (not def. for t.gt.50 c)

                 ionic strength =  5.212521E+00 molal
              sum of molalities =   36.8425168411306
            osmotic coefficient =    0.00502
                 equiv. stoich. ionic strength =  9.933879E-02 molal

                      mass of solution =   2.847811 kg
                      mass of solvent  =   1.006656 kg
                      mass of solutes  =   1.841155 kg
                      conc of solutes  =  64.651586 per cent (w/w)
```

species	moles	grams	conc	log conc	log g	log act
H2O	5.58782E+01	1.00666E+03				
NA+	2.60353E-01	5.98546E+00	2.58632E-01	-0.58732	-0.32065	-0.90797
K+	1.47005E+00	5.74765E+01	1.46033E+00	0.16445	-0.32065	-0.15620
CA++	1.55306E-04	6.22467E-03	1.54279E-04	-3.81169	-1.28261	-5.09430
MG++	1.21184E-05	2.94537E-04	1.20383E-05	-4.91944	-1.28261	-6.20205
AL+++	4.54301E-19	1.22577E-17	4.51297E-19	-18.34554	-2.88588	-21.23141
SIO2(AQ)	4.00361E-01	2.40554E+01	3.97714E-01	-0.40043	0.00000	-0.40043
H+	3.83914E-05	3.86947E-05	3.81375E-05	-4.41865	-0.32065	-4.73930
CO3--	2.23756E-01	1.34274E+01	2.22276E-01	-0.65311	-1.28261	-1.93572
CL-	8.40901E-02	2.98125E+00	8.35341E-02	-1.07814	-0.32065	-1.39879
SO4--	2.94742E-04	2.83122E-02	2.92793E-04	-3.53344	-1.28261	-4.81605
FE++	9.24504E-08	5.16308E-06	9.18392E-08	-7.03697	-1.28261	-8.31958
O2(AQ)	1.87676E-16	6.00542E-15	1.86435E-16	-15.72947	0.00000	-15.72947
H2(AQ)	1.31316E-02	2.64708E-02	1.30448E-02	-1.88456	0.00000	-1.88456
CH4(AQ)	5.08577E-02	8.15890E-01	5.05214E-02	-1.29652	0.00000	-1.29652
HS-	4.00429E-02	1.32414E+00	3.97782E-02	-1.40036	-0.32065	-1.72101
FE+++	1.52100E-14	8.49433E-13	1.51094E-14	-13.82075	-2.88588	-16.70663
HCO3-	5.04562E-01	3.07869E+01	5.01226E-01	-0.29997	-0.32065	-0.62062
CLO4-	1.64173E-42	1.63271E-40	1.63087E-42	-41.78758	-0.32065	-42.10823
OH-	5.38005E-01	9.15002E+00	5.34448E-01	-0.27209	-0.32065	-0.59275
HCOO-	2.54654E+00	1.14639E+02	2.52970E+00	0.40307	-0.32065	0.08242
CH3COO-	2.97125E-02	1.75436E+00	2.95160E-02	-1.52994	-0.32065	-1.85060
CH3CH2COO-	4.97538E-01	3.63558E+01	4.94248E-01	-0.30605	-0.32065	-0.62671
CO(AQ)	6.42903E-02	1.80080E+00	6.38652E-02	-1.19474	0.00000	-1.19474
ETHANE(AQ)	2.16344E-04	6.50533E-03	2.14913E-04	-3.66774	0.00000	-3.66774
ETHYLENE(AQ)	4.69415E-07	1.31688E-05	4.66311E-07	-6.33132	0.00000	-6.33132
PROPANE(AQ)	1.23547E-06	5.44796E-05	1.22730E-06	-5.91105	0.00000	-5.91105
HEXANE(AQ)	2.99017E-13	2.57683E-11	2.97040E-13	-12.52719	0.00000	-12.52719
BENZENE(AQ)	9.96425E-13	7.78341E-11	9.89836E-13	-12.00444	0.00000	-12.00444
TOLUENE(AQ)	4.01636E-15	3.70068E-13	3.98980E-15	-14.39905	0.00000	-14.39905
SI2O4(AQ)	2.15488E-02	2.58948E+00	2.14063E-02	-1.66946	0.00000	-1.66946
ALO2-	6.95630E-06	4.10285E-04	6.91031E-06	-5.16050	-0.32065	-5.48116
ALO2(SIO2)-	8.82976E-05	1.05131E-02	8.77138E-05	-4.05693	-0.32065	-4.37759
CACL+	2.32037E-04	1.75265E-02	2.30503E-04	-3.63732	-0.32065	-3.95798
CACL2(AQ)	3.94251E-07	4.37563E-05	3.91644E-07	-6.40711	0.00000	-6.40711
CACO3(AQ)	5.71481E-03	5.71990E-01	5.67702E-03	-2.24588	0.00000	-2.24588
CA(HCO3)+	3.16653E+00	3.20127E+02	3.14559E+00	0.49770	-0.24503	0.25267
CA(OH)+	4.15710E-04	2.37318E-02	4.12962E-04	-3.38409	-0.32065	-3.70474
CA(HSIO3)+	5.45300E-02	6.38937E+00	5.41694E-02	-1.26625	-0.32065	-1.58690
FECL+	5.36301E-08	4.89643E-06	5.32755E-08	-7.27347	-0.32065	-7.59413
FECL2(AQ)	4.40019E-05	5.57737E-03	4.37109E-05	-4.35941	0.00000	-4.35941
FE(HSIO3)+	1.62688E-02	2.16275E+00	1.61612E-02	-1.79153	-0.32065	-2.11218
KCL(AQ)	1.44968E-02	1.08076E+00	1.44010E-02	-1.84161	0.00000	-1.84161
KOH	2.15439E-01	1.20873E+01	2.14014E-01	-0.66956	0.00000	-0.66956

KSO4-	1.26999E-05	1.71646E-03	1.26159E-05	-4.89908	-0.32065	-5.21973
MGCL+	5.12472E-06	3.06243E-04	5.09084E-06	-5.29321	-0.32065	-5.61386
MG(HCO3)+	6.75537E-04	5.76383E-02	6.71071E-04	-3.17323	-0.32065	-3.49388
MG(HSIO3)+	5.35663E-02	5.43144E+00	5.32121E-02	-1.27399	-0.32065	-1.59464
MGSO4(AQ)	9.63123E-09	1.15924E-06	9.56755E-09	-8.01920	0.00000	-8.01920
NACL(AQ)	8.17153E-04	4.77567E-02	8.11750E-04	-3.09058	0.00000	-3.09058
NACO3-	5.43077E-04	4.50748E-02	5.39486E-04	-3.26802	-0.32065	-3.58867
NAHCO3(AQ)	7.04311E-03	5.91670E-01	6.99654E-03	-2.15512	0.00000	-2.15512
NAHSIO3(AQ)	2.29464E-04	2.29651E-02	2.27947E-04	-3.64217	0.00000	-3.64217
NAOH(AQ)	3.10143E-02	1.24048E+00	3.08093E-02	-1.51132	0.00000	-1.51132
HSIO3-	3.31860E-01	2.55836E+01	3.29666E-01	-0.48193	-0.32065	-0.80258
HSO4-	1.89242E-03	1.83689E-01	1.87991E-03	-2.72586	-0.32065	-3.04652
H2S(AQ)	1.43386E-01	4.88600E+00	1.42438E-01	-0.84637	0.00000	-0.84637
CO2(AQ)	2.62922E+01	1.15711E+03	2.61183E+01	1.41695	0.00000	1.41695
HCL(AQ)	2.69904E-04	9.84093E-03	2.68119E-04	-3.57167	0.00000	-3.57167
CH3CH2COOH	5.20604E-05	3.85659E-03	5.17162E-05	-4.28637	0.00000	-4.28637
CH3COOH	1.69982E-03	1.02078E-01	1.68858E-03	-2.77248	0.00000	-2.77248
HCOOH	3.15827E-03	1.45361E-01	3.13739E-03	-2.50343	0.00000	-2.50343

--- activity ratios of cations ---

log (NA+ /h+**0)	3.8313292
log (K+ /h+**0)	4.5830991
log (CA++ /h+**0)	4.3842967
log (MG++ /h+**0)	3.2765528
log (AL+++ /h+**0)	-7.0135123
log (FE++ /h+**0)	1.1590172
log (FE+++ /h+**0)	-2.4887267

--- grand summary of solid phases (e.s.+p.r.s.+reactants) ---

phase/end-member	log moles	moles	grams	volume, cc
COESITE	1.3010300	2.00000E+01	1.20169E+03	4.53760E+02
CLINOPYROXENE(SS)	1.3010300	2.00000E+01		
DIOPSIDE	0.6020600	4.00000E+00	8.66210E+02	2.64800E+02
HEDENBERGITE	0.3010300	2.00000E+00	4.96189E+02	1.32400E+02
JADEITE	1.1461280	1.40000E+01	2.82994E+03	8.44760E+02
GARNET(SS)	1.3010300	2.00000E+01		
PYROPE	1.0791812	1.20000E+01	1.61259E+03	1.35792E+03
ALMANDINE	0.7781512	6.00000E+00	9.95734E+02	6.78960E+02
GROSSULAR	0.3010300	2.00000E+00	3.00346E+02	2.50760E+02

	mass, grams	volume, cc
created	0.000000E+00	0.000000E+00
destroyed	1.312771E-04	6.298245E-05
net	-1.312771E-04	-6.298245E-05

warning-- these volume totals may be incomplete because
of missing partial molar volume data in the data base

--- mineral saturation state summary ---

mineral	affinity, kcal	state	mineral	affinity, kcal	state
DIAMOND	0.0000		BRUCITE	-7.2209	
CALCITE	-4.1051		ARAGONITE	-2.3809	
MAGNESITE	-0.7879		DOLOMITE	-3.6567	
FORSTERITE	-9.8292		ENSTATITE-CL	-4.6060	
ENSTATITE-OR	-4.5110		ENSTATITE-PR	-5.7108	
DIOPSIDE	-8.5220		FERROSILITE	-9.5953	
COESITE	-3.5190		GRAPHITE	-0.3430	
PYRRHOTITE	-3.9971		PYRITE	-4.8369	

```
FERROUS_OXIDE          -6.7195               SEPIOLITE            1034.8824  ssatd
SIDERITE               -5.0450               QUARTZ-ALPHA           -4.1572
QUARTZ-BETA            -5.2352               CRISTOBALITE-ALPHA     -8.9596
CHALCEDONY             -5.1117               AMORPHOUS_SILICA       -6.4505


                    --- summary of solid solutions  ---

mineral               aff. kcal/mol  mole frac.    lambda      state

ORTHOPYROXENE(SS)        -4.2615
    FERROSILITE               -4.26151    0.1014824     1.00000
    ENSTATITE-OR              -4.26151    0.8985176     1.00000

OLIVINE                  -9.7264
    FAYALITE                  -9.72637    0.0431397     1.00000
    FORSTERITE                -9.72637    0.9568603     1.00000

BIOTITE                 -29.4490
    PHLOGOPITE               -29.44902    0.9992090     1.00000
    ANNITE                   -29.44902    0.0007910     1.00000

CLINOPYROXENE(SS)        -8.3055
    DIOPSIDE                  -8.30550    0.9113350     1.00000
    HEDENBERGITE              -8.30550    0.0884591     1.00000
    JADEITE                   -8.30550    0.0002059     1.00000

GARNET(SS)              -15.1826
    PYROPE                   -15.18259    0.5831518     1.00000
    ALMANDINE                -15.18259    0.1916145     1.00000
    GROSSULAR                -15.18259    0.2252337     1.00000

CALCITE(SS)               0.0000
    CALCITE                    0.00001    0.1718998     1.00000
    MAGNESITE                  0.00001    0.7132331     1.00000
    SIDERITE                   0.00001    0.1148671     1.00000


solid solution product phases

           xbar     lambda   activity  log xbar  log lambda log activity


          --- summary of gas species ---

          gas               log fugacity         fugacity         partial pressure

  CO2(G)                        9.10553       1.27505E+09
  O2(G)                        -9.60427       2.48730E-10
  S2(G)                         3.99701       9.93132E+03
  CH4(G)                        6.97348       9.40753E+06
  H2(G)                         3.80949       6.44893E+03
  H2S(G)                        7.27169       1.86935E+07
  H2O(G)                        6.90035       7.94972E+06



- - - - - - - - - - - - - - - - - - - - - - - - - - - - - - - -



stepping to zi=  1.0000E-06, delzi=  6.8377E-07, nord= 2
              ncycle=  0
steps completed =     9, iter =  8, ncorr = 0
most rapidly changing is zvclg1(AL+++            ) =    -18.3384
- - - - - - - - - - - - - - - - - - - - - - - - - - - - - - - -



                    reaction progress        =  9.99999999999995E-07
                    log of reaction progress =      -6.0000000

                    temperature    =    900.000 degrees c
                    total pressure =  50000.000 bars

                    computing units remaining =      0.000
```

step size is limited by the print requirement

--- reactant summary ---

reactant	moles	delta moles	grams	delta grams
CLINOPYROXENE(SS)	2.00000E+01	1.00000E-06	4.19234E+03	2.09617E-04
GARNET(SS)	2.00000E+01	1.00000E-06	2.90866E+03	1.45433E-04
COESITE	2.00000E+01	1.00000E-06	1.20169E+03	6.00843E-05

current total mass = 8.30269E+03 grams
delta total mass = 4.15135E-04 grams
delta total volume = 0.00020 cc

reactant	affinity	rel. rate
CLINOPYROXENE(SS)	20.8811	1.00000E+00
GARNET(SS)	15.3315	1.00000E+00
COESITE	3.5190	1.00000E+00

affinity of the overall irreversible reaction= 39.732 kcal
contributions from irreversible reactions
with no thermodynamic data are not included

--- element totals for the aqueous phase ---

element	mg/kg soln.	molal conc.	moles
O	7.273300E+05	1.286050E+02	1.294610E+02
NA	2.421841E+03	2.980171E-01	3.000007E-01
K	2.333972E+04	1.688759E+00	1.700000E+00
CA	4.542477E+04	3.206234E+00	3.227574E+00
MG	4.630854E+02	5.390084E-02	5.425961E-02
AL	9.113308E-01	9.555192E-05	9.618792E-05
SI	8.875961E+03	8.940531E-01	9.000040E-01
H	4.331045E+04	1.215641E+02	1.223733E+02
C	1.451786E+05	3.419430E+01	3.442190E+01
CL	1.244921E+03	9.933879E-02	1.000000E-01
S	2.089768E+03	1.844016E-01	1.856290E-01
FE	3.199106E+02	1.620537E-02	1.631323E-02
co3--		0.000000E+00	0.000000E+00
so4--		0.000000E+00	0.000000E+00
s--		0.000000E+00	0.000000E+00

warning-- co3--, so4--, and s-- totals require that routine comp1
have the names of non-carbonate carbon, sulfide sulfur,
and non-sulfate sulfur aqueous species

single ion activities and activity coefficients are here defined
with respect to the internal ph scale

	ph	eh	pe
internal ph scale	4.7393	1.3458	5.7816E+00
modified nbs ph scale	4.4186	1.4204	6.1023E+00
rational ph scale	4.4186	1.4204	6.1023E+00

phcl = 6.1381

oxygen fugacity = 2.48730E-10
log oxygen fugacity = -9.60427

activity of water = 0.99667
log activity of water = -0.00145
alkalinity = 0.000000E+00 equiv/kg solvent
(not def. for t.gt.50 c)

ionic strength = 5.212522E+00 molal
sum of molalities = 36.8425206064619
osmotic coefficient = 0.00502
equiv. stoich. ionic strength = 9.933879E-02 molal

mass of solution = 2.847811 kg
mass of solvent = 1.006656 kg
mass of solutes = 1.841155 kg
conc of solutes = 64.651590 per cent (w/w)

species	moles	grams	conc	log conc	log g	log act
H2O	5.58782E+01	1.00666E+03				
NA+	2.60354E-01	5.98547E+00	2.58632E-01	-0.58732	-0.32065	-0.90797
K+	1.47005E+00	5.74765E+01	1.46033E+00	0.16445	-0.32065	-0.15620
CA++	1.55306E-04	6.22467E-03	1.54279E-04	-3.81169	-1.28261	-5.09430
MG++	1.21185E-05	2.94540E-04	1.20384E-05	-4.91943	-1.28261	-6.20204
AL+++	4.58755E-19	1.23779E-17	4.55722E-19	-18.34130	-2.88588	-21.22718
SIO2(AQ)	4.00361E-01	2.40554E+01	3.97714E-01	-0.40043	0.00000	-0.40043
H+	3.83914E-05	3.86947E-05	3.81375E-05	-4.41865	-0.32065	-4.73930
CO3--	2.23756E-01	1.34274E+01	2.22276E-01	-0.65311	-1.28261	-1.93572
CL-	8.40901E-02	2.98125E+00	8.35341E-02	-1.07814	-0.32065	-1.39879
SO4--	2.94742E-04	2.83122E-02	2.92793E-04	-3.53344	-1.28261	-4.81605
FE++	9.24519E-08	5.16316E-06	9.18406E-08	-7.03697	-1.28261	-8.31958
O2(AQ)	1.87676E-16	6.00542E-15	1.86435E-16	-15.72947	0.00000	-15.72947
H2(AQ)	1.31316E-02	2.64708E-02	1.30448E-02	-1.88456	0.00000	-1.88456
CH4(AQ)	5.08577E-02	8.15890E-01	5.05214E-02	-1.29652	0.00000	-1.29652
HS-	4.00429E-02	1.32414E+00	3.97782E-02	-1.40036	-0.32065	-1.72101
FE+++	1.52102E-14	8.49446E-13	1.51097E-14	-13.82074	-2.88588	-16.70662
HCO3-	5.04562E-01	3.07869E+01	5.01226E-01	-0.29997	-0.32065	-0.62062
CLO4-	1.64173E-42	1.63271E-40	1.63087E-42	-41.78758	-0.32065	-42.10823
OH-	5.38005E-01	9.15002E+00	5.34448E-01	-0.27209	-0.32065	-0.59275
HCOO-	2.54654E+00	1.14639E+02	2.52970E+00	0.40307	-0.32065	0.08242
CH3COO-	2.97125E-02	1.75436E+00	2.95160E-02	-1.52994	-0.32065	-1.85060
CH3CH2COO-	4.97538E-01	3.63558E+01	4.94248E-01	-0.30605	-0.32065	-0.62671
CO(AQ)	6.42903E-02	1.80080E+00	6.38652E-02	-1.19474	0.00000	-1.19474
ETHANE(AQ)	2.16344E-04	6.50533E-03	2.14913E-04	-3.66774	0.00000	-3.66774
ETHYLENE(AQ)	4.69415E-07	1.31688E-05	4.66311E-07	-6.33132	0.00000	-6.33132
PROPANE(AQ)	1.23547E-06	5.44795E-05	1.22730E-06	-5.91105	0.00000	-5.91105
HEXANE(AQ)	2.99017E-13	2.57683E-11	2.97040E-13	-12.52719	0.00000	-12.52719
BENZENE(AQ)	9.96425E-13	7.78341E-11	9.89836E-13	-12.00444	0.00000	-12.00444
TOLUENE(AQ)	4.01636E-15	3.70068E-13	3.98980E-15	-14.39905	0.00000	-14.39905
SI2O4(AQ)	2.15488E-02	2.58949E+00	2.14063E-02	-1.66946	0.00000	-1.66946
ALO2-	7.02450E-06	4.14308E-04	6.97806E-06	-5.15627	-0.32065	-5.47692
ALO2(SIO2)-	8.91634E-05	1.06162E-02	8.85739E-05	-4.05269	-0.32065	-4.37335
CACL+	2.32037E-04	1.75265E-02	2.30503E-04	-3.63732	-0.32065	-3.95798
CACL2(AQ)	3.94251E-07	4.37563E-05	3.91644E-07	-6.40711	0.00000	-6.40711
CACO3(AQ)	5.71481E-03	5.71990E-01	5.67702E-03	-2.24588	0.00000	-2.24588
CA(HCO3)+	3.16653E+00	3.20127E+02	3.14559E+00	0.49770	-0.24503	0.25267
CA(OH)+	4.15710E-04	2.37318E-02	4.12962E-04	-3.38409	-0.32065	-3.70474
CA(HSIO3)+	5.45301E-02	6.38937E+00	5.41695E-02	-1.26625	-0.32065	-1.58690
FECL+	5.36310E-08	4.89651E-06	5.32764E-08	-7.27347	-0.32065	-7.59412
FECL2(AQ)	4.40025E-05	5.57745E-03	4.37116E-05	-4.35940	0.00000	-4.35940
FE(HSIO3)+	1.62691E-02	2.16279E+00	1.61615E-02	-1.79152	-0.32065	-2.11217
KCL(AQ)	1.44968E-02	1.08076E+00	1.44010E-02	-1.84161	0.00000	-1.84161
KOH	2.15439E-01	1.20873E+01	2.14014E-01	-0.66956	0.00000	-0.66956
KSO4-	1.26999E-05	1.71646E-03	1.26159E-05	-4.89908	-0.32065	-5.21973
MGCL+	5.12477E-06	3.06246E-04	5.09088E-06	-5.29321	-0.32065	-5.61386
MG(HCO3)+	6.75543E-04	5.76388E-02	6.71077E-04	-3.17323	-0.32065	-3.49388
MG(HSIO3)+	5.35668E-02	5.43149E+00	5.32126E-02	-1.27399	-0.32065	-1.59464
MGSO4(AQ)	9.63132E-09	1.15925E-06	9.56764E-09	-8.01920	0.00000	-8.01920
NACL(AQ)	8.17154E-04	4.77567E-02	8.11751E-04	-3.09058	0.00000	-3.09058
NACO3-	5.43078E-04	4.50749E-02	5.39487E-04	-3.26802	-0.32065	-3.58867
NAHCO3(AQ)	7.04312E-03	5.91671E-01	6.99655E-03	-2.15512	0.00000	-2.15512
NAHSIO3(AQ)	2.29465E-04	2.29652E-02	2.27948E-04	-3.64216	0.00000	-3.64216
NAOH(AQ)	3.10144E-02	1.24048E+00	3.08093E-02	-1.51132	0.00000	-1.51132
HSIO3-	3.31860E-01	2.55837E+01	3.29666E-01	-0.48193	-0.32065	-0.80258

HSO4-	1.89242E-03	1.83689E-01	1.87991E-03	-2.72586	-0.32065	-3.04652
H2S(AQ)	1.43386E-01	4.88600E+00	1.42438E-01	-0.84637	0.00000	-0.84637
CO2(AQ)	2.62922E+01	1.15711E+03	2.61183E+01	1.41695	0.00000	1.41695
HCL(AQ)	2.69904E-04	9.84093E-03	2.68119E-04	-3.57167	0.00000	-3.57167
CH3CH2COOH	5.20604E-05	3.85659E-03	5.17162E-05	-4.28637	0.00000	-4.28637
CH3COOH	1.69982E-03	1.02078E-01	1.68858E-03	-2.77248	0.00000	-2.77248
HCOOH	3.15827E-03	1.45361E-01	3.13739E-03	-2.50343	0.00000	-2.50343

--- activity ratios of cations ---

log (NA+ /h+**0)	3.8313299
log (K+ /h+**0)	4.5830991
log (CA++ /h+**0)	4.3842967
log (MG++ /h+**0)	3.2765567
log (AL+++ /h+**0)	-7.0092750
log (FE++ /h+**0)	1.1590239
log (FE+++ /h+**0)	-2.4887200

--- grand summary of solid phases (e.s.+p.r.s.+reactants) ---

phase/end-member	log moles	moles	grams	volume, cc
COESITE	1.3010300	2.00000E+01	1.20169E+03	4.53760E+02
CLINOPYROXENE(SS)	1.3010300	2.00000E+01		
DIOPSIDE	0.6020600	4.00000E+00	8.66210E+02	2.64800E+02
HEDENBERGITE	0.3010300	2.00000E+00	4.96189E+02	1.32400E+02
JADEITE	1.1461280	1.40000E+01	2.82994E+03	8.44760E+02
GARNET(SS)	1.3010300	2.00000E+01		
PYROPE	1.0791812	1.20000E+01	1.61259E+03	1.35792E+03
ALMANDINE	0.7781512	6.00000E+00	9.95734E+02	6.78960E+02
GROSSULAR	0.3010300	2.00000E+00	3.00346E+02	2.50760E+02

	mass, grams	volume, cc
created	0.000000E+00	0.000000E+00
destroyed	4.151346E-04	1.991680E-04
net	-4.151346E-04	-1.991680E-04

warning-- these volume totals may be incomplete because
of missing partial molar volume data in the data base

--- mineral saturation state summary ---

mineral	affinity, kcal	state	mineral	affinity, kcal	state
DIAMOND	0.0000		BRUCITE	-7.2209	
CALCITE	-4.1051		ARAGONITE	-2.3809	
MAGNESITE	-0.7878		DOLOMITE	-3.6567	
FORSTERITE	-9.8291		ENSTATITE-CL	-4.6060	
ENSTATITE-OR	-4.5110		ENSTATITE-PR	-5.7107	
DIOPSIDE	-8.5219		FERROSILITE	-9.5953	
COESITE	-3.5190		GRAPHITE	-0.3430	
PYRRHOTITE	-3.9970		PYRITE	-4.8369	
FERROUS_OXIDE	-6.7194		SEPIOLITE	1034.8824	ssatd
SIDERITE	-5.0450		QUARTZ-ALPHA	-4.1572	
QUARTZ-BETA	-5.2352		CRISTOBALITE-ALPHA	-8.9596	
CHALCEDONY	-5.1117		AMORPHOUS_SILICA	-6.4505	

--- summary of solid solutions ---

mineral	aff. kcal/mol	mole frac.	lambda	state
ORTHOPYROXENE(SS)	-4.2615			

```
    FERROSILITE                -4.26148    0.1014831     1.00000
    ENSTATITE-OR               -4.26148    0.8985169     1.00000

OLIVINE                  -9.7263
    FAYALITE                   -9.72632    0.0431403     1.00000
    FORSTERITE                 -9.72632    0.9568597     1.00000

BIOTITE                 -29.4262
    PHLOGOPITE                -29.42620    0.9992090     1.00000
    ANNITE                    -29.42620    0.0007910     1.00000

CLINOPYROXENE(SS)        -8.3055
    DIOPSIDE                   -8.30547    0.9113326     1.00000
    HEDENBERGITE               -8.30547    0.0884594     1.00000
    JADEITE                    -8.30547    0.0002079     1.00000

GARNET(SS)              -15.1674
    PYROPE                    -15.16742    0.5831522     1.00000
    ALMANDINE                 -15.16742    0.1916160     1.00000
    GROSSULAR                 -15.16742    0.2252318     1.00000

CALCITE(SS)               0.0000
    CALCITE                     0.00003    0.1718984     1.00000
    MAGNESITE                   0.00003    0.7132336     1.00000
    SIDERITE                    0.00003    0.1148680     1.00000


solid solution product phases

           xbar     lambda   activity  log xbar  log lambda log activity


          --- summary of gas species ---

          gas               log fugacity         fugacity         partial pressure

  CO2(G)                        9.10553       1.27505E+09
  O2(G)                        -9.60427       2.48730E-10
  S2(G)                         3.99701       9.93132E+03
  CH4(G)                        6.97348       9.40753E+06
  H2(G)                         3.80949       6.44893E+03
  H2S(G)                        7.27169       1.86935E+07
  H2O(G)                        6.90035       7.94972E+06



- - - - - - - - - - - - - - - - - - - - - - - - - - - - - - - -



stepping to zi=  3.1623E-06, delzi=  2.1623E-06, nord= 2
              ncycle=  0
steps completed =    10, iter =  9, ncorr = 0
most rapidly changing is zvclg1(AL+++             ) =    -18.3253
- - - - - - - - - - - - - - - - - - - - - - - - - - - - - - - -



                    reaction progress        =  3.16227766016836E-06
                    log of reaction progress =      -5.5000000

                    temperature    =    900.000 degrees c
                    total pressure =  50000.000 bars

                    computing units remaining =      0.000


step size is limited by the print requirement


                              --- reactant summary ---



        reactant            moles       delta moles      grams        delta grams
```

CLINOPYROXENE(SS)	2.00000E+01	3.16228E-06	4.19234E+03	6.62867E-04
GARNET(SS)	2.00000E+01	3.16228E-06	2.90866E+03	4.59900E-04
COESITE	2.00000E+01	3.16228E-06	1.20169E+03	1.90003E-04

current total mass = 8.30269E+03 grams
delta total mass = 1.31277E-03 grams
delta total volume = 0.00063 cc

reactant	affinity	rel. rate
CLINOPYROXENE(SS)	20.8317	1.00000E+00
GARNET(SS)	15.2845	1.00000E+00
COESITE	3.5190	1.00000E+00

affinity of the overall irreversible reaction= 39.635 kcal
contributions from irreversible reactions
with no thermodynamic data are not included

--- element totals for the aqueous phase ---

element	mg/kg soln.	molal conc.	moles
O	7.273299E+05	1.286051E+02	1.294611E+02
NA	2.421853E+03	2.980186E-01	3.000022E-01
K	2.333971E+04	1.688760E+00	1.700000E+00
CA	4.542477E+04	3.206235E+00	3.227575E+00
MG	4.631000E+02	5.390257E-02	5.426134E-02
AL	9.393150E-01	9.848607E-05	9.914160E-05
SI	8.876043E+03	8.940618E-01	9.000127E-01
H	4.331044E+04	1.215641E+02	1.223733E+02
C	1.451786E+05	3.419430E+01	3.442190E+01
CL	1.244921E+03	9.933880E-02	1.000000E-01
S	2.089767E+03	1.844016E-01	1.856290E-01
FE	3.199275E+02	1.620623E-02	1.631410E-02
co3--		0.000000E+00	0.000000E+00
so4--		0.000000E+00	0.000000E+00
s--		0.000000E+00	0.000000E+00

warning-- co3--, so4--, and s-- totals require that routine comp1
have the names of non-carbonate carbon, sulfide sulfur,
and non-sulfate sulfur aqueous species

single ion activities and activity coefficients are here defined
with respect to the internal ph scale

	ph	eh	pe
internal ph scale	4.7393	1.3458	5.7816E+00
modified nbs ph scale	4.4186	1.4204	6.1023E+00
rational ph scale	4.4186	1.4204	6.1023E+00

phcl = 6.1381

oxygen fugacity = 2.48730E-10
log oxygen fugacity = -9.60427

activity of water = 0.99667
log activity of water = -0.00145
alkalinity = 0.000000E+00 equiv/kg solvent
(not def. for t.gt.50 c)

ionic strength = 5.212527E+00 molal
sum of molalities = 36.8425325136912
osmotic coefficient = 0.00502

equiv. stoich. ionic strength = 9.933880E-02 molal

mass of solution = 2.847812 kg
mass of solvent = 1.006656 kg
mass of solutes = 1.841156 kg
conc of solutes = 64.651602 per cent (w/w)

species	moles	grams	conc	log conc	log g	log act
H2O	5.58781E+01	1.00666E+03				
NA+	2.60355E-01	5.98550E+00	2.58633E-01	-0.58732	-0.32065	-0.90797
K+	1.47005E+00	5.74765E+01	1.46033E+00	0.16445	-0.32065	-0.15620
CA++	1.55306E-04	6.22467E-03	1.54279E-04	-3.81169	-1.28261	-5.09430
MG++	1.21188E-05	2.94548E-04	1.20387E-05	-4.91942	-1.28261	-6.20203
AL+++	4.72841E-19	1.27580E-17	4.69715E-19	-18.32817	-2.88588	-21.21404
SIO2(AQ)	4.00363E-01	2.40555E+01	3.97716E-01	-0.40043	0.00000	-0.40043
H+	3.83914E-05	3.86947E-05	3.81375E-05	-4.41865	-0.32065	-4.73930
CO3--	2.23756E-01	1.34274E+01	2.22276E-01	-0.65311	-1.28261	-1.93572
CL-	8.40901E-02	2.98125E+00	8.35341E-02	-1.07814	-0.32065	-1.39879
SO4--	2.94742E-04	2.83122E-02	2.92793E-04	-3.53344	-1.28261	-4.81605
FE++	9.24565E-08	5.16342E-06	9.18451E-08	-7.03694	-1.28261	-8.31956
O2(AQ)	1.87676E-16	6.00542E-15	1.86435E-16	-15.72947	0.00000	-15.72947
H2(AQ)	1.31316E-02	2.64708E-02	1.30448E-02	-1.88456	0.00000	-1.88456
CH4(AQ)	5.08577E-02	8.15890E-01	5.05214E-02	-1.29652	0.00000	-1.29652
HS-	4.00429E-02	1.32414E+00	3.97782E-02	-1.40036	-0.32065	-1.72101
FE+++	1.52110E-14	8.49489E-13	1.51104E-14	-13.82072	-2.88588	-16.70660
HCO3-	5.04563E-01	3.07869E+01	5.01226E-01	-0.29997	-0.32065	-0.62062
CLO4-	1.64173E-42	1.63271E-40	1.63087E-42	-41.78758	-0.32065	-42.10823
OH-	5.38005E-01	9.15002E+00	5.34448E-01	-0.27209	-0.32065	-0.59275
HCOO-	2.54654E+00	1.14639E+02	2.52970E+00	0.40307	-0.32065	0.08242
CH3COO-	2.97125E-02	1.75436E+00	2.95160E-02	-1.52994	-0.32065	-1.85060
CH3CH2COO-	4.97538E-01	3.63558E+01	4.94248E-01	-0.30605	-0.32065	-0.62671
CO(AQ)	6.42903E-02	1.80080E+00	6.38652E-02	-1.19474	0.00000	-1.19474
ETHANE(AQ)	2.16344E-04	6.50533E-03	2.14913E-04	-3.66774	0.00000	-3.66774
ETHYLENE(AQ)	4.69415E-07	1.31688E-05	4.66311E-07	-6.33132	0.00000	-6.33132
PROPANE(AQ)	1.23547E-06	5.44795E-05	1.22730E-06	-5.91105	0.00000	-5.91105
HEXANE(AQ)	2.99017E-13	2.57682E-11	2.97040E-13	-12.52719	0.00000	-12.52719
BENZENE(AQ)	9.96424E-13	7.78341E-11	9.89836E-13	-12.00444	0.00000	-12.00444
TOLUENE(AQ)	4.01635E-15	3.70068E-13	3.98980E-15	-14.39905	0.00000	-14.39905
SI2O4(AQ)	2.15490E-02	2.58951E+00	2.14065E-02	-1.66945	0.00000	-1.66945
ALO2-	7.24018E-06	4.27028E-04	7.19231E-06	-5.14313	-0.32065	-5.46378
ALO2(SIO2)-	9.19014E-05	1.09422E-02	9.12938E-05	-4.03956	-0.32065	-4.36021
CACL+	2.32037E-04	1.75265E-02	2.30503E-04	-3.63732	-0.32065	-3.95798
CACL2(AQ)	3.94250E-07	4.37563E-05	3.91644E-07	-6.40711	0.00000	-6.40711
CACO3(AQ)	5.71481E-03	5.71991E-01	5.67702E-03	-2.24588	0.00000	-2.24588
CA(HCO3)+	3.16653E+00	3.20127E+02	3.14559E+00	0.49770	-0.24503	0.25267
CA(OH)+	4.15711E-04	2.37318E-02	4.12962E-04	-3.38409	-0.32065	-3.70474
CA(HSIO3)+	5.45303E-02	6.38940E+00	5.41697E-02	-1.26624	-0.32065	-1.58690
FECL+	5.36336E-08	4.89675E-06	5.32790E-08	-7.27344	-0.32065	-7.59410
FECL2(AQ)	4.40047E-05	5.57773E-03	4.37137E-05	-4.35938	0.00000	-4.35938
FE(HSIO3)+	1.62699E-02	2.16290E+00	1.61624E-02	-1.79149	-0.32065	-2.11215
KCL(AQ)	1.44968E-02	1.08076E+00	1.44010E-02	-1.84161	0.00000	-1.84161
KOH	2.15439E-01	1.20873E+01	2.14014E-01	-0.66956	0.00000	-0.66956
KSO4-	1.26999E-05	1.71646E-03	1.26159E-05	-4.89908	-0.32065	-5.21973
MGCL+	5.12491E-06	3.06255E-04	5.09103E-06	-5.29319	-0.32065	-5.61385
MG(HCO3)+	6.75562E-04	5.76404E-02	6.71095E-04	-3.17322	-0.32065	-3.49387
MG(HSIO3)+	5.35685E-02	5.43167E+00	5.32143E-02	-1.27397	-0.32065	-1.59462
MGSO4(AQ)	9.63159E-09	1.15928E-06	9.56791E-09	-8.01918	0.00000	-8.01918
NACL(AQ)	8.17158E-04	4.77570E-02	8.11755E-04	-3.09058	0.00000	-3.09058
NACO3-	5.43081E-04	4.50752E-02	5.39490E-04	-3.26802	-0.32065	-3.58867
NAHCO3(AQ)	7.04316E-03	5.91674E-01	6.99659E-03	-2.15511	0.00000	-2.15511
NAHSIO3(AQ)	2.29467E-04	2.29654E-02	2.27950E-04	-3.64216	0.00000	-3.64216
NAOH(AQ)	3.10145E-02	1.24049E+00	3.08095E-02	-1.51132	0.00000	-1.51132
HSIO3-	3.31862E-01	2.55838E+01	3.29667E-01	-0.48192	-0.32065	-0.80258
HSO4-	1.89242E-03	1.83689E-01	1.87991E-03	-2.72586	-0.32065	-3.04652
H2S(AQ)	1.43386E-01	4.88600E+00	1.42438E-01	-0.84637	0.00000	-0.84637
CO2(AQ)	2.62922E+01	1.15711E+03	2.61183E+01	1.41695	0.00000	1.41695
HCL(AQ)	2.69904E-04	9.84093E-03	2.68119E-04	-3.57167	0.00000	-3.57167
CH3CH2COOH	5.20604E-05	3.85659E-03	5.17162E-05	-4.28637	0.00000	-4.28637
CH3COOH	1.69982E-03	1.02078E-01	1.68858E-03	-2.77248	0.00000	-2.77248
HCOOH	3.15827E-03	1.45361E-01	3.13739E-03	-2.50343	0.00000	-2.50343

--- activity ratios of cations ---

log (NA+ /h+**0)	3.8313321
log (K+ /h+**0)	4.5830991
log (CA++ /h+**0)	4.3842969
log (MG++ /h+**0)	3.2765689
log (AL+++ /h+**0)	-6.9961412
log (FE++ /h+**0)	1.1590454
log (FE+++ /h+**0)	-2.4886985

--- grand summary of solid phases (e.s.+p.r.s.+reactants) ---

phase/end-member	log moles	moles	grams	volume, cc
COESITE	1.3010299	2.00000E+01	1.20169E+03	4.53760E+02
CLINOPYROXENE(SS)	1.3010299	2.00000E+01		
DIOPSIDE	0.6020599	4.00000E+00	8.66209E+02	2.64800E+02
HEDENBERGITE	0.3010299	2.00000E+00	4.96189E+02	1.32400E+02
JADEITE	1.1461280	1.40000E+01	2.82994E+03	8.44760E+02
GARNET(SS)	1.3010299	2.00000E+01		
PYROPE	1.0791812	1.20000E+01	1.61259E+03	1.35792E+03
ALMANDINE	0.7781512	6.00000E+00	9.95734E+02	6.78960E+02
GROSSULAR	0.3010299	2.00000E+00	3.00346E+02	2.50760E+02

	mass, grams	volume, cc
created	0.000000E+00	0.000000E+00
destroyed	1.312771E-03	6.298245E-04
net	-1.312771E-03	-6.298245E-04

warning-- these volume totals may be incomplete because
of missing partial molar volume data in the data base

--- mineral saturation state summary ---

mineral	affinity, kcal	state	mineral	affinity, kcal	state
DIAMOND	0.0000		BRUCITE	-7.2208	
CALCITE	-4.1051		ARAGONITE	-2.3809	
MAGNESITE	-0.7878		DOLOMITE	-3.6566	
FORSTERITE	-9.8290		ENSTATITE-CL	-4.6059	
ENSTATITE-OR	-4.5109		ENSTATITE-PR	-5.7107	
DIOPSIDE	-8.5218		FERROSILITE	-9.5952	
COESITE	-3.5190		GRAPHITE	-0.3430	
PYRRHOTITE	-3.9969		PYRITE	-4.8367	
FERROUS_OXIDE	-6.7193		SEPIOLITE	1034.8826	ssatd
SIDERITE	-5.0448		QUARTZ-ALPHA	-4.1572	
QUARTZ-BETA	-5.2352		CRISTOBALITE-ALPHA	-8.9596	
CHALCEDONY	-5.1117		AMORPHOUS_SILICA	-6.4505	

--- summary of solid solutions ---

mineral	aff. kcal/mol	mole frac.	lambda	state
ORTHOPYROXENE(SS)	-4.2614			
FERROSILITE	-4.26140	0.1014850	1.00000	
ENSTATITE-OR	-4.26140	0.8985150	1.00000	
OLIVINE	-9.7262			
FAYALITE	-9.72618	0.0431420	1.00000	
FORSTERITE	-9.72618	0.9568580	1.00000	
BIOTITE	-29.3555			
PHLOGOPITE	-29.35548	0.9992090	1.00000	
ANNITE	-29.35548	0.0007910	1.00000	

```
CLINOPYROXENE(SS)        -8.3054
    DIOPSIDE                  -8.30536    0.9113251     1.00000
    HEDENBERGITE              -8.30536    0.0884606     1.00000
    JADEITE                   -8.30536    0.0002143     1.00000

GARNET(SS)              -15.1204
    PYROPE                   -15.12039    0.5831535     1.00000
    ALMANDINE                -15.12039    0.1916204     1.00000
    GROSSULAR                -15.12039    0.2252260     1.00000

CALCITE(SS)               0.0001
    CALCITE                    0.00009    0.1718940     1.00000
    MAGNESITE                  0.00009    0.7132353     1.00000
    SIDERITE                   0.00009    0.1148707     1.00000


solid solution product phases

           xbar     lambda   activity  log xbar  log lambda log activity


          --- summary of gas species ---

          gas               log fugacity         fugacity         partial pressure

  CO2(G)                       9.10553       1.27505E+09
  O2(G)                       -9.60427       2.48730E-10
  S2(G)                        3.99701       9.93132E+03
  CH4(G)                       6.97348       9.40753E+06
  H2(G)                        3.80949       6.44893E+03
  H2S(G)                       7.27169       1.86935E+07
  H2O(G)                       6.90035       7.94972E+06



- - - - - - - - - - - - - - - - - - - - - - - - - - - - - - - -



stepping to zi=  1.0000E-05, delzi=  6.8377E-06, nord= 2
              ncycle=  0
steps completed =    11, iter = 10, ncorr = 0
most rapidly changing is zvclg1(AL+++             ) =    -18.2862
- - - - - - - - - - - - - - - - - - - - - - - - - - - - - - - -



                    reaction progress        =  9.99999999999993E-06
                    log of reaction progress =      -5.0000000

                    temperature    =    900.000 degrees c
                    total pressure =  50000.000 bars

                    computing units remaining =      0.000


step size is limited by the print requirement


                              --- reactant summary ---



        reactant            moles       delta moles      grams        delta grams

  CLINOPYROXENE(SS)       2.00000E+01    1.00000E-05    4.19234E+03    2.09617E-03
  GARNET(SS)              2.00000E+01    1.00000E-05    2.90866E+03    1.45433E-03
  COESITE                 2.00000E+01    1.00000E-05    1.20169E+03    6.00843E-04


               current total mass =  8.30269E+03 grams
               delta total mass   =  4.15135E-03 grams
               delta total volume =      0.00199 cc


        reactant           affinity     rel. rate
```

```
CLINOPYROXENE(SS)         20.6847    1.00000E+00
GARNET(SS)                15.1444    1.00000E+00
COESITE                    3.5189    1.00000E+00

       affinity of the overall irreversible reaction=    39.348 kcal
       contributions from irreversible reactions
       with no thermodynamic data are not included



   --- element totals for the aqueous phase ---

element         mg/kg soln.        molal conc.          moles

   O         7.273297E+05       1.286052E+02       1.294611E+02
   NA        2.421889E+03       2.980234E-01       3.000070E-01
   K         2.333969E+04       1.688760E+00       1.700000E+00
   CA        4.542476E+04       3.206238E+00       3.227578E+00
   MG        4.631463E+02       5.390801E-02       5.426682E-02
   AL        1.027809E+00       1.077647E-04       1.084819E-04
   SI        8.876304E+03       8.940891E-01       9.000400E-01
   H         4.331040E+04       1.215641E+02       1.223733E+02
   C         1.451784E+05       3.419430E+01       3.442190E+01
   CL        1.244919E+03       9.933881E-02       1.000000E-01
   S         2.089765E+03       1.844016E-01       1.856290E-01
   FE        3.199808E+02       1.620895E-02       1.631684E-02

   co3--                        0.000000E+00       0.000000E+00
   so4--                        0.000000E+00       0.000000E+00
   s--                          0.000000E+00       0.000000E+00

 warning-- co3--, so4--, and s-- totals require that routine comp1
 have the names of non-carbonate carbon, sulfide sulfur,
 and non-sulfate sulfur aqueous species


single ion activities and activity coefficients are here defined
with respect to the internal ph scale



                                  ph           eh           pe

  internal ph scale             4.7393       1.3458    5.7816E+00
  modified nbs ph scale         4.4186       1.4204    6.1023E+00
  rational ph scale             4.4186       1.4204    6.1023E+00

phcl =      6.1381



       oxygen fugacity =  2.48730E-10
       log oxygen fugacity =  -9.60427



                           activity of water =   0.99667
            log activity of water =  -0.00145
            alkalinity =  0.000000E+00 equiv/kg solvent
                                     (not def. for t.gt.50 c)

                 ionic strength =  5.212544E+00 molal
              sum of molalities =   36.8425701679749
            osmotic coefficient =    0.00502
                 equiv. stoich. ionic strength =  9.933881E-02 molal

                      mass of solution =   2.847815 kg
                      mass of solvent  =   1.006656 kg
                      mass of solutes  =   1.841159 kg
                      conc of solutes  =  64.651643 per cent (w/w)


species                moles        grams          conc        log conc      log g      log act

H2O                 5.58781E+01   1.00666E+03
```

NA+	2.60359E-01	5.98559E+00	2.58638E-01	-0.58731	-0.32065	-0.90796
K+	1.47005E+00	5.74765E+01	1.46033E+00	0.16445	-0.32065	-0.15620
CA++	1.55306E-04	6.22468E-03	1.54280E-04	-3.81169	-1.28261	-5.09430
MG++	1.21199E-05	2.94575E-04	1.20398E-05	-4.91938	-1.28261	-6.20199
AL+++	5.17383E-19	1.39598E-17	5.13962E-19	-18.28907	-2.88588	-21.17495
SIO2(AQ)	4.00368E-01	2.40558E+01	3.97720E-01	-0.40042	0.00000	-0.40042
H+	3.83914E-05	3.86946E-05	3.81375E-05	-4.41865	-0.32065	-4.73930
CO3--	2.23756E-01	1.34274E+01	2.22277E-01	-0.65311	-1.28261	-1.93572
CL-	8.40901E-02	2.98125E+00	8.35341E-02	-1.07814	-0.32065	-1.39879
SO4--	2.94743E-04	2.83123E-02	2.92794E-04	-3.53344	-1.28261	-4.81605
FE++	9.24709E-08	5.16422E-06	9.18595E-08	-7.03688	-1.28261	-8.31949
O2(AQ)	1.87676E-16	6.00542E-15	1.86435E-16	-15.72947	0.00000	-15.72947
H2(AQ)	1.31316E-02	2.64707E-02	1.30448E-02	-1.88456	0.00000	-1.88456
CH4(AQ)	5.08577E-02	8.15889E-01	5.05214E-02	-1.29652	0.00000	-1.29652
HS-	4.00430E-02	1.32414E+00	3.97782E-02	-1.40035	-0.32065	-1.72101
FE+++	1.52134E-14	8.49623E-13	1.51128E-14	-13.82065	-2.88588	-16.70653
HCO3-	5.04563E-01	3.07870E+01	5.01227E-01	-0.29997	-0.32065	-0.62062
CLO4-	1.64173E-42	1.63271E-40	1.63087E-42	-41.78758	-0.32065	-42.10823
OH-	5.38006E-01	9.15002E+00	5.34448E-01	-0.27209	-0.32065	-0.59275
HCOO-	2.54654E+00	1.14639E+02	2.52971E+00	0.40307	-0.32065	0.08242
CH3COO-	2.97125E-02	1.75436E+00	2.95160E-02	-1.52994	-0.32065	-1.85060
CH3CH2COO-	4.97538E-01	3.63557E+01	4.94248E-01	-0.30605	-0.32065	-0.62671
CO(AQ)	6.42903E-02	1.80080E+00	6.38652E-02	-1.19474	0.00000	-1.19474
ETHANE(AQ)	2.16344E-04	6.50532E-03	2.14913E-04	-3.66774	0.00000	-3.66774
ETHYLENE(AQ)	4.69414E-07	1.31688E-05	4.66311E-07	-6.33132	0.00000	-6.33132
PROPANE(AQ)	1.23547E-06	5.44795E-05	1.22730E-06	-5.91105	0.00000	-5.91105
HEXANE(AQ)	2.99016E-13	2.57682E-11	2.97039E-13	-12.52719	0.00000	-12.52719
BENZENE(AQ)	9.96423E-13	7.78340E-11	9.89835E-13	-12.00444	0.00000	-12.00444
TOLUENE(AQ)	4.01635E-15	3.70067E-13	3.98979E-15	-14.39905	0.00000	-14.39905
SI2O4(AQ)	2.15495E-02	2.58957E+00	2.14070E-02	-1.66944	0.00000	-1.66944
ALO2-	7.92221E-06	4.67254E-04	7.86983E-06	-5.10403	-0.32065	-5.42469
ALO2(SIO2)-	1.00560E-04	1.19731E-02	9.98948E-05	-4.00046	-0.32065	-4.32111
CACL+	2.32037E-04	1.75265E-02	2.30503E-04	-3.63732	-0.32065	-3.95798
CACL2(AQ)	3.94250E-07	4.37563E-05	3.91644E-07	-6.40711	0.00000	-6.40711
CACO3(AQ)	5.71481E-03	5.71991E-01	5.67703E-03	-2.24588	0.00000	-2.24588
CA(HCO3)+	3.16653E+00	3.20127E+02	3.14559E+00	0.49770	-0.24503	0.25267
CA(OH)+	4.15711E-04	2.37318E-02	4.12962E-04	-3.38409	-0.32065	-3.70474
CA(HSIO3)+	5.45310E-02	6.38948E+00	5.41704E-02	-1.26624	-0.32065	-1.58689
FECL+	5.36419E-08	4.89751E-06	5.32873E-08	-7.27338	-0.32065	-7.59403
FECL2(AQ)	4.40115E-05	5.57859E-03	4.37205E-05	-4.35931	0.00000	-4.35931
FE(HSIO3)+	1.62727E-02	2.16327E+00	1.61651E-02	-1.79142	-0.32065	-2.11208
KCL(AQ)	1.44968E-02	1.08076E+00	1.44010E-02	-1.84161	0.00000	-1.84161
KOH	2.15439E-01	1.20873E+01	2.14014E-01	-0.66956	0.00000	-0.66956
KSO4-	1.26999E-05	1.71646E-03	1.26159E-05	-4.89908	-0.32065	-5.21973
MGCL+	5.12537E-06	3.06282E-04	5.09148E-06	-5.29316	-0.32065	-5.61381
MG(HCO3)+	6.75622E-04	5.76455E-02	6.71155E-04	-3.17318	-0.32065	-3.49383
MG(HSIO3)+	5.35739E-02	5.43222E+00	5.32197E-02	-1.27393	-0.32065	-1.59458
MGSO4(AQ)	9.63245E-09	1.15939E-06	9.56877E-09	-8.01914	0.00000	-8.01914
NACL(AQ)	8.17170E-04	4.77577E-02	8.11767E-04	-3.09057	0.00000	-3.09057
NACO3-	5.43090E-04	4.50759E-02	5.39499E-04	-3.26801	-0.32065	-3.58866
NAHCO3(AQ)	7.04327E-03	5.91683E-01	6.99670E-03	-2.15511	0.00000	-2.15511
NAHSIO3(AQ)	2.29474E-04	2.29660E-02	2.27956E-04	-3.64215	0.00000	-3.64215
NAOH(AQ)	3.10150E-02	1.24051E+00	3.08100E-02	-1.51131	0.00000	-1.51131
HSIO3-	3.31866E-01	2.55841E+01	3.29672E-01	-0.48192	-0.32065	-0.80257
HSO4-	1.89242E-03	1.83689E-01	1.87991E-03	-2.72586	-0.32065	-3.04652
H2S(AQ)	1.43386E-01	4.88600E+00	1.42438E-01	-0.84637	0.00000	-0.84637
CO2(AQ)	2.62922E+01	1.15711E+03	2.61183E+01	1.41695	0.00000	1.41695
HCL(AQ)	2.69903E-04	9.84092E-03	2.68119E-04	-3.57167	0.00000	-3.57167
CH3CH2COOH	5.20604E-05	3.85659E-03	5.17161E-05	-4.28637	0.00000	-4.28637
CH3COOH	1.69982E-03	1.02078E-01	1.68858E-03	-2.77248	0.00000	-2.77248
HCOOH	3.15827E-03	1.45361E-01	3.13739E-03	-2.50343	0.00000	-2.50343

--- activity ratios of cations ---

log (NA+ /h+**0)	3.8313391
log (K+ /h+**0)	4.5830992
log (CA++ /h+**0)	4.3842972
log (MG++ /h+**0)	3.2766076
log (AL+++ /h+**0)	-6.9570447
log (FE++ /h+**0)	1.1591131
log (FE+++ /h+**0)	-2.4886308

--- grand summary of solid phases (e.s.+p.r.s.+reactants) ---

phase/end-member	log moles	moles	grams	volume, cc
COESITE	1.3010298	2.00000E+01	1.20169E+03	4.53760E+02
CLINOPYROXENE(SS)	1.3010298	2.00000E+01		
DIOPSIDE	0.6020598	4.00000E+00	8.66209E+02	2.64800E+02
HEDENBERGITE	0.3010298	2.00000E+00	4.96189E+02	1.32400E+02
JADEITE	1.1461278	1.40000E+01	2.82994E+03	8.44760E+02
GARNET(SS)	1.3010298	2.00000E+01		
PYROPE	1.0791810	1.20000E+01	1.61258E+03	1.35792E+03
ALMANDINE	0.7781510	6.00000E+00	9.95733E+02	6.78960E+02
GROSSULAR	0.3010298	2.00000E+00	3.00346E+02	2.50760E+02

	mass, grams	volume, cc
created	0.000000E+00	0.000000E+00
destroyed	4.151346E-03	1.991680E-03
net	-4.151346E-03	-1.991680E-03

warning-- these volume totals may be incomplete because
of missing partial molar volume data in the data base

--- mineral saturation state summary ---

mineral	affinity, kcal	state	mineral	affinity, kcal	state
DIAMOND	0.0000		BRUCITE	-7.2206	
CALCITE	-4.1051		ARAGONITE	-2.3809	
MAGNESITE	-0.7876		DOLOMITE	-3.6564	
FORSTERITE	-9.8285		ENSTATITE-CL	-4.6057	
ENSTATITE-OR	-4.5106		ENSTATITE-PR	-5.7104	
DIOPSIDE	-8.5216		FERROSILITE	-9.5948	
COESITE	-3.5189		GRAPHITE	-0.3430	
PYRRHOTITE	-3.9966		PYRITE	-4.8364	
FERROUS_OXIDE	-6.7190		SEPIOLITE	1034.8831	ssatd
SIDERITE	-5.0445		QUARTZ-ALPHA	-4.1572	
QUARTZ-BETA	-5.2351		CRISTOBALITE-ALPHA	-8.9595	
CHALCEDONY	-5.1117		AMORPHOUS_SILICA	-6.4505	

--- summary of solid solutions ---

mineral	aff. kcal/mol		mole frac.	lambda	state
ORTHOPYROXENE(SS)	-4.2611				
FERROSILITE		-4.26115	0.1014911	1.00000	
ENSTATITE-OR		-4.26115	0.8985089	1.00000	
OLIVINE	-9.7257				
FAYALITE		-9.72572	0.0431475	1.00000	
FORSTERITE		-9.72572	0.9568525	1.00000	
BIOTITE	-29.1449				
PHLOGOPITE		-29.14489	0.9992088	1.00000	
ANNITE		-29.14489	0.0007912	1.00000	
CLINOPYROXENE(SS)	-8.3050				
DIOPSIDE		-8.30504	0.9113014	1.00000	
HEDENBERGITE		-8.30504	0.0884642	1.00000	
JADEITE		-8.30504	0.0002345	1.00000	
GARNET(SS)	-14.9804				
PYROPE		-14.98040	0.5831577	1.00000	
ALMANDINE		-14.98040	0.1916346	1.00000	
GROSSULAR		-14.98040	0.2252077	1.00000	
CALCITE(SS)	0.0003				

```
    CALCITE                     0.00028    0.1718801     1.00000
    MAGNESITE                   0.00028    0.7132406     1.00000
    SIDERITE                    0.00028    0.1148792     1.00000


solid solution product phases

           xbar     lambda   activity  log xbar  log lambda log activity


          --- summary of gas species ---

          gas               log fugacity         fugacity         partial pressure

  CO2(G)                         9.10553       1.27505E+09
  O2(G)                         -9.60427       2.48730E-10
  S2(G)                          3.99701       9.93133E+03
  CH4(G)                         6.97348       9.40753E+06
  H2(G)                          3.80949       6.44893E+03
  H2S(G)                         7.27169       1.86935E+07
  H2O(G)                         6.90035       7.94972E+06



- - - - - - - - - - - - - - - - - - - - - - - - - - - - - - - -



stepping to zi=  3.1623E-05, delzi=  2.1623E-05, nord= 2
              ncycle=  0
steps completed =    12, iter = 11, ncorr = 0
most rapidly changing is zvclg1(AL+++            ) =    -18.1816
- - - - - - - - - - - - - - - - - - - - - - - - - - - - - - - -



                    reaction progress        =  3.16227766016836E-05
                    log of reaction progress =      -4.5000000

                    temperature    =    900.000 degrees c
                    total pressure =  50000.000 bars

                    computing units remaining =      0.000


step size is limited by the print requirement


                              --- reactant summary ---



        reactant            moles       delta moles      grams        delta grams

  CLINOPYROXENE(SS)       2.00000E+01    3.16228E-05    4.19233E+03    6.62867E-03
  GARNET(SS)              2.00000E+01    3.16228E-05    2.90866E+03    4.59900E-03
  COESITE                 2.00000E+01    3.16228E-05    1.20168E+03    1.90003E-03


               current total mass =  8.30268E+03 grams
               delta total mass   =  1.31277E-02 grams
               delta total volume =      0.00630 cc


        reactant           affinity     rel. rate

  CLINOPYROXENE(SS)         20.2912    1.00000E+00
  GARNET(SS)                14.7698    1.00000E+00
  COESITE                    3.5188    1.00000E+00

         affinity of the overall irreversible reaction=    38.580 kcal
         contributions from irreversible reactions
         with no thermodynamic data are not included
```

--- element totals for the aqueous phase ---

element	mg/kg soln.	molal conc.	moles
O	7.273288E+05	1.286055E+02	1.294614E+02
NA	2.422003E+03	2.980386E-01	3.000221E-01
K	2.333961E+04	1.688761E+00	1.700000E+00
CA	4.542474E+04	3.206248E+00	3.227587E+00
MG	4.632926E+02	5.392523E-02	5.428413E-02
AL	1.307649E+00	1.371061E-04	1.380186E-04
SI	8.877129E+03	8.941754E-01	9.001265E-01
H	4.331026E+04	1.215642E+02	1.223733E+02
C	1.451780E+05	3.419432E+01	3.442190E+01
CL	1.244915E+03	9.933886E-02	1.000000E-01
S	2.089758E+03	1.844017E-01	1.856290E-01
FE	3.201496E+02	1.621756E-02	1.632549E-02
co3--		0.000000E+00	0.000000E+00
so4--		0.000000E+00	0.000000E+00
s--		0.000000E+00	0.000000E+00

warning-- co3--, so4--, and s-- totals require that routine comp1
have the names of non-carbonate carbon, sulfide sulfur,
and non-sulfate sulfur aqueous species

single ion activities and activity coefficients are here defined
with respect to the internal ph scale

	ph	eh	pe
internal ph scale	4.7393	1.3458	5.7816E+00
modified nbs ph scale	4.4186	1.4204	6.1023E+00
rational ph scale	4.4186	1.4204	6.1023E+00

phcl = 6.1381

oxygen fugacity = 2.48730E-10
log oxygen fugacity = -9.60427

activity of water = 0.99667
log activity of water = -0.00145
alkalinity = 0.000000E+00 equiv/kg solvent
(not def. for t.gt.50 c)

ionic strength = 5.212594E+00 molal
sum of molalities = 36.8426892416430
osmotic coefficient = 0.00502
equiv. stoich. ionic strength = 9.933886E-02 molal

mass of solution = 2.847824 kg
mass of solvent = 1.006655 kg
mass of solutes = 1.841169 kg
conc of solutes = 64.651770 per cent (w/w)

species	moles	grams	conc	log conc	log g	log act
H2O	5.58781E+01	1.00666E+03				
NA+	2.60372E-01	5.98589E+00	2.58651E-01	-0.58729	-0.32065	-0.90794
K+	1.47005E+00	5.74765E+01	1.46033E+00	0.16445	-0.32065	-0.15620
CA++	1.55307E-04	6.22471E-03	1.54280E-04	-3.81169	-1.28261	-5.09430
MG++	1.21234E-05	2.94658E-04	1.20432E-05	-4.91926	-1.28261	-6.20187
AL+++	6.58232E-19	1.77601E-17	6.53880E-19	-18.18450	-2.88588	-21.07038
SIO2(AQ)	4.00383E-01	2.40567E+01	3.97735E-01	-0.40041	0.00000	-0.40041
H+	3.83913E-05	3.86946E-05	3.81375E-05	-4.41865	-0.32065	-4.73930
CO3--	2.23757E-01	1.34275E+01	2.22278E-01	-0.65310	-1.28261	-1.93572
CL-	8.40900E-02	2.98124E+00	8.35341E-02	-1.07814	-0.32065	-1.39879
SO4--	2.94744E-04	2.83124E-02	2.92796E-04	-3.53344	-1.28261	-4.81605
FE++	9.25167E-08	5.16678E-06	9.19050E-08	-7.03666	-1.28261	-8.31927

O2(AQ)	1.87676E-16	6.00542E-15	1.86436E-16	-15.72947	0.00000	-15.72947
H2(AQ)	1.31316E-02	2.64707E-02	1.30448E-02	-1.88456	0.00000	-1.88456
CH4(AQ)	5.08576E-02	8.15888E-01	5.05214E-02	-1.29653	0.00000	-1.29653
HS-	4.00430E-02	1.32414E+00	3.97783E-02	-1.40035	-0.32065	-1.72101
FE+++	1.52210E-14	8.50046E-13	1.51203E-14	-13.82044	-2.88588	-16.70632
HCO3-	5.04563E-01	3.07870E+01	5.01227E-01	-0.29997	-0.32065	-0.62062
CLO4-	1.64173E-42	1.63271E-40	1.63088E-42	-41.78758	-0.32065	-42.10823
OH-	5.38006E-01	9.15003E+00	5.34449E-01	-0.27209	-0.32065	-0.59275
HCOO-	2.54654E+00	1.14640E+02	2.52971E+00	0.40307	-0.32065	0.08242
CH3COO-	2.97125E-02	1.75436E+00	2.95160E-02	-1.52994	-0.32065	-1.85060
CH3CH2COO-	4.97538E-01	3.63557E+01	4.94248E-01	-0.30605	-0.32065	-0.62671
CO(AQ)	6.42902E-02	1.80079E+00	6.38652E-02	-1.19474	0.00000	-1.19474
ETHANE(AQ)	2.16343E-04	6.50531E-03	2.14913E-04	-3.66774	0.00000	-3.66774
ETHYLENE(AQ)	4.69413E-07	1.31687E-05	4.66310E-07	-6.33133	0.00000	-6.33133
PROPANE(AQ)	1.23547E-06	5.44793E-05	1.22730E-06	-5.91105	0.00000	-5.91105
HEXANE(AQ)	2.99015E-13	2.57681E-11	2.97038E-13	-12.52719	0.00000	-12.52719
BENZENE(AQ)	9.96419E-13	7.78337E-11	9.89831E-13	-12.00444	0.00000	-12.00444
TOLUENE(AQ)	4.01633E-15	3.70065E-13	3.98978E-15	-14.39905	0.00000	-14.39905
SI2O4(AQ)	2.15511E-02	2.58977E+00	2.14086E-02	-1.66941	0.00000	-1.66941
ALO2-	1.00789E-05	5.94454E-04	1.00122E-05	-4.99947	-0.32065	-5.32012
ALO2(SIO2)-	1.27940E-04	1.52331E-02	1.27094E-04	-3.89588	-0.32065	-4.21653
CACL+	2.32038E-04	1.75265E-02	2.30503E-04	-3.63732	-0.32065	-3.95798
CACL2(AQ)	3.94250E-07	4.37562E-05	3.91643E-07	-6.40711	0.00000	-6.40711
CACO3(AQ)	5.71483E-03	5.71992E-01	5.67704E-03	-2.24588	0.00000	-2.24588
CA(HCO3)+	3.16654E+00	3.20128E+02	3.14560E+00	0.49770	-0.24503	0.25267
CA(OH)+	4.15712E-04	2.37319E-02	4.12963E-04	-3.38409	-0.32065	-3.70474
CA(HSIO3)+	5.45332E-02	6.38974E+00	5.41726E-02	-1.26622	-0.32065	-1.58687
FECL+	5.36683E-08	4.89991E-06	5.33134E-08	-7.27316	-0.32065	-7.59382
FECL2(AQ)	4.40330E-05	5.58132E-03	4.37419E-05	-4.35910	0.00000	-4.35910
FE(HSIO3)+	1.62813E-02	2.16441E+00	1.61737E-02	-1.79119	-0.32065	-2.11184
KCL(AQ)	1.44968E-02	1.08075E+00	1.44009E-02	-1.84161	0.00000	-1.84161
KOH	2.15439E-01	1.20873E+01	2.14014E-01	-0.66956	0.00000	-0.66956
KSO4-	1.26999E-05	1.71647E-03	1.26160E-05	-4.89908	-0.32065	-5.21973
MGCL+	5.12680E-06	3.06367E-04	5.09291E-06	-5.29303	-0.32065	-5.61369
MG(HCO3)+	6.75813E-04	5.76618E-02	6.71345E-04	-3.17305	-0.32065	-3.49371
MG(HSIO3)+	5.35911E-02	5.43395E+00	5.32367E-02	-1.27379	-0.32065	-1.59444
MGSO4(AQ)	9.63518E-09	1.15971E-06	9.57147E-09	-8.01902	0.00000	-8.01902
NACL(AQ)	8.17210E-04	4.77600E-02	8.11807E-04	-3.09055	0.00000	-3.09055
NACO3-	5.43118E-04	4.50782E-02	5.39527E-04	-3.26799	-0.32065	-3.58864
NAHCO3(AQ)	7.04363E-03	5.91713E-01	6.99706E-03	-2.15508	0.00000	-2.15508
NAHSIO3(AQ)	2.29494E-04	2.29680E-02	2.27976E-04	-3.64211	0.00000	-3.64211
NAOH(AQ)	3.10166E-02	1.24057E+00	3.08115E-02	-1.51129	0.00000	-1.51129
HSIO3-	3.31879E-01	2.55851E+01	3.29685E-01	-0.48190	-0.32065	-0.80255
HSO4-	1.89243E-03	1.83689E-01	1.87991E-03	-2.72586	-0.32065	-3.04652
H2S(AQ)	1.43386E-01	4.88600E+00	1.42438E-01	-0.84637	0.00000	-0.84637
CO2(AQ)	2.62922E+01	1.15711E+03	2.61184E+01	1.41695	0.00000	1.41695
HCL(AQ)	2.69903E-04	9.84090E-03	2.68118E-04	-3.57167	0.00000	-3.57167
CH3CH2COOH	5.20603E-05	3.85658E-03	5.17161E-05	-4.28637	0.00000	-4.28637
CH3COOH	1.69981E-03	1.02078E-01	1.68858E-03	-2.77248	0.00000	-2.77248
HCOOH	3.15827E-03	1.45361E-01	3.13739E-03	-2.50343	0.00000	-2.50343

--- activity ratios of cations ---

log (NA+ /h+**0)	3.8313611
log (K+ /h+**0)	4.5830993
log (CA++ /h+**0)	4.3842984
log (MG++ /h+**0)	3.2767301
log (AL+++ /h+**0)	-6.8524801
log (FE++ /h+**0)	1.1593271
log (FE+++ /h+**0)	-2.4884167

--- grand summary of solid phases (e.s.+p.r.s.+reactants) ---

phase/end-member	log moles	moles	grams	volume, cc
COESITE	1.3010293	2.00000E+01	1.20168E+03	4.53759E+02
CLINOPYROXENE(SS)	1.3010293	2.00000E+01		
DIOPSIDE	0.6020593	3.99999E+00	8.66208E+02	2.64800E+02
HEDENBERGITE	0.3010293	2.00000E+00	4.96188E+02	1.32400E+02
JADEITE	1.1461273	1.40000E+01	2.82994E+03	8.44759E+02

GARNET(SS)	1.3010293	2.00000E+01		
PYROPE	1.0791806	1.20000E+01	1.61258E+03	1.35792E+03
ALMANDINE	0.7781506	5.99999E+00	9.95732E+02	6.78959E+02
GROSSULAR	0.3010293	2.00000E+00	3.00345E+02	2.50760E+02

	mass, grams	volume, cc
created	0.000000E+00	0.000000E+00
destroyed	1.312771E-02	6.298245E-03
net	-1.312771E-02	-6.298245E-03

warning-- these volume totals may be incomplete because
of missing partial molar volume data in the data base

--- mineral saturation state summary ---

mineral	affinity, kcal	state	mineral	affinity, kcal	state
DIAMOND	0.0000		BRUCITE	-7.2200	
CALCITE	-4.1051		ARAGONITE	-2.3809	
MAGNESITE	-0.7869		DOLOMITE	-3.6558	
FORSTERITE	-9.8271		ENSTATITE-CL	-4.6049	
ENSTATITE-OR	-4.5099		ENSTATITE-PR	-5.7097	
DIOPSIDE	-8.5207		FERROSILITE	-9.5935	
COESITE	-3.5188		GRAPHITE	-0.3430	
PYRRHOTITE	-3.9954		PYRITE	-4.8352	
FERROUS_OXIDE	-6.7178		SEPIOLITE	1034.8847	ssatd
SIDERITE	-5.0433		QUARTZ-ALPHA	-4.1571	
QUARTZ-BETA	-5.2350		CRISTOBALITE-ALPHA	-8.9595	
CHALCEDONY	-5.1116		AMORPHOUS_SILICA	-6.4504	

--- summary of solid solutions ---

mineral	aff. kcal/mol		mole frac.	lambda	state
ORTHOPYROXENE(SS)	-4.2604				
FERROSILITE		-4.26035	0.1015103	1.00000	
ENSTATITE-OR		-4.26035	0.8984897	1.00000	
OLIVINE	-9.7243				
FAYALITE		-9.72427	0.0431650	1.00000	
FORSTERITE		-9.72427	0.9568350	1.00000	
BIOTITE	-28.5813				
PHLOGOPITE		-28.58134	0.9992083	1.00000	
ANNITE		-28.58134	0.0007917	1.00000	
CLINOPYROXENE(SS)	-8.3040				
DIOPSIDE		-8.30401	0.9112263	1.00000	
HEDENBERGITE		-8.30401	0.0884756	1.00000	
JADEITE		-8.30401	0.0002982	1.00000	
GARNET(SS)	-14.6059				
PYROPE		-14.60587	0.5831708	1.00000	
ALMANDINE		-14.60587	0.1916794	1.00000	
GROSSULAR		-14.60587	0.2251498	1.00000	
CALCITE(SS)	0.0009				supersatd., ignored
CALCITE		0.00088	0.1718362	1.00000	
MAGNESITE		0.00088	0.7132576	1.00000	
SIDERITE		0.00088	0.1149062	1.00000	

solid solution product phases

xbar	lambda	activity	log xbar	log lambda	log activity

--- summary of gas species ---

gas	log fugacity	fugacity	partial pressure
CO2(G)	9.10553	1.27505E+09	
O2(G)	-9.60427	2.48730E-10	
S2(G)	3.99701	9.93133E+03	
CH4(G)	6.97347	9.40752E+06	
H2(G)	3.80949	6.44893E+03	
H2S(G)	7.27169	1.86935E+07	
H2O(G)	6.90035	7.94972E+06	

- -

```
stepping to zi=  8.5680E-05, delzi=  5.4057E-05, nord= 3
              ncycle=  0
steps completed =    13, iter = 12, ncorr = 0
most rapidly changing is zvclg1(AL+++            ) =    -17.9955

stepping to zi=  1.0000E-04, delzi=  1.4320E-05, nord= 3
              ncycle=  0
steps completed =    14, iter = 11, ncorr = 0
most rapidly changing is zvclg1(AL+++            ) =    -17.9572
- - - - - - - - - - - - - - - - - - - - - - - - - - - - - - - -
```

reaction progress   = 9.99999999999994E-05
log of reaction progress =   -4.0000000

temperature   =   900.000 degrees c
total pressure = 50000.000 bars

computing units remaining =   0.000

step size is limited by the print requirement

--- reactant summary ---

reactant	moles	delta moles	grams	delta grams
CLINOPYROXENE(SS)	1.99999E+01	1.00000E-04	4.19232E+03	2.09617E-02
GARNET(SS)	1.99999E+01	1.00000E-04	2.90865E+03	1.45433E-02
COESITE	1.99999E+01	1.00000E-04	1.20168E+03	6.00843E-03

current total mass = 8.30265E+03 grams
delta total mass   = 4.15135E-02 grams
delta total volume =   0.01992 cc

reactant	affinity	rel. rate
CLINOPYROXENE(SS)	19.4463	1.00000E+00
GARNET(SS)	13.9648	1.00000E+00
COESITE	3.5186	1.00000E+00

affinity of the overall irreversible reaction=   36.930 kcal
contributions from irreversible reactions
with no thermodynamic data are not included

--- element totals for the aqueous phase ---

element	mg/kg soln.	molal conc.	moles
O	7.273262E+05	1.286065E+02	1.294622E+02
NA	2.422366E+03	2.980865E-01	3.000700E-01

K	2.333938E+04	1.688763E+00	1.700000E+00
CA	4.542467E+04	3.206280E+00	3.227614E+00
MG	4.637551E+02	5.397969E-02	5.433887E-02
AL	2.192571E+00	2.298922E-04	2.314219E-04
SI	8.879738E+03	8.944484E-01	9.004000E-01
H	4.330983E+04	1.215644E+02	1.223733E+02
C	1.451765E+05	3.419437E+01	3.442190E+01
CL	1.244903E+03	9.933900E-02	1.000000E-01
S	2.089738E+03	1.844020E-01	1.856290E-01
FE	3.206831E+02	1.624477E-02	1.635286E-02
co3--		0.000000E+00	0.000000E+00
so4--		0.000000E+00	0.000000E+00
s--		0.000000E+00	0.000000E+00

warning-- co3--, so4--, and s-- totals require that routine comp1
have the names of non-carbonate carbon, sulfide sulfur,
and non-sulfate sulfur aqueous species

single ion activities and activity coefficients are here defined
with respect to the internal ph scale

	ph	eh	pe
internal ph scale	4.7393	1.3458	5.7816E+00
modified nbs ph scale	4.4186	1.4204	6.1023E+00
rational ph scale	4.4186	1.4204	6.1023E+00

phcl = 6.1381

oxygen fugacity = 2.48731E-10
log oxygen fugacity = -9.60427

activity of water = 0.99667
log activity of water = -0.00145
alkalinity = 0.000000E+00 equiv/kg solvent
(not def. for t.gt.50 c)

ionic strength = 5.212755E+00 molal
sum of molalities = 36.8430657864566
osmotic coefficient = 0.00502
equiv. stoich. ionic strength = 9.933900E-02 molal

mass of solution = 2.847853 kg
mass of solvent = 1.006654 kg
mass of solutes = 1.841199 kg
conc of solutes = 64.652174 per cent (w/w)

species	moles	grams	conc	log conc	log g	log act
H2O	5.58780E+01	1.00665E+03				
NA+	2.60414E-01	5.98685E+00	2.58692E-01	-0.58722	-0.32065	-0.90787
K+	1.47005E+00	5.74765E+01	1.46034E+00	0.16445	-0.32065	-0.15620
CA++	1.55309E-04	6.22479E-03	1.54283E-04	-3.81168	-1.28262	-5.09430
MG++	1.21342E-05	2.94922E-04	1.20540E-05	-4.91887	-1.28262	-6.20149
AL+++	1.10358E-18	2.97763E-17	1.09628E-18	-17.96008	-2.88589	-20.84597
SIO2(AQ)	4.00430E-01	2.40596E+01	3.97783E-01	-0.40035	0.00000	-0.40035
H+	3.83913E-05	3.86946E-05	3.81375E-05	-4.41865	-0.32065	-4.73930
CO3--	2.23760E-01	1.34277E+01	2.22281E-01	-0.65310	-1.28262	-1.93572
CL-	8.40898E-02	2.98124E+00	8.35340E-02	-1.07814	-0.32065	-1.39879
SO4--	2.94750E-04	2.83129E-02	2.92801E-04	-3.53343	-1.28262	-4.81605
FE++	9.26613E-08	5.17485E-06	9.20488E-08	-7.03598	-1.28262	-8.31860
O2(AQ)	1.87676E-16	6.00542E-15	1.86436E-16	-15.72947	0.00000	-15.72947
H2(AQ)	1.31316E-02	2.64707E-02	1.30448E-02	-1.88456	0.00000	-1.88456
CH4(AQ)	5.08574E-02	8.15884E-01	5.05212E-02	-1.29653	0.00000	-1.29653
HS-	4.00432E-02	1.32414E+00	3.97785E-02	-1.40035	-0.32065	-1.72101
FE+++	1.52449E-14	8.51385E-13	1.51442E-14	-13.81975	-2.88589	-16.70565
HCO3-	5.04565E-01	3.07871E+01	5.01230E-01	-0.29996	-0.32065	-0.62062

CLO4-	1.64173E-42	1.63271E-40	1.63088E-42	-41.78758	-0.32065	-42.10823
OH-	5.38008E-01	9.15007E+00	5.34452E-01	-0.27209	-0.32065	-0.59275
HCOO-	2.54655E+00	1.14640E+02	2.52972E+00	0.40307	-0.32065	0.08242
CH3COO-	2.97125E-02	1.75436E+00	2.95161E-02	-1.52994	-0.32065	-1.85060
CH3CH2COO-	4.97537E-01	3.63557E+01	4.94248E-01	-0.30605	-0.32065	-0.62671
CO(AQ)	6.42901E-02	1.80079E+00	6.38651E-02	-1.19474	0.00000	-1.19474
ETHANE(AQ)	2.16342E-04	6.50526E-03	2.14912E-04	-3.66774	0.00000	-3.66774
ETHYLENE(AQ)	4.69411E-07	1.31687E-05	4.66308E-07	-6.33133	0.00000	-6.33133
PROPANE(AQ)	1.23545E-06	5.44788E-05	1.22729E-06	-5.91105	0.00000	-5.91105
HEXANE(AQ)	2.99010E-13	2.57676E-11	2.97033E-13	-12.52719	0.00000	-12.52719
BENZENE(AQ)	9.96406E-13	7.78327E-11	9.89820E-13	-12.00444	0.00000	-12.00444
TOLUENE(AQ)	4.01627E-15	3.70060E-13	3.98972E-15	-14.39906	0.00000	-14.39906
SI2O4(AQ)	2.15562E-02	2.59038E+00	2.14137E-02	-1.66931	0.00000	-1.66931
ALO2-	1.68978E-05	9.96637E-04	1.67861E-05	-4.77505	-0.32065	-5.09571
ALO2(SIO2)-	2.14524E-04	2.55422E-02	2.13106E-04	-3.67140	-0.32065	-3.99206
CACL+	2.32038E-04	1.75265E-02	2.30504E-04	-3.63732	-0.32065	-3.95798
CACL2(AQ)	3.94248E-07	4.37560E-05	3.91642E-07	-6.40711	0.00000	-6.40711
CACO3(AQ)	5.71487E-03	5.71996E-01	5.67709E-03	-2.24587	0.00000	-2.24587
CA(HCO3)+	3.16656E+00	3.20130E+02	3.14562E+00	0.49771	-0.24504	0.25267
CA(OH)+	4.15715E-04	2.37320E-02	4.12967E-04	-3.38408	-0.32065	-3.70474
CA(HSIO3)+	5.45401E-02	6.39055E+00	5.41796E-02	-1.26616	-0.32065	-1.58682
FECL+	5.37515E-08	4.90751E-06	5.33962E-08	-7.27249	-0.32065	-7.59314
FECL2(AQ)	4.41011E-05	5.58994E-03	4.38095E-05	-4.35843	0.00000	-4.35843
FE(HSIO3)+	1.63086E-02	2.16804E+00	1.62008E-02	-1.79046	-0.32065	-2.11112
KCL(AQ)	1.44967E-02	1.08075E+00	1.44009E-02	-1.84161	0.00000	-1.84161
KOH	2.15439E-01	1.20873E+01	2.14014E-01	-0.66956	0.00000	-0.66956
KSO4-	1.27000E-05	1.71648E-03	1.26161E-05	-4.89908	-0.32065	-5.21973
MGCL+	5.13134E-06	3.06638E-04	5.09742E-06	-5.29265	-0.32065	-5.61330
MG(HCO3)+	6.76415E-04	5.77131E-02	6.71943E-04	-3.17267	-0.32065	-3.49332
MG(HSIO3)+	5.36452E-02	5.43944E+00	5.32906E-02	-1.27335	-0.32065	-1.59400
MGSO4(AQ)	9.64378E-09	1.16075E-06	9.58004E-09	-8.01863	0.00000	-8.01863
NACL(AQ)	8.17335E-04	4.77674E-02	8.11933E-04	-3.09048	0.00000	-3.09048
NACO3-	5.43207E-04	4.50856E-02	5.39617E-04	-3.26791	-0.32065	-3.58857
NAHCO3(AQ)	7.04475E-03	5.91807E-01	6.99818E-03	-2.15501	0.00000	-2.15501
NAHSIO3(AQ)	2.29558E-04	2.29745E-02	2.28040E-04	-3.64199	0.00000	-3.64199
NAOH(AQ)	3.10215E-02	1.24077E+00	3.08165E-02	-1.51122	0.00000	-1.51122
HSIO3-	3.31920E-01	2.55882E+01	3.29726E-01	-0.48185	-0.32065	-0.80250
HSO4-	1.89244E-03	1.83691E-01	1.87993E-03	-2.72586	-0.32065	-3.04651
H2S(AQ)	1.43386E-01	4.88599E+00	1.42438E-01	-0.84637	0.00000	-0.84637
CO2(AQ)	2.62921E+01	1.15711E+03	2.61184E+01	1.41695	0.00000	1.41695
HCL(AQ)	2.69901E-04	9.84083E-03	2.68117E-04	-3.57168	0.00000	-3.57168
CH3CH2COOH	5.20599E-05	3.85656E-03	5.17158E-05	-4.28638	0.00000	-4.28638
CH3COOH	1.69981E-03	1.02077E-01	1.68857E-03	-2.77248	0.00000	-2.77248
HCOOH	3.15826E-03	1.45361E-01	3.13739E-03	-2.50343	0.00000	-2.50343

--- activity ratios of cations ---

log (NA+ /h+**0)	3.8314308
log (K+ /h+**0)	4.5830998
log (CA++ /h+**0)	4.3843020
log (MG++ /h+**0)	3.2771169
log (AL+++ /h+**0)	-6.6280630
log (FE++ /h+**0)	1.1600033
log (FE+++ /h+**0)	-2.4877404

--- grand summary of solid phases (e.s.+p.r.s.+reactants) ---

phase/end-member	log moles	moles	grams	volume, cc
COESITE	1.3010278	1.99999E+01	1.20168E+03	4.53758E+02
CLINOPYROXENE(SS)	1.3010278	1.99999E+01		
DIOPSIDE	0.6020578	3.99998E+00	8.66205E+02	2.64799E+02
HEDENBERGITE	0.3010278	1.99999E+00	4.96186E+02	1.32399E+02
JADEITE	1.1461259	1.39999E+01	2.82993E+03	8.44756E+02
GARNET(SS)	1.3010278	1.99999E+01		
PYROPE	1.0791791	1.19999E+01	1.61258E+03	1.35791E+03
ALMANDINE	0.7781491	5.99997E+00	9.95729E+02	6.78957E+02
GROSSULAR	0.3010278	1.99999E+00	3.00344E+02	2.50759E+02

	mass, grams	volume, cc
created	0.000000E+00	0.000000E+00
destroyed	4.151346E-02	1.991680E-02
net	-4.151346E-02	-1.991680E-02

warning-- these volume totals may be incomplete because
of missing partial molar volume data in the data base

--- mineral saturation state summary ---

mineral	affinity, kcal	state	mineral	affinity, kcal	state
DIAMOND	0.0000		BRUCITE	-7.2179	
CALCITE	-4.1051		ARAGONITE	-2.3809	
MAGNESITE	-0.7848		DOLOMITE	-3.6537	
FORSTERITE	-9.8227		ENSTATITE-CL	-4.6026	
ENSTATITE-OR	-4.5075		ENSTATITE-PR	-5.7073	
DIOPSIDE	-8.5181		FERROSILITE	-9.5896	
COESITE	-3.5186		GRAPHITE	-0.3430	
PYRRHOTITE	-3.9918		PYRITE	-4.8316	
FERROUS_OXIDE	-6.7142		SEPIOLITE	1034.8897	ssatd
SIDERITE	-5.0397		QUARTZ-ALPHA	-4.1568	
QUARTZ-BETA	-5.2348		CRISTOBALITE-ALPHA	-8.9592	
CHALCEDONY	-5.1113		AMORPHOUS_SILICA	-6.4501	

--- summary of solid solutions ---

mineral	aff. kcal/mol		mole frac.	lambda	state
ORTHOPYROXENE(SS)	-4.2578				
FERROSILITE		-4.25784	0.1015711	1.00000	
ENSTATITE-OR		-4.25784	0.8984289	1.00000	
OLIVINE	-9.7197				
FAYALITE		-9.71971	0.0432200	1.00000	
FORSTERITE		-9.71971	0.9567800	1.00000	
BIOTITE	-27.3696				
PHLOGOPITE		-27.36956	0.9992067	1.00000	
ANNITE		-27.36956	0.0007933	1.00000	
CLINOPYROXENE(SS)	-8.3007				
DIOPSIDE		-8.30074	0.9109891	1.00000	
HEDENBERGITE		-8.30074	0.0885115	1.00000	
JADEITE		-8.30074	0.0004994	1.00000	
GARNET(SS)	-13.8013				
PYROPE		-13.80134	0.5832122	1.00000	
ALMANDINE		-13.80134	0.1918209	1.00000	
GROSSULAR		-13.80134	0.2249670	1.00000	
CALCITE(SS)	0.0028				supersatd., ignored
CALCITE		0.00278	0.1716975	1.00000	
MAGNESITE		0.00278	0.7133111	1.00000	
SIDERITE		0.00278	0.1149914	1.00000	

solid solution product phases

xbar	lambda	activity	log xbar	log lambda	log activity

--- summary of gas species ---

gas	log fugacity	fugacity	partial pressure
CO2(G)	9.10553	1.27505E+09	
O2(G)	-9.60427	2.48731E-10	
S2(G)	3.99701	9.93136E+03	

```
  CH4(G)                         6.97347       9.40749E+06
  H2(G)                          3.80949       6.44892E+03
  H2S(G)                         7.27169       1.86935E+07
  H2O(G)                         6.90035       7.94972E+06



- - - - - - - - - - - - - - - - - - - - - - - - - - - - - - - -



stepping to zi=  1.2864E-04, delzi=  2.8641E-05, nord= 2
              ncycle=  0
steps completed =    15, iter = 12, ncorr = 0
most rapidly changing is zvclg1(AL+++            ) =    -17.8894

stepping to zi=  1.8592E-04, delzi=  5.7281E-05, nord= 2
              ncycle=  0
iter =   12
      0 supersaturated pure minerals
      1 supersaturated solid solutions

                         the most supersaturated phases     affinity, kcal

                           1   51500   CALCITE(SS)             0.00517059


  attempted species assemblage no.   2

             1      1  H2O
             2      2  NA+
             3      3  K+
             4      4  CA++
             5      5  MG++
             6      6  AL+++
             7      7  SIO2(AQ)
             8     13  H+
             9     14  CO3--
            10     16  CL-
            11     17  SO4--
            12     21  FE++
            13     29  O2(G)
            14      1  CALCITE(CALCITE
            15      2  CALCITE(MAGNESITE
            16      3  CALCITE(SIDERITE

steps completed =    16, iter = 18, ncorr = 0
- - - - - - - - - - - - - - - - - - - - - - - - - - - - - - - -



                    reaction progress        =  1.85921691364639E-04
                    log of reaction progress =      -3.7306699

                    temperature    =    900.000 degrees c
                    total pressure =  50000.000 bars

                    computing units remaining =      0.000


change in the product phase assemblage


                              --- reactant summary ---



        reactant            moles       delta moles      grams        delta grams

  CLINOPYROXENE(SS)       1.99998E+01    1.85922E-04    4.19230E+03    3.89724E-02
  GARNET(SS)              1.99998E+01    1.85922E-04    2.90864E+03    2.70392E-02
  COESITE                 1.99998E+01    1.85922E-04    1.20167E+03    1.11710E-02


               current total mass =  8.30261E+03 grams
               delta total mass   =  7.71825E-02 grams
```

delta total volume =   0.03703 cc

reactant	affinity	rel. rate
CLINOPYROXENE(SS)	18.7762	1.00000E+00
GARNET(SS)	13.3296	1.00000E+00
COESITE	3.5177	1.00000E+00

affinity of the overall irreversible reaction=   35.624 kcal
contributions from irreversible reactions
with no thermodynamic data are not included

--- element totals for the aqueous phase ---

element	mg/kg soln.	molal conc.	moles
O	7.273241E+05	1.286069E+02	1.294627E+02
NA	2.422836E+03	2.981462E-01	3.001301E-01
K	2.333923E+04	1.688762E+00	1.700000E+00
CA	4.542440E+04	3.206279E+00	3.227615E+00
MG	4.631494E+02	5.390950E-02	5.426824E-02
AL	3.304545E+00	3.464853E-04	3.487910E-04
SI	8.883071E+03	8.947894E-01	9.007437E-01
H	4.330956E+04	1.215643E+02	1.223733E+02
C	1.451748E+05	3.419416E+01	3.442170E+01
CL	1.244895E+03	9.933896E-02	1.000000E-01
S	2.089724E+03	1.844019E-01	1.856290E-01
FE	3.209140E+02	1.625656E-02	1.636474E-02
co3--		0.000000E+00	0.000000E+00
so4--		0.000000E+00	0.000000E+00
s--		0.000000E+00	0.000000E+00

warning-- co3--, so4--, and s-- totals require that routine comp1
have the names of non-carbonate carbon, sulfide sulfur,
and non-sulfate sulfur aqueous species

single ion activities and activity coefficients are here defined
with respect to the internal ph scale

	ph	eh	pe
internal ph scale	4.7393	1.3458	5.7816E+00
modified nbs ph scale	4.4186	1.4204	6.1023E+00
rational ph scale	4.4186	1.4204	6.1023E+00

phcl =   6.1381

oxygen fugacity = 2.48724E-10
log oxygen fugacity = -9.60428

activity of water =   0.99667
log activity of water = -0.00145
alkalinity = 0.000000E+00 equiv/kg solvent
(not def. for t.gt.50 c)

ionic strength = 5.212734E+00 molal
sum of molalities =   36.8431903636743
osmotic coefficient =   0.00502
equiv. stoich. ionic strength = 9.933896E-02 molal

mass of solution =   2.847870 kg
mass of solvent =   1.006654 kg
mass of solutes =   1.841216 kg
conc of solutes = 64.652381 per cent (w/w)

species	moles	grams	conc	log conc	log g	log act
H2O	5.58781E+01	1.00665E+03				
NA+	2.60468E-01	5.98809E+00	2.58746E-01	-0.58713	-0.32065	-0.90778
K+	1.47006E+00	5.74769E+01	1.46034E+00	0.16446	-0.32065	-0.15620
CA++	1.55317E-04	6.22510E-03	1.54290E-04	-3.81166	-1.28262	-5.09428
MG++	1.21148E-05	2.94451E-04	1.20348E-05	-4.91956	-1.28262	-6.20218
AL+++	1.66309E-18	4.48727E-17	1.65209E-18	-17.78197	-2.88589	-20.66786
SIO2(AQ)	4.00573E-01	2.40681E+01	3.97925E-01	-0.40020	0.00000	-0.40020
H+	3.83934E-05	3.86967E-05	3.81396E-05	-4.41862	-0.32065	-4.73928
CO3--	2.23735E-01	1.34262E+01	2.22256E-01	-0.65315	-1.28262	-1.93576
CL-	8.40896E-02	2.98123E+00	8.35337E-02	-1.07814	-0.32065	-1.39879
SO4--	2.94705E-04	2.83087E-02	2.92757E-04	-3.53349	-1.28262	-4.81611
FE++	9.27006E-08	5.17705E-06	9.20879E-08	-7.03580	-1.28262	-8.31842
O2(AQ)	1.87672E-16	6.00526E-15	1.86431E-16	-15.72948	0.00000	-15.72948
H2(AQ)	1.31318E-02	2.64710E-02	1.30450E-02	-1.88456	0.00000	-1.88456
CH4(AQ)	5.08599E-02	8.15925E-01	5.05237E-02	-1.29650	0.00000	-1.29650
HS-	4.00415E-02	1.32409E+00	3.97768E-02	-1.40037	-0.32065	-1.72102
FE+++	1.52521E-14	8.51786E-13	1.51513E-14	-13.81955	-2.88589	-16.70544
HCO3-	5.04536E-01	3.07853E+01	5.01201E-01	-0.29999	-0.32065	-0.62064
CLO4-	1.64164E-42	1.63262E-40	1.63079E-42	-41.78760	-0.32065	-42.10826
OH-	5.37979E-01	9.14956E+00	5.34422E-01	-0.27212	-0.32065	-0.59277
HCOO-	2.54644E+00	1.14635E+02	2.52961E+00	0.40305	-0.32065	0.08240
CH3COO-	2.97123E-02	1.75435E+00	2.95158E-02	-1.52994	-0.32065	-1.85060
CH3CH2COO-	4.97551E-01	3.63567E+01	4.94262E-01	-0.30604	-0.32065	-0.62670
CO(AQ)	6.42908E-02	1.80081E+00	6.38658E-02	-1.19473	0.00000	-1.19473
ETHANE(AQ)	2.16360E-04	6.50583E-03	2.14930E-04	-3.66770	0.00000	-3.66770
ETHYLENE(AQ)	4.69445E-07	1.31696E-05	4.66342E-07	-6.33130	0.00000	-6.33130
PROPANE(AQ)	1.23561E-06	5.44856E-05	1.22744E-06	-5.91100	0.00000	-5.91100
HEXANE(AQ)	2.99080E-13	2.57737E-11	2.97103E-13	-12.52709	0.00000	-12.52709
BENZENE(AQ)	9.96587E-13	7.78468E-11	9.89999E-13	-12.00437	0.00000	-12.00437
TOLUENE(AQ)	4.01715E-15	3.70141E-13	3.99059E-15	-14.39896	0.00000	-14.39896
SI2O4(AQ)	2.15716E-02	2.59223E+00	2.14290E-02	-1.66900	0.00000	-1.66900
ALO2-	2.54594E-05	1.50160E-03	2.52911E-05	-4.59703	-0.32065	-4.91769
ALO2(SIO2)-	3.23332E-04	3.84974E-02	3.21194E-04	-3.49323	-0.32065	-3.81389
CACL+	2.32049E-04	1.75274E-02	2.30515E-04	-3.63730	-0.32065	-3.95795
CACL2(AQ)	3.94266E-07	4.37580E-05	3.91660E-07	-6.40709	0.00000	-6.40709
CACO3(AQ)	5.71452E-03	5.71962E-01	5.67674E-03	-2.24590	0.00000	-2.24590
CA(HCO3)+	3.16654E+00	3.20128E+02	3.14561E+00	0.49770	-0.24504	0.25267
CA(OH)+	4.15713E-04	2.37320E-02	4.12965E-04	-3.38409	-0.32065	-3.70474
CA(HSIO3)+	5.45593E-02	6.39280E+00	5.41986E-02	-1.26601	-0.32065	-1.58667
FECL+	5.37742E-08	4.90959E-06	5.34188E-08	-7.27231	-0.32065	-7.59296
FECL2(AQ)	4.41196E-05	5.59229E-03	4.38279E-05	-4.35825	0.00000	-4.35825
FE(HSIO3)+	1.63205E-02	2.16962E+00	1.62126E-02	-1.79015	-0.32065	-2.11080
KCL(AQ)	1.44968E-02	1.08075E+00	1.44009E-02	-1.84161	0.00000	-1.84161
KOH	2.15428E-01	1.20867E+01	2.14004E-01	-0.66958	0.00000	-0.66958
KSO4-	1.26982E-05	1.71624E-03	1.26143E-05	-4.89914	-0.32065	-5.21979
MGCL+	5.12313E-06	3.06148E-04	5.08927E-06	-5.29334	-0.32065	-5.61400
MG(HCO3)+	6.75296E-04	5.76177E-02	6.70832E-04	-3.17339	-0.32065	-3.49404
MG(HSIO3)+	5.35757E-02	5.43239E+00	5.32215E-02	-1.27391	-0.32065	-1.59457
MGSO4(AQ)	9.62696E-09	1.15873E-06	9.56332E-09	-8.01939	0.00000	-8.01939
NACL(AQ)	8.17503E-04	4.77771E-02	8.12099E-04	-3.09039	0.00000	-3.09039
NACO3-	5.43259E-04	4.50899E-02	5.39667E-04	-3.26787	-0.32065	-3.58853
NAHCO3(AQ)	7.04581E-03	5.91896E-01	6.99923E-03	-2.15495	0.00000	-2.15495
NAHSIO3(AQ)	2.29674E-04	2.29861E-02	2.28156E-04	-3.64177	0.00000	-3.64177
NAOH(AQ)	3.10263E-02	1.24096E+00	3.08212E-02	-1.51115	0.00000	-1.51115
HSIO3-	3.32020E-01	2.55959E+01	3.29825E-01	-0.48172	-0.32065	-0.80237
HSO4-	1.89226E-03	1.83673E-01	1.87975E-03	-2.72590	-0.32065	-3.04655
H2S(AQ)	1.43388E-01	4.88605E+00	1.42440E-01	-0.84637	0.00000	-0.84637
CO2(AQ)	2.62921E+01	1.15711E+03	2.61183E+01	1.41694	0.00000	1.41694
HCL(AQ)	2.69915E-04	9.84134E-03	2.68131E-04	-3.57165	0.00000	-3.57165
CH3CH2COOH	5.20643E-05	3.85688E-03	5.17201E-05	-4.28634	0.00000	-4.28634
CH3COOH	1.69989E-03	1.02082E-01	1.68865E-03	-2.77246	0.00000	-2.77246
HCOOH	3.15830E-03	1.45362E-01	3.13742E-03	-2.50343	0.00000	-2.50343

--- activity ratios of cations ---

log (NA+ /h+**0)	3.8314969
log (K+ /h+**0)	4.5830789
log (CA++ /h+**0)	4.3842766
log (MG++ /h+**0)	3.2763755
log (AL+++ /h+**0)	-6.4500216
log (FE++ /h+**0)	1.1601404
log (FE+++ /h+**0)	-2.4876061

--- summary of solid product phases---

product	log moles	moles	grams	volume, cc
CALCITE(SS)	-3.7087159	1.95562E-04	1.77293E-02	5.81013E-03
CALCITE	-4.4734600	3.36155E-05	3.36455E-03	1.24041E-03
MAGNESITE	-3.8556610	1.39424E-04	1.17555E-02	3.90807E-03
SIDERITE	-4.6473962	2.25218E-05	2.60929E-03	6.61646E-04

--- grand summary of solid phases (e.s.+p.r.s.+reactants) ---

phase/end-member	log moles	moles	grams	volume, cc
COESITE	1.3010260	1.99998E+01	1.20167E+03	4.53756E+02
CLINOPYROXENE(SS)	1.3010260	1.99998E+01		
DIOPSIDE	0.6020560	3.99996E+00	8.66202E+02	2.64798E+02
HEDENBERGITE	0.3010260	1.99998E+00	4.96184E+02	1.32399E+02
JADEITE	1.1461240	1.39999E+01	2.82992E+03	8.44752E+02
GARNET(SS)	1.3010260	1.99998E+01		
PYROPE	1.0791772	1.19999E+01	1.61257E+03	1.35791E+03
ALMANDINE	0.7781472	5.99994E+00	9.95725E+02	6.78954E+02
GROSSULAR	0.3010260	1.99998E+00	3.00343E+02	2.50758E+02
CALCITE(SS)	-3.7087159	1.95562E-04		
CALCITE	-4.4734600	3.36155E-05	3.36455E-03	1.24041E-03
MAGNESITE	-3.8556610	1.39424E-04	1.17555E-02	3.90807E-03
SIDERITE	-4.6473962	2.25218E-05	2.60929E-03	6.61646E-04

	mass, grams	volume, cc
created	1.772931E-02	5.810127E-03
destroyed	7.718252E-02	3.702965E-02
net	-5.945321E-02	-3.121952E-02

warning-- these volume totals may be incomplete because
of missing partial molar volume data in the data base

--- mineral saturation state summary ---

mineral	affinity, kcal	state	mineral	affinity, kcal	state
DIAMOND	0.0001	ssatd	BRUCITE	-7.2219	
CALCITE	-4.1053		ARAGONITE	-2.3810	
MAGNESITE	-0.7888		DOLOMITE	-3.6578	
FORSTERITE	-9.8298		ENSTATITE-CL	-4.6057	
ENSTATITE-OR	-4.5107		ENSTATITE-PR	-5.7105	
DIOPSIDE	-8.5205		FERROSILITE	-9.5881	
COESITE	-3.5177		GRAPHITE	-0.3430	
PYRRHOTITE	-3.9910		PYRITE	-4.8308	
FERROUS_OXIDE	-6.7134		SEPIOLITE	1034.8842	ssatd
SIDERITE	-5.0390		QUARTZ-ALPHA	-4.1560	
QUARTZ-BETA	-5.2339		CRISTOBALITE-ALPHA	-8.9584	
CHALCEDONY	-5.1105		AMORPHOUS_SILICA	-6.4493	

--- summary of solid solutions ---

mineral	aff. kcal/mol	mole frac.	lambda	state

```
ORTHOPYROXENE(SS)        -4.2605
    FERROSILITE                -4.26051    0.1017558     1.00000
    ENSTATITE-OR               -4.26051    0.8982442     1.00000

OLIVINE                  -9.7264
    FAYALITE                   -9.72643    0.0433876     1.00000
    FORSTERITE                 -9.72643    0.9566124     1.00000

BIOTITE                 -26.4234
    PHLOGOPITE                -26.42336    0.9992019     1.00000
    ANNITE                    -26.42336    0.0007981     1.00000

CLINOPYROXENE(SS)        -8.3022
    DIOPSIDE                   -8.30219    0.9105941     1.00000
    HEDENBERGITE               -8.30219    0.0886522     1.00000
    JADEITE                    -8.30219    0.0007536     1.00000

GARNET(SS)              -13.1662
    PYROPE                    -13.16619    0.5827690     1.00000
    ALMANDINE                 -13.16619    0.1920636     1.00000
    GROSSULAR                 -13.16619    0.2251673     1.00000

CALCITE(SS)               0.0000                            saturated
    CALCITE                     0.00000    0.1718921     1.00000
    MAGNESITE                   0.00000    0.7129431     1.00000
    SIDERITE                    0.00000    0.1151648     1.00000


solid solution product phases

           xbar     lambda   activity  log xbar  log lambda log activity

  CALCITE(SS)
ideal solution

    CALCITE
            0.1719    1.0000    0.1719   -0.7647    0.0000   -0.7647
    MAGNESITE
            0.7129    1.0000    0.7129   -0.1469    0.0000   -0.1469
    SIDERITE
            0.1152    1.0000    0.1152   -0.9387    0.0000   -0.9387


          --- summary of gas species ---

          gas               log fugacity         fugacity         partial pressure

  CO2(G)                        9.10553       1.27505E+09
  O2(G)                        -9.60428       2.48724E-10
  S2(G)                         3.99701       9.93135E+03
  CH4(G)                        6.97350       9.40796E+06
  H2(G)                         3.80949       6.44901E+03
  H2S(G)                        7.27170       1.86938E+07
  H2O(G)                        6.90035       7.94972E+06



- - - - - - - - - - - - - - - - - - - - - - - - - - - - - - - -



stepping to zi=  1.8593E-04, delzi=  1.0000E-08, nord= 0
              ncycle=  0
steps completed =    17, iter =  7, ncorr = 0
most rapidly changing is zvclg1(SIDERITE          ) =     -4.6474

stepping to zi=  1.8594E-04, delzi=  1.0000E-08, nord= 0
              ncycle=  0
steps completed =    18, iter =  7, ncorr = 0
most rapidly changing is zvclg1(SIDERITE          ) =     -4.6473

stepping to zi=  1.8595E-04, delzi=  1.0000E-08, nord= 0
              ncycle=  0
steps completed =    19, iter =  7, ncorr = 0
most rapidly changing is zvclg1(SIDERITE          ) =     -4.6473
```

```
stepping to zi=  1.8597E-04, delzi=  1.8545E-08, nord= 1
              ncycle=  0
steps completed =    20, iter =  6, ncorr = 0
most rapidly changing is zvclg1(SIDERITE          ) =     -4.6473

stepping to zi=  1.8616E-04, delzi=  1.8545E-07, nord= 2
              ncycle=  0
steps completed =    21, iter =  8, ncorr = 0
most rapidly changing is zvclg1(SIDERITE          ) =     -4.6468

stepping to zi=  1.8801E-04, delzi=  1.8545E-06, nord= 2
              ncycle=  0
steps completed =    22, iter = 11, ncorr = 0
most rapidly changing is zvclg1(SIDERITE          ) =     -4.6425

stepping to zi=  2.0655E-04, delzi=  1.8545E-05, nord= 2
              ncycle=  0
steps completed =    23, iter = 13, ncorr = 0
most rapidly changing is zvclg1(SIDERITE          ) =     -4.6016

stepping to zi=  3.1623E-04, delzi=  1.0967E-04, nord= 3
              ncycle=  0
steps completed =    24, iter = 15, ncorr = 0
most rapidly changing is zvclg1(SIDERITE          ) =     -4.4159
- - - - - - - - - - - - - - - - - - - - - - - - - - - - - - - -



                    reaction progress        =  3.16227766016836E-04
                    log of reaction progress =      -3.5000000

                    temperature    =    900.000 degrees c
                    total pressure =  50000.000 bars

                    computing units remaining =      0.000


step size is limited by the print requirement


                              --- reactant summary ---



        reactant            moles       delta moles      grams        delta grams

  CLINOPYROXENE(SS)       1.99997E+01    3.16228E-04    4.19227E+03    6.62867E-02
  GARNET(SS)              1.99997E+01    3.16228E-04    2.90862E+03    4.59900E-02
  COESITE                 1.99997E+01    3.16228E-04    1.20167E+03    1.90003E-02


               current total mass =  8.30256E+03 grams
               delta total mass   =  1.31277E-01 grams
               delta total volume =      0.06298 cc


        reactant           affinity     rel. rate

  CLINOPYROXENE(SS)         18.1013    1.00000E+00
  GARNET(SS)                12.6882    1.00000E+00
  COESITE                    3.5169    1.00000E+00

         affinity of the overall irreversible reaction=    34.306 kcal
         contributions from irreversible reactions
         with no thermodynamic data are not included



     --- element totals for the aqueous phase ---

  element         mg/kg soln.         molal conc.          moles

     O         7.273199E+05        1.286082E+02        1.294638E+02
     NA        2.423537E+03        2.982371E-01        3.002214E-01
     K         2.333889E+04        1.688764E+00        1.700000E+00
     CA        4.542414E+04        3.206311E+00        3.227643E+00
```

MG	4.631994E+02	5.391617E-02	5.427489E-02
AL	4.990877E+00	5.233074E-04	5.267891E-04
SI	8.888082E+03	8.953082E-01	9.012649E-01
H	4.330892E+04	1.215645E+02	1.223733E+02
C	1.451721E+05	3.419406E+01	3.442156E+01
CL	1.244877E+03	9.933908E-02	1.000000E-01
S	2.089694E+03	1.844021E-01	1.856290E-01
FE	3.216212E+02	1.629264E-02	1.640104E-02
co3--		0.000000E+00	0.000000E+00
so4--		0.000000E+00	0.000000E+00
s--		0.000000E+00	0.000000E+00

warning-- co3--, so4--, and s-- totals require that routine comp1
have the names of non-carbonate carbon, sulfide sulfur,
and non-sulfate sulfur aqueous species

single ion activities and activity coefficients are here defined
with respect to the internal ph scale

	ph	eh	pe
internal ph scale	4.7393	1.3458	5.7816E+00
modified nbs ph scale	4.4186	1.4204	6.1023E+00
rational ph scale	4.4186	1.4204	6.1023E+00

phcl = 6.1381

oxygen fugacity = 2.48720E-10
log oxygen fugacity = -9.60429

activity of water = 0.99667
log activity of water = -0.00145
alkalinity = 0.000000E+00 equiv/kg solvent
(not def. for t.gt.50 c)

ionic strength = 5.212884E+00 molal
sum of molalities = 36.8436635590417
osmotic coefficient = 0.00502
equiv. stoich. ionic strength = 9.933908E-02 molal

mass of solution = 2.847912 kg
mass of solvent = 1.006653 kg
mass of solutes = 1.841259 kg
conc of solutes = 64.652940 per cent (w/w)

species	moles	grams	conc	log conc	log g	log act
H2O	5.58780E+01	1.00665E+03				
NA+	2.60548E-01	5.98994E+00	2.58826E-01	-0.58699	-0.32066	-0.90765
K+	1.47007E+00	5.74772E+01	1.46035E+00	0.16446	-0.32066	-0.15620
CA++	1.55325E-04	6.22541E-03	1.54298E-04	-3.81164	-1.28262	-5.09426
MG++	1.21124E-05	2.94392E-04	1.20324E-05	-4.91965	-1.28262	-6.20227
AL+++	2.51136E-18	6.77604E-17	2.49476E-18	-17.60297	-2.88590	-20.48887
SIO2(AQ)	4.00721E-01	2.40770E+01	3.98073E-01	-0.40004	0.00000	-0.40004
H+	3.83949E-05	3.86982E-05	3.81411E-05	-4.41861	-0.32066	-4.73926
CO3--	2.23720E-01	1.34253E+01	2.22242E-01	-0.65317	-1.28262	-1.93580
CL-	8.40892E-02	2.98121E+00	8.35334E-02	-1.07814	-0.32066	-1.39880
SO4--	2.94679E-04	2.83062E-02	2.92732E-04	-3.53353	-1.28262	-4.81615
FE++	9.28759E-08	5.18684E-06	9.22621E-08	-7.03498	-1.28262	-8.31760
O2(AQ)	1.87668E-16	6.00516E-15	1.86428E-16	-15.72949	0.00000	-15.72949
H2(AQ)	1.31319E-02	2.64712E-02	1.30451E-02	-1.88455	0.00000	-1.88455
CH4(AQ)	5.08615E-02	8.15950E-01	5.05253E-02	-1.29649	0.00000	-1.29649
HS-	4.00404E-02	1.32405E+00	3.97758E-02	-1.40038	-0.32066	-1.72104
FE+++	1.52817E-14	8.53435E-13	1.51807E-14	-13.81871	-2.88590	-16.70461
HCO3-	5.04518E-01	3.07842E+01	5.01183E-01	-0.30000	-0.32066	-0.62066
CLO4-	1.64158E-42	1.63256E-40	1.63073E-42	-41.78762	-0.32066	-42.10827
OH-	5.37960E-01	9.14924E+00	5.34404E-01	-0.27213	-0.32066	-0.59279

HCOO-	2.54637E+00	1.14632E+02	2.52954E+00	0.40304	-0.32066	0.08239
CH3COO-	2.97121E-02	1.75434E+00	2.95157E-02	-1.52995	-0.32066	-1.85060
CH3CH2COO-	4.97561E-01	3.63574E+01	4.94272E-01	-0.30603	-0.32066	-0.62669
CO(AQ)	6.42911E-02	1.80082E+00	6.38662E-02	-1.19473	0.00000	-1.19473
ETHANE(AQ)	2.16372E-04	6.50618E-03	2.14942E-04	-3.66768	0.00000	-3.66768
ETHYLENE(AQ)	4.69467E-07	1.31702E-05	4.66364E-07	-6.33128	0.00000	-6.33128
PROPANE(AQ)	1.23570E-06	5.44898E-05	1.22754E-06	-5.91097	0.00000	-5.91097
HEXANE(AQ)	2.99124E-13	2.57775E-11	2.97147E-13	-12.52703	0.00000	-12.52703
BENZENE(AQ)	9.96701E-13	7.78557E-11	9.90114E-13	-12.00431	0.00000	-12.00431
TOLUENE(AQ)	4.01770E-15	3.70192E-13	3.99115E-15	-14.39890	0.00000	-14.39890
SI2O4(AQ)	2.15876E-02	2.59415E+00	2.14449E-02	-1.66868	0.00000	-1.66868
ALO2-	3.84387E-05	2.26713E-03	3.81847E-05	-4.41811	-0.32066	-4.73877
ALO2(SIO2)-	4.88350E-04	5.81453E-02	4.85123E-04	-3.31415	-0.32066	-3.63480
CACL+	2.32057E-04	1.75280E-02	2.30524E-04	-3.63728	-0.32066	-3.95794
CACL2(AQ)	3.94277E-07	4.37592E-05	3.91671E-07	-6.40708	0.00000	-6.40708
CACO3(AQ)	5.71432E-03	5.71942E-01	5.67655E-03	-2.24592	0.00000	-2.24592
CA(HCO3)+	3.16655E+00	3.20129E+02	3.14562E+00	0.49771	-0.24504	0.25267
CA(OH)+	4.15716E-04	2.37321E-02	4.12968E-04	-3.38408	-0.32066	-3.70474
CA(HSIO3)+	5.45799E-02	6.39521E+00	5.42191E-02	-1.26585	-0.32066	-1.58650
FECL+	5.38752E-08	4.91880E-06	5.35191E-08	-7.27149	-0.32066	-7.59215
FECL2(AQ)	4.42020E-05	5.60274E-03	4.39099E-05	-4.35744	0.00000	-4.35744
FE(HSIO3)+	1.63567E-02	2.17444E+00	1.62486E-02	-1.78918	-0.32066	-2.10984
KCL(AQ)	1.44967E-02	1.08075E+00	1.44009E-02	-1.84161	0.00000	-1.84161
KOH	2.15421E-01	1.20863E+01	2.13997E-01	-0.66959	0.00000	-0.66959
KSO4-	1.26970E-05	1.71608E-03	1.26131E-05	-4.89918	-0.32066	-5.21983
MGCL+	5.12203E-06	3.06083E-04	5.08818E-06	-5.29344	-0.32066	-5.61409
MG(HCO3)+	6.75130E-04	5.76035E-02	6.70668E-04	-3.17349	-0.32066	-3.49415
MG(HSIO3)+	5.35825E-02	5.43308E+00	5.32284E-02	-1.27386	-0.32066	-1.59451
MGSO4(AQ)	9.62400E-09	1.15837E-06	9.56039E-09	-8.01952	0.00000	-8.01952
NACL(AQ)	8.17748E-04	4.77915E-02	8.12343E-04	-3.09026	0.00000	-3.09026
NACO3-	5.43386E-04	4.51005E-02	5.39795E-04	-3.26777	-0.32066	-3.58843
NAHCO3(AQ)	7.04770E-03	5.92055E-01	7.00112E-03	-2.15483	0.00000	-2.15483
NAHSIO3(AQ)	2.29822E-04	2.30009E-02	2.28303E-04	-3.64149	0.00000	-3.64149
NAOH(AQ)	3.10346E-02	1.24129E+00	3.08295E-02	-1.51103	0.00000	-1.51103
HSIO3-	3.32131E-01	2.56045E+01	3.29936E-01	-0.48157	-0.32066	-0.80223
HSO4-	1.89215E-03	1.83662E-01	1.87964E-03	-2.72592	-0.32066	-3.04658
H2S(AQ)	1.43389E-01	4.88609E+00	1.42441E-01	-0.84636	0.00000	-0.84636
CO2(AQ)	2.62920E+01	1.15711E+03	2.61182E+01	1.41694	0.00000	1.41694
HCL(AQ)	2.69923E-04	9.84163E-03	2.68139E-04	-3.57164	0.00000	-3.57164
CH3CH2COOH	5.20670E-05	3.85708E-03	5.17229E-05	-4.28632	0.00000	-4.28632
CH3COOH	1.69994E-03	1.02085E-01	1.68870E-03	-2.77245	0.00000	-2.77245
HCOOH	3.15831E-03	1.45363E-01	3.13744E-03	-2.50342	0.00000	-2.50342

--- activity ratios of cations ---

log (NA+ /h+**0)	3.8316147
log (K+ /h+**0)	4.5830647
log (CA++ /h+**0)	4.3842625
log (MG++ /h+**0)	3.2762530
log (AL+++ /h+**0)	-6.2710846
log (FE++ /h+**0)	1.1609253
log (FE+++ /h+**0)	-2.4868230

--- summary of solid product phases---

product	log moles	moles	grams	volume, cc
CALCITE(SS)	-3.4780030	3.32657E-04	3.01603E-02	9.88330E-03
CALCITE	-4.2427619	5.71792E-05	5.72302E-03	2.10991E-03
MAGNESITE	-3.6250714	2.37098E-04	1.99908E-02	6.64587E-03
SIDERITE	-4.4158990	3.83796E-05	4.44652E-03	1.12752E-03

--- grand summary of solid phases (e.s.+p.r.s.+reactants) ---

phase/end-member	log moles	moles	grams	volume, cc
COESITE	1.3010231	1.99997E+01	1.20167E+03	4.53753E+02
CLINOPYROXENE(SS)	1.3010231	1.99997E+01		
DIOPSIDE	0.6020531	3.99994E+00	8.66196E+02	2.64796E+02

HEDENBERGITE	0.3010231	1.99997E+00	4.96181E+02	1.32398E+02
JADEITE	1.1461212	1.39998E+01	2.82990E+03	8.44747E+02
GARNET(SS)	1.3010231	1.99997E+01		
PYROPE	1.0791744	1.19998E+01	1.61256E+03	1.35790E+03
ALMANDINE	0.7781444	5.99991E+00	9.95718E+02	6.78949E+02
GROSSULAR	0.3010231	1.99997E+00	3.00341E+02	2.50756E+02
CALCITE(SS)	-3.4780030	3.32657E-04		
CALCITE	-4.2427619	5.71792E-05	5.72302E-03	2.10991E-03
MAGNESITE	-3.6250714	2.37098E-04	1.99908E-02	6.64587E-03
SIDERITE	-4.4158990	3.83796E-05	4.44652E-03	1.12752E-03

	mass, grams	volume, cc
created	3.016030E-02	9.883298E-03
destroyed	1.312771E-01	6.298245E-02
net	-1.011168E-01	-5.309915E-02

warning-- these volume totals may be incomplete because
of missing partial molar volume data in the data base

--- mineral saturation state summary ---

mineral	affinity, kcal	state	mineral	affinity, kcal	state
DIAMOND	0.0001	ssatd	BRUCITE	-7.2225	
CALCITE	-4.1053		ARAGONITE	-2.3811	
MAGNESITE	-0.7895		DOLOMITE	-3.6585	
FORSTERITE	-9.8303		ENSTATITE-CL	-4.6055	
ENSTATITE-OR	-4.5105		ENSTATITE-PR	-5.7103	
DIOPSIDE	-8.5195		FERROSILITE	-9.5830	
COESITE	-3.5169		GRAPHITE	-0.3429	
PYRRHOTITE	-3.9868		PYRITE	-4.8266	
FERROUS_OXIDE	-6.7092		SEPIOLITE	1034.8855	ssatd
SIDERITE	-5.0348		QUARTZ-ALPHA	-4.1551	
QUARTZ-BETA	-5.2331		CRISTOBALITE-ALPHA	-8.9575	
CHALCEDONY	-5.1096		AMORPHOUS_SILICA	-6.4484	

--- summary of solid solutions ---

mineral	aff. kcal/mol		mole frac.	lambda	state
ORTHOPYROXENE(SS)	-4.2598				
FERROSILITE		-4.25981	0.1019470	1.00000	
ENSTATITE-OR		-4.25981	0.8980530	1.00000	
OLIVINE	-9.7265				
FAYALITE		-9.72645	0.0435614	1.00000	
FORSTERITE		-9.72645	0.9564386	1.00000	
BIOTITE	-25.4622				
PHLOGOPITE		-25.46224	0.9991969	1.00000	
ANNITE		-25.46224	0.0008031	1.00000	
CLINOPYROXENE(SS)	-8.2999				
DIOPSIDE		-8.29986	0.9100751	1.00000	
HEDENBERGITE		-8.29986	0.0887870	1.00000	
JADEITE		-8.29986	0.0011379	1.00000	
GARNET(SS)	-12.5252				
PYROPE		-12.52519	0.5825020	1.00000	
ALMANDINE		-12.52519	0.1923776	1.00000	
GROSSULAR		-12.52519	0.2251204	1.00000	
CALCITE(SS)	0.0000				saturated

```
    CALCITE                     0.00000    0.1718863     1.00000
    MAGNESITE                   0.00000    0.7127408     1.00000
    SIDERITE                    0.00000    0.1153729     1.00000


solid solution product phases

           xbar     lambda   activity  log xbar  log lambda log activity

  CALCITE(SS)
ideal solution

    CALCITE
            0.1719    1.0000    0.1719   -0.7648    0.0000   -0.7648
    MAGNESITE
            0.7127    1.0000    0.7127   -0.1471    0.0000   -0.1471
    SIDERITE
            0.1154    1.0000    0.1154   -0.9379    0.0000   -0.9379


          --- summary of gas species ---

          gas               log fugacity        fugacity        partial pressure

  CO2(G)                         9.10553      1.27505E+09
  O2(G)                         -9.60429      2.48720E-10
  S2(G)                          3.99701      9.93137E+03
  CH4(G)                         6.97351      9.40825E+06
  H2(G)                          3.80950      6.44906E+03
  H2S(G)                         7.27170      1.86940E+07
  H2O(G)                         6.90035      7.94972E+06



- - - - - - - - - - - - - - - - - - - - - - - - - - - - - - - - -



stepping to zi=  8.9547E-04, delzi=  5.7925E-04, nord= 3
              ncycle=  0
steps completed =    25, iter = 17, ncorr = 0
most rapidly changing is zvclg1(SIDERITE          ) =     -3.9602

stepping to zi=  1.0000E-03, delzi=  1.0453E-04, nord= 4
              ncycle=  0
steps completed =    26, iter = 15, ncorr = 0
most rapidly changing is zvclg1(SIDERITE          ) =     -3.9116
- - - - - - - - - - - - - - - - - - - - - - - - - - - - - - - - -



                    reaction progress        =  9.99999999999992E-04
                    log of reaction progress =      -3.0000000

                    temperature    =    900.000 degrees c
                    total pressure =  50000.000 bars

                    computing units remaining =      0.000


step size is limited by the print requirement


                              --- reactant summary ---



        reactant            moles       delta moles      grams        delta grams

  CLINOPYROXENE(SS)       1.99990E+01    1.00000E-03    4.19213E+03    2.09617E-01
  GARNET(SS)              1.99990E+01    1.00000E-03    2.90852E+03    1.45433E-01
  COESITE                 1.99990E+01    1.00000E-03    1.20163E+03    6.00843E-02


               current total mass =  8.30228E+03 grams
               delta total mass   =  4.15135E-01 grams
```

delta total volume =   0.19917 cc

reactant	affinity	rel. rate
CLINOPYROXENE(SS)	16.4267	1.00000E+00
GARNET(SS)	11.0980	1.00000E+00
COESITE	3.5123	1.00000E+00

affinity of the overall irreversible reaction=   31.037 kcal
contributions from irreversible reactions
with no thermodynamic data are not included

--- element totals for the aqueous phase ---

element	mg/kg soln.	molal conc.	moles
O	7.272981E+05	1.286150E+02	1.294699E+02
NA	2.427214E+03	2.987144E-01	3.007000E-01
K	2.333710E+04	1.688775E+00	1.700000E+00
CA	4.542276E+04	3.206480E+00	3.227793E+00
MG	4.634666E+02	5.395174E-02	5.431036E-02
AL	1.383898E+01	1.451176E-03	1.460822E-03
SI	8.914370E+03	8.980308E-01	9.040000E-01
H	4.330560E+04	1.215652E+02	1.223733E+02
C	1.451579E+05	3.419356E+01	3.442084E+01
CL	1.244781E+03	9.933969E-02	1.000000E-01
S	2.089534E+03	1.844032E-01	1.856290E-01
FE	3.253127E+02	1.648101E-02	1.659056E-02
co3--		0.000000E+00	0.000000E+00
so4--		0.000000E+00	0.000000E+00
s--		0.000000E+00	0.000000E+00

warning-- co3--, so4--, and s-- totals require that routine comp1
have the names of non-carbonate carbon, sulfide sulfur,
and non-sulfate sulfur aqueous species

single ion activities and activity coefficients are here defined
with respect to the internal ph scale

	ph	eh	pe
internal ph scale	4.7392	1.3458	5.7817E+00
modified nbs ph scale	4.4185	1.4204	6.1024E+00
rational ph scale	4.4185	1.4204	6.1024E+00

phcl =   6.1380

oxygen fugacity = 2.48698E-10
log oxygen fugacity = -9.60433

activity of water =   0.99667
log activity of water = -0.00145
alkalinity = 0.000000E+00 equiv/kg solvent
(not def. for t.gt.50 c)

ionic strength = 5.213671E+00 molal
sum of molalities =   36.8461456422517
osmotic coefficient =   0.00502
equiv. stoich. ionic strength = 9.933969E-02 molal

mass of solution =   2.848131 kg
mass of solvent =   1.006647 kg
mass of solutes =   1.841484 kg
conc of solutes = 64.655870 per cent (w/w)

species	moles	grams	conc	log conc	log g	log act
H2O	5.58776E+01	1.00665E+03				
NA+	2.60970E-01	5.99964E+00	2.59247E-01	-0.58629	-0.32066	-0.90695
K+	1.47011E+00	5.74787E+01	1.46040E+00	0.16447	-0.32066	-0.15619
CA++	1.55365E-04	6.22701E-03	1.54339E-04	-3.81153	-1.28265	-5.09417
MG++	1.20998E-05	2.94086E-04	1.20199E-05	-4.92010	-1.28265	-6.20274
AL+++	6.95764E-18	1.87728E-16	6.91170E-18	-17.16042	-2.88595	-20.04637
SIO2(AQ)	4.01500E-01	2.41239E+01	3.98849E-01	-0.39919	0.00000	-0.39919
H+	3.84025E-05	3.87058E-05	3.81489E-05	-4.41852	-0.32066	-4.73918
CO3--	2.23644E-01	1.34207E+01	2.22167E-01	-0.65332	-1.28265	-1.93597
CL-	8.40872E-02	2.98114E+00	8.35320E-02	-1.07815	-0.32066	-1.39881
SO4--	2.94544E-04	2.82932E-02	2.92599E-04	-3.53373	-1.28265	-4.81637
FE++	9.37883E-08	5.23779E-06	9.31690E-08	-7.03073	-1.28265	-8.31337
O2(AQ)	1.87651E-16	6.00460E-15	1.86412E-16	-15.72953	0.00000	-15.72953
H2(AQ)	1.31324E-02	2.64722E-02	1.30456E-02	-1.88453	0.00000	-1.88453
CH4(AQ)	5.08696E-02	8.16081E-01	5.05337E-02	-1.29642	0.00000	-1.29642
HS-	4.00351E-02	1.32388E+00	3.97707E-02	-1.40044	-0.32066	-1.72110
FE+++	1.54354E-14	8.62022E-13	1.53335E-14	-13.81436	-2.88595	-16.70031
HCO3-	5.04421E-01	3.07783E+01	5.01090E-01	-0.30008	-0.32066	-0.62075
CLO4-	1.64125E-42	1.63224E-40	1.63042E-42	-41.78770	-0.32066	-42.10836
OH-	5.37861E-01	9.14756E+00	5.34309E-01	-0.27221	-0.32066	-0.59287
HCOO-	2.54599E+00	1.14615E+02	2.52918E+00	0.40298	-0.32066	0.08232
CH3COO-	2.97114E-02	1.75429E+00	2.95152E-02	-1.52995	-0.32066	-1.85062
CH3CH2COO-	4.97609E-01	3.63610E+01	4.94323E-01	-0.30599	-0.32066	-0.62665
CO(AQ)	6.42930E-02	1.80087E+00	6.38685E-02	-1.19471	0.00000	-1.19471
ETHANE(AQ)	2.16433E-04	6.50802E-03	2.15004E-04	-3.66755	0.00000	-3.66755
ETHYLENE(AQ)	4.69579E-07	1.31734E-05	4.66478E-07	-6.33117	0.00000	-6.33117
PROPANE(AQ)	1.23620E-06	5.45119E-05	1.22804E-06	-5.91079	0.00000	-5.91079
HEXANE(AQ)	2.99356E-13	2.57975E-11	2.97379E-13	-12.52669	0.00000	-12.52669
BENZENE(AQ)	9.97299E-13	7.79024E-11	9.90714E-13	-12.00405	0.00000	-12.00405
TOLUENE(AQ)	4.02060E-15	3.70459E-13	3.99405E-15	-14.39859	0.00000	-14.39859
SI2O4(AQ)	2.16718E-02	2.60427E+00	2.15287E-02	-1.66698	0.00000	-1.66698
ALO2-	1.06401E-04	6.27556E-03	1.05698E-04	-3.97593	-0.32066	-4.29659
ALO2(SIO2)-	1.35442E-03	1.61264E-01	1.34548E-03	-2.87112	-0.32066	-3.19178
CACL+	2.32101E-04	1.75313E-02	2.30568E-04	-3.63720	-0.32066	-3.95786
CACL2(AQ)	3.94333E-07	4.37655E-05	3.91729E-07	-6.40701	0.00000	-6.40701
CACO3(AQ)	5.71326E-03	5.71836E-01	5.67554E-03	-2.24599	0.00000	-2.24599
CA(HCO3)+	3.16659E+00	3.20133E+02	3.14568E+00	0.49771	-0.24504	0.25268
CA(OH)+	4.15727E-04	2.37327E-02	4.12982E-04	-3.38407	-0.32066	-3.70473
CA(HSIO3)+	5.46879E-02	6.40786E+00	5.43268E-02	-1.26499	-0.32066	-1.58565
FECL+	5.44006E-08	4.96677E-06	5.40414E-08	-7.26727	-0.32066	-7.58793
FECL2(AQ)	4.46311E-05	5.65713E-03	4.43364E-05	-4.35324	0.00000	-4.35324
FE(HSIO3)+	1.65458E-02	2.19957E+00	1.64365E-02	-1.78419	-0.32066	-2.10485
KCL(AQ)	1.44965E-02	1.08073E+00	1.44007E-02	-1.84162	0.00000	-1.84162
KOH	2.15383E-01	1.20842E+01	2.13960E-01	-0.66967	0.00000	-0.66967
KSO4-	1.26909E-05	1.71526E-03	1.26071E-05	-4.89938	-0.32066	-5.22004
MGCL+	5.11635E-06	3.05743E-04	5.08256E-06	-5.29392	-0.32066	-5.61458
MG(HCO3)+	6.74267E-04	5.75299E-02	6.69815E-04	-3.17405	-0.32066	-3.49471
MG(HSIO3)+	5.36189E-02	5.43677E+00	5.32648E-02	-1.27356	-0.32066	-1.59422
MGSO4(AQ)	9.60861E-09	1.15652E-06	9.54516E-09	-8.02022	0.00000	-8.02022
NACL(AQ)	8.19037E-04	4.78668E-02	8.13629E-04	-3.08957	0.00000	-3.08957
NACO3-	5.44054E-04	4.51560E-02	5.40462E-04	-3.26723	-0.32066	-3.58790
NAHCO3(AQ)	7.05762E-03	5.92888E-01	7.01101E-03	-2.15422	0.00000	-2.15422
NAHSIO3(AQ)	2.30596E-04	2.30784E-02	2.29074E-04	-3.64002	0.00000	-3.64002
NAOH(AQ)	3.10786E-02	1.24305E+00	3.08733E-02	-1.51042	0.00000	-1.51042
HSIO3-	3.32718E-01	2.56498E+01	3.30521E-01	-0.48080	-0.32066	-0.80146
HSO4-	1.89156E-03	1.83606E-01	1.87907E-03	-2.72606	-0.32066	-3.04672
H2S(AQ)	1.43395E-01	4.88630E+00	1.42448E-01	-0.84634	0.00000	-0.84634
CO2(AQ)	2.62916E+01	1.15709E+03	2.61180E+01	1.41694	0.00000	1.41694
HCL(AQ)	2.69964E-04	9.84314E-03	2.68182E-04	-3.57157	0.00000	-3.57157
CH3CH2COOH	5.20813E-05	3.85814E-03	5.17374E-05	-4.28619	0.00000	-4.28619
CH3COOH	1.70020E-03	1.02101E-01	1.68897E-03	-2.77238	0.00000	-2.77238
HCOOH	3.15841E-03	1.45368E-01	3.13755E-03	-2.50341	0.00000	-2.50341

--- activity ratios of cations ---

log (NA+ /h+**0)	3.8322317
log (K+ /h+**0)	4.5829902
log (CA++ /h+**0)	4.3841884
log (MG++ /h+**0)	3.2756151
log (AL+++ /h+**0)	-5.8288293
log (FE++ /h+**0)	1.1649848
log (FE+++ /h+**0)	-2.4827729

--- summary of solid product phases---

product	log moles	moles	grams	volume, cc
CALCITE(SS)	-2.9777797	1.05250E-03	9.54597E-02	3.12711E-02
CALCITE	-3.7426161	1.80877E-04	1.81039E-02	6.67437E-03
MAGNESITE	-3.1254894	7.49050E-04	6.31555E-02	2.09959E-02
SIDERITE	-3.9116198	1.22569E-04	1.42004E-02	3.60083E-03

--- grand summary of solid phases (e.s.+p.r.s.+reactants) ---

phase/end-member	log moles	moles	grams	volume, cc
COESITE	1.3010083	1.99990E+01	1.20163E+03	4.53737E+02
CLINOPYROXENE(SS)	1.3010083	1.99990E+01		
DIOPSIDE	0.6020383	3.99980E+00	8.66166E+02	2.64787E+02
HEDENBERGITE	0.3010083	1.99990E+00	4.96164E+02	1.32393E+02
JADEITE	1.1461063	1.39993E+01	2.82980E+03	8.44718E+02
GARNET(SS)	1.3010083	1.99990E+01		
PYROPE	1.0791595	1.19994E+01	1.61250E+03	1.35785E+03
ALMANDINE	0.7781295	5.99970E+00	9.95684E+02	6.78926E+02
GROSSULAR	0.3010083	1.99990E+00	3.00331E+02	2.50747E+02
CALCITE(SS)	-2.9777797	1.05250E-03		
CALCITE	-3.7426161	1.80877E-04	1.81039E-02	6.67437E-03
MAGNESITE	-3.1254894	7.49050E-04	6.31555E-02	2.09959E-02
SIDERITE	-3.9116198	1.22569E-04	1.42004E-02	3.60083E-03

	mass, grams	volume, cc
created	9.545974E-02	3.127106E-02
destroyed	4.151346E-01	1.991680E-01
net	-3.196748E-01	-1.678969E-01

warning-- these volume totals may be incomplete because
of missing partial molar volume data in the data base

--- mineral saturation state summary ---

mineral	affinity, kcal	state	mineral	affinity, kcal	state
DIAMOND	0.0003	ssatd	BRUCITE	-7.2260	
CALCITE	-4.1058		ARAGONITE	-2.3815	
MAGNESITE	-0.7929		DOLOMITE	-3.6624	
FORSTERITE	-9.8326		ENSTATITE-CL	-4.6044	
ENSTATITE-OR	-4.5094		ENSTATITE-PR	-5.7091	
DIOPSIDE	-8.5143		FERROSILITE	-9.5567	
COESITE	-3.5123		GRAPHITE	-0.3428	
PYRRHOTITE	-3.9649		PYRITE	-4.8047	
FERROUS_OXIDE	-6.6874		SEPIOLITE	1034.8923	ssatd
SIDERITE	-5.0130		QUARTZ-ALPHA	-4.1506	
QUARTZ-BETA	-5.2285		CRISTOBALITE-ALPHA	-8.9529	
CHALCEDONY	-5.1051		AMORPHOUS_SILICA	-6.4439	

--- summary of solid solutions ---

mineral	aff. kcal/mol	mole frac.	lambda	state

```
ORTHOPYROXENE(SS)        -4.2561
    FERROSILITE                -4.25610    0.1029415     1.00000
    ENSTATITE-OR               -4.25610    0.8970585     1.00000

OLIVINE                  -9.7265
    FAYALITE                   -9.72654    0.0444716     1.00000
    FORSTERITE                 -9.72654    0.9555284     1.00000

BIOTITE                 -23.0851
    PHLOGOPITE                -23.08513    0.9991704     1.00000
    ANNITE                    -23.08513    0.0008296     1.00000

CLINOPYROXENE(SS)        -8.2876
    DIOPSIDE                   -8.28764    0.9073643     1.00000
    HEDENBERGITE               -8.28764    0.0894852     1.00000
    JADEITE                    -8.28764    0.0031505     1.00000

GARNET(SS)              -10.9374
    PYROPE                    -10.93737    0.5811149     1.00000
    ALMANDINE                 -10.93737    0.1940087     1.00000
    GROSSULAR                 -10.93737    0.2248764     1.00000

CALCITE(SS)               0.0000                             saturated
    CALCITE                     0.00000    0.1718555     1.00000
    MAGNESITE                   0.00000    0.7116890     1.00000
    SIDERITE                    0.00000    0.1164555     1.00000


solid solution product phases

           xbar     lambda   activity  log xbar  log lambda log activity

  CALCITE(SS)
ideal solution

    CALCITE
            0.1719    1.0000    0.1719   -0.7648    0.0000   -0.7648
    MAGNESITE
            0.7117    1.0000    0.7117   -0.1477    0.0000   -0.1477
    SIDERITE
            0.1165    1.0000    0.1165   -0.9338    0.0000   -0.9338


          --- summary of gas species ---

          gas               log fugacity         fugacity         partial pressure

  CO2(G)                         9.10552       1.27504E+09
  O2(G)                         -9.60433       2.48698E-10
  S2(G)                          3.99701       9.93147E+03
  CH4(G)                         6.97358       9.40982E+06
  H2(G)                          3.80952       6.44934E+03
  H2S(G)                         7.27172       1.86949E+07
  H2O(G)                         6.90035       7.94972E+06



- - - - - - - - - - - - - - - - - - - - - - - - - - - - - - - -



stepping to zi=  2.0453E-03, delzi=  1.0453E-03, nord= 4
              ncycle=  0
steps completed =    27, iter = 17, ncorr = 0
most rapidly changing is zvclg1(SIDERITE          ) =     -3.5945

stepping to zi=  3.1623E-03, delzi=  1.1170E-03, nord= 5
              ncycle=  0
steps completed =    28, iter = 17, ncorr = 0
most rapidly changing is zvclg1(SIDERITE          ) =     -3.3987
- - - - - - - - - - - - - - - - - - - - - - - - - - - - - - - -



                    reaction progress        =  3.16227766016835E-03
                    log of reaction progress =      -2.5000000
```

temperature   =   900.000 degrees c
total pressure = 50000.000 bars

computing units remaining =   0.000

step size is limited by the print requirement

--- reactant summary ---

reactant	moles	delta moles	grams	delta grams
CLINOPYROXENE(SS)	1.99968E+01	3.16228E-03	4.19168E+03	6.62867E-01
GARNET(SS)	1.99968E+01	3.16228E-03	2.90821E+03	4.59900E-01
COESITE	1.99968E+01	3.16228E-03	1.20150E+03	1.90003E-01

current total mass = 8.30138E+03 grams
delta total mass   = 1.31277E+00 grams
delta total volume =   0.62982 cc

reactant	affinity	rel. rate
CLINOPYROXENE(SS)	14.5903	1.00000E+00
GARNET(SS)	9.3613	1.00000E+00
COESITE	3.4980	1.00000E+00

affinity of the overall irreversible reaction=   27.450 kcal
contributions from irreversible reactions
with no thermodynamic data are not included

--- element totals for the aqueous phase ---

element	mg/kg soln.	molal conc.	moles
O	7.272291E+05	1.286364E+02	1.294890E+02
NA	2.438841E+03	3.002239E-01	3.022136E-01
K	2.333144E+04	1.688808E+00	1.700000E+00
CA	4.541841E+04	3.207012E+00	3.228267E+00
MG	4.643651E+02	5.407049E-02	5.442884E-02
AL	4.181022E+01	4.385429E-03	4.414493E-03
SI	8.997479E+03	9.066404E-01	9.126491E-01
H	4.329510E+04	1.215676E+02	1.223733E+02
C	1.451131E+05	3.419196E+01	3.441856E+01
CL	1.244480E+03	9.934162E-02	1.000000E-01
S	2.089027E+03	1.844068E-01	1.856290E-01
FE	3.367776E+02	1.706632E-02	1.717942E-02
co3--		0.000000E+00	0.000000E+00
so4--		0.000000E+00	0.000000E+00
s--		0.000000E+00	0.000000E+00

warning-- co3--, so4--, and s-- totals require that routine comp1
have the names of non-carbonate carbon, sulfide sulfur,
and non-sulfate sulfur aqueous species

single ion activities and activity coefficients are here defined
with respect to the internal ph scale

	ph	eh	pe
internal ph scale	4.7389	1.3458	5.7819E+00
modified nbs ph scale	4.4182	1.4205	6.1026E+00
rational ph scale	4.4182	1.4205	6.1026E+00

phcl =   6.1378

oxygen fugacity = 2.48630E-10
log oxygen fugacity = -9.60445

activity of water =   0.99667
log activity of water = -0.00145
alkalinity = 0.000000E+00 equiv/kg solvent
(not def. for t.gt.50 c)

ionic strength = 5.216153E+00 molal
sum of molalities =   36.8539841707671
osmotic coefficient =   0.00502
equiv. stoich. ionic strength = 9.934162E-02 molal

mass of solution =   2.848821 kg
mass of solvent =   1.006627 kg
mass of solutes =   1.842194 kg
conc of solutes = 64.665122 per cent (w/w)

species	moles	grams	conc	log conc	log g	log act
H2O	5.58766E+01	1.00663E+03				
NA+	2.62305E-01	6.03033E+00	2.60578E-01	-0.58406	-0.32068	-0.90474
K+	1.47023E+00	5.74835E+01	1.46055E+00	0.16452	-0.32068	-0.15616
CA++	1.55491E-04	6.23210E-03	1.54468E-04	-3.81116	-1.28272	-5.09388
MG++	1.20617E-05	2.93160E-04	1.19823E-05	-4.92146	-1.28272	-6.20418
AL+++	2.09636E-17	5.65631E-16	2.08256E-17	-16.68140	-2.88612	-19.56752
SIO2(AQ)	4.03966E-01	2.42720E+01	4.01307E-01	-0.39652	0.00000	-0.39652
H+	3.84265E-05	3.87301E-05	3.81735E-05	-4.41824	-0.32068	-4.73892
CO3--	2.23401E-01	1.34061E+01	2.21931E-01	-0.65378	-1.28272	-1.93650
CL-	8.40810E-02	2.98093E+00	8.35275E-02	-1.07817	-0.32068	-1.39885
SO4--	2.94115E-04	2.82520E-02	2.92178E-04	-3.53435	-1.28272	-4.81707
FE++	9.65945E-08	5.39451E-06	9.59585E-08	-7.01792	-1.28272	-8.30064
O2(AQ)	1.87595E-16	6.00282E-15	1.86360E-16	-15.72965	0.00000	-15.72965
H2(AQ)	1.31339E-02	2.64754E-02	1.30474E-02	-1.88447	0.00000	-1.88447
CH4(AQ)	5.08955E-02	8.16496E-01	5.05604E-02	-1.29619	0.00000	-1.29619
HS-	4.00180E-02	1.32331E+00	3.97545E-02	-1.40061	-0.32068	-1.72129
FE+++	1.59091E-14	8.88474E-13	1.58043E-14	-13.80122	-2.88612	-16.68734
HCO3-	5.04115E-01	3.07596E+01	5.00796E-01	-0.30034	-0.32068	-0.62102
CLO4-	1.64023E-42	1.63121E-40	1.62943E-42	-41.78797	-0.32068	-42.10864
OH-	5.37549E-01	9.14225E+00	5.34009E-01	-0.27245	-0.32068	-0.59313
HCOO-	2.54480E+00	1.14561E+02	2.52804E+00	0.40278	-0.32068	0.08210
CH3COO-	2.97090E-02	1.75415E+00	2.95134E-02	-1.52998	-0.32068	-1.85066
CH3CH2COO-	4.97763E-01	3.63722E+01	4.94486E-01	-0.30585	-0.32068	-0.62653
CO(AQ)	6.42990E-02	1.80104E+00	6.38757E-02	-1.19466	0.00000	-1.19466
ETHANE(AQ)	2.16628E-04	6.51386E-03	2.15201E-04	-3.66715	0.00000	-3.66715
ETHYLENE(AQ)	4.69936E-07	1.31834E-05	4.66842E-07	-6.33083	0.00000	-6.33083
PROPANE(AQ)	1.23779E-06	5.45820E-05	1.22965E-06	-5.91022	0.00000	-5.91022
HEXANE(AQ)	3.00091E-13	2.58608E-11	2.98115E-13	-12.52562	0.00000	-12.52562
BENZENE(AQ)	9.99196E-13	7.80506E-11	9.92617E-13	-12.00322	0.00000	-12.00322
TOLUENE(AQ)	4.02982E-15	3.71308E-13	4.00328E-15	-14.39758	0.00000	-14.39758
SI2O4(AQ)	2.19392E-02	2.63641E+00	2.17948E-02	-1.66165	0.00000	-1.66165
ALO2-	3.19709E-04	1.88565E-02	3.17604E-04	-3.49811	-0.32068	-3.81879
ALO2(SIO2)-	4.09478E-03	4.87544E-01	4.06782E-03	-2.39064	-0.32068	-2.71132
CACL+	2.32239E-04	1.75417E-02	2.30710E-04	-3.63693	-0.32068	-3.95761
CACL2(AQ)	3.94513E-07	4.37854E-05	3.91916E-07	-6.40681	0.00000	-6.40681
CACO3(AQ)	5.70992E-03	5.71501E-01	5.67233E-03	-2.24624	0.00000	-2.24624
CA(HCO3)+	3.16672E+00	3.20147E+02	3.14587E+00	0.49774	-0.24505	0.25269
CA(OH)+	4.15763E-04	2.37348E-02	4.13025E-04	-3.38402	-0.32068	-3.70470
CA(HSIO3)+	5.50295E-02	6.44790E+00	5.46672E-02	-1.26227	-0.32068	-1.58295
FECL+	5.60159E-08	5.11425E-06	5.56471E-08	-7.25456	-0.32068	-7.57524
FECL2(AQ)	4.59500E-05	5.82430E-03	4.56474E-05	-4.34058	0.00000	-4.34058
FE(HSIO3)+	1.71333E-02	2.27768E+00	1.70205E-02	-1.76903	-0.32068	-2.08971
KCL(AQ)	1.44957E-02	1.08067E+00	1.44002E-02	-1.84163	0.00000	-1.84163
KOH	2.15261E-01	1.20774E+01	2.13844E-01	-0.66990	0.00000	-0.66990
KSO4-	1.26716E-05	1.71265E-03	1.25882E-05	-4.90004	-0.32068	-5.22072
MGCL+	5.09910E-06	3.04712E-04	5.06553E-06	-5.29538	-0.32068	-5.61605
MG(HCO3)+	6.71635E-04	5.73053E-02	6.67213E-04	-3.17574	-0.32068	-3.49641
MG(HSIO3)+	5.37400E-02	5.44906E+00	5.33862E-02	-1.27257	-0.32068	-1.59325
MGSO4(AQ)	9.56136E-09	1.15083E-06	9.49841E-09	-8.02235	0.00000	-8.02235
NACL(AQ)	8.23113E-04	4.81050E-02	8.17693E-04	-3.08741	0.00000	-3.08741

NACO3-	5.46163E-04	4.53310E-02	5.42567E-04	-3.26555	-0.32068	-3.58623
NAHCO3(AQ)	7.08895E-03	5.95521E-01	7.04228E-03	-2.15229	0.00000	-2.15229
NAHSIO3(AQ)	2.33053E-04	2.33243E-02	2.31519E-04	-3.63541	0.00000	-3.63541
NAOH(AQ)	3.12174E-02	1.24860E+00	3.10118E-02	-1.50847	0.00000	-1.50847
HSIO3-	3.34574E-01	2.57928E+01	3.32371E-01	-0.47838	-0.32068	-0.79906
HSO4-	1.88971E-03	1.83426E-01	1.87727E-03	-2.72647	-0.32068	-3.04715
H2S(AQ)	1.43414E-01	4.88696E+00	1.42470E-01	-0.84628	0.00000	-0.84628
CO2(AQ)	2.62905E+01	1.15704E+03	2.61174E+01	1.41693	0.00000	1.41693
HCL(AQ)	2.70096E-04	9.84795E-03	2.68318E-04	-3.57135	0.00000	-3.57135
CH3CH2COOH	5.21267E-05	3.86151E-03	5.17835E-05	-4.28581	0.00000	-4.28581
CH3COOH	1.70102E-03	1.02150E-01	1.68982E-03	-2.77216	0.00000	-2.77216
HCOOH	3.15870E-03	1.45381E-01	3.13790E-03	-2.50336	0.00000	-2.50336

--- activity ratios of cations ---

log (NA+ /h+**0)	3.8341755
log (K+ /h+**0)	4.5827543
log (CA++ /h+**0)	4.3839539
log (MG++ /h+**0)	3.2736561
log (AL+++ /h+**0)	-5.3507670
log (FE++ /h+**0)	1.1771997
log (FE+++ /h+**0)	-2.4705881

--- summary of solid product phases---

product	log moles	moles	grams	volume, cc
CALCITE(SS)	-2.4770973	3.33352E-03	3.02689E-01	9.90553E-02
CALCITE	-3.2421797	5.72559E-04	5.73070E-02	2.11274E-02
MAGNESITE	-2.6267774	2.36169E-03	1.99124E-01	6.61981E-02
SIDERITE	-3.3987339	3.99269E-04	4.62578E-02	1.17297E-02

--- grand summary of solid phases (e.s.+p.r.s.+reactants) ---

phase/end-member	log moles	moles	grams	volume, cc
COESITE	1.3009613	1.99968E+01	1.20150E+03	4.53688E+02
CLINOPYROXENE(SS)	1.3009613	1.99968E+01		
DIOPSIDE	0.6019913	3.99937E+00	8.66073E+02	2.64758E+02
HEDENBERGITE	0.3009613	1.99968E+00	4.96110E+02	1.32379E+02
JADEITE	1.1460594	1.39978E+01	2.82949E+03	8.44626E+02
GARNET(SS)	1.3009613	1.99968E+01		
PYROPE	1.0791126	1.19981E+01	1.61233E+03	1.35771E+03
ALMANDINE	0.7780826	5.99905E+00	9.95576E+02	6.78853E+02
GROSSULAR	0.3009613	1.99968E+00	3.00298E+02	2.50720E+02
CALCITE(SS)	-2.4770973	3.33352E-03		
CALCITE	-3.2421797	5.72559E-04	5.73070E-02	2.11274E-02
MAGNESITE	-2.6267774	2.36169E-03	1.99124E-01	6.61981E-02
SIDERITE	-3.3987339	3.99269E-04	4.62578E-02	1.17297E-02

	mass, grams	volume, cc
created	3.026887E-01	9.905529E-02
destroyed	1.312771E+00	6.298245E-01
net	-1.010082E+00	-5.307692E-01

warning-- these volume totals may be incomplete because
of missing partial molar volume data in the data base

--- mineral saturation state summary ---

mineral	affinity, kcal	state	mineral	affinity, kcal	state
DIAMOND	0.0009	ssatd	BRUCITE	-7.2365	
CALCITE	-4.1071		ARAGONITE	-2.3828	
MAGNESITE	-0.8035		DOLOMITE	-3.6743	
FORSTERITE	-9.8393		ENSTATITE-CL	-4.6006	
ENSTATITE-OR	-4.5056		ENSTATITE-PR	-5.7053	
DIOPSIDE	-8.4974		FERROSILITE	-9.4768	
COESITE	-3.4980		GRAPHITE	-0.3422	
PYRRHOTITE	-3.8989		PYRITE	-4.7387	
FERROUS_OXIDE	-6.6219		SEPIOLITE	1034.9142	ssatd
SIDERITE	-4.9475		QUARTZ-ALPHA	-4.1363	
QUARTZ-BETA	-5.2142		CRISTOBALITE-ALPHA	-8.9386	
CHALCEDONY	-5.0907		AMORPHOUS_SILICA	-6.4296	

--- summary of solid solutions ---

mineral	aff. kcal/mol	mole frac.	lambda	state
ORTHOPYROXENE(SS)	-4.2444			
FERROSILITE	-4.24435	0.1059946	1.00000	
ENSTATITE-OR	-4.24435	0.8940054	1.00000	
OLIVINE	-9.7263			
FAYALITE	-9.72627	0.0473293	1.00000	
FORSTERITE	-9.72627	0.9526707	1.00000	
BIOTITE	-20.5085			
PHLOGOPITE	-20.50848	0.9990852	1.00000	
ANNITE	-20.50848	0.0009148	1.00000	
CLINOPYROXENE(SS)	-8.2490			
DIOPSIDE	-8.24901	0.8989315	1.00000	
HEDENBERGITE	-8.24901	0.0915947	1.00000	
JADEITE	-8.24901	0.0094738	1.00000	
GARNET(SS)	-9.2074			
PYROPE	-9.20737	0.5768816	1.00000	
ALMANDINE	-9.20737	0.1989912	1.00000	
GROSSULAR	-9.20737	0.2241273	1.00000	
CALCITE(SS)	0.0000			saturated
CALCITE	0.00000	0.1717583	1.00000	
MAGNESITE	0.00000	0.7084675	1.00000	
SIDERITE	0.00000	0.1197743	1.00000	

solid solution product phases

CALCITE(SS)
ideal solution

	xbar	lambda	activity	log xbar	log lambda	log activity
CALCITE	0.1718	1.0000	0.1718	-0.7651	0.0000	-0.7651
MAGNESITE	0.7085	1.0000	0.7085	-0.1497	0.0000	-0.1497
SIDERITE	0.1198	1.0000	0.1198	-0.9216	0.0000	-0.9216

--- summary of gas species ---

gas	log fugacity	fugacity	partial pressure
CO2(G)	9.10551	1.27501E+09	
O2(G)	-9.60445	2.48630E-10	
S2(G)	3.99703	9.93180E+03	
CH4(G)	6.97381	9.41478E+06	
H2(G)	3.80958	6.45024E+03	
H2S(G)	7.27179	1.86978E+07	

```
  H2O(G)                         6.90035       7.94972E+06



- - - - - - - - - - - - - - - - - - - - - - - - - - - - - - - -



stepping to zi=  5.1067E-03, delzi=  1.9444E-03, nord= 5
              ncycle=  0
steps completed =    29, iter = 17, ncorr = 0
most rapidly changing is zvclg1(SIDERITE          ) =     -3.1798

stepping to zi=  8.9956E-03, delzi=  3.8889E-03, nord= 4
              ncycle=  0
steps completed =    30, iter = 17, ncorr = 0
most rapidly changing is zvclg1(SIDERITE          ) =     -2.9142

stepping to zi=  1.0000E-02, delzi=  1.0044E-03, nord= 5
              ncycle=  0
steps completed =    31, iter = 15, ncorr = 0
most rapidly changing is zvclg1(SIDERITE          ) =     -2.8635
- - - - - - - - - - - - - - - - - - - - - - - - - - - - - - - -



                    reaction progress        =  9.99999999999991E-03
                    log of reaction progress =      -2.0000000

                    temperature    =    900.000 degrees c
                    total pressure =  50000.000 bars

                    computing units remaining =      0.000


step size is limited by the print requirement


                              --- reactant summary ---



        reactant            moles       delta moles      grams        delta grams

  CLINOPYROXENE(SS)       1.99900E+01    1.00000E-02    4.19024E+03    2.09617E+00
  GARNET(SS)              1.99900E+01    1.00000E-02    2.90721E+03    1.45433E+00
  COESITE                 1.99900E+01    1.00000E-02    1.20109E+03    6.00843E-01


               current total mass =  8.29854E+03 grams
               delta total mass   =  4.15135E+00 grams
               delta total volume =      1.99168 cc


        reactant           affinity     rel. rate

  CLINOPYROXENE(SS)         12.6387    1.00000E+00
  GARNET(SS)                 7.5416    1.00000E+00
  COESITE                    3.4532    1.00000E+00

         affinity of the overall irreversible reaction=    23.634 kcal
         contributions from irreversible reactions
         with no thermodynamic data are not included



     --- element totals for the aqueous phase ---

  element         mg/kg soln.         molal conc.          moles

     O         7.270122E+05        1.287042E+02        1.295492E+02
     NA        2.475575E+03        3.049973E-01        3.070000E-01
     K         2.331363E+04        1.688910E+00        1.700000E+00
     CA        4.540472E+04        3.208691E+00        3.229760E+00
     MG        4.676936E+02        5.450299E-02        5.486087E-02
     AL        1.301742E+02        1.366509E-02        1.375482E-02
```

SI	9.260046E+03	9.338680E-01	9.400000E-01
H	4.326205E+04	1.215750E+02	1.223733E+02
C	1.449718E+05	3.418683E+01	3.441131E+01
CL	1.243530E+03	9.934766E-02	1.000000E-01
S	2.087432E+03	1.844180E-01	1.856290E-01
FE	3.711350E+02	1.882290E-02	1.894650E-02
co3--		0.000000E+00	0.000000E+00
so4--		0.000000E+00	0.000000E+00
s--		0.000000E+00	0.000000E+00

warning-- co3--, so4--, and s-- totals require that routine comp1
have the names of non-carbonate carbon, sulfide sulfur,
and non-sulfate sulfur aqueous species

single ion activities and activity coefficients are here defined
with respect to the internal ph scale

	ph	eh	pe
internal ph scale	4.7381	1.3460	5.7827E+00
modified nbs ph scale	4.4173	1.4207	6.1034E+00
rational ph scale	4.4173	1.4207	6.1034E+00

phcl = 6.1371

oxygen fugacity = 2.48411E-10
log oxygen fugacity = -9.60483

activity of water = 0.99667
log activity of water = -0.00145
alkalinity = 0.000000E+00 equiv/kg solvent
(not def. for t.gt.50 c)

ionic strength = 5.223959E+00 molal
sum of molalities = 36.8786758181493
osmotic coefficient = 0.00502
equiv. stoich. ionic strength = 9.934766E-02 molal

mass of solution = 2.850998 kg
mass of solvent = 1.006566 kg
mass of solutes = 1.844432 kg
conc of solutes = 64.694248 per cent (w/w)

species	moles	grams	conc	log conc	log g	log act
H2O	5.58732E+01	1.00657E+03				
NA+	2.66528E-01	6.12741E+00	2.64789E-01	-0.57710	-0.32074	-0.89784
K+	1.47062E+00	5.74986E+01	1.46102E+00	0.16466	-0.32074	-0.15608
CA++	1.55894E-04	6.24822E-03	1.54877E-04	-3.81001	-1.28295	-5.09296
MG++	1.19568E-05	2.90609E-04	1.18788E-05	-4.92523	-1.28295	-6.20818
AL+++	6.47277E-17	1.74645E-15	6.43054E-17	-16.19175	-2.88663	-19.07838
SIO2(AQ)	4.11772E-01	2.47410E+01	4.09086E-01	-0.38819	0.00000	-0.38819
H+	3.85030E-05	3.88072E-05	3.82518E-05	-4.41735	-0.32074	-4.73808
CO3--	2.22631E-01	1.33599E+01	2.21179E-01	-0.65526	-1.28295	-1.93820
CL-	8.40621E-02	2.98025E+00	8.35137E-02	-1.07824	-0.32074	-1.39898
SO4--	2.92754E-04	2.81212E-02	2.90844E-04	-3.53634	-1.28295	-4.81929
FE++	1.04751E-07	5.85005E-06	1.04068E-07	-6.98268	-1.28295	-8.26563
O2(AQ)	1.87419E-16	5.99718E-15	1.86196E-16	-15.73003	0.00000	-15.73003
H2(AQ)	1.31389E-02	2.64854E-02	1.30532E-02	-1.88428	0.00000	-1.88428
CH4(AQ)	5.09777E-02	8.17815E-01	5.06452E-02	-1.29546	0.00000	-1.29546
HS-	3.99637E-02	1.32152E+00	3.97030E-02	-1.40118	-0.32074	-1.72191
FE+++	1.72932E-14	9.65775E-13	1.71804E-14	-13.76497	-2.88663	-16.65160
HCO3-	5.03143E-01	3.07003E+01	4.99861E-01	-0.30115	-0.32074	-0.62189
CLO4-	1.63697E-42	1.62798E-40	1.62629E-42	-41.78880	-0.32074	-42.10954
OH-	5.36557E-01	9.12538E+00	5.33056E-01	-0.27323	-0.32074	-0.59396
HCOO-	2.54101E+00	1.14390E+02	2.52443E+00	0.40216	-0.32074	0.08143
CH3COO-	2.97014E-02	1.75371E+00	2.95077E-02	-1.53006	-0.32074	-1.85080

CH3CH2COO-	4.98252E-01	3.64079E+01	4.95002E-01	-0.30539	-0.32074	-0.62613
CO(AQ)	6.43180E-02	1.80157E+00	6.38985E-02	-1.19451	0.00000	-1.19451
ETHANE(AQ)	2.17246E-04	6.53246E-03	2.15829E-04	-3.66589	0.00000	-3.66589
ETHYLENE(AQ)	4.71070E-07	1.32152E-05	4.67997E-07	-6.32976	0.00000	-6.32976
PROPANE(AQ)	1.24286E-06	5.48055E-05	1.23476E-06	-5.90842	0.00000	-5.90842
HEXANE(AQ)	3.02439E-13	2.60632E-11	3.00466E-13	-12.52220	0.00000	-12.52220
BENZENE(AQ)	1.00524E-12	7.85231E-11	9.98687E-13	-12.00057	0.00000	-12.00057
TOLUENE(AQ)	4.05923E-15	3.74018E-13	4.03275E-15	-14.39440	0.00000	-14.39440
SI2O4(AQ)	2.27967E-02	2.73944E+00	2.26479E-02	-1.64497	0.00000	-1.64497
ALO2-	9.78564E-04	5.77160E-02	9.72181E-04	-3.01225	-0.32074	-3.33299
ALO2(SIO2)-	1.27763E-02	1.52120E+00	1.26929E-02	-1.89644	-0.32074	-2.21718
CACL+	2.32679E-04	1.75749E-02	2.31161E-04	-3.63609	-0.32074	-3.95682
CACL2(AQ)	3.95091E-07	4.38496E-05	3.92514E-07	-6.40615	0.00000	-6.40615
CACO3(AQ)	5.69930E-03	5.70438E-01	5.66212E-03	-2.24702	0.00000	-2.24702
CA(HCO3)+	3.16714E+00	3.20189E+02	3.14648E+00	0.49783	-0.24508	0.25274
CA(OH)+	4.15875E-04	2.37412E-02	4.13162E-04	-3.38388	-0.32074	-3.70462
CA(HSIO3)+	5.61115E-02	6.57467E+00	5.57454E-02	-1.25379	-0.32074	-1.57453
FECL+	6.07041E-08	5.54229E-06	6.03081E-08	-7.21962	-0.32074	-7.54036
FECL2(AQ)	4.97745E-05	6.30906E-03	4.94498E-05	-4.30584	0.00000	-4.30584
FE(HSIO3)+	1.88966E-02	2.51208E+00	1.87733E-02	-1.72646	-0.32074	-2.04720
KCL(AQ)	1.44933E-02	1.08049E+00	1.43987E-02	-1.84168	0.00000	-1.84168
KOH	2.14877E-01	1.20558E+01	2.13476E-01	-0.67065	0.00000	-0.67065
KSO4-	1.26104E-05	1.70437E-03	1.25282E-05	-4.90211	-0.32074	-5.22285
MGCL+	5.05124E-06	3.01852E-04	5.01829E-06	-5.29944	-0.32074	-5.62018
MG(HCO3)+	6.64198E-04	5.66708E-02	6.59865E-04	-3.18054	-0.32074	-3.50128
MG(HSIO3)+	5.41797E-02	5.49363E+00	5.38262E-02	-1.26901	-0.32074	-1.58974
MGSO4(AQ)	9.42494E-09	1.13441E-06	9.36346E-09	-8.02856	0.00000	-8.02856
NACL(AQ)	8.36005E-04	4.88585E-02	8.30552E-04	-3.08063	0.00000	-3.08063
NACO3-	5.52785E-04	4.58806E-02	5.49179E-04	-3.26029	-0.32074	-3.58102
NAHCO3(AQ)	7.18772E-03	6.03818E-01	7.14084E-03	-2.14625	0.00000	-2.14625
NAHSIO3(AQ)	2.40901E-04	2.41097E-02	2.39330E-04	-3.62100	0.00000	-3.62100
NAOH(AQ)	3.16550E-02	1.26611E+00	3.14485E-02	-1.50240	0.00000	-1.50240
HSIO3-	3.40430E-01	2.62443E+01	3.38209E-01	-0.47081	-0.32074	-0.79155
HSO4-	1.88383E-03	1.82855E-01	1.87154E-03	-2.72780	-0.32074	-3.04854
H2S(AQ)	1.43476E-01	4.88906E+00	1.42540E-01	-0.84606	0.00000	-0.84606
CO2(AQ)	2.62867E+01	1.15687E+03	2.61152E+01	1.41689	0.00000	1.41689
HCL(AQ)	2.70518E-04	9.86333E-03	2.68753E-04	-3.57065	0.00000	-3.57065
CH3CH2COOH	5.22712E-05	3.87221E-03	5.19302E-05	-4.28458	0.00000	-4.28458
CH3COOH	1.70362E-03	1.02307E-01	1.69251E-03	-2.77147	0.00000	-2.77147
HCOOH	3.15963E-03	1.45424E-01	3.13902E-03	-2.50321	0.00000	-2.50321

--- activity ratios of cations ---

```
log (NA+   /h+**0)          3.8402476
log (K+    /h+**0)          4.5820049
log (CA++  /h+**0)          4.3832080
log (MG++  /h+**0)          3.2679928
log (AL+++ /h+**0)         -4.8641302
log (FE++  /h+**0)          1.2105393
log (FE+++ /h+**0)         -2.4373440
```

--- summary of solid product phases---

product	log moles	moles	grams	volume, cc
CALCITE(SS)	-1.9751574	1.05887E-02	9.64608E-01	3.14750E-01
CALCITE	-2.7410218	1.81542E-03	1.81704E-01	6.69892E-02
MAGNESITE	-2.1305371	7.40394E-03	6.24257E-01	2.07532E-01
SIDERITE	-2.8634906	1.36933E-03	1.58646E-01	4.02283E-02

--- grand summary of solid phases (e.s.+p.r.s.+reactants) ---

phase/end-member	log moles	moles	grams	volume, cc
COESITE	1.3008128	1.99900E+01	1.20109E+03	4.53533E+02
CLINOPYROXENE(SS)	1.3008128	1.99900E+01		
DIOPSIDE	0.6018428	3.99800E+00	8.65776E+02	2.64668E+02
HEDENBERGITE	0.3008128	1.99900E+00	4.95941E+02	1.32334E+02
JADEITE	1.1459108	1.39930E+01	2.82853E+03	8.44338E+02

GARNET(SS)	1.3008128	1.99900E+01		
PYROPE	1.0789640	1.19940E+01	1.61178E+03	1.35724E+03
ALMANDINE	0.7779340	5.99700E+00	9.95236E+02	6.78621E+02
GROSSULAR	0.3008128	1.99900E+00	3.00196E+02	2.50635E+02
CALCITE(SS)	-1.9751574	1.05887E-02		
CALCITE	-2.7410218	1.81542E-03	1.81704E-01	6.69892E-02
MAGNESITE	-2.1305371	7.40394E-03	6.24257E-01	2.07532E-01
SIDERITE	-2.8634906	1.36933E-03	1.58646E-01	4.02283E-02

	mass, grams	volume, cc
created	9.646075E-01	3.147499E-01
destroyed	4.151346E+00	1.991680E+00
net	-3.186738E+00	-1.676930E+00

warning-- these volume totals may be incomplete because
of missing partial molar volume data in the data base

--- mineral saturation state summary ---

mineral	affinity, kcal	state	mineral	affinity, kcal	state
DIAMOND	0.0027	ssatd	BRUCITE	-7.2669	
DIASPORE	-9.5962		CALCITE	-4.1113	
ARAGONITE	-2.3870		MAGNESITE	-0.8341	
DOLOMITE	-3.7091		FORSTERITE	-9.8554	
ENSTATITE-CL	-4.5862		ENSTATITE-OR	-4.4912	
ENSTATITE-PR	-5.6910		DIOPSIDE	-8.4423	
FERROSILITE	-9.2530		PYROPE	-8.7357	
COESITE	-3.4532		GRAPHITE	-0.3403	
PYRRHOTITE	-3.7188		PYRITE	-4.5585	
FERROUS_OXIDE	-6.4429		SEPIOLITE	1034.9876	ssatd
SIDERITE	-4.7687		QUARTZ-ALPHA	-4.0915	
QUARTZ-BETA	-5.1694		CRISTOBALITE-ALPHA	-8.8939	
CHALCEDONY	-5.0460		AMORPHOUS_SILICA	-6.3848	

--- summary of solid solutions ---

mineral	aff. kcal/mol		mole frac.	lambda	state
ORTHOPYROXENE(SS)	-4.2069				
FERROSILITE		-4.20689	0.1148108	1.00000	
ENSTATITE-OR		-4.20689	0.8851892	1.00000	
OLIVINE	-9.7207				
FAYALITE		-9.72070	0.0561190	1.00000	
FORSTERITE		-9.72070	0.9438810	1.00000	
BIOTITE	-17.8564				
PHLOGOPITE		-17.85644	0.9988027	1.00000	
ANNITE		-17.85644	0.0011973	1.00000	
CLINOPYROXENE(SS)	-8.1272				
DIOPSIDE		-8.12718	0.8735710	1.00000	
HEDENBERGITE		-8.12718	0.0973744	1.00000	
JADEITE		-8.12718	0.0290546	1.00000	
GARNET(SS)	-7.4042				
PYROPE		-7.40415	0.5648656	1.00000	
ALMANDINE		-7.40415	0.2131740	1.00000	
GROSSULAR		-7.40415	0.2219605	1.00000	
CALCITE(SS)	0.0000				saturated
CALCITE		0.00000	0.1714492	1.00000	

```
    MAGNESITE                    0.00000    0.6992304     1.00000
    SIDERITE                     0.00000    0.1293203     1.00000


solid solution product phases

           xbar     lambda   activity  log xbar  log lambda log activity

  CALCITE(SS)
ideal solution

    CALCITE
            0.1714    1.0000    0.1714   -0.7659    0.0000   -0.7659
    MAGNESITE
            0.6992    1.0000    0.6992   -0.1554    0.0000   -0.1554
    SIDERITE
            0.1293    1.0000    0.1293   -0.8883    0.0000   -0.8883


          --- summary of gas species ---

          gas                log fugacity         fugacity         partial pressure

  CO2(G)                         9.10548       1.27490E+09
  O2(G)                         -9.60483       2.48411E-10
  S2(G)                          3.99707       9.93280E+03
  CH4(G)                         6.97454       9.43057E+06
  H2(G)                          3.80977       6.45307E+03
  H2S(G)                         7.27200       1.87069E+07
  H2O(G)                         6.90035       7.94972E+06



- - - - - - - - - - - - - - - - - - - - - - - - - - - - - - - -



stepping to zi=  1.2009E-02, delzi=  2.0088E-03, nord= 3
              ncycle=  0
steps completed =    32, iter = 16, ncorr = 0
most rapidly changing is zvclg1(SIDERITE          ) =     -2.7750

stepping to zi=  1.6026E-02, delzi=  4.0176E-03, nord= 4
              ncycle=  0
steps completed =    33, iter = 16, ncorr = 0
most rapidly changing is zvclg1(SIDERITE          ) =     -2.6331

stepping to zi=  2.4062E-02, delzi=  8.0353E-03, nord= 5
              ncycle=  0
iter =   17
      1 supersaturated pure minerals
      0 supersaturated solid solutions

                         the most supersaturated phases     affinity, kcal

                           1       2   DIAMOND                0.00655254


  attempted species assemblage no.   2

             1      1  H2O
             2      2  NA+
             3      3  K+
             4      4  CA++
             5      5  MG++
             6      6  AL+++
             7      7  SIO2(AQ)
             8     13  H+
             9     14  CO3--
            10     16  CL-
            11     17  SO4--
            12     21  FE++
            13     29  O2(G)
            14      2  DIAMOND
            15      1  CALCITE(CALCITE
            16      2  CALCITE(MAGNESITE
```

17   3 CALCITE(SIDERITE

steps completed =   34, iter = 16, ncorr = 0

- -

reaction progress   = 2.40617703453101E-02
log of reaction progress =   -1.6186724

temperature   =   900.000 degrees c
total pressure = 50000.000 bars

computing units remaining =   0.000

change in the product phase assemblage

--- reactant summary ---

reactant	moles	delta moles	grams	delta grams
CLINOPYROXENE(SS)	1.99759E+01	2.40618E-02	4.18730E+03	5.04376E+00
GARNET(SS)	1.99759E+01	2.40618E-02	2.90517E+03	3.49938E+00
COESITE	1.99759E+01	2.40618E-02	1.20024E+03	1.44573E+00

current total mass = 8.29270E+03 grams
delta total mass   = 9.98887E+00 grams
delta total volume =   4.79233 cc

reactant	affinity	rel. rate
CLINOPYROXENE(SS)	11.0266	1.00000E+00
GARNET(SS)	6.0969	1.00000E+00
COESITE	3.3650	1.00000E+00

affinity of the overall irreversible reaction=   20.488 kcal
contributions from irreversible reactions
with no thermodynamic data are not included

--- element totals for the aqueous phase ---

element	mg/kg soln.	molal conc.	moles
O	7.266169E+05	1.288202E+02	1.296722E+02
NA	2.551138E+03	3.147614E-01	3.168432E-01
K	2.327885E+04	1.688830E+00	1.700000E+00
CA	4.537941E+04	3.211540E+00	3.232781E+00
MG	4.754777E+02	5.549026E-02	5.585728E-02
AL	3.114949E+02	3.274661E-02	3.296320E-02
SI	9.799503E+03	9.897012E-01	9.962471E-01
H	4.319752E+04	1.215692E+02	1.223733E+02
C	1.446297E+05	3.415550E+01	3.438141E+01
CL	1.241675E+03	9.934294E-02	1.000000E-01
S	2.084318E+03	1.844093E-01	1.856290E-01
FE	4.339878E+02	2.204245E-02	2.218824E-02
co3--		0.000000E+00	0.000000E+00
so4--		0.000000E+00	0.000000E+00
s--		0.000000E+00	0.000000E+00

warning-- co3--, so4--, and s-- totals require that routine comp1
have the names of non-carbonate carbon, sulfide sulfur,
and non-sulfate sulfur aqueous species

single ion activities and activity coefficients are here defined
with respect to the internal ph scale

	ph	eh	pe
internal ph scale	4.7370	1.3463	5.7838E+00
modified nbs ph scale	4.4162	1.4210	6.1047E+00
rational ph scale	4.4162	1.4210	6.1047E+00

phcl = 6.1363

oxygen fugacity = 2.48577E-10
log oxygen fugacity = -9.60454

activity of water = 0.99667
log activity of water = -0.00145
alkalinity = 0.000000E+00 equiv/kg solvent
(not def. for t.gt.50 c)

ionic strength = 5.239066E+00 molal
sum of molalities = 36.9169906976992
osmotic coefficient = 0.00501
equiv. stoich. ionic strength = 9.934294E-02 molal

mass of solution = 2.855257 kg
mass of solvent = 1.006614 kg
mass of solutes = 1.848643 kg
conc of solutes = 64.745238 per cent (w/w)

species	moles	grams	conc	log conc	log g	log act
H2O	5.58758E+01	1.00661E+03				
NA+	2.75166E-01	6.32601E+00	2.73358E-01	-0.56327	-0.32085	-0.88412
K+	1.47113E+00	5.75187E+01	1.46147E+00	0.16479	-0.32085	-0.15606
CA++	1.56530E-04	6.27373E-03	1.55502E-04	-3.80826	-1.28339	-5.09165
MG++	1.17652E-05	2.85953E-04	1.16879E-05	-4.93226	-1.28339	-6.21565
AL+++	1.51540E-16	4.08878E-15	1.50544E-16	-15.82234	-2.88763	-18.70996
SIO2(AQ)	4.27669E-01	2.56962E+01	4.24859E-01	-0.37176	0.00000	-0.37176
H+	3.86092E-05	3.89142E-05	3.83555E-05	-4.41617	-0.32085	-4.73702
CO3--	2.21668E-01	1.33021E+01	2.20212E-01	-0.65716	-1.28339	-1.94055
CL-	8.40304E-02	2.97913E+00	8.34783E-02	-1.07843	-0.32085	-1.39927
SO4--	2.92150E-04	2.80632E-02	2.90230E-04	-3.53726	-1.28339	-4.82065
FE++	1.18512E-07	6.61853E-06	1.17733E-07	-6.92910	-1.28339	-8.21249
O2(AQ)	1.87553E-16	6.00147E-15	1.86321E-16	-15.72974	0.00000	-15.72974
H2(AQ)	1.31351E-02	2.64778E-02	1.30488E-02	-1.88443	0.00000	-1.88443
CH4(AQ)	5.08869E-02	8.16358E-01	5.05525E-02	-1.29626	0.00000	-1.29626
HS-	3.98953E-02	1.31926E+00	3.96332E-02	-1.40194	-0.32085	-1.72279
FE+++	1.96412E-14	1.09690E-12	1.95121E-14	-13.70969	-2.88763	-16.59732
HCO3-	5.01814E-01	3.06192E+01	4.98516E-01	-0.30232	-0.32085	-0.62317
CLO4-	1.63854E-42	1.62954E-40	1.62778E-42	-41.78840	-0.32085	-42.10925
OH-	5.35404E-01	9.10577E+00	5.31886E-01	-0.27418	-0.32085	-0.59503
HCOO-	2.53345E+00	1.14050E+02	2.51680E+00	0.40085	-0.32085	0.08000
CH3COO-	2.95688E-02	1.74587E+00	2.93745E-02	-1.53203	-0.32085	-1.85288
CH3CH2COO-	4.95285E-01	3.61911E+01	4.92030E-01	-0.30801	-0.32085	-0.62886
CO(AQ)	6.42678E-02	1.80017E+00	6.38455E-02	-1.19487	0.00000	-1.19487
ETHANE(AQ)	2.16535E-04	6.51107E-03	2.15112E-04	-3.66734	0.00000	-3.66734
ETHYLENE(AQ)	4.69684E-07	1.31763E-05	4.66598E-07	-6.33106	0.00000	-6.33106
PROPANE(AQ)	1.23694E-06	5.45444E-05	1.22881E-06	-5.91051	0.00000	-5.91051
HEXANE(AQ)	2.99649E-13	2.58228E-11	2.97680E-13	-12.52625	0.00000	-12.52625
BENZENE(AQ)	9.97304E-13	7.79028E-11	9.90751E-13	-12.00404	0.00000	-12.00404
TOLUENE(AQ)	4.02114E-15	3.70508E-13	3.99472E-15	-14.39851	0.00000	-14.39851
SI2O4(AQ)	2.45896E-02	2.95490E+00	2.44281E-02	-1.61211	0.00000	-1.61211
ALO2-	2.26403E-03	1.33533E-01	2.24915E-03	-2.64798	-0.32085	-2.96883
ALO2(SIO2)-	3.06992E-02	3.65519E+00	3.04975E-02	-1.51574	-0.32085	-1.83658
CACL+	2.33292E-04	1.76212E-02	2.31759E-04	-3.63496	-0.32085	-3.95581
CACL2(AQ)	3.95763E-07	4.39242E-05	3.93163E-07	-6.40543	0.00000	-6.40543
CACO3(AQ)	5.68596E-03	5.69104E-01	5.64860E-03	-2.24806	0.00000	-2.24806
CA(HCO3)+	3.16796E+00	3.20272E+02	3.14715E+00	0.49792	-0.24515	0.25277
CA(OH)+	4.16233E-04	2.37616E-02	4.13498E-04	-3.38353	-0.32085	-3.70437
CA(HSIO3)+	5.83250E-02	6.83403E+00	5.79418E-02	-1.23701	-0.32085	-1.55786
FECL+	6.85794E-08	6.26130E-06	6.81288E-08	-7.16667	-0.32085	-7.48752
FECL2(AQ)	5.61794E-05	7.12091E-03	5.58103E-05	-4.25329	0.00000	-4.25329

FE(HSIO3)+	2.21319E-02	2.94218E+00	2.19865E-02	-1.65784	-0.32085	-1.97869
KCL(AQ)	1.44848E-02	1.07986E+00	1.43896E-02	-1.84195	0.00000	-1.84195
KOH	2.14371E-01	1.20274E+01	2.12963E-01	-0.67170	0.00000	-0.67170
KSO4-	1.25754E-05	1.69964E-03	1.24928E-05	-4.90334	-0.32085	-5.22419
MGCL+	4.96315E-06	2.96588E-04	4.93054E-06	-5.30711	-0.32085	-5.62795
MG(HCO3)+	6.51137E-04	5.55564E-02	6.46858E-04	-3.18919	-0.32085	-3.51004
MG(HSIO3)+	5.51894E-02	5.59602E+00	5.48268E-02	-1.26101	-0.32085	-1.58185
MGSO4(AQ)	9.23556E-09	1.11162E-06	9.17488E-09	-8.03740	0.00000	-8.03740
NACL(AQ)	8.62296E-04	5.03950E-02	8.56631E-04	-3.06721	0.00000	-3.06721
NACO3-	5.67628E-04	4.71125E-02	5.63898E-04	-3.24880	-0.32085	-3.56965
NAHCO3(AQ)	7.39697E-03	6.21396E-01	7.34837E-03	-2.13381	0.00000	-2.13381
NAHSIO3(AQ)	2.57600E-04	2.57810E-02	2.55908E-04	-3.59192	0.00000	-3.59192
NAOH(AQ)	3.25926E-02	1.30361E+00	3.23784E-02	-1.48974	0.00000	-1.48974
HSIO3-	3.52796E-01	2.71976E+01	3.50478E-01	-0.45534	-0.32085	-0.77619
HSO4-	1.88312E-03	1.82786E-01	1.87075E-03	-2.72798	-0.32085	-3.04883
H2S(AQ)	1.43546E-01	4.89144E+00	1.42603E-01	-0.84587	0.00000	-0.84587
CO2(AQ)	2.62749E+01	1.15635E+03	2.61023E+01	1.41668	0.00000	1.41668
HCL(AQ)	2.71011E-04	9.88131E-03	2.69230E-04	-3.56988	0.00000	-3.56988
CH3CH2COOH	5.20742E-05	3.85762E-03	5.17321E-05	-4.28624	0.00000	-4.28624
CH3COOH	1.69975E-03	1.02074E-01	1.68858E-03	-2.77248	0.00000	-2.77248
HCOOH	3.15717E-03	1.45310E-01	3.13642E-03	-2.50357	0.00000	-2.50357

--- activity ratios of cations ---

log (NA+ /h+**0)	3.8529041
log (K+ /h+**0)	4.5809605
log (CA++ /h+**0)	4.3823848
log (MG++ /h+**0)	3.2583858
log (AL+++ /h+**0)	-4.4989033
log (FE++ /h+**0)	1.2615483
log (FE+++ /h+**0)	-2.3862625

--- summary of solid product phases---

product	log moles	moles	grams	volume, cc
DIAMOND	-1.8342132	1.46483E-02	1.75941E-01	5.00971E-02
CALCITE(SS)	-1.5876754	2.58419E-02	2.36705E+00	7.68618E-01
CALCITE	-2.3545778	4.42000E-03	4.42394E-01	1.63098E-01
MAGNESITE	-1.7528768	1.76654E-02	1.48944E+00	4.95161E-01
SIDERITE	-2.4252143	3.75652E-03	4.35216E-01	1.10359E-01

--- grand summary of solid phases (e.s.+p.r.s.+reactants) ---

phase/end-member	log moles	moles	grams	volume, cc
DIAMOND	-1.8342132	1.46483E-02	1.75941E-01	5.00971E-02
COESITE	1.3005072	1.99759E+01	1.20024E+03	4.53214E+02
CLINOPYROXENE(SS)	1.3005072	1.99759E+01		
DIOPSIDE	0.6015372	3.99519E+00	8.65167E+02	2.64481E+02
HEDENBERGITE	0.3005072	1.99759E+00	4.95592E+02	1.32241E+02
JADEITE	1.1456052	1.39832E+01	2.82654E+03	8.43744E+02
GARNET(SS)	1.3005072	1.99759E+01		
PYROPE	1.0786584	1.19856E+01	1.61065E+03	1.35629E+03
ALMANDINE	0.7776284	5.99278E+00	9.94536E+02	6.78143E+02
GROSSULAR	0.3005072	1.99759E+00	2.99984E+02	2.50458E+02
CALCITE(SS)	-1.5876754	2.58419E-02		
CALCITE	-2.3545778	4.42000E-03	4.42394E-01	1.63098E-01
MAGNESITE	-1.7528768	1.76654E-02	1.48944E+00	4.95161E-01
SIDERITE	-2.4252143	3.75652E-03	4.35216E-01	1.10359E-01

```
                         mass, grams        volume, cc

          created         2.542994E+00       8.187150E-01
          destroyed       9.988873E+00       4.792335E+00
          net            -7.445879E+00      -3.973620E+00

         warning-- these volume totals may be incomplete because
         of missing partial molar volume data in the data base



          --- mineral saturation state summary ---


  mineral        affinity, kcal    state        mineral        affinity, kcal    state

DIAMOND                0.0000  satd         BRUCITE                -7.3184
DIASPORE              -7.6356               CALCITE                -4.1168
ARAGONITE             -2.3926               MAGNESITE              -0.8868
DOLOMITE              -3.7674               GROSSULAR              -9.5239
FORSTERITE            -9.8703               ENSTATITE-CL           -4.5496
ENSTATITE-OR          -4.4546               ENSTATITE-PR           -5.6544
DIOPSIDE              -8.3219               FERROSILITE            -8.8910
PYROPE                -7.3934               ALMANDINE              -9.3396
COESITE               -3.3650               GRAPHITE               -0.3430
PYRRHOTITE            -3.4440               PYRITE                 -4.2818
FERROUS_OXIDE         -6.1691               SEPIOLITE            1035.1491  ssatd
SIDERITE              -4.4960               QUARTZ-ALPHA           -4.0033
QUARTZ-BETA           -5.0812               CRISTOBALITE-ALPHA     -8.8057
CHALCEDONY            -4.9578               AMORPHOUS_SILICA       -6.2966


                    --- summary of solid solutions  ---

mineral               aff. kcal/mol  mole frac.    lambda      state

ORTHOPYROXENE(SS)        -4.1305
    FERROSILITE                -4.13051    0.1297758     1.00000
    ENSTATITE-OR               -4.13051    0.8702242     1.00000

OLIVINE                  -9.6939
    FAYALITE                   -9.69389    0.0728726     1.00000
    FORSTERITE                 -9.69389    0.9271274     1.00000

BIOTITE                 -15.7901
    PHLOGOPITE                -15.79012    0.9981812     1.00000
    ANNITE                    -15.79012    0.0018188     1.00000

CLINOPYROXENE(SS)        -7.8785
    DIOPSIDE                   -7.87847    0.8267954     1.00000
    HEDENBERGITE               -7.87847    0.1059645     1.00000
    JADEITE                    -7.87847    0.0672401     1.00000

GARNET(SS)               -5.9782
    PYROPE                     -5.97824    0.5449721     1.00000
    ALMANDINE                  -5.97824    0.2365048     1.00000
    GROSSULAR                  -5.97824    0.2185231     1.00000

CALCITE(SS)               0.0000                             saturated
    CALCITE                     0.00000    0.1710400     1.00000
    MAGNESITE                   0.00000    0.6835946     1.00000
    SIDERITE                    0.00000    0.1453654     1.00000


solid solution product phases

           xbar     lambda   activity  log xbar  log lambda log activity

  CALCITE(SS)
ideal solution

    CALCITE
            0.1710    1.0000    0.1710   -0.7669    0.0000   -0.7669
    MAGNESITE
            0.6836    1.0000    0.6836   -0.1652    0.0000   -0.1652
    SIDERITE
```

```
           0.1454    1.0000    0.1454   -0.8375    0.0000   -0.8375


         --- summary of gas species ---

         gas                log fugacity         fugacity         partial pressure

  CO2(G)                        9.10526       1.27427E+09
  O2(G)                        -9.60454       2.48577E-10
  S2(G)                         3.99774       9.94815E+03
  CH4(G)                        6.97374       9.41332E+06
  H2(G)                         3.80962       6.45092E+03
  H2S(G)                        7.27219       1.87151E+07
  H2O(G)                        6.90035       7.94972E+06



- - - - - - - - - - - - - - - - - - - - - - - - - - - - - - - -



stepping to zi=  2.4062E-02, delzi=  1.0000E-08, nord= 0
              ncycle=  0
steps completed =    35, iter =  4, ncorr = 0
most rapidly changing is zvclg1(SIDERITE          ) =     -2.4252

stepping to zi=  2.4062E-02, delzi=  1.0000E-08, nord= 0
              ncycle=  0
steps completed =    36, iter =  4, ncorr = 0
most rapidly changing is zvclg1(SIDERITE          ) =     -2.4252

stepping to zi=  2.4062E-02, delzi=  1.0000E-08, nord= 0
              ncycle=  0
steps completed =    37, iter =  4, ncorr = 0
most rapidly changing is zvclg1(SIDERITE          ) =     -2.4252

stepping to zi=  2.4062E-02, delzi=  1.0000E-07, nord= 1
              ncycle=  0
steps completed =    38, iter =  5, ncorr = 0
most rapidly changing is zvclg1(SIDERITE          ) =     -2.4252

stepping to zi=  2.4063E-02, delzi=  1.0000E-06, nord= 1
              ncycle=  0
steps completed =    39, iter =  7, ncorr = 0
most rapidly changing is zvclg1(SIDERITE          ) =     -2.4252

stepping to zi=  2.4073E-02, delzi=  1.0000E-05, nord= 2
              ncycle=  0
steps completed =    40, iter = 10, ncorr = 0
most rapidly changing is zvclg1(SIDERITE          ) =     -2.4250

stepping to zi=  2.4173E-02, delzi=  1.0000E-04, nord= 2
              ncycle=  0
steps completed =    41, iter = 12, ncorr = 0
most rapidly changing is zvclg1(SIDERITE          ) =     -2.4229

stepping to zi=  2.5173E-02, delzi=  1.0000E-03, nord= 3
              ncycle=  0
steps completed =    42, iter = 15, ncorr = 0
most rapidly changing is zvclg1(SIDERITE          ) =     -2.4021

stepping to zi=  2.8853E-02, delzi=  3.6805E-03, nord= 3
              ncycle=  0
steps completed =    43, iter = 16, ncorr = 0
most rapidly changing is zvclg1(SIDERITE          ) =     -2.3320

stepping to zi=  3.1623E-02, delzi=  2.7694E-03, nord= 4
              ncycle=  0
steps completed =    44, iter = 16, ncorr = 0
most rapidly changing is zvclg1(SIDERITE          ) =     -2.2845
- - - - - - - - - - - - - - - - - - - - - - - - - - - - - - - -



                    reaction progress        =  3.16227766016835E-02
                    log of reaction progress =      -1.5000000
```

temperature = 900.000 degrees c
total pressure = 50000.000 bars

computing units remaining = 0.000

step size is limited by the print requirement

--- reactant summary ---

reactant	moles	delta moles	grams	delta grams
CLINOPYROXENE(SS)	1.99684E+01	3.16228E-02	4.18571E+03	6.62867E+00
GARNET(SS)	1.99684E+01	3.16228E-02	2.90407E+03	4.59900E+00
COESITE	1.99684E+01	3.16228E-02	1.19979E+03	1.90003E+00

current total mass = 8.28956E+03 grams
delta total mass = 1.31277E+01 grams
delta total volume = 6.29825 cc

reactant	affinity	rel. rate
CLINOPYROXENE(SS)	10.4867	1.00000E+00
GARNET(SS)	5.6344	1.00000E+00
COESITE	3.3187	1.00000E+00

affinity of the overall irreversible reaction= 19.440 kcal
contributions from irreversible reactions
with no thermodynamic data are not included

--- element totals for the aqueous phase ---

element	mg/kg soln.	molal conc.	moles
O	7.263963E+05	1.288872E+02	1.297383E+02
NA	2.591646E+03	3.200229E-01	3.221359E-01
K	2.325994E+04	1.688849E+00	1.700000E+00
CA	4.536535E+04	3.213191E+00	3.234407E+00
MG	4.806742E+02	5.614294E-02	5.651365E-02
AL	4.087630E+02	4.300756E-02	4.329153E-02
SI	1.008879E+04	1.019758E+00	1.026491E+00
H	4.316242E+04	1.215705E+02	1.223733E+02
C	1.444581E+05	3.414309E+01	3.436854E+01
CL	1.240666E+03	9.934404E-02	1.000000E-01
S	2.082625E+03	1.844113E-01	1.856290E-01
FE	4.647030E+02	2.362194E-02	2.377792E-02
co3--		0.000000E+00	0.000000E+00
so4--		0.000000E+00	0.000000E+00
s--		0.000000E+00	0.000000E+00

warning-- co3--, so4--, and s-- totals require that routine comp1
have the names of non-carbonate carbon, sulfide sulfur,
and non-sulfate sulfur aqueous species

single ion activities and activity coefficients are here defined
with respect to the internal ph scale

	ph	eh	pe
internal ph scale	4.7363	1.3464	5.7845E+00
modified nbs ph scale	4.4154	1.4211	6.1054E+00
rational ph scale	4.4154	1.4211	6.1054E+00

phcl = 6.1357

oxygen fugacity = 2.48528E-10
log oxygen fugacity = -9.60463

activity of water = 0.99667
log activity of water = -0.00145
alkalinity = 0.000000E+00 equiv/kg solvent
(not def. for t.gt.50 c)

ionic strength = 5.247287E+00 molal
sum of molalities = 36.9400844553653
osmotic coefficient = 0.00501
equiv. stoich. ionic strength = 9.934404E-02 molal

mass of solution = 2.857579 kg
mass of solvent = 1.006603 kg
mass of solutes = 1.850976 kg
conc of solutes = 64.774270 per cent (w/w)

species	moles	grams	conc	log conc	log g	log act
H2O	5.58752E+01	1.00660E+03				
NA+	2.79826E-01	6.43313E+00	2.77990E-01	-0.55597	-0.32091	-0.87688
K+	1.47147E+00	5.75320E+01	1.46182E+00	0.16489	-0.32091	-0.15601
CA++	1.56918E-04	6.28927E-03	1.55889E-04	-3.80719	-1.28363	-5.09081
MG++	1.16961E-05	2.84273E-04	1.16194E-05	-4.93482	-1.28363	-6.21845
AL+++	1.96890E-16	5.31239E-15	1.95598E-16	-15.70864	-2.88816	-18.59680
SIO2(AQ)	4.36256E-01	2.62121E+01	4.33394E-01	-0.36312	0.00000	-0.36312
H+	3.86785E-05	3.89841E-05	3.84248E-05	-4.41539	-0.32091	-4.73630
CO3--	2.21007E-01	1.32624E+01	2.19557E-01	-0.65845	-1.28363	-1.94208
CL-	8.40132E-02	2.97852E+00	8.34621E-02	-1.07851	-0.32091	-1.39942
SO4--	2.91326E-04	2.79841E-02	2.89415E-04	-3.53848	-1.28363	-4.82211
FE++	1.24766E-07	6.96783E-06	1.23948E-07	-6.90676	-1.28363	-8.19039
O2(AQ)	1.87514E-16	6.00021E-15	1.86284E-16	-15.72983	0.00000	-15.72983
H2(AQ)	1.31363E-02	2.64801E-02	1.30501E-02	-1.88439	0.00000	-1.88439
CH4(AQ)	5.08964E-02	8.16511E-01	5.05626E-02	-1.29617	0.00000	-1.29617
HS-	3.98485E-02	1.31771E+00	3.95871E-02	-1.40245	-0.32091	-1.72335
FE+++	2.07255E-14	1.15746E-12	2.05896E-14	-13.68635	-2.88816	-16.57452
HCO3-	5.00943E-01	3.05661E+01	4.97657E-01	-0.30307	-0.32091	-0.62398
CLO4-	1.63756E-42	1.62856E-40	1.62682E-42	-41.78866	-0.32091	-42.10957
OH-	5.34581E-01	9.09177E+00	5.31074E-01	-0.27485	-0.32091	-0.59575
HCOO-	2.52930E+00	1.13863E+02	2.51271E+00	0.40014	-0.32091	0.07924
CH3COO-	2.95233E-02	1.74319E+00	2.93296E-02	-1.53269	-0.32091	-1.85360
CH3CH2COO-	4.94572E-01	3.61390E+01	4.91328E-01	-0.30863	-0.32091	-0.62954
CO(AQ)	6.42607E-02	1.79997E+00	6.38392E-02	-1.19491	0.00000	-1.19491
ETHANE(AQ)	2.16597E-04	6.51293E-03	2.15176E-04	-3.66721	0.00000	-3.66721
ETHYLENE(AQ)	4.69772E-07	1.31788E-05	4.66691E-07	-6.33097	0.00000	-6.33097
PROPANE(AQ)	1.23742E-06	5.45654E-05	1.22930E-06	-5.91034	0.00000	-5.91034
HEXANE(AQ)	2.99854E-13	2.58404E-11	2.97887E-13	-12.52595	0.00000	-12.52595
BENZENE(AQ)	9.97589E-13	7.79251E-11	9.91045E-13	-12.00391	0.00000	-12.00391
TOLUENE(AQ)	4.02269E-15	3.70651E-13	3.99630E-15	-14.39834	0.00000	-14.39834
SI2O4(AQ)	2.55873E-02	3.07479E+00	2.54195E-02	-1.59483	0.00000	-1.59483
ALO2-	2.91880E-03	1.72152E-01	2.89966E-03	-2.53765	-0.32091	-2.85856
ALO2(SIO2)-	4.03727E-02	4.80696E+00	4.01079E-02	-1.39677	-0.32091	-1.71768
CACL+	2.33696E-04	1.76517E-02	2.32163E-04	-3.63421	-0.32091	-3.95511
CACL2(AQ)	3.96261E-07	4.39795E-05	3.93662E-07	-6.40488	0.00000	-6.40488
CACO3(AQ)	5.67683E-03	5.68189E-01	5.63959E-03	-2.24875	0.00000	-2.24875
CA(HCO3)+	3.16840E+00	3.20316E+02	3.14762E+00	0.49798	-0.24518	0.25280
CA(OH)+	4.16397E-04	2.37710E-02	4.13666E-04	-3.38335	-0.32091	-3.70426
CA(HSIO3)+	5.95203E-02	6.97409E+00	5.91299E-02	-1.22819	-0.32091	-1.54910
FECL+	7.21450E-08	6.58683E-06	7.16717E-08	-7.14465	-0.32091	-7.46556
FECL2(AQ)	5.90724E-05	7.48761E-03	5.86849E-05	-4.23147	0.00000	-4.23147
FE(HSIO3)+	2.37186E-02	3.15312E+00	2.35631E-02	-1.62777	-0.32091	-1.94868
KCL(AQ)	1.44814E-02	1.07960E+00	1.43864E-02	-1.84205	0.00000	-1.84205
KOH	2.14034E-01	1.20085E+01	2.12631E-01	-0.67237	0.00000	-0.67237
KSO4-	1.25361E-05	1.69432E-03	1.24538E-05	-4.90470	-0.32091	-5.22560
MGCL+	4.93031E-06	2.94626E-04	4.89797E-06	-5.30998	-0.32091	-5.63089
MG(HCO3)+	6.45838E-04	5.51043E-02	6.41602E-04	-3.19273	-0.32091	-3.51364
MG(HSIO3)+	5.58512E-02	5.66312E+00	5.54848E-02	-1.25583	-0.32091	-1.57673
MGSO4(AQ)	9.14541E-09	1.10077E-06	9.08542E-09	-8.04165	0.00000	-8.04165
NACL(AQ)	8.76486E-04	5.12242E-02	8.70736E-04	-3.06011	0.00000	-3.06011

NACO3-	5.75206E-04	4.77415E-02	5.71433E-04	-3.24304	-0.32091	-3.56394
NAHCO3(AQ)	7.50718E-03	6.30654E-01	7.45793E-03	-2.12738	0.00000	-2.12738
NAHSIO3(AQ)	2.66744E-04	2.66961E-02	2.64994E-04	-3.57676	0.00000	-3.57676
NAOH(AQ)	3.30848E-02	1.32329E+00	3.28677E-02	-1.48323	0.00000	-1.48323
HSIO3-	3.59331E-01	2.77014E+01	3.56974E-01	-0.44736	-0.32091	-0.76827
HSO4-	1.88017E-03	1.82499E-01	1.86783E-03	-2.72866	-0.32091	-3.04957
H2S(AQ)	1.43596E-01	4.89316E+00	1.42654E-01	-0.84571	0.00000	-0.84571
CO2(AQ)	2.62694E+01	1.15611E+03	2.60971E+01	1.41659	0.00000	1.41659
HCL(AQ)	2.71370E-04	9.89439E-03	2.69590E-04	-3.56930	0.00000	-3.56930
CH3CH2COOH	5.20788E-05	3.85796E-03	5.17372E-05	-4.28620	0.00000	-4.28620
CH3COOH	1.69973E-03	1.02073E-01	1.68858E-03	-2.77248	0.00000	-2.77248
HCOOH	3.15682E-03	1.45294E-01	3.13611E-03	-2.50361	0.00000	-2.50361

--- activity ratios of cations ---

log (NA+ /h+**0)	3.8594179
log (K+ /h+**0)	4.5802824
log (CA++ /h+**0)	4.3817775
log (MG++ /h+**0)	3.2541449
log (AL+++ /h+**0)	-4.3879124
log (FE++ /h+**0)	1.2822025
log (FE+++ /h+**0)	-2.3656298

--- summary of solid product phases---

product	log moles	moles	grams	volume, cc
DIAMOND	-1.7148196	1.92833E-02	2.31611E-01	6.59487E-02
CALCITE(SS)	-1.4675678	3.40747E-02	3.12859E+00	1.01373E+00
CALCITE	-2.2351637	5.81884E-03	5.82403E-01	2.14715E-01
MAGNESITE	-1.6370963	2.30624E-02	1.94448E+00	6.46438E-01
SIDERITE	-2.2845387	5.19351E-03	6.01701E-01	1.52575E-01

--- grand summary of solid phases (e.s.+p.r.s.+reactants) ---

phase/end-member	log moles	moles	grams	volume, cc
DIAMOND	-1.7148196	1.92833E-02	2.31611E-01	6.59487E-02
COESITE	1.3003428	1.99684E+01	1.19979E+03	4.53043E+02
CLINOPYROXENE(SS)	1.3003428	1.99684E+01		
DIOPSIDE	0.6013728	3.99368E+00	8.64840E+02	2.64381E+02
HEDENBERGITE	0.3003428	1.99684E+00	4.95404E+02	1.32191E+02
JADEITE	1.1454408	1.39779E+01	2.82547E+03	8.43424E+02
GARNET(SS)	1.3003428	1.99684E+01		
PYROPE	1.0784940	1.19810E+01	1.61004E+03	1.35577E+03
ALMANDINE	0.7774640	5.99051E+00	9.94160E+02	6.77886E+02
GROSSULAR	0.3003428	1.99684E+00	2.99871E+02	2.50364E+02
CALCITE(SS)	-1.4675678	3.40747E-02		
CALCITE	-2.2351637	5.81884E-03	5.82403E-01	2.14715E-01
MAGNESITE	-1.6370963	2.30624E-02	1.94448E+00	6.46438E-01
SIDERITE	-2.2845387	5.19351E-03	6.01701E-01	1.52575E-01

	mass, grams	volume, cc
created	3.360199E+00	1.079677E+00
destroyed	1.312771E+01	6.298245E+00
net	-9.767509E+00	-5.218568E+00

warning-- these volume totals may be incomplete because
of missing partial molar volume data in the data base

```
         --- mineral saturation state summary ---


  mineral        affinity, kcal    state        mineral        affinity, kcal    state

DIAMOND                0.0000  satd         BRUCITE                -7.3412
DIASPORE              -7.0398               CALCITE                -4.1206
ARAGONITE             -2.3963               MAGNESITE              -0.9101
DOLOMITE              -3.7943               GROSSULAR              -9.0840
FORSTERITE            -9.8695               ENSTATITE-CL           -4.5260
ENSTATITE-OR          -4.4310               ENSTATITE-PR           -5.6308
DIOPSIDE              -8.2552               FERROSILITE            -8.7338
PYROPE                -6.9730               ALMANDINE              -8.7854
COESITE               -3.3187               GRAPHITE               -0.3430
PYRRHOTITE            -3.3323               PYRITE                 -4.1695
FERROUS_OXIDE         -6.0582               SEPIOLITE            1035.2427  ssatd
SIDERITE              -4.3856               QUARTZ-ALPHA           -3.9569
QUARTZ-BETA           -5.0349               CRISTOBALITE-ALPHA     -8.7593
CHALCEDONY            -4.9114               AMORPHOUS_SILICA       -6.2502


                   --- summary of solid solutions  ---

mineral               aff. kcal/mol  mole frac.    lambda      state

ORTHOPYROXENE(SS)        -4.0891
    FERROSILITE                -4.08912    0.1363880     1.00000
    ENSTATITE-OR               -4.08912    0.8636120     1.00000

OLIVINE                  -9.6725
    FAYALITE                   -9.67250    0.0810078     1.00000
    FORSTERITE                 -9.67250    0.9189922     1.00000

BIOTITE                 -15.1263
    PHLOGOPITE                -15.12632    0.9978406     1.00000
    ANNITE                    -15.12632    0.0021594     1.00000

CLINOPYROXENE(SS)        -7.7471
    DIOPSIDE                   -7.74706    0.8041718     1.00000
    HEDENBERGITE               -7.74706    0.1091456     1.00000
    JADEITE                    -7.74706    0.0866826     1.00000

GARNET(SS)               -5.5213
    PYROPE                     -5.52128    0.5364926     1.00000
    ALMANDINE                  -5.52128    0.2465752     1.00000
    GROSSULAR                  -5.52128    0.2169322     1.00000

CALCITE(SS)               0.0000                                   saturated
    CALCITE                     0.00000    0.1707671     1.00000
    MAGNESITE                   0.00000    0.6768174     1.00000
    SIDERITE                    0.00000    0.1524155     1.00000


solid solution product phases

           xbar     lambda   activity  log xbar  log lambda log activity

  CALCITE(SS)
ideal solution

    CALCITE
            0.1708    1.0000    0.1708   -0.7676    0.0000   -0.7676
    MAGNESITE
            0.6768    1.0000    0.6768   -0.1695    0.0000   -0.1695
    SIDERITE
            0.1524    1.0000    0.1524   -0.8170    0.0000   -0.8170


          --- summary of gas species ---

          gas               log fugacity         fugacity         partial pressure

  CO2(G)                        9.10517       1.27402E+09
  O2(G)                        -9.60463       2.48528E-10
```

```
  S2(G)                         3.99797      9.95342E+03
  CH4(G)                        6.97383      9.41519E+06
  H2(G)                         3.80966      6.45156E+03
  H2S(G)                        7.27235      1.87219E+07
  H2O(G)                        6.90035      7.94972E+06



- - - - - - - - - - - - - - - - - - - - - - - - - - - - - - - -



stepping to zi=  4.1978E-02, delzi=  1.0355E-02, nord= 5
              ncycle=  0
steps completed =    45, iter = 16, ncorr = 0
most rapidly changing is zvclg1(SIDERITE          ) =     -2.1368

stepping to zi=  5.9800E-02, delzi=  1.7822E-02, nord= 5
              ncycle=  0
steps completed =    46, iter = 18, ncorr = 0
most rapidly changing is zvclg1(SIDERITE          ) =     -1.9509

stepping to zi=  8.7675E-02, delzi=  2.7875E-02, nord= 6
              ncycle=  0
steps completed =    47, iter = 18, ncorr = 0
most rapidly changing is zvclg1(SIDERITE          ) =     -1.7502

stepping to zi=  1.0000E-01, delzi=  1.2325E-02, nord= 6
              ncycle=  0
steps completed =    48, iter = 17, ncorr = 0
most rapidly changing is zvclg1(SIDERITE          ) =     -1.6818
- - - - - - - - - - - - - - - - - - - - - - - - - - - - - - - -



                    reaction progress        =  9.99999999999992E-02
                    log of reaction progress =      -1.0000000

                    temperature    =    900.000 degrees c
                    total pressure =  50000.000 bars

                    computing units remaining =      0.000


step size is limited by the print requirement


                              --- reactant summary ---



        reactant            moles       delta moles      grams        delta grams

  CLINOPYROXENE(SS)       1.99000E+01    1.00000E-01    4.17138E+03    2.09617E+01
  GARNET(SS)              1.99000E+01    1.00000E-01    2.89412E+03    1.45433E+01
  COESITE                 1.99000E+01    1.00000E-01    1.19568E+03    6.00843E+00


               current total mass =  8.26118E+03 grams
               delta total mass   =  4.15135E+01 grams
               delta total volume =     19.91680 cc


        reactant           affinity     rel. rate

  CLINOPYROXENE(SS)          7.8544    1.00000E+00
  GARNET(SS)                 3.6038    1.00000E+00
  COESITE                    2.9383    1.00000E+00

         affinity of the overall irreversible reaction=    14.396 kcal
         contributions from irreversible reactions
         with no thermodynamic data are not included



     --- element totals for the aqueous phase ---
```

element	mg/kg soln.	molal conc.	moles
O	7.244412E+05	1.294887E+02	1.303327E+02
NA	2.955170E+03	3.676042E-01	3.700000E-01
K	2.309154E+04	1.688992E+00	1.700000E+00
CA	4.524236E+04	3.228123E+00	3.249162E+00
MG	5.412659E+02	6.368654E-02	6.410161E-02
AL	1.281342E+03	1.358097E-01	1.366948E-01
SI	1.268445E+04	1.291582E+00	1.300000E+00
H	4.284993E+04	1.215809E+02	1.223733E+02
C	1.429240E+05	3.402974E+01	3.425153E+01
CL	1.231683E+03	9.935247E-02	1.000000E-01
S	2.067547E+03	1.844270E-01	1.856290E-01
FE	6.894941E+02	3.530720E-02	3.553731E-02
co3--		0.000000E+00	0.000000E+00
so4--		0.000000E+00	0.000000E+00
s--		0.000000E+00	0.000000E+00

warning-- co3--, so4--, and s-- totals require that routine comp1
have the names of non-carbonate carbon, sulfide sulfur,
and non-sulfate sulfur aqueous species

single ion activities and activity coefficients are here defined
with respect to the internal ph scale

	ph	eh	pe
internal ph scale	4.7297	1.3479	5.7909E+00
modified nbs ph scale	4.4083	1.4227	6.1123E+00
rational ph scale	4.4083	1.4227	6.1123E+00

phcl = 6.1304

oxygen fugacity = 2.48067E-10
log oxygen fugacity = -9.60543

activity of water = 0.99667
log activity of water = -0.00145
alkalinity = 0.000000E+00 equiv/kg solvent
(not def. for t.gt.50 c)

ionic strength = 5.320770E+00 molal
sum of molalities = 37.1459468390593
osmotic coefficient = 0.00498
equiv. stoich. ionic strength = 9.935247E-02 molal

mass of solution = 2.878418 kg
mass of solvent = 1.006517 kg
mass of solutes = 1.871901 kg
conc of solutes = 65.032271 per cent (w/w)

species	moles	grams	conc	log conc	log g	log act
H2O	5.58705E+01	1.00652E+03				
NA+	3.22052E-01	7.40391E+00	3.19967E-01	-0.49490	-0.32144	-0.81633
K+	1.47452E+00	5.76514E+01	1.46498E+00	0.16583	-0.32144	-0.15561
CA++	1.60468E-04	6.43157E-03	1.59429E-04	-3.79743	-1.28575	-5.08318
MG++	1.15028E-05	2.79576E-04	1.14283E-05	-4.94202	-1.28575	-6.22777
AL+++	5.72236E-16	1.54398E-14	5.68530E-16	-15.24525	-2.89294	-18.13819
SIO2(AQ)	5.13533E-01	3.08553E+01	5.10208E-01	-0.29225	0.00000	-0.29225
H+	3.93107E-05	3.96213E-05	3.90562E-05	-4.40831	-0.32144	-4.72975
CO3--	2.15075E-01	1.29065E+01	2.13682E-01	-0.67023	-1.28575	-1.95598
CL-	8.38731E-02	2.97355E+00	8.33300E-02	-1.07920	-0.32144	-1.40064
SO4--	2.83908E-04	2.72715E-02	2.82069E-04	-3.54964	-1.28575	-4.83540
FE++	1.61451E-07	9.01654E-06	1.60405E-07	-6.79478	-1.28575	-8.08053
O2(AQ)	1.87150E-16	5.98859E-15	1.85939E-16	-15.73063	0.00000	-15.73063

H2(AQ)	1.31474E-02	2.65024E-02	1.30622E-02	-1.88398	0.00000	-1.88398
CH4(AQ)	5.09865E-02	8.17956E-01	5.06564E-02	-1.29537	0.00000	-1.29537
HS-	3.94254E-02	1.30372E+00	3.91702E-02	-1.40704	-0.32144	-1.72848
FE+++	2.73808E-14	1.52914E-12	2.72035E-14	-13.56537	-2.89294	-16.45831
HCO3-	4.93093E-01	3.00871E+01	4.89900E-01	-0.30989	-0.32144	-0.63133
CLO4-	1.62878E-42	1.61983E-40	1.61823E-42	-41.79096	-0.32144	-42.11240
OH-	5.27180E-01	8.96591E+00	5.23766E-01	-0.28086	-0.32144	-0.60230
HCOO-	2.49197E+00	1.12183E+02	2.47584E+00	0.39372	-0.32144	0.07228
CH3COO-	2.91146E-02	1.71906E+00	2.89261E-02	-1.53871	-0.32144	-1.86015
CH3CH2COO-	4.88177E-01	3.56718E+01	4.85016E-01	-0.31424	-0.32144	-0.63568
CO(AQ)	6.41957E-02	1.79815E+00	6.37800E-02	-1.19532	0.00000	-1.19532
ETHANE(AQ)	2.17181E-04	6.53051E-03	2.15775E-04	-3.66600	0.00000	-3.66600
ETHYLENE(AQ)	4.70604E-07	1.32021E-05	4.67556E-07	-6.33017	0.00000	-6.33017
PROPANE(AQ)	1.24191E-06	5.47634E-05	1.23387E-06	-5.90873	0.00000	-5.90873
HEXANE(AQ)	3.01780E-13	2.60064E-11	2.99826E-13	-12.52313	0.00000	-12.52313
BENZENE(AQ)	1.00028E-12	7.81354E-11	9.93805E-13	-12.00270	0.00000	-12.00270
TOLUENE(AQ)	4.03728E-15	3.71996E-13	4.01114E-15	-14.39673	0.00000	-14.39673
SI2O4(AQ)	3.54581E-02	4.26095E+00	3.52285E-02	-1.45311	0.00000	-1.45311
ALO2-	7.90899E-03	4.66475E-01	7.85778E-03	-2.10470	-0.32144	-2.42614
ALO2(SIO2)-	1.28786E-01	1.53338E+01	1.27952E-01	-0.89295	-0.32144	-1.21439
CACL+	2.37442E-04	1.79347E-02	2.35904E-04	-3.62726	-0.32144	-3.94870
CACL2(AQ)	4.00996E-07	4.45049E-05	3.98399E-07	-6.39968	0.00000	-6.39968
CACO3(AQ)	5.59499E-03	5.59998E-01	5.55877E-03	-2.25502	0.00000	-2.25502
CA(HCO3)+	3.17243E+00	3.20723E+02	3.15188E+00	0.49857	-0.24549	0.25308
CA(OH)+	4.17912E-04	2.38575E-02	4.15206E-04	-3.38174	-0.32144	-3.70317
CA(HSIO3)+	7.03244E-02	8.24002E+00	6.98690E-02	-1.15572	-0.32144	-1.47715
FECL+	9.27552E-08	8.46855E-06	9.21546E-08	-7.03548	-0.32144	-7.35692
FECL2(AQ)	7.56429E-05	9.58797E-03	7.51531E-05	-4.12405	0.00000	-4.12405
FE(HSIO3)+	3.54614E-02	4.71419E+00	3.52318E-02	-1.45307	-0.32144	-1.77450
KCL(AQ)	1.44531E-02	1.07750E+00	1.43595E-02	-1.84286	0.00000	-1.84286
KOH	2.11011E-01	1.18389E+01	2.09645E-01	-0.67852	0.00000	-0.67852
KSO4-	1.21836E-05	1.64668E-03	1.21047E-05	-4.91705	-0.32144	-5.23849
MGCL+	4.81758E-06	2.87889E-04	4.78639E-06	-5.31999	-0.32144	-5.64143
MG(HCO3)+	6.22220E-04	5.30891E-02	6.18191E-04	-3.20888	-0.32144	-3.53032
MG(HSIO3)+	6.34631E-02	6.43494E+00	6.30521E-02	-1.20030	-0.32144	-1.52174
MGSO4(AQ)	8.68078E-09	1.04484E-06	8.62457E-09	-8.06426	0.00000	-8.06426
NACL(AQ)	1.00470E-03	5.87172E-02	9.98191E-04	-3.00079	0.00000	-3.00079
NACO3-	6.41152E-04	5.32150E-02	6.37000E-04	-3.19586	-0.32144	-3.51730
NAHCO3(AQ)	8.48461E-03	7.12766E-01	8.42967E-03	-2.07419	0.00000	-2.07419
NAHSIO3(AQ)	3.55564E-04	3.55853E-02	3.53262E-04	-3.45190	0.00000	-3.45190
NAOH(AQ)	3.74618E-02	1.49836E+00	3.72192E-02	-1.42923	0.00000	-1.42923
HSIO3-	4.17161E-01	3.21596E+01	4.14460E-01	-0.38252	-0.32144	-0.70396
HSO4-	1.85332E-03	1.79893E-01	1.84132E-03	-2.73487	-0.32144	-3.05631
H2S(AQ)	1.44054E-01	4.90876E+00	1.43121E-01	-0.84430	0.00000	-0.84430
CO2(AQ)	2.62185E+01	1.15387E+03	2.60488E+01	1.41579	0.00000	1.41579
HCL(AQ)	2.74697E-04	1.00157E-02	2.72918E-04	-3.56397	0.00000	-3.56397
CH3CH2COOH	5.21227E-05	3.86121E-03	5.17852E-05	-4.28579	0.00000	-4.28579
CH3COOH	1.69958E-03	1.02064E-01	1.68858E-03	-2.77248	0.00000	-2.77248
HCOOH	3.15362E-03	1.45147E-01	3.13320E-03	-2.50401	0.00000	-2.50401

--- activity ratios of cations ---

log (NA+ /h+**0)	3.9134153
log (K+ /h+**0)	4.5741408
log (CA++ /h+**0)	4.3763135
log (MG++ /h+**0)	3.2317288
log (AL+++ /h+**0)	-3.9489415
log (FE++ /h+**0)	1.3789638
log (FE+++ /h+**0)	-2.2690696

--- summary of solid product phases---

product	log moles	moles	grams	volume, cc
DIAMOND	-1.2152228	6.09224E-02	7.31739E-01	2.08355E-01
CALCITE(SS)	-0.9608066	1.09444E-01	1.01746E+01	3.25917E+00
CALCITE	-1.7346718	1.84216E-02	1.84381E+00	6.79758E-01
MAGNESITE	-1.1535565	7.02172E-02	5.92031E+00	1.96819E+00
SIDERITE	-1.6818215	2.08055E-02	2.41045E+00	6.11225E-01

--- grand summary of solid phases (e.s.+p.r.s.+reactants) ---

phase/end-member	log moles	moles	grams	volume, cc
DIAMOND	-1.2152228	6.09224E-02	7.31739E-01	2.08355E-01
COESITE	1.2988531	1.99000E+01	1.19568E+03	4.51491E+02
CLINOPYROXENE(SS)	1.2988531	1.99000E+01		
DIOPSIDE	0.5998831	3.98000E+00	8.61879E+02	2.63476E+02
HEDENBERGITE	0.2988531	1.99000E+00	4.93708E+02	1.31738E+02
JADEITE	1.1439511	1.39300E+01	2.81579E+03	8.40536E+02
GARNET(SS)	1.2988531	1.99000E+01		
PYROPE	1.0770043	1.19400E+01	1.60452E+03	1.35113E+03
ALMANDINE	0.7759743	5.97000E+00	9.90755E+02	6.75565E+02
GROSSULAR	0.2988531	1.99000E+00	2.98844E+02	2.49506E+02
CALCITE(SS)	-0.9608066	1.09444E-01		
CALCITE	-1.7346718	1.84216E-02	1.84381E+00	6.79758E-01
MAGNESITE	-1.1535565	7.02172E-02	5.92031E+00	1.96819E+00
SIDERITE	-1.6818215	2.08055E-02	2.41045E+00	6.11225E-01

	mass, grams	volume, cc
created	1.090630E+01	3.467526E+00
destroyed	4.151346E+01	1.991680E+01
net	-3.060716E+01	-1.644927E+01

warning-- these volume totals may be incomplete because
of missing partial molar volume data in the data base

--- mineral saturation state summary ---

mineral	affinity, kcal	state	mineral	affinity, kcal	state
DIAMOND	0.0000	satd	CORUNDUM	-8.8125	
BRUCITE	-7.4615		DIASPORE	-4.6834	
CALCITE	-4.1542		ARAGONITE	-2.4300	
MAGNESITE	-1.0347		DOLOMITE	-3.9526	
KYANITE	-9.0286		GROSSULAR	-7.1635	
FORSTERITE	-9.7297		ENSTATITE-CL	-4.2659	
ENSTATITE-OR	-4.1709		ENSTATITE-PR	-5.3707	
DIOPSIDE	-7.6440		FERROSILITE	-7.8339	
PYROPE	-5.1437		ALMANDINE	-6.3156	
COESITE	-2.9383		GRAPHITE	-0.3430	
PYRRHOTITE	-2.8052		PYRITE	-3.6370	
FERROUS_OXIDE	-5.5388		GIBBSITE	-8.8455	
SEPIOLITE	1036.1432	ssatd	SIDERITE	-3.8705	
QUARTZ-ALPHA	-3.5765		QUARTZ-BETA	-4.6545	
CRISTOBALITE-ALPHA	-8.3789		CHALCEDONY	-4.5310	
AMORPHOUS_SILICA	-5.8698				

--- summary of solid solutions ---

mineral	aff. kcal/mol		mole frac.	lambda	state
ORTHOPYROXENE(SS)	-3.7307				
FERROSILITE		-3.73075	0.1720449	1.00000	
ENSTATITE-OR		-3.73075	0.8279551	1.00000	
OLIVINE	-9.3986				
FAYALITE		-9.39860	0.1324004	1.00000	
FORSTERITE		-9.39860	0.8675996	1.00000	
BIOTITE	-12.0162				
PHLOGOPITE		-12.01620	0.9950947	1.00000	

```
    ANNITE                    -12.01620    0.0049053     1.00000

CLINOPYROXENE(SS)        -6.6431
    DIOPSIDE                  -6.64313    0.6509531     1.00000
    HEDENBERGITE              -6.64313    0.1162478     1.00000
    JADEITE                   -6.64313    0.2327990     1.00000

GARNET(SS)               -3.4983
    PYROPE                    -3.49832    0.4937387     1.00000
    ALMANDINE                 -3.49832    0.2986617     1.00000
    GROSSULAR                 -3.49832    0.2075996     1.00000

CALCITE(SS)               0.0000                          saturated
    CALCITE                    0.00000    0.1683197     1.00000
    MAGNESITE                  0.00000    0.6415790     1.00000
    SIDERITE                   0.00000    0.1901013     1.00000


solid solution product phases

           xbar     lambda   activity  log xbar  log lambda log activity

  CALCITE(SS)
ideal solution

    CALCITE
            0.1683    1.0000    0.1683   -0.7739    0.0000   -0.7739
    MAGNESITE
            0.6416    1.0000    0.6416   -0.1927    0.0000   -0.1927
    SIDERITE
            0.1901    1.0000    0.1901   -0.7210    0.0000   -0.7210


          --- summary of gas species ---

          gas               log fugacity         fugacity         partial pressure

  CO2(G)                        9.10437       1.27166E+09
  O2(G)                        -9.60543       2.48067E-10
  S2(G)                         4.00001       1.00001E+04
  CH4(G)                        6.97463       9.43266E+06
  H2(G)                         3.81007       6.45754E+03
  H2S(G)                        7.27377       1.87832E+07
  H2O(G)                        6.90035       7.94972E+06



- - - - - - - - - - - - - - - - - - - - - - - - - - - - - - - -



stepping to zi=  1.3537E-01, delzi=  3.5369E-02, nord= 6
              ncycle=  0
steps completed =    49, iter = 18, ncorr = 0
most rapidly changing is zvclg1(SIDERITE          ) =     -1.5261

stepping to zi=  1.7510E-01, delzi=  3.9732E-02, nord= 6
              ncycle=  0
steps completed =    50, iter = 18, ncorr = 0
most rapidly changing is zvclg1(SIDERITE          ) =     -1.3962

stepping to zi=  2.2064E-01, delzi=  4.5540E-02, nord= 6
              ncycle=  0
steps completed =    51, iter = 18, ncorr = 0
most rapidly changing is zvclg1(SIDERITE          ) =     -1.2817

stepping to zi=  2.7410E-01, delzi=  5.3457E-02, nord= 6
              ncycle=  0
steps completed =    52, iter = 18, ncorr = 0
most rapidly changing is zvclg1(SIDERITE          ) =     -1.1763

stepping to zi=  3.1623E-01, delzi=  4.2131E-02, nord= 6
              ncycle=  0
steps completed =    53, iter = 18, ncorr = 0
most rapidly changing is zvclg1(SIDERITE          ) =     -1.1079
- - - - - - - - - - - - - - - - - - - - - - - - - - - - - - - -
```

reaction progress   = 3.16227766016835E-01
log of reaction progress =   -0.5000000

temperature   =   900.000 degrees c
total pressure = 50000.000 bars

computing units remaining =   0.000

step size is limited by the print requirement

--- reactant summary ---

reactant	moles	delta moles	grams	delta grams
CLINOPYROXENE(SS)	1.96838E+01	3.16228E-01	4.12605E+03	6.62867E+01
GARNET(SS)	1.96838E+01	3.16228E-01	2.86267E+03	4.59900E+01
COESITE	1.96838E+01	3.16228E-01	1.18269E+03	1.90003E+01

current total mass = 8.17141E+03 grams
delta total mass   = 1.31277E+02 grams
delta total volume =   62.98245 cc

reactant	affinity	rel. rate
CLINOPYROXENE(SS)	4.2397	1.00000E+00
GARNET(SS)	1.3671	1.00000E+00
COESITE	2.0528	1.00000E+00

affinity of the overall irreversible reaction=   7.660 kcal
contributions from irreversible reactions
with no thermodynamic data are not included

--- element totals for the aqueous phase ---

element	mg/kg soln.	molal conc.	moles
O	7.184606E+05	1.313993E+02	1.322149E+02
NA	4.070902E+03	5.181433E-01	5.213594E-01
K	2.257488E+04	1.689513E+00	1.700000E+00
CA	4.489677E+04	3.277791E+00	3.298137E+00
MG	7.797305E+02	9.387345E-02	9.445613E-02
AL	3.959419E+03	4.293966E-01	4.320620E-01
SI	2.065099E+04	2.151556E+00	2.164911E+00
H	4.189119E+04	1.216184E+02	1.223733E+02
C	1.382592E+05	3.368285E+01	3.389192E+01
CL	1.204125E+03	9.938312E-02	1.000000E-01
S	2.021287E+03	1.844839E-01	1.856290E-01
FE	1.230911E+03	6.449418E-02	6.489450E-02
co3--		0.000000E+00	0.000000E+00
so4--		0.000000E+00	0.000000E+00
s--		0.000000E+00	0.000000E+00

warning-- co3--, so4--, and s-- totals require that routine comp1
have the names of non-carbonate carbon, sulfide sulfur,
and non-sulfate sulfur aqueous species

single ion activities and activity coefficients are here defined
with respect to the internal ph scale

ph   eh   pe

internal ph scale	4.7100	1.3524	5.8100E+00
modified nbs ph scale	4.3869	1.4276	6.1331E+00
rational ph scale	4.3869	1.4276	6.1331E+00

phcl = 6.1141

oxygen fugacity = 2.46543E-10
log oxygen fugacity = -9.60811

activity of water = 0.99667
log activity of water = -0.00145
alkalinity = 0.000000E+00 equiv/kg solvent
(not def. for t.gt.50 c)

ionic strength = 5.556300E+00 molal
sum of molalities = 37.7924498015065
osmotic coefficient = 0.00490
equiv. stoich. ionic strength = 9.938312E-02 molal

mass of solution = 2.944295 kg
mass of solvent = 1.006207 kg
mass of solutes = 1.938087 kg
conc of solutes = 65.825189 per cent (w/w)

species	moles	grams	conc	log conc	log g	log act
H2O	5.58532E+01	1.00621E+03				
NA+	4.56504E-01	1.04949E+01	4.53688E-01	-0.34324	-0.32308	-0.66632
K+	1.48354E+00	5.80038E+01	1.47439E+00	0.16861	-0.32308	-0.15447
CA++	1.72013E-04	6.89427E-03	1.70952E-04	-3.76713	-1.29231	-5.05944
MG++	1.23114E-05	2.99229E-04	1.22355E-05	-4.91238	-1.29231	-6.20469
AL+++	1.55850E-15	4.20508E-14	1.54889E-15	-14.80998	-2.90770	-17.71768
SIO2(AQ)	7.50556E-01	4.50966E+01	7.45926E-01	-0.12730	0.00000	-0.12730
H+	4.12863E-05	4.16124E-05	4.10316E-05	-4.38688	-0.32308	-4.70996
CO3--	1.98044E-01	1.18844E+01	1.96822E-01	-0.70593	-1.29231	-1.99824
CL-	8.34877E-02	2.95989E+00	8.29727E-02	-1.08106	-0.32308	-1.40414
SO4--	2.62354E-04	2.52011E-02	2.60735E-04	-3.58380	-1.29231	-4.87611
FE++	2.13599E-07	1.19289E-05	2.12281E-07	-6.67309	-1.29231	-7.96540
O2(AQ)	1.85943E-16	5.94996E-15	1.84796E-16	-15.73331	0.00000	-15.73331
H2(AQ)	1.31838E-02	2.65760E-02	1.31025E-02	-1.88265	0.00000	-1.88265
CH4(AQ)	5.12858E-02	8.22757E-01	5.09694E-02	-1.29269	0.00000	-1.29269
HS-	3.81689E-02	1.26216E+00	3.79334E-02	-1.42098	-0.32308	-1.74406
FE+++	3.85767E-14	2.15439E-12	3.83387E-14	-13.41636	-2.90770	-16.32406
HCO3-	4.69860E-01	2.86695E+01	4.66962E-01	-0.33072	-0.32308	-0.65380
CLO4-	1.60143E-42	1.59263E-40	1.59155E-42	-41.79818	-0.32308	-42.12126
OH-	5.05447E-01	8.59629E+00	5.02329E-01	-0.29901	-0.32308	-0.62209
HCOO-	2.38189E+00	1.07227E+02	2.36720E+00	0.37423	-0.32308	0.05116
CH3COO-	2.79143E-02	1.64819E+00	2.77421E-02	-1.55686	-0.32308	-1.87994
CH3CH2COO-	4.69496E-01	3.43067E+01	4.66600E-01	-0.33106	-0.32308	-0.65413
CO(AQ)	6.39785E-02	1.79206E+00	6.35838E-02	-1.19665	0.00000	-1.19665
ETHANE(AQ)	2.19130E-04	6.58911E-03	2.17778E-04	-3.66199	0.00000	-3.66199
ETHYLENE(AQ)	4.73366E-07	1.32796E-05	4.70446E-07	-6.32749	0.00000	-6.32749
PROPANE(AQ)	1.25692E-06	5.54253E-05	1.24916E-06	-5.90338	0.00000	-5.90338
HEXANE(AQ)	3.08263E-13	2.65650E-11	3.06361E-13	-12.51377	0.00000	-12.51377
BENZENE(AQ)	1.00926E-12	7.88365E-11	1.00303E-12	-11.99869	0.00000	-11.99869
TOLUENE(AQ)	4.08608E-15	3.76492E-13	4.06087E-15	-14.39138	0.00000	-14.39138
SI2O4(AQ)	7.57668E-02	9.10479E+00	7.52994E-02	-1.12321	0.00000	-1.12321
ALO2-	1.74173E-02	1.02728E+00	1.73098E-02	-1.76171	-0.32308	-2.08478
ALO2(SIO2)-	4.14645E-01	4.93695E+01	4.12087E-01	-0.38501	-0.32308	-0.70809
CACL+	2.49634E-04	1.88556E-02	2.48094E-04	-3.60538	-0.32308	-3.92846
CACL2(AQ)	4.16620E-07	4.62390E-05	4.14050E-07	-6.38295	0.00000	-6.38295
CACO3(AQ)	5.35991E-03	5.36469E-01	5.32684E-03	-2.27353	0.00000	-2.27353
CA(HCO3)+	3.18782E+00	3.22279E+02	3.16815E+00	0.50081	-0.24645	0.25436
CA(OH)+	4.23202E-04	2.41595E-02	4.20591E-04	-3.37614	-0.32308	-3.69922
CA(HSIO3)+	1.04116E-01	1.21994E+01	1.03474E-01	-0.98517	-0.32308	-1.30825
FECL+	1.20357E-07	1.09886E-05	1.19615E-07	-6.92221	-0.32308	-7.24529
FECL2(AQ)	9.69967E-05	1.22946E-02	9.63983E-05	-4.01593	0.00000	-4.01593
FE(HSIO3)+	6.47972E-02	8.61405E+00	6.43974E-02	-1.19113	-0.32308	-1.51421
KCL(AQ)	1.43702E-02	1.07131E+00	1.42815E-02	-1.84523	0.00000	-1.84523
KOH	2.02080E-01	1.13378E+01	2.00833E-01	-0.69716	0.00000	-0.69716
KSO4-	1.11611E-05	1.50849E-03	1.10922E-05	-4.95498	-0.32308	-5.27806

MGCL+	5.05717E-06	3.02207E-04	5.02598E-06	-5.29878	-0.32308	-5.62186
MG(HCO3)+	6.25263E-04	5.33487E-02	6.21406E-04	-3.20662	-0.32308	-3.52970
MG(HSIO3)+	9.38135E-02	9.51237E+00	9.32348E-02	-1.03042	-0.32308	-1.35350
MGSO4(AQ)	8.33275E-09	1.00295E-06	8.28135E-09	-8.08190	0.00000	-8.08190
NACL(AQ)	1.40737E-03	8.22504E-02	1.39868E-03	-2.85428	0.00000	-2.85428
NACO3-	8.24565E-04	6.84381E-02	8.19479E-04	-3.08646	-0.32308	-3.40954
NAHCO3(AQ)	1.13774E-02	9.55783E-01	1.13073E-02	-1.94664	0.00000	-1.94664
NAHSIO3(AQ)	7.01384E-04	7.01955E-02	6.97057E-04	-3.15673	0.00000	-3.15673
NAOH(AQ)	5.05449E-02	2.02165E+00	5.02331E-02	-1.29901	0.00000	-1.29901
HSIO3-	5.84749E-01	4.50792E+01	5.81142E-01	-0.23572	-0.32308	-0.55880
HSO4-	1.77227E-03	1.72026E-01	1.76134E-03	-2.75416	-0.32308	-3.07723
H2S(AQ)	1.45414E-01	4.95511E+00	1.44517E-01	-0.84008	0.00000	-0.84008
CO2(AQ)	2.60494E+01	1.14643E+03	2.58887E+01	1.41311	0.00000	1.41311
HCL(AQ)	2.85104E-04	1.03951E-02	2.83345E-04	-3.54768	0.00000	-3.54768
CH3CH2COOH	5.22672E-05	3.87192E-03	5.19448E-05	-4.28446	0.00000	-4.28446
CH3COOH	1.69906E-03	1.02032E-01	1.68857E-03	-2.77248	0.00000	-2.77248
HCOOH	3.14295E-03	1.44656E-01	3.12356E-03	-2.50535	0.00000	-2.50535

--- activity ratios of cations ---

log (NA+ /h+**0)	4.0436389
log (K+ /h+**0)	4.5554934
log (CA++ /h+**0)	4.3604821
log (MG++ /h+**0)	3.2152301
log (AL+++ /h+**0)	-3.5877988
log (FE++ /h+**0)	1.4545209
log (FE+++ /h+**0)	-2.1941815

--- summary of solid product phases---

product	log moles	moles	grams	volume, cc
DIAMOND	-0.7374726	1.83032E-01	2.19840E+00	6.25970E-01
CALCITE(SS)	-0.4597466	3.46939E-01	3.25951E+01	1.03262E+01
CALCITE	-1.2521202	5.59603E-02	5.60102E+00	2.06493E+00
MAGNESITE	-0.6716721	2.12975E-01	1.79568E+01	5.96968E+00
SIDERITE	-1.1078814	7.80043E-02	9.03728E+00	2.29161E+00

--- grand summary of solid phases (e.s.+p.r.s.+reactants) ---

phase/end-member	log moles	moles	grams	volume, cc
DIAMOND	-0.7374726	1.83032E-01	2.19840E+00	6.25970E-01
COESITE	1.2941083	1.96838E+01	1.18269E+03	4.46585E+02
CLINOPYROXENE(SS)	1.2941083	1.96838E+01		
DIOPSIDE	0.5951383	3.93675E+00	8.52514E+02	2.60613E+02
HEDENBERGITE	0.2941083	1.96838E+00	4.88343E+02	1.30307E+02
JADEITE	1.1392064	1.37786E+01	2.78520E+03	8.31403E+02
GARNET(SS)	1.2941083	1.96838E+01		
PYROPE	1.0722596	1.18103E+01	1.58709E+03	1.33645E+03
ALMANDINE	0.7712296	5.90513E+00	9.79990E+02	6.68225E+02
GROSSULAR	0.2941083	1.96838E+00	2.95597E+02	2.46795E+02
CALCITE(SS)	-0.4597466	3.46939E-01		
CALCITE	-1.2521202	5.59603E-02	5.60102E+00	2.06493E+00
MAGNESITE	-0.6716721	2.12975E-01	1.79568E+01	5.96968E+00
SIDERITE	-1.1078814	7.80043E-02	9.03728E+00	2.29161E+00

	mass, grams	volume, cc
created	3.479349E+01	1.095219E+01
destroyed	1.312771E+02	6.298245E+01

```
          net            -9.648359E+01      -5.203026E+01

         warning-- these volume totals may be incomplete because
         of missing partial molar volume data in the data base



          --- mineral saturation state summary ---


  mineral        affinity, kcal    state        mineral        affinity, kcal    state

DIAMOND                0.0000  satd         CORUNDUM               -4.9352
BRUCITE               -7.5501               DIASPORE               -2.7447
CALCITE               -4.2536               ARAGONITE              -2.5293
MAGNESITE             -1.1376               DOLOMITE               -4.1549
KYANITE               -4.2658               SILLIMANITE            -8.8684
GROSSULAR             -5.0720               LAWSONITE              -6.5422
FORSTERITE            -9.0214               ENSTATITE-CL           -3.4690
ENSTATITE-OR          -3.3740               ENSTATITE-PR           -4.5738
DIOPSIDE              -6.0466               HEDENBERGITE           -9.5687
JADEITE               -5.6326               FERROSILITE            -6.5429
MUSCOVITE             -8.6111               PHLOGOPITE             -7.7984
PYROPE                -3.0557               ALMANDINE              -3.7330
COESITE               -2.0528               GRAPHITE               -0.3430
PYRRHOTITE            -2.3770               PYRITE                 -3.1933
FERROUS_OXIDE         -5.1332               GIBBSITE               -6.9069
SEPIOLITE           1038.6225  ssatd        SIDERITE               -3.4793
QUARTZ-ALPHA          -2.6911               QUARTZ-BETA            -3.7690
CRISTOBALITE-ALPHA    -7.4934               CRISTOBALITE-BETA      -9.5628
CHALCEDONY            -3.6455               AMORPHOUS_SILICA       -4.9843


                    --- summary of solid solutions  ---

mineral               aff. kcal/mol  mole frac.    lambda      state

ORTHOPYROXENE(SS)        -2.8410
    FERROSILITE               -2.84102    0.2043643     1.00000
    ENSTATITE-OR              -2.84102    0.7956357     1.00000

OLIVINE                  -8.5328
    FAYALITE                  -8.53275    0.1890849     1.00000
    FORSTERITE                -8.53275    0.8109151     1.00000

BIOTITE                  -7.7768
    PHLOGOPITE                -7.77680    0.9907754     1.00000
    ANNITE                    -7.77680    0.0092246     1.00000

CLINOPYROXENE(SS)        -3.9910
    DIOPSIDE                  -3.99101    0.4140642     1.00000
    HEDENBERGITE              -3.99101    0.0914026     1.00000
    JADEITE                   -3.99101    0.4945333     1.00000

GARNET(SS)               -1.2506
    PYROPE                    -1.25063    0.4610444     1.00000
    ALMANDINE                 -1.25063    0.3448044     1.00000
    GROSSULAR                 -1.25063    0.1941512     1.00000

CALCITE(SS)               0.0000                               saturated
    CALCITE                    0.00000    0.1612971     1.00000
    MAGNESITE                  0.00000    0.6138673     1.00000
    SIDERITE                   0.00000    0.2248357     1.00000


solid solution product phases

           xbar     lambda   activity  log xbar  log lambda log activity

  CALCITE(SS)
ideal solution

    CALCITE
            0.1613    1.0000    0.1613   -0.7924    0.0000   -0.7924
    MAGNESITE
            0.6139    1.0000    0.6139   -0.2119    0.0000   -0.2119
```

```
    SIDERITE
            0.2248    1.0000    0.2248   -0.6481    0.0000   -0.6481


          --- summary of gas species ---

          gas                log fugacity         fugacity         partial pressure

  CO2(G)                        9.10169       1.26384E+09
  O2(G)                        -9.60811       2.46543E-10
  S2(G)                         4.00576       1.01335E+04
  CH4(G)                        6.97731       9.49095E+06
  H2(G)                         3.81140       6.47746E+03
  H2S(G)                        7.27798       1.89664E+07
  H2O(G)                        6.90035       7.94971E+06



- - - - - - - - - - - - - - - - - - - - - - - - - - - - - - - - -



stepping to zi=  3.9194E-01, delzi=  7.5708E-02, nord= 6
              ncycle=  0
steps completed =    54, iter = 18, ncorr = 0
most rapidly changing is zvclg1(SIDERITE          ) =     -1.0068

stepping to zi=  4.8235E-01, delzi=  9.0412E-02, nord= 6
              ncycle=  0
steps completed =    55, iter = 18, ncorr = 0
most rapidly changing is zvclg1(SIDERITE          ) =     -0.9107

stepping to zi=  5.6836E-01, delzi=  8.6016E-02, nord= 6
              ncycle=  0
iter =   14
      0 supersaturated pure minerals
      1 supersaturated solid solutions

                         the most supersaturated phases     affinity, kcal

                           1   51000   GARNET(SS)             0.01109699
--- go back to reduce the extent of supersaturation ---
--- cutting step size and trying again ---

stepping to zi=  5.0385E-01, delzi=  2.1504E-02, nord= 6
              ncycle=  0
steps completed =    56, iter = 17, ncorr = 0
most rapidly changing is zvclg1(SIDERITE          ) =     -0.8907

stepping to zi=  5.4686E-01, delzi=  4.3008E-02, nord= 3
              ncycle=  0
steps completed =    57, iter = 17, ncorr = 0
most rapidly changing is zvclg1(SIDERITE          ) =     -0.8534

stepping to zi=  5.6834E-01, delzi=  2.1477E-02, nord= 4
              ncycle=  0
iter =   15
      0 supersaturated pure minerals
      1 supersaturated solid solutions

                         the most supersaturated phases     affinity, kcal

                           1   51000   GARNET(SS)             0.01099223
--- go back to reduce the extent of supersaturation ---
--- cutting step size and trying again ---

stepping to zi=  5.5223E-01, delzi=  5.3693E-03, nord= 4
              ncycle=  0
steps completed =    58, iter = 16, ncorr = 0
most rapidly changing is zvclg1(SIDERITE          ) =     -0.8490

stepping to zi=  5.6297E-01, delzi=  1.0739E-02, nord= 2
              ncycle=  0
steps completed =    59, iter = 16, ncorr = 0
most rapidly changing is zvclg1(SIDERITE          ) =     -0.8403
```

```
 stepping to zi=  5.6747E-01, delzi=  4.5058E-03, nord= 3
              ncycle=  0
 iter =   14
       0 supersaturated pure minerals
       1 supersaturated solid solutions

                         the most supersaturated phases     affinity, kcal

                           1   51000   GARNET(SS)              0.00760535


   attempted species assemblage no.   2

              1      1  H2O
              2      2  NA+
              3      3  K+
              4      4  CA++
              5      5  MG++
              6      6  AL+++
              7      7  SIO2(AQ)
              8     13  H+
              9     14  CO3--
             10     16  CL-
             11     17  SO4--
             12     21  FE++
             13     29  O2(G)
             14      2  DIAMOND
             15      1  GARNET(SPYROPE
             16      2  GARNET(SALMANDINE
             17      3  GARNET(SGROSSULAR
             18      1  CALCITE(CALCITE
             19      2  CALCITE(MAGNESITE
             20      3  CALCITE(SIDERITE

 * note- reactant GARNET(SS)               has saturated but differs        in composition from the corresponding product.  it
must        remain in the reactant subsystem (rsatch)
 steps completed =    60, iter = 17, ncorr = 0
 - - - - - - - - - - - - - - - - - - - - - - - - - - - - - - - -



                    reaction progress        =  5.67473069524742E-01
                    log of reaction progress =      -0.2460547

                    temperature    =    900.000 degrees c
                    total pressure =  50000.000 bars

                    computing units remaining =      0.000


 change in the product phase assemblage


                              --- reactant summary ---



        reactant            moles       delta moles      grams        delta grams

   CLINOPYROXENE(SS)       1.94325E+01    5.67473E-01    4.07339E+03    1.18952E+02
   GARNET(SS)              1.94325E+01    5.67473E-01    2.82614E+03    8.25295E+01
   COESITE                 1.94325E+01    5.67473E-01    1.16759E+03    3.40962E+01


               current total mass =  8.06711E+03 grams
               delta total mass   =  2.35578E+02 grams
               delta total volume =    113.02248 cc


        reactant           affinity     rel. rate

   CLINOPYROXENE(SS)          1.9069    1.00000E+00
   GARNET(SS)                 0.1161    0.00000E+00
   COESITE                    1.3552    1.00000E+00

         affinity of the overall irreversible reaction=     3.262 kcal
```

contributions from irreversible reactions
with no thermodynamic data are not included

--- element totals for the aqueous phase ---

element	mg/kg soln.	molal conc.	moles
O	7.117773E+05	1.336636E+02	1.344186E+02
NA	5.305087E+03	6.933150E-01	6.972312E-01
K	2.199824E+04	1.690452E+00	1.700000E+00
CA	4.456101E+04	3.340412E+00	3.359280E+00
MG	1.076892E+03	1.331218E-01	1.338738E-01
AL	6.899152E+03	7.682484E-01	7.725878E-01
SI	2.942775E+04	3.148094E+00	3.165875E+00
H	4.082114E+04	1.216859E+02	1.223733E+02
C	1.331886E+05	3.331658E+01	3.350477E+01
CL	1.173368E+03	9.943833E-02	1.000000E-01
S	1.969656E+03	1.845863E-01	1.856290E-01
FE	1.801823E+03	9.693600E-02	9.748353E-02
co3--		0.000000E+00	0.000000E+00
so4--		0.000000E+00	0.000000E+00
s--		0.000000E+00	0.000000E+00

warning-- co3--, so4--, and s-- totals require that routine comp1
have the names of non-carbonate carbon, sulfide sulfur,
and non-sulfate sulfur aqueous species

single ion activities and activity coefficients are here defined
with respect to the internal ph scale

	ph	eh	pe
internal ph scale	4.6892	1.3570	5.8299E+00
modified nbs ph scale	4.3643	1.4327	6.1549E+00
rational ph scale	4.3643	1.4327	6.1549E+00

phcl = 6.0972

oxygen fugacity = 2.44748E-10
log oxygen fugacity = -9.61128

activity of water = 0.99667
log activity of water = -0.00145
alkalinity = 0.000000E+00 equiv/kg solvent
(not def. for t.gt.50 c)

ionic strength = 5.841660E+00 molal
sum of molalities = 38.5553948826778
osmotic coefficient = 0.00481
equiv. stoich. ionic strength = 9.943833E-02 molal

mass of solution = 3.021474 kg
mass of solvent = 1.005648 kg
mass of solutes = 2.015826 kg
conc of solutes = 66.716630 per cent (w/w)

species	moles	grams	conc	log conc	log g	log act
H2O	5.58222E+01	1.00565E+03				
NA+	6.14178E-01	1.41198E+01	6.10728E-01	-0.21415	-0.32495	-0.53910
K+	1.49271E+00	5.83624E+01	1.48433E+00	0.17153	-0.32495	-0.15342
CA++	1.85769E-04	7.44561E-03	1.84725E-04	-3.73347	-1.29980	-5.03327
MG++	1.37709E-05	3.34701E-04	1.36935E-05	-4.86348	-1.29980	-6.16328
AL+++	2.61629E-15	7.05915E-14	2.60159E-15	-14.58476	-2.92455	-17.50931
SIO2(AQ)	1.01181E+00	6.07940E+01	1.00613E+00	0.00265	0.00000	0.00265

H+	4.34697E-05	4.38131E-05	4.32256E-05	-4.36426	-0.32495	-4.68921
CO3--	1.81691E-01	1.09032E+01	1.80671E-01	-0.74311	-1.29980	-2.04291
CL-	8.30714E-02	2.94513E+00	8.26048E-02	-1.08299	-0.32495	-1.40794
SO4--	2.41345E-04	2.31830E-02	2.39989E-04	-3.61981	-1.29980	-4.91961
FE++	2.52865E-07	1.41218E-05	2.51445E-07	-6.59956	-1.29980	-7.89936
O2(AQ)	1.84487E-16	5.90335E-15	1.83450E-16	-15.73648	0.00000	-15.73648
H2(AQ)	1.32247E-02	2.66584E-02	1.31505E-02	-1.88106	0.00000	-1.88106
CH4(AQ)	5.16332E-02	8.28330E-01	5.13431E-02	-1.28952	0.00000	-1.28952
HS-	3.68927E-02	1.21996E+00	3.66855E-02	-1.43551	-0.32495	-1.76046
FE+++	4.88579E-14	2.72857E-12	4.85835E-14	-13.31351	-2.92455	-16.23806
HCO3-	4.46349E-01	2.72349E+01	4.43842E-01	-0.35277	-0.32495	-0.67772
CLO4-	1.57032E-42	1.56169E-40	1.56150E-42	-41.80646	-0.32495	-42.13141
OH-	4.83678E-01	8.22605E+00	4.80961E-01	-0.31789	-0.32495	-0.64284
HCOO-	2.27099E+00	1.02235E+02	2.25824E+00	0.35377	-0.32495	0.02882
CH3COO-	2.67120E-02	1.57720E+00	2.65620E-02	-1.57574	-0.32495	-1.90069
CH3CH2COO-	4.50918E-01	3.29492E+01	4.48386E-01	-0.34835	-0.32495	-0.67330
CO(AQ)	6.37097E-02	1.78453E+00	6.33519E-02	-1.19824	0.00000	-1.19824
ETHANE(AQ)	2.21422E-04	6.65801E-03	2.20178E-04	-3.65723	0.00000	-3.65723
ETHYLENE(AQ)	4.76572E-07	1.33696E-05	4.73895E-07	-6.32432	0.00000	-6.32432
PROPANE(AQ)	1.27471E-06	5.62098E-05	1.26755E-06	-5.89704	0.00000	-5.89704
HEXANE(AQ)	3.16071E-13	2.72379E-11	3.14296E-13	-12.50266	0.00000	-12.50266
BENZENE(AQ)	1.01981E-12	7.96609E-11	1.01408E-12	-11.99393	0.00000	-11.99393
TOLUENE(AQ)	4.14392E-15	3.81821E-13	4.12064E-15	-14.38504	0.00000	-14.38504
SI2O4(AQ)	1.37770E-01	1.65556E+01	1.36996E-01	-0.86329	0.00000	-0.86329
ALO2-	2.33333E-02	1.37621E+00	2.32022E-02	-1.63447	-0.32495	-1.95942
ALO2(SIO2)-	7.49254E-01	8.92097E+01	7.45046E-01	-0.12782	-0.32495	-0.45277
CACL+	2.63812E-04	1.99265E-02	2.62330E-04	-3.58115	-0.32495	-3.90610
CACL2(AQ)	4.34567E-07	4.82308E-05	4.32126E-07	-6.36439	0.00000	-6.36439
CACO3(AQ)	5.13338E-03	5.13796E-01	5.10455E-03	-2.29204	0.00000	-2.29204
CA(HCO3)+	3.21054E+00	3.24576E+02	3.19250E+00	0.50413	-0.24754	0.25660
CA(OH)+	4.30121E-04	2.45545E-02	4.27705E-04	-3.36886	-0.32495	-3.69381
CA(HSIO3)+	1.42731E-01	1.67240E+01	1.41929E-01	-0.84793	-0.32495	-1.17288
FECL+	1.39425E-07	1.27295E-05	1.38642E-07	-6.85810	-0.32495	-7.18305
FECL2(AQ)	1.10905E-04	1.40576E-02	1.10282E-04	-3.95749	0.00000	-3.95749
FE(HSIO3)+	9.73722E-02	1.29445E+01	9.68253E-02	-1.01401	-0.32495	-1.33896
KCL(AQ)	1.42713E-02	1.06394E+00	1.41911E-02	-1.84798	0.00000	-1.84798
KOH	1.93009E-01	1.08289E+01	1.91925E-01	-0.71687	0.00000	-0.71687
KSO4-	1.01598E-05	1.37315E-03	1.01027E-05	-4.99556	-0.32495	-5.32051
MGCL+	5.53529E-06	3.30778E-04	5.50420E-06	-5.25931	-0.32495	-5.58426
MG(HCO3)+	6.53390E-04	5.57486E-02	6.49721E-04	-3.18727	-0.32495	-3.51222
MG(HSIO3)+	1.33201E-01	1.35061E+01	1.32453E-01	-0.87794	-0.32495	-1.20289
MGSO4(AQ)	8.28805E-09	9.97572E-07	8.24150E-09	-8.08399	0.00000	-8.08399
NACL(AQ)	1.86888E-03	1.09223E-01	1.85838E-03	-2.73086	0.00000	-2.73086
NACO3-	1.00092E-03	8.30753E-02	9.95298E-04	-3.00205	-0.32495	-3.32700
NAHCO3(AQ)	1.44244E-02	1.21174E+00	1.43433E-02	-1.84335	0.00000	-1.84335
NAHSIO3(AQ)	1.20820E-03	1.20919E-01	1.20142E-03	-2.92031	0.00000	-2.92031
NAOH(AQ)	6.45511E-02	2.58186E+00	6.41886E-02	-1.19254	0.00000	-1.19254
HSIO3-	7.54758E-01	5.81855E+01	7.50519E-01	-0.12464	-0.32495	-0.44959
HSO4-	1.68816E-03	1.63862E-01	1.67867E-03	-2.77503	-0.32495	-3.09998
H2S(AQ)	1.46797E-01	5.00221E+00	1.45972E-01	-0.83573	0.00000	-0.83573
CO2(AQ)	2.58453E+01	1.13745E+03	2.57002E+01	1.40994	0.00000	1.40994
HCL(AQ)	2.96285E-04	1.08028E-02	2.94621E-04	-3.53074	0.00000	-3.53074
CH3CH2COOH	5.24292E-05	3.88391E-03	5.21347E-05	-4.28287	0.00000	-4.28287
CH3COOH	1.69811E-03	1.01975E-01	1.68857E-03	-2.77248	0.00000	-2.77248
HCOOH	3.12974E-03	1.44048E-01	3.11216E-03	-2.50694	0.00000	-2.50694

--- activity ratios of cations ---

log (NA+ /h+**0)	4.1501071
log (K+ /h+**0)	4.5357886
log (CA++ /h+**0)	4.3451446
log (MG++ /h+**0)	3.2151341
log (AL+++ /h+**0)	-3.4416830
log (FE++ /h+**0)	1.4790616
log (FE+++ /h+**0)	-2.1704340

--- summary of solid product phases---

product	log moles	moles	grams	volume, cc
DIAMOND	-0.5155353	3.05116E-01	3.66475E+00	1.04350E+00
GARNET(SS)	-2.3961094	4.01690E-03	5.97203E-01	4.63631E-01

PYROPE	-2.7381665	1.82740E-03	2.45570E-01	2.06789E-01
ALMANDINE	-2.8396750	1.44652E-03	2.40058E-01	1.63688E-01
GROSSULAR	-3.1290259	7.42975E-04	1.11575E-01	9.31542E-02
CALCITE(SS)	-0.2132402	6.12012E-01	5.76526E+01	1.81886E+01
CALCITE	-1.0241265	9.45962E-02	9.46805E+00	3.49060E+00
MAGNESITE	-0.4284370	3.72875E-01	3.14386E+01	1.04517E+01
SIDERITE	-0.8400095	1.44541E-01	1.67459E+01	4.24632E+00

--- grand summary of solid phases (e.s.+p.r.s.+reactants) ---

phase/end-member	log moles	moles	grams	volume, cc
DIAMOND	-0.5155353	3.05116E-01	3.66475E+00	1.04350E+00
COESITE	1.2885293	1.94325E+01	1.16759E+03	4.40885E+02
CLINOPYROXENE(SS)	1.2885293	1.94325E+01		
DIOPSIDE	0.5895593	3.88651E+00	8.41632E+02	2.57287E+02
HEDENBERGITE	0.2885293	1.94325E+00	4.82110E+02	1.28643E+02
JADEITE	1.1336273	1.36028E+01	2.74965E+03	8.20791E+02
GARNET(SS)	1.2886190	1.94365E+01		
PYROPE	1.0667486	1.16613E+01	1.56708E+03	1.31960E+03
ALMANDINE	0.7657583	5.83120E+00	9.67721E+02	6.59859E+02
GROSSULAR	0.2886953	1.94400E+00	2.91935E+02	2.43738E+02
CALCITE(SS)	-0.2132402	6.12012E-01		
CALCITE	-1.0241265	9.45962E-02	9.46805E+00	3.49060E+00
MAGNESITE	-0.4284370	3.72875E-01	3.14386E+01	1.04517E+01
SIDERITE	-0.8400095	1.44541E-01	1.67459E+01	4.24632E+00

	mass, grams	volume, cc
created	6.191459E+01	1.969573E+01
destroyed	2.355777E+02	1.130225E+02
net	-1.736631E+02	-9.332675E+01

warning-- these volume totals may be incomplete because
of missing partial molar volume data in the data base

--- mineral saturation state summary ---

mineral	affinity, kcal	state	mineral	affinity, kcal	state
DIAMOND	0.0000	satd	CORUNDUM	-3.3664	
SPINEL	-9.9253		BRUCITE	-7.5506	
DIASPORE	-1.9603		CALCITE	-4.3530	
ARAGONITE	-2.6287		MAGNESITE	-1.1552	
DOLOMITE	-4.2719		ANDALUSITE	-9.2835	
KYANITE	-1.9994		SILLIMANITE	-6.6021	
GROSSULAR	-3.9344		LAWSONITE	-3.6606	
FORSTERITE	-8.3248		ENSTATITE-CL	-2.7719	
ENSTATITE-OR	-2.6769		ENSTATITE-PR	-3.8767	
DIOPSIDE	-4.7342		HEDENBERGITE	-8.1241	
JADEITE	-2.8814		FERROSILITE	-5.7135	
K-FELDSPAR	-9.3177		MUSCOVITE	-4.2709	
PHLOGOPITE	-5.0285		PYROPE	-1.8362	
ALMANDINE	-2.3811		COESITE	-1.3552	
GRAPHITE	-0.3430		PYRRHOTITE	-2.2219	
PYRITE	-3.0234		FERROUS_OXIDE	-5.0014	
GIBBSITE	-6.1225		SEPIOLITE	1040.7143	ssatd
SIDERITE	-3.3646		QUARTZ-ALPHA	-1.9934	
QUARTZ-BETA	-3.0714		CRISTOBALITE-ALPHA	-6.7958	
CRISTOBALITE-BETA	-8.8652		CHALCEDONY	-2.9479	
AMORPHOUS_SILICA	-4.2867				

```
                    --- summary of solid solutions  ---

mineral               aff. kcal/mol  mole frac.    lambda      state

ORTHOPYROXENE(SS)        -2.1163
    FERROSILITE                -2.11626    0.2137430     1.00000
    ENSTATITE-OR               -2.11626    0.7862570     1.00000

OLIVINE                  -7.7838
    FAYALITE                   -7.78379    0.2070975     1.00000
    FORSTERITE                 -7.78379    0.7929025     1.00000

BIOTITE                  -5.0029
    PHLOGOPITE                 -5.00290    0.9890827     1.00000
    ANNITE                     -5.00290    0.0109173     1.00000

CLINOPYROXENE(SS)        -1.8489
    DIOPSIDE                   -1.84886    0.2900682     1.00000
    HEDENBERGITE               -1.84886    0.0677685     1.00000
    JADEITE                    -1.84886    0.6421634     1.00000

GARNET(SS)                0.0000                             saturated
    PYROPE                      0.00000    0.4549283     1.00000
    ALMANDINE                   0.00000    0.3601093     1.00000
    GROSSULAR                   0.00000    0.1849624     1.00000

CALCITE(SS)               0.0000                             saturated
    CALCITE                     0.00000    0.1545659     1.00000
    MAGNESITE                   0.00000    0.6092608     1.00000
    SIDERITE                    0.00000    0.2361733     1.00000


solid solution product phases

           xbar     lambda   activity  log xbar  log lambda log activity

  GARNET(SS)
ideal solution

    PYROPE
            0.4549    1.0000    0.4549   -0.3421    0.0000   -0.3421
    ALMANDINE
            0.3601    1.0000    0.3601   -0.4436    0.0000   -0.4436
    GROSSULAR
            0.1850    1.0000    0.1850   -0.7329    0.0000   -0.7329

  CALCITE(SS)
ideal solution

    CALCITE
            0.1546    1.0000    0.1546   -0.8109    0.0000   -0.8109
    MAGNESITE
            0.6093    1.0000    0.6093   -0.2152    0.0000   -0.2152
    SIDERITE
            0.2362    1.0000    0.2362   -0.6268    0.0000   -0.6268


          --- summary of gas species ---

          gas               log fugacity         fugacity         partial pressure

  CO2(G)                        9.09852       1.25464E+09
  O2(G)                        -9.61128       2.44748E-10
  S2(G)                         4.01129       1.02633E+04
  CH4(G)                        6.98048       9.56054E+06
  H2(G)                         3.81299       6.50117E+03
  H2S(G)                        7.28234       1.91573E+07
  H2O(G)                        6.90035       7.94970E+06


- - - - - - - - - - - - - - - - - - - - - - - - - - - - - - - -
```

```
 stepping to zi=  5.6747E-01, delzi=  1.0000E-08, nord= 0
              ncycle=  0
 * note- reactant GARNET(SS)               has saturated but differs        in composition from the corresponding product.  it
must        remain in the reactant subsystem (rsatch)
 steps completed =    61, iter =  6, ncorr = 0
 most rapidly changing is zvclg1(PYROPE           ) =     -2.7382

 stepping to zi=  5.6747E-01, delzi=  1.0000E-08, nord= 0
              ncycle=  0
 * note- reactant GARNET(SS)               has saturated but differs        in composition from the corresponding product.  it
must        remain in the reactant subsystem (rsatch)
 steps completed =    62, iter =  6, ncorr = 0
 most rapidly changing is zvclg1(PYROPE           ) =     -2.7382

 stepping to zi=  5.6747E-01, delzi=  1.0000E-08, nord= 0
              ncycle=  0
 * note- reactant GARNET(SS)               has saturated but differs        in composition from the corresponding product.  it
must        remain in the reactant subsystem (rsatch)
 steps completed =    63, iter =  6, ncorr = 0
 most rapidly changing is zvclg1(PYROPE           ) =     -2.7382

 stepping to zi=  5.6747E-01, delzi=  1.0000E-07, nord= 1
              ncycle=  0
 * note- reactant GARNET(SS)               has saturated but differs        in composition from the corresponding product.  it
must        remain in the reactant subsystem (rsatch)
 steps completed =    64, iter =  6, ncorr = 0
 most rapidly changing is zvclg1(PYROPE           ) =     -2.7381

 stepping to zi=  5.6747E-01, delzi=  1.0000E-06, nord= 2
              ncycle=  0
 * note- reactant GARNET(SS)               has saturated but differs        in composition from the corresponding product.  it
must        remain in the reactant subsystem (rsatch)
 steps completed =    65, iter =  8, ncorr = 0
 most rapidly changing is zvclg1(PYROPE           ) =     -2.7379

 stepping to zi=  5.6748E-01, delzi=  1.0000E-05, nord= 2
              ncycle=  0
 * note- reactant GARNET(SS)               has saturated but differs        in composition from the corresponding product.  it
must        remain in the reactant subsystem (rsatch)
 steps completed =    66, iter = 10, ncorr = 0
 most rapidly changing is zvclg1(PYROPE           ) =     -2.7357

 stepping to zi=  5.6758E-01, delzi=  1.0000E-04, nord= 2
              ncycle=  0
 * note- reactant GARNET(SS)               has saturated but differs        in composition from the corresponding product.  it
must        remain in the reactant subsystem (rsatch)
 steps completed =    67, iter = 13, ncorr = 0
 most rapidly changing is zvclg1(PYROPE           ) =     -2.7139

 stepping to zi=  5.6858E-01, delzi=  1.0000E-03, nord= 3
              ncycle=  0
 * note- reactant GARNET(SS)               has saturated but differs        in composition from the corresponding product.  it
must        remain in the reactant subsystem (rsatch)
 steps completed =    68, iter = 14, ncorr = 0
 most rapidly changing is zvclg1(PYROPE           ) =     -2.5407

 stepping to zi=  5.7495E-01, delzi=  6.3705E-03, nord= 3
              ncycle=  0
 * note- reactant GARNET(SS)               has saturated but differs        in composition from the corresponding product.  it
must        remain in the reactant subsystem (rsatch)
 steps completed =    69, iter = 15, ncorr = 0
 most rapidly changing is zvclg1(PYROPE           ) =     -2.0486

 stepping to zi=  5.9375E-01, delzi=  1.8795E-02, nord= 4
              ncycle=  0
 * note- reactant GARNET(SS)               has saturated but differs        in composition from the corresponding product.  it
must        remain in the reactant subsystem (rsatch)
 steps completed =    70, iter = 16, ncorr = 0
 most rapidly changing is zvclg1(PYROPE           ) =     -1.5666

 stepping to zi=  6.3121E-01, delzi=  3.7464E-02, nord= 4
              ncycle=  0
 * note- reactant GARNET(SS)               has saturated but differs        in composition from the corresponding product.  it
must        remain in the reactant subsystem (rsatch)
 steps completed =    71, iter = 16, ncorr = 0
```

```
 most rapidly changing is zvclg1(PYROPE            ) =     -1.1899

 stepping to zi=  7.2160E-01, delzi=  9.0384E-02, nord= 5
              ncycle=  0
 * note- reactant GARNET(SS)               has saturated but differs        in composition from the corresponding product.  it
must        remain in the reactant subsystem (rsatch)
 steps completed =    72, iter = 17, ncorr = 0
 most rapidly changing is zvclg1(PYROPE            ) =     -0.7957

 stepping to zi=  8.2969E-01, delzi=  1.0809E-01, nord= 5
              ncycle=  0
 * note- reactant GARNET(SS)               has saturated but differs        in composition from the corresponding product.  it
must        remain in the reactant subsystem (rsatch)
 steps completed =    73, iter = 17, ncorr = 0
 most rapidly changing is zvclg1(PYROPE            ) =     -0.5518

 stepping to zi=  9.9639E-01, delzi=  1.6670E-01, nord= 6
              ncycle=  0
 * note- reactant GARNET(SS)               has saturated but differs        in composition from the corresponding product.  it
must        remain in the reactant subsystem (rsatch)
 steps completed =    74, iter = 17, ncorr = 0
 most rapidly changing is zvclg1(PYROPE            ) =     -0.3241

 stepping to zi=  1.0000E+00, delzi=  3.6109E-03, nord= 6
              ncycle=  0
 * note- reactant GARNET(SS)               has saturated but differs        in composition from the corresponding product.  it
must        remain in the reactant subsystem (rsatch)
 steps completed =    75, iter = 13, ncorr = 0
 most rapidly changing is zvclg1(SIDERITE          ) =     -1.2740
 - - - - - - - - - - - - - - - - - - - - - - - - - - - - - - -



                    reaction progress        =  9.99999999999992E-01
                    log of reaction progress =       0.0000000

                    temperature    =    900.000 degrees c
                    total pressure =  50000.000 bars

                    computing units remaining =      0.000


 step size is limited by the print requirement


                              --- reactant summary ---



        reactant            moles       delta moles      grams        delta grams

   CLINOPYROXENE(SS)       1.90000E+01    1.00000E+00    3.98272E+03    2.09617E+02
   GARNET(SS)              1.90000E+01    1.00000E+00    2.76323E+03    1.45433E+02
   COESITE                 1.90000E+01    1.00000E+00    1.14160E+03    6.00843E+01


               current total mass =  7.88756E+03 grams
               delta total mass   =  4.15135E+02 grams
               delta total volume =    199.16800 cc


        reactant           affinity     rel. rate

   CLINOPYROXENE(SS)          0.6007    1.00000E+00
   GARNET(SS)                 0.0842    0.00000E+00
   COESITE                    0.7408    1.00000E+00

          affinity of the overall irreversible reaction=     1.342 kcal
          contributions from irreversible reactions
          with no thermodynamic data are not included



     --- element totals for the aqueous phase ---

   element         mg/kg soln.        molal conc.          moles
```

O	7.071690E+05	1.366929E+02	1.368617E+02
NA	7.424586E+03	9.987671E-01	1.000000E+00
K	2.146567E+04	1.697904E+00	1.700000E+00
CA	4.398578E+04	3.393995E+00	3.398185E+00
MG	1.433052E+03	1.823446E-01	1.825697E-01
AL	6.505207E+03	7.456259E-01	7.465463E-01
SI	3.600664E+04	3.964853E+00	3.969747E+00
H	3.983287E+04	1.222224E+02	1.223733E+02
C	1.314071E+05	3.383500E+01	3.387677E+01
CL	1.144961E+03	9.987671E-02	1.000000E-01
S	1.921971E+03	1.854001E-01	1.856290E-01
FE	1.703214E+03	9.431829E-02	9.443472E-02
co3--		0.000000E+00	0.000000E+00
so4--		0.000000E+00	0.000000E+00
s--		0.000000E+00	0.000000E+00

warning-- co3--, so4--, and s-- totals require that routine comp1
have the names of non-carbonate carbon, sulfide sulfur,
and non-sulfate sulfur aqueous species

single ion activities and activity coefficients are here defined
with respect to the internal ph scale

	ph	eh	pe
internal ph scale	4.6978	1.3554	5.8229E+00
modified nbs ph scale	4.3705	1.4316	6.1502E+00
rational ph scale	4.3705	1.4316	6.1502E+00

phcl = 6.1088

oxygen fugacity = 2.48244E-10
log oxygen fugacity = -9.60512

activity of water = 0.99665
log activity of water = -0.00146
alkalinity = 0.000000E+00 equiv/kg solvent
(not def. for t.gt.50 c)

ionic strength = 6.227032E+00 molal
sum of molalities = 40.0899759690773
osmotic coefficient = 0.00464
equiv. stoich. ionic strength = 9.987671E-02 molal

mass of solution = 3.096438 kg
mass of solvent = 1.001234 kg
mass of solutes = 2.095203 kg
conc of solutes = 67.664959 per cent (w/w)

species	moles	grams	conc	log conc	log g	log act
H2O	5.55772E+01	1.00123E+03				
NA+	8.78652E-01	2.02000E+01	8.77569E-01	-0.05672	-0.32730	-0.38402
K+	1.49042E+00	5.82729E+01	1.48858E+00	0.17277	-0.32730	-0.15453
CA++	1.82820E-04	7.32742E-03	1.82594E-04	-3.73851	-1.30921	-5.04772
MG++	1.43950E-05	3.49871E-04	1.43773E-05	-4.84232	-1.30921	-6.15153
AL+++	1.88750E-15	5.09277E-14	1.88517E-15	-14.72465	-2.94572	-17.67037
SIO2(AQ)	1.31109E+00	7.87757E+01	1.30947E+00	0.11710	0.00000	0.11710
H+	4.26621E-05	4.29992E-05	4.26095E-05	-4.37049	-0.32730	-4.69780
CO3--	1.95055E-01	1.17051E+01	1.94814E-01	-0.71038	-1.30921	-2.01959
CL-	8.25789E-02	2.92767E+00	8.24771E-02	-1.08367	-0.32730	-1.41097
SO4--	2.62464E-04	2.52117E-02	2.62140E-04	-3.58147	-1.30921	-4.89067
FE++	1.87609E-07	1.04774E-05	1.87378E-07	-6.72728	-1.30921	-8.03649
O2(AQ)	1.86301E-16	5.96139E-15	1.86071E-16	-15.73032	0.00000	-15.73032
H2(AQ)	1.30735E-02	2.63535E-02	1.30574E-02	-1.88414	0.00000	-1.88414
CH4(AQ)	5.06811E-02	8.13057E-01	5.06186E-02	-1.29569	0.00000	-1.29569

HS-	3.76194E-02	1.24399E+00	3.75730E-02	-1.42512	-0.32730	-1.75243
FE+++	3.66451E-14	2.04652E-12	3.65999E-14	-13.43652	-2.94572	-16.38224
HCO3-	4.62227E-01	2.82038E+01	4.61657E-01	-0.33568	-0.32730	-0.66298
CLO4-	1.60593E-42	1.59710E-40	1.60395E-42	-41.79481	-0.32730	-42.12211
OH-	4.93830E-01	8.39871E+00	4.93221E-01	-0.30696	-0.32730	-0.63426
HCOO-	2.33516E+00	1.05123E+02	2.33228E+00	0.36778	-0.32730	0.04048
CH3COO-	2.72723E-02	1.61028E+00	2.72386E-02	-1.56481	-0.32730	-1.89212
CH3CH2COO-	4.57116E-01	3.34021E+01	4.56552E-01	-0.34051	-0.32730	-0.66781
CO(AQ)	6.38815E-02	1.78935E+00	6.38027E-02	-1.19516	0.00000	-1.19516
ETHANE(AQ)	2.15800E-04	6.48897E-03	2.15534E-04	-3.66648	0.00000	-3.66648
ETHYLENE(AQ)	4.67785E-07	1.31230E-05	4.67208E-07	-6.33049	0.00000	-6.33049
PROPANE(AQ)	1.23355E-06	5.43948E-05	1.23203E-06	-5.90938	0.00000	-5.90938
HEXANE(AQ)	2.99414E-13	2.58024E-11	2.99044E-13	-12.52426	0.00000	-12.52426
BENZENE(AQ)	9.93919E-13	7.76384E-11	9.92694E-13	-12.00318	0.00000	-12.00318
TOLUENE(AQ)	4.01011E-15	3.69492E-13	4.00516E-15	-14.39738	0.00000	-14.39738
SI2O4(AQ)	2.32341E-01	2.79201E+01	2.32055E-01	-0.63441	0.00000	-0.63441
ALO2-	1.74458E-02	1.02896E+00	1.74243E-02	-1.75884	-0.32730	-2.08615
ALO2(SIO2)-	7.29100E-01	8.68101E+01	7.28202E-01	-0.13775	-0.32730	-0.46505
CACL+	2.53668E-04	1.91603E-02	2.53355E-04	-3.59627	-0.32730	-3.92357
CACL2(AQ)	4.12716E-07	4.58057E-05	4.12208E-07	-6.38488	0.00000	-6.38488
CACO3(AQ)	5.21641E-03	5.22106E-01	5.20998E-03	-2.28316	0.00000	-2.28316
CA(HCO3)+	3.20865E+00	3.24385E+02	3.20469E+00	0.50579	-0.24890	0.25689
CA(OH)+	4.24781E-04	2.42496E-02	4.24258E-04	-3.37237	-0.32730	-3.69967
CA(HSIO3)+	1.83457E-01	2.14960E+01	1.83231E-01	-0.73700	-0.32730	-1.06430
FECL+	1.01071E-07	9.22779E-06	1.00946E-07	-6.99591	-0.32730	-7.32321
FECL2(AQ)	7.94073E-05	1.00651E-02	7.93094E-05	-4.10068	0.00000	-4.10068
FE(HSIO3)+	9.43550E-02	1.25434E+01	9.42387E-02	-1.02577	-0.32730	-1.35307
KCL(AQ)	1.40741E-02	1.04924E+00	1.40567E-02	-1.85212	0.00000	-1.85212
KOH	1.95496E-01	1.09684E+01	1.95255E-01	-0.70940	0.00000	-0.70940
KSO4-	1.08431E-05	1.46550E-03	1.08297E-05	-4.96538	-0.32730	-5.29269
MGCL+	5.65343E-06	3.37838E-04	5.64646E-06	-5.24822	-0.32730	-5.57553
MG(HCO3)+	6.95196E-04	5.93155E-02	6.94338E-04	-3.15843	-0.32730	-3.48573
MG(HSIO3)+	1.81854E-01	1.84394E+01	1.81630E-01	-0.74081	-0.32730	-1.06811
MGSO4(AQ)	9.06214E-09	1.09074E-06	9.05096E-09	-8.04331	0.00000	-8.04331
NACL(AQ)	2.64076E-03	1.54333E-01	2.63750E-03	-2.57881	0.00000	-2.57881
NACO3-	1.51094E-03	1.25406E-01	1.50908E-03	-2.82129	-0.32730	-3.14859
NAHCO3(AQ)	2.12328E-02	1.78370E+00	2.12066E-02	-1.67353	0.00000	-1.67353
NAHSIO3(AQ)	2.28209E-03	2.28394E-01	2.27927E-03	-2.64220	0.00000	-2.64220
NAOH(AQ)	9.36816E-02	3.74699E+00	9.35661E-02	-1.02888	0.00000	-1.02888
HSIO3-	1.00293E+00	7.73174E+01	1.00169E+00	0.00073	-0.32730	-0.32657
HSO4-	1.77094E-03	1.71897E-01	1.76875E-03	-2.75233	-0.32730	-3.07963
H2S(AQ)	1.45965E-01	4.97388E+00	1.45785E-01	-0.83629	0.00000	-0.83629
CO2(AQ)	2.60995E+01	1.14863E+03	2.60673E+01	1.41610	0.00000	1.41610
HCL(AQ)	2.87203E-04	1.04717E-02	2.86849E-04	-3.54235	0.00000	-3.54235
CH3CH2COOH	5.18280E-05	3.83937E-03	5.17641E-05	-4.28597	0.00000	-4.28597
CH3COOH	1.69060E-03	1.01525E-01	1.68852E-03	-2.77249	0.00000	-2.77249
HCOOH	3.13813E-03	1.44434E-01	3.13426E-03	-2.50386	0.00000	-2.50386

--- activity ratios of cations ---

log (NA+ /h+**0)	4.3137741
log (K+ /h+**0)	4.5432658
log (CA++ /h+**0)	4.3478697
log (MG++ /h+**0)	3.2440591
log (AL+++ /h+**0)	-3.5769814
log (FE++ /h+**0)	1.3591001
log (FE+++ /h+**0)	-2.2888524

--- summary of solid product phases---

product	log moles	moles	grams	volume, cc
DIAMOND	-0.5979860	2.52356E-01	3.03105E+00	8.63058E-01
GARNET(SS)	-0.0313989	9.30253E-01	1.36385E+02	1.07505E+02
PYROPE	-0.3201816	4.78430E-01	6.42924E+01	5.41391E+01
ALMANDINE	-0.5707255	2.68704E-01	4.45930E+01	3.04066E+01
GROSSULAR	-0.7372672	1.83119E-01	2.74995E+01	2.29594E+01
CALCITE(SS)	-0.5334686	2.92773E-01	2.70917E+01	8.68784E+00
CALCITE	-1.3354764	4.61874E-02	4.62286E+00	1.70432E+00
MAGNESITE	-0.7135870	1.93381E-01	1.63047E+01	5.42046E+00
SIDERITE	-1.2740460	5.32052E-02	6.16415E+00	1.56306E+00

--- grand summary of solid phases (e.s.+p.r.s.+reactants) ---

phase/end-member	log moles	moles	grams	volume, cc
DIAMOND	-0.5979860	2.52356E-01	3.03105E+00	8.63058E-01
COESITE	1.2787536	1.90000E+01	1.14160E+03	4.31072E+02
CLINOPYROXENE(SS)	1.2787536	1.90000E+01		
DIOPSIDE	0.5797836	3.80000E+00	8.22899E+02	2.51560E+02
HEDENBERGITE	0.2787536	1.90000E+00	4.71379E+02	1.25780E+02
JADEITE	1.1238516	1.33000E+01	2.68844E+03	8.02522E+02
GARNET(SS)	1.2995128	1.99303E+01		
PYROPE	1.0747590	1.18784E+01	1.59625E+03	1.34416E+03
ALMANDINE	0.7758801	5.96870E+00	9.90540E+02	6.75419E+02
GROSSULAR	0.3187140	2.08312E+00	3.12828E+02	2.61181E+02
CALCITE(SS)	-0.5334686	2.92773E-01		
CALCITE	-1.3354764	4.61874E-02	4.62286E+00	1.70432E+00
MAGNESITE	-0.7135870	1.93381E-01	1.63047E+01	5.42046E+00
SIDERITE	-1.2740460	5.32052E-02	6.16415E+00	1.56306E+00

	mass, grams	volume, cc
created	1.665077E+02	1.170560E+02
destroyed	4.151346E+02	1.991680E+02
net	-2.486269E+02	-8.211197E+01

warning-- these volume totals may be incomplete because
of missing partial molar volume data in the data base

--- mineral saturation state summary ---

mineral	affinity, kcal	state	mineral	affinity, kcal	state
DIAMOND	0.0000	satd	CORUNDUM	-4.8191	
BRUCITE	-7.3954		DIASPORE	-2.6867	
CALCITE	-4.3053		ARAGONITE	-2.5810	
MAGNESITE	-0.9669		DOLOMITE	-4.0359	
KYANITE	-2.8378		SILLIMANITE	-7.4404	
GROSSULAR	-3.7892		LAWSONITE	-3.8701	
FORSTERITE	-7.4000		ENSTATITE-CL	-2.0023	
ENSTATITE-OR	-1.9073		ENSTATITE-PR	-3.1071	
DIOPSIDE	-3.3357		HEDENBERGITE	-7.5248	
JADEITE	-1.5005		FERROSILITE	-5.7432	
K-FELDSPAR	-8.1609		TALC	-9.6120	
MUSCOVITE	-4.5668		PHLOGOPITE	-3.4060	
PYROPE	-1.5502		ALMANDINE	-2.8952	
COESITE	-0.7408		GRAPHITE	-0.3430	
PYRRHOTITE	-2.8688		PYRITE	-3.6568	
FERROUS_OXIDE	-5.6454		GIBBSITE	-6.8489	
SEPIOLITE	1042.8677	ssatd	SIDERITE	-3.9755	
QUARTZ-ALPHA	-1.3791		QUARTZ-BETA	-2.4570	
CRISTOBALITE-ALPHA	-6.1814		CRISTOBALITE-BETA	-8.2509	
CHALCEDONY	-2.3336		AMORPHOUS_SILICA	-3.6724	

--- summary of solid solutions ---

mineral	aff. kcal/mol		mole frac.	lambda	state
ORTHOPYROXENE(SS)	-1.4960				
FERROSILITE		-1.49599	0.1617407	1.00000	
ENSTATITE-OR		-1.49599	0.8382593	1.00000	

```
 OLIVINE                  -7.1118
     FAYALITE                   -7.11179    0.1162780     1.00000
     FORSTERITE                 -7.11179    0.8837220     1.00000

 BIOTITE                  -3.3968
     PHLOGOPITE                 -3.39683    0.9960689     1.00000
     ANNITE                     -3.39683    0.0039311     1.00000

 CLINOPYROXENE(SS)        -0.5082
     DIOPSIDE                   -0.50818    0.2973542     1.00000
     HEDENBERGITE               -0.50818    0.0493077     1.00000
     JADEITE                    -0.50818    0.6533381     1.00000

 GARNET(SS)                0.0000                              saturated
     PYROPE                      0.00000    0.5143010     1.00000
     ALMANDINE                   0.00000    0.2888507     1.00000
     GROSSULAR                   0.00000    0.1968484     1.00000

 CALCITE(SS)               0.0000                              saturated
     CALCITE                     0.00000    0.1577583     1.00000
     MAGNESITE                   0.00000    0.6605134     1.00000
     SIDERITE                    0.00000    0.1817283     1.00000


 solid solution product phases

            xbar     lambda   activity  log xbar  log lambda log activity

   GARNET(SS)
 ideal solution

     PYROPE
             0.5143    1.0000    0.5143   -0.2888    0.0000   -0.2888
     ALMANDINE
             0.2889    1.0000    0.2889   -0.5393    0.0000   -0.5393
     GROSSULAR
             0.1968    1.0000    0.1968   -0.7059    0.0000   -0.7059

   CALCITE(SS)
 ideal solution

     CALCITE
             0.1578    1.0000    0.1578   -0.8020    0.0000   -0.8020
     MAGNESITE
             0.6605    1.0000    0.6605   -0.1801    0.0000   -0.1801
     SIDERITE
             0.1817    1.0000    0.1817   -0.7406    0.0000   -0.7406


           --- summary of gas species ---

           gas               log fugacity         fugacity         partial pressure

   CO2(G)                         9.10468       1.27256E+09
   O2(G)                         -9.60512       2.48244E-10
   S2(G)                          4.01635       1.03836E+04
   CH4(G)                         6.97431       9.42563E+06
   H2(G)                          3.80991       6.45513E+03
   H2S(G)                         7.28178       1.91328E+07
   H2O(G)                         6.90034       7.94958E+06



 - - - - - - - - - - - - - - - - - - - - - - - - - - - - - - - - -



 stepping to zi=  1.0361E+00, delzi=  3.6109E-02, nord= 4
               ncycle=  0
 * note- reactant GARNET(SS)               has saturated but differs        in composition from the corresponding product.  it
must        remain in the reactant subsystem (rsatch)
 steps completed =    76, iter = 15, ncorr = 0
 most rapidly changing is zvclg1(SIDERITE          ) =     -1.3209

 stepping to zi=  1.2025E+00, delzi=  1.6641E-01, nord= 6
```

```
              ncycle=  0
 * note- reactant GARNET(SS)               has saturated but differs        in composition from the corresponding product.  it
must        remain in the reactant subsystem (rsatch)
 steps completed =    77, iter = 16, ncorr = 0
 most rapidly changing is zvclg1(SIDERITE          ) =     -1.6073

 stepping to zi=  1.2201E+00, delzi=  1.7558E-02, nord= 6
              ncycle=  0
 iter =   14
       0 supersaturated pure minerals
       1 supersaturated solid solutions

                         the most supersaturated phases     affinity, kcal

                           1   50900   CLINOPYROXENE(SS)       0.00901584


   attempted species assemblage no.   2

             1      1  H2O
             2      2  NA+
             3      3  K+
             4      4  CA++
             5      5  MG++
             6      6  AL+++
             7      7  SIO2(AQ)
             8     13  H+
             9     14  CO3--
            10     16  CL-
            11     17  SO4--
            12     21  FE++
            13     29  O2(G)
            14      2  DIAMOND
            15      1  CLINOPYRDIOPSIDE
            16      2  CLINOPYRHEDENBERGITE
            17      3  CLINOPYRJADEITE
            18      1  GARNET(SPYROPE
            19      2  GARNET(SALMANDINE
            20      3  GARNET(SGROSSULAR
            21      1  CALCITE(CALCITE
            22      2  CALCITE(MAGNESITE
            23      3  CALCITE(SIDERITE

 * note- reactant CLINOPYROXENE(SS)        has saturated but differs        in composition from the corresponding product.  it
must        remain in the reactant subsystem (rsatch)
 * note- reactant GARNET(SS)               has saturated but differs        in composition from the corresponding product.  it
must        remain in the reactant subsystem (rsatch)
 steps completed =    78, iter = 19, ncorr = 0
 - - - - - - - - - - - - - - - - - - - - - - - - - - - - - - - -



                    reaction progress        =  1.22007493643591E+00
                    log of reaction progress =       0.0863865

                    temperature    =    900.000 degrees c
                    total pressure =  50000.000 bars

                    computing units remaining =      0.000


 change in the product phase assemblage


                              --- reactant summary ---



        reactant            moles       delta moles      grams        delta grams

   CLINOPYROXENE(SS)       1.87799E+01    1.22007E+00    3.93659E+03    2.55748E+02
   GARNET(SS)              1.87799E+01    1.22007E+00    2.73123E+03    1.77439E+02
   COESITE                 1.87799E+01    1.22007E+00    1.12838E+03    7.33073E+01


               current total mass =  7.79620E+03 grams
```

delta total mass = 5.06495E+02 grams
delta total volume = 242.99988 cc

reactant	affinity	rel. rate
CLINOPYROXENE(SS)	0.1011	0.00000E+00
GARNET(SS)	0.0861	0.00000E+00
COESITE	0.4960	1.00000E+00

affinity of the overall irreversible reaction= 0.496 kcal
contributions from irreversible reactions
with no thermodynamic data are not included

--- element totals for the aqueous phase ---

element	mg/kg soln.	molal conc.	moles
O	7.049936E+05	1.381848E+02	1.380636E+02
NA	8.442352E+03	1.151616E+00	1.150605E+00
K	2.121335E+04	1.701493E+00	1.700000E+00
CA	4.364411E+04	3.414890E+00	3.411894E+00
MG	1.569130E+03	2.024613E-01	2.022836E-01
AL	6.285937E+03	7.306040E-01	7.299630E-01
SI	3.915714E+04	4.372277E+00	4.368441E+00
H	3.936465E+04	1.224807E+02	1.223733E+02
C	1.305675E+05	3.409061E+01	3.406070E+01
CL	1.131502E+03	1.000878E-01	1.000000E-01
S	1.899379E+03	1.857920E-01	1.856290E-01
FE	1.731352E+03	9.722196E-02	9.713666E-02
co3--		0.000000E+00	0.000000E+00
so4--		0.000000E+00	0.000000E+00
s--		0.000000E+00	0.000000E+00

warning-- co3--, so4--, and s-- totals require that routine comp1
have the names of non-carbonate carbon, sulfide sulfur,
and non-sulfate sulfur aqueous species

single ion activities and activity coefficients are here defined
with respect to the internal ph scale

	ph	eh	pe
internal ph scale	4.7019	1.3546	5.8196E+00
modified nbs ph scale	4.3735	1.4310	6.1480E+00
rational ph scale	4.3735	1.4310	6.1480E+00

phcl = 6.1143

oxygen fugacity = 2.50016E-10
log oxygen fugacity = -9.60203

activity of water = 0.99665
log activity of water = -0.00146
alkalinity = 0.000000E+00 equiv/kg solvent
(not def. for t.gt.50 c)

ionic strength = 6.413077E+00 molal
sum of molalities = 40.8469260536211
osmotic coefficient = 0.00456
equiv. stoich. ionic strength = 1.000878E-01 molal

mass of solution = 3.133268 kg
mass of solvent = 0.999123 kg
mass of solutes = 2.134146 kg
conc of solutes = 68.112446 per cent (w/w)

species	moles	grams	conc	log conc	log g	log act
H2O	5.54600E+01	9.99123E+02				
NA+	1.00973E+00	2.32134E+01	1.01061E+00	0.00458	-0.32837	-0.32379
K+	1.48931E+00	5.82293E+01	1.49061E+00	0.17337	-0.32837	-0.15501
CA++	1.81067E-04	7.25718E-03	1.81226E-04	-3.74178	-1.31349	-5.05527
MG++	1.43364E-05	3.48447E-04	1.43490E-05	-4.84318	-1.31349	-6.15667
AL+++	1.63614E-15	4.41454E-14	1.63757E-15	-14.78580	-2.95535	-17.74115
SIO2(AQ)	1.45317E+00	8.73130E+01	1.45445E+00	0.16270	0.00000	0.16270
H+	4.22789E-05	4.26129E-05	4.23161E-05	-4.37349	-0.32837	-4.70187
CO3--	2.01720E-01	1.21050E+01	2.01897E-01	-0.69487	-1.31349	-2.00836
CL-	8.23208E-02	2.91852E+00	8.23931E-02	-1.08411	-0.32837	-1.41248
SO4--	2.73204E-04	2.62433E-02	2.73444E-04	-3.56313	-1.31349	-4.87662
FE++	1.73414E-07	9.68468E-06	1.73567E-07	-6.76053	-1.31349	-8.07402
O2(AQ)	1.87235E-16	5.99128E-15	1.87399E-16	-15.72723	0.00000	-15.72723
H2(AQ)	1.29995E-02	2.62044E-02	1.30109E-02	-1.88569	0.00000	-1.88569
CH4(AQ)	5.02151E-02	8.05580E-01	5.02592E-02	-1.29878	0.00000	-1.29878
HS-	3.79638E-02	1.25538E+00	3.79972E-02	-1.42025	-0.32837	-1.74862
FE+++	3.40329E-14	1.90064E-12	3.40628E-14	-13.46772	-2.95535	-16.42307
HCO3-	4.70074E-01	2.86825E+01	4.70486E-01	-0.32745	-0.32837	-0.65582
CLO4-	1.62384E-42	1.61492E-40	1.62527E-42	-41.78907	-0.32837	-42.11745
OH-	4.98653E-01	8.48075E+00	4.99091E-01	-0.30182	-0.32837	-0.63019
HCOO-	2.36637E+00	1.06528E+02	2.36845E+00	0.37446	-0.32837	0.04609
CH3COO-	2.75385E-02	1.62600E+00	2.75627E-02	-1.55968	-0.32837	-1.88805
CH3CH2COO-	4.59936E-01	3.36081E+01	4.60340E-01	-0.33692	-0.32837	-0.66529
CO(AQ)	6.39738E-02	1.79193E+00	6.40300E-02	-1.19362	0.00000	-1.19362
ETHANE(AQ)	2.13055E-04	6.40643E-03	2.13242E-04	-3.67113	0.00000	-3.67113
ETHYLENE(AQ)	4.63483E-07	1.30024E-05	4.63890E-07	-6.33358	0.00000	-6.33358
PROPANE(AQ)	1.21353E-06	5.35119E-05	1.21459E-06	-5.91557	0.00000	-5.91557
HEXANE(AQ)	2.91422E-13	2.51137E-11	2.91678E-13	-12.53510	0.00000	-12.53510
BENZENE(AQ)	9.81277E-13	7.66509E-11	9.82139E-13	-12.00783	0.00000	-12.00783
TOLUENE(AQ)	3.94502E-15	3.63495E-13	3.94848E-15	-14.40357	0.00000	-14.40357
SI2O4(AQ)	2.86033E-01	3.43722E+01	2.86285E-01	-0.54320	0.00000	-0.54320
ALO2-	1.53938E-02	9.07930E-01	1.54073E-02	-1.81227	-0.32837	-2.14065
ALO2(SIO2)-	7.14569E-01	8.50799E+01	7.15197E-01	-0.14557	-0.32837	-0.47395
CACL+	2.48520E-04	1.87714E-02	2.48738E-04	-3.60426	-0.32837	-3.93263
CACL2(AQ)	4.01943E-07	4.46100E-05	4.02296E-07	-6.39545	0.00000	-6.39545
CACO3(AQ)	5.24974E-03	5.25442E-01	5.25435E-03	-2.27948	0.00000	-2.27948
CA(HCO3)+	3.20358E+00	3.23872E+02	3.20639E+00	0.50602	-0.24952	0.25650
CA(OH)+	4.21542E-04	2.40647E-02	4.21912E-04	-3.37478	-0.32837	-3.70315
CA(HSIO3)+	2.02215E-01	2.36939E+01	2.02393E-01	-0.69380	-0.32837	-1.02218
FECL+	9.24137E-08	8.43737E-06	9.24949E-08	-7.03388	-0.32837	-7.36225
FECL2(AQ)	7.21751E-05	9.14842E-03	7.22385E-05	-4.14123	0.00000	-4.14123
FE(HSIO3)+	9.70642E-02	1.29036E+01	9.71495E-02	-1.01256	-0.32837	-1.34093
KCL(AQ)	1.39802E-02	1.04224E+00	1.39925E-02	-1.85411	0.00000	-1.85411
KOH	1.96703E-01	1.10361E+01	1.96876E-01	-0.70581	0.00000	-0.70581
KSO4-	1.11913E-05	1.51257E-03	1.12012E-05	-4.95074	-0.32837	-5.27911
MGCL+	5.56953E-06	3.32824E-04	5.57442E-06	-5.25380	-0.32837	-5.58217
MG(HCO3)+	6.98688E-04	5.96135E-02	6.99302E-04	-3.15534	-0.32837	-3.48371
MG(HSIO3)+	2.01565E-01	2.04380E+01	2.01742E-01	-0.69520	-0.32837	-1.02358
MGSO4(AQ)	9.23069E-09	1.11103E-06	9.23879E-09	-8.03438	0.00000	-8.03438
NACL(AQ)	3.01671E-03	1.76305E-01	3.01936E-03	-2.52009	0.00000	-2.52009
NACO3-	1.78182E-03	1.47889E-01	1.78338E-03	-2.74876	-0.32837	-3.07713
NAHCO3(AQ)	2.47446E-02	2.07872E+00	2.47664E-02	-1.60614	0.00000	-1.60614
NAHSIO3(AQ)	2.93306E-03	2.93545E-01	2.93564E-03	-2.53230	0.00000	-2.53230
NAOH(AQ)	1.08403E-01	4.33579E+00	1.08498E-01	-0.96458	0.00000	-0.96458
HSIO3-	1.12485E+00	8.67167E+01	1.12584E+00	0.05148	-0.32837	-0.27690
HSO4-	1.81275E-03	1.75956E-01	1.81435E-03	-2.74128	-0.32837	-3.06965
H2S(AQ)	1.45568E-01	4.96035E+00	1.45696E-01	-0.83655	0.00000	-0.83655
CO2(AQ)	2.62303E+01	1.15439E+03	2.62533E+01	1.41918	0.00000	1.41918
HCL(AQ)	2.82936E-04	1.03161E-02	2.83185E-04	-3.54793	0.00000	-3.54793
CH3CH2COOH	5.15340E-05	3.81759E-03	5.15792E-05	-4.28753	0.00000	-4.28753
CH3COOH	1.68701E-03	1.01309E-01	1.68849E-03	-2.77250	0.00000	-2.77250
HCOOH	3.14265E-03	1.44642E-01	3.14541E-03	-2.50232	0.00000	-2.50232

--- activity ratios of cations ---

log (NA+ /h+**0)	4.3780798
log (K+ /h+**0)	4.5468599
log (CA++ /h+**0)	4.3484672
log (MG++ /h+**0)	3.2470684
log (AL+++ /h+**0)	-3.6355467
log (FE++ /h+**0)	1.3297124

log (FE+++ /h+**0)   -2.3174664

--- summary of solid product phases---

product	log moles	moles	grams	volume, cc
DIAMOND	-0.6432231	2.27393E-01	2.73122E+00	7.77684E-01
CLINOPYROXENE(SS)	-2.2799625	5.24853E-03	1.09447E+00	3.27253E-01
DIOPSIDE	-2.8065465	1.56118E-03	3.38078E-01	1.03350E-01
HEDENBERGITE	-3.6193026	2.40269E-04	5.96093E-02	1.59058E-02
JADEITE	-2.4625490	3.44708E-03	6.96788E-01	2.07997E-01
GARNET(SS)	0.1465503	1.40136E+00	2.04873E+02	1.62005E+02
PYROPE	-0.1326269	7.36840E-01	9.90181E+01	8.33808E+01
ALMANDINE	-0.4156003	3.84061E-01	6.37370E+01	4.34603E+01
GROSSULAR	-0.5521267	2.80462E-01	4.21177E+01	3.51643E+01
CALCITE(SS)	-0.8735271	1.33805E-01	1.23394E+01	3.97024E+00
CALCITE	-1.6718516	2.12887E-02	2.13077E+00	7.85552E-01
MAGNESITE	-1.0475503	8.96292E-02	7.55702E+00	2.51231E+00
SIDERITE	-1.6404063	2.28873E-02	2.65163E+00	6.72382E-01

--- grand summary of solid phases (e.s.+p.r.s.+reactants) ---

phase/end-member	log moles	moles	grams	volume, cc
DIAMOND	-0.6432231	2.27393E-01	2.73122E+00	7.77684E-01
COESITE	1.2736939	1.87799E+01	1.12838E+03	4.26079E+02
CLINOPYROXENE(SS)	1.2738152	1.87852E+01		
DIOPSIDE	0.5749043	3.75755E+00	8.13706E+02	2.48750E+02
HEDENBERGITE	0.2737494	1.87823E+00	4.65979E+02	1.24339E+02
JADEITE	1.1189058	1.31494E+01	2.65800E+03	7.93434E+02
GARNET(SS)	1.3049489	2.01813E+01		
PYROPE	1.0793547	1.20048E+01	1.61323E+03	1.35846E+03
ALMANDINE	0.7794549	6.01804E+00	9.98727E+02	6.81001E+02
GROSSULAR	0.3341428	2.15845E+00	3.24141E+02	2.70627E+02
CALCITE(SS)	-0.8735271	1.33805E-01		
CALCITE	-1.6718516	2.12887E-02	2.13077E+00	7.85552E-01
MAGNESITE	-1.0475503	8.96292E-02	7.55702E+00	2.51231E+00
SIDERITE	-1.6404063	2.28873E-02	2.65163E+00	6.72382E-01

	mass, grams	volume, cc
created	2.210379E+02	1.670805E+02
destroyed	5.064953E+02	2.429999E+02
net	-2.854573E+02	-7.591934E+01

warning-- these volume totals may be incomplete because
of missing partial molar volume data in the data base

--- mineral saturation state summary ---

mineral	affinity, kcal	state	mineral	affinity, kcal	state
DIAMOND	0.0000	satd	CORUNDUM	-5.4480	
BRUCITE	-7.3793		DIASPORE	-3.0011	
CALCITE	-4.2855		ARAGONITE	-2.5613	
MAGNESITE	-0.9342		DOLOMITE	-3.9834	
KYANITE	-3.2218		SILLIMANITE	-7.8244	

GROSSULAR	-3.7506	LAWSONITE	-4.0061
FORSTERITE	-7.1229	ENSTATITE-CL	-1.7414
ENSTATITE-OR	-1.6464	ENSTATITE-PR	-2.8461
DIOPSIDE	-2.8268	HEDENBERGITE	-7.1898
JADEITE	-0.9802	FERROSILITE	-5.6561
K-FELDSPAR	-7.7216	TALC	-8.5844
MUSCOVITE	-4.7564	PHLOGOPITE	-2.9183
PYROPE	-1.4987	ALMANDINE	-3.0177
COESITE	-0.4960	GRAPHITE	-0.3430
PYRRHOTITE	-3.0280	PYRITE	-3.8091
FERROUS_OXIDE	-5.8032	GIBBSITE	-7.1634
SEPIOLITE	1043.6344 ssatd	SIDERITE	-4.1167
QUARTZ-ALPHA	-1.1343	QUARTZ-BETA	-2.2122
CRISTOBALITE-ALPHA	-5.9366	CRISTOBALITE-BETA	-8.0061
CHALCEDONY	-2.0887	AMORPHOUS_SILICA	-3.4276

--- summary of solid solutions ---

mineral	aff. kcal/mol	mole frac.	lambda	state
ORTHOPYROXENE(SS)	-1.2623			
FERROSILITE	-1.26231	0.1518802	1.00000	
ENSTATITE-OR	-1.26231	0.8481198	1.00000	
OLIVINE	-6.8726			
FAYALITE	-6.87258	0.1018028	1.00000	
FORSTERITE	-6.87258	0.8981972	1.00000	
BIOTITE	-2.9110			
PHLOGOPITE	-2.91098	0.9968547	1.00000	
ANNITE	-2.91098	0.0031453	1.00000	
CLINOPYROXENE(SS)	0.0000			saturated
DIOPSIDE	0.00000	0.2974514	1.00000	
HEDENBERGITE	0.00000	0.0457783	1.00000	
JADEITE	0.00000	0.6567703	1.00000	
GARNET(SS)	0.0000			saturated
PYROPE	0.00000	0.5258027	1.00000	
ALMANDINE	0.00000	0.2740624	1.00000	
GROSSULAR	0.00000	0.2001350	1.00000	
CALCITE(SS)	0.0000			saturated
CALCITE	0.00000	0.1591020	1.00000	
MAGNESITE	0.00000	0.6698489	1.00000	
SIDERITE	0.00000	0.1710491	1.00000	

solid solution product phases

	xbar	lambda	activity	log xbar	log lambda	log activity
CLINOPYROXENE(SS) ideal solution						
DIOPSIDE	0.2975	1.0000	0.2975	-0.5266	0.0000	-0.5266
HEDENBERGITE	0.0458	1.0000	0.0458	-1.3393	0.0000	-1.3393
JADEITE	0.6568	1.0000	0.6568	-0.1826	0.0000	-0.1826
GARNET(SS) ideal solution						
PYROPE	0.5258	1.0000	0.5258	-0.2792	0.0000	-0.2792
ALMANDINE	0.2741	1.0000	0.2741	-0.5622	0.0000	-0.5622
GROSSULAR	0.2001	1.0000	0.2001	-0.6987	0.0000	-0.6987

CALCITE(SS)
ideal solution

```
     CALCITE
             0.1591    1.0000    0.1591   -0.7983    0.0000   -0.7983
     MAGNESITE
             0.6698    1.0000    0.6698   -0.1740    0.0000   -0.1740
     SIDERITE
             0.1710    1.0000    0.1710   -0.7669    0.0000   -0.7669


          --- summary of gas species ---

          gas               log fugacity         fugacity         partial pressure

   CO2(G)                        9.10777       1.28164E+09
   O2(G)                        -9.60203       2.50016E-10
   S2(G)                         4.01891       1.04450E+04
   CH4(G)                        6.97122       9.35869E+06
   H2(G)                         3.80836       6.43217E+03
   H2S(G)                        7.28151       1.91211E+07
   H2O(G)                        6.90034       7.94953E+06



 - - - - - - - - - - - - - - - - - - - - - - - - - - - - - - - -



 stepping to zi=  1.2201E+00, delzi=  1.0000E-08, nord= 0
               ncycle=  0
  * note- reactant CLINOPYROXENE(SS)        has saturated but differs        in composition from the corresponding product.  it
must        remain in the reactant subsystem (rsatch)
  * note- reactant GARNET(SS)               has saturated but differs        in composition from the corresponding product.  it
must        remain in the reactant subsystem (rsatch)
 steps completed =    79, iter =  5, ncorr = 0
 most rapidly changing is zvclg1(HEDENBERGITE      ) =     -3.6193

 stepping to zi=  1.2201E+00, delzi=  1.0000E-08, nord= 0
               ncycle=  0
  * note- reactant CLINOPYROXENE(SS)        has saturated but differs        in composition from the corresponding product.  it
must        remain in the reactant subsystem (rsatch)
  * note- reactant GARNET(SS)               has saturated but differs        in composition from the corresponding product.  it
must        remain in the reactant subsystem (rsatch)
 steps completed =    80, iter =  5, ncorr = 0
 most rapidly changing is zvclg1(HEDENBERGITE      ) =     -3.6193

 stepping to zi=  1.2201E+00, delzi=  1.0000E-08, nord= 0
               ncycle=  0
  * note- reactant CLINOPYROXENE(SS)        has saturated but differs        in composition from the corresponding product.  it
must        remain in the reactant subsystem (rsatch)
  * note- reactant GARNET(SS)               has saturated but differs        in composition from the corresponding product.  it
must        remain in the reactant subsystem (rsatch)
 steps completed =    81, iter =  5, ncorr = 0
 most rapidly changing is zvclg1(HEDENBERGITE      ) =     -3.6193

 stepping to zi=  1.2201E+00, delzi=  1.0000E-07, nord= 1
               ncycle=  0
  * note- reactant CLINOPYROXENE(SS)        has saturated but differs        in composition from the corresponding product.  it
must        remain in the reactant subsystem (rsatch)
  * note- reactant GARNET(SS)               has saturated but differs        in composition from the corresponding product.  it
must        remain in the reactant subsystem (rsatch)
 steps completed =    82, iter =  5, ncorr = 0
 most rapidly changing is zvclg1(HEDENBERGITE      ) =     -3.6193

 stepping to zi=  1.2201E+00, delzi=  1.0000E-06, nord= 2
               ncycle=  0
  * note- reactant CLINOPYROXENE(SS)        has saturated but differs        in composition from the corresponding product.  it
must        remain in the reactant subsystem (rsatch)
  * note- reactant GARNET(SS)               has saturated but differs        in composition from the corresponding product.  it
must        remain in the reactant subsystem (rsatch)
 steps completed =    83, iter =  7, ncorr = 0
 most rapidly changing is zvclg1(HEDENBERGITE      ) =     -3.6192

 stepping to zi=  1.2201E+00, delzi=  1.0000E-05, nord= 2
               ncycle=  0
  * note- reactant CLINOPYROXENE(SS)        has saturated but differs        in composition from the corresponding product.  it
must        remain in the reactant subsystem (rsatch)
  * note- reactant GARNET(SS)               has saturated but differs        in composition from the corresponding product.  it
```

```
must        remain in the reactant subsystem (rsatch)
 steps completed =    84, iter =  9, ncorr = 0
 most rapidly changing is zvclg1(HEDENBERGITE      ) =     -3.6181

 stepping to zi=  1.2202E+00, delzi=  1.0000E-04, nord= 2
              ncycle=  0
 * note- reactant CLINOPYROXENE(SS)        has saturated but differs        in composition from the corresponding product.  it
must        remain in the reactant subsystem (rsatch)
 * note- reactant GARNET(SS)               has saturated but differs        in composition from the corresponding product.  it
must        remain in the reactant subsystem (rsatch)
 steps completed =    85, iter = 11, ncorr = 0
 most rapidly changing is zvclg1(HEDENBERGITE      ) =     -3.6077

 stepping to zi=  1.2212E+00, delzi=  1.0000E-03, nord= 2
              ncycle=  0
 * note- reactant CLINOPYROXENE(SS)        has saturated but differs        in composition from the corresponding product.  it
must        remain in the reactant subsystem (rsatch)
 * note- reactant GARNET(SS)               has saturated but differs        in composition from the corresponding product.  it
must        remain in the reactant subsystem (rsatch)
 steps completed =    86, iter = 13, ncorr = 0
 most rapidly changing is zvclg1(HEDENBERGITE      ) =     -3.5156

 stepping to zi=  1.2312E+00, delzi=  1.0000E-02, nord= 3
              ncycle=  0
 * note- reactant CLINOPYROXENE(SS)        has saturated but differs        in composition from the corresponding product.  it
must        remain in the reactant subsystem (rsatch)
 * note- reactant GARNET(SS)               has saturated but differs        in composition from the corresponding product.  it
must        remain in the reactant subsystem (rsatch)
 steps completed =    87, iter = 15, ncorr = 0
 most rapidly changing is zvclg1(HEDENBERGITE      ) =     -3.0501

 stepping to zi=  1.2527E+00, delzi=  2.1479E-02, nord= 4
              ncycle=  0
 * note- reactant CLINOPYROXENE(SS)        has saturated but differs        in composition from the corresponding product.  it
must        remain in the reactant subsystem (rsatch)
 * note- reactant GARNET(SS)               has saturated but differs        in composition from the corresponding product.  it
must        remain in the reactant subsystem (rsatch)
 steps completed =    88, iter = 15, ncorr = 0
 most rapidly changing is zvclg1(HEDENBERGITE      ) =     -2.6647

 stepping to zi=  1.3414E+00, delzi=  8.8707E-02, nord= 4
              ncycle=  0
 * note- reactant CLINOPYROXENE(SS)        has saturated but differs        in composition from the corresponding product.  it
must        remain in the reactant subsystem (rsatch)
 * note- reactant GARNET(SS)               has saturated but differs        in composition from the corresponding product.  it
must        remain in the reactant subsystem (rsatch)
 steps completed =    89, iter = 15, ncorr = 0
 most rapidly changing is zvclg1(HEDENBERGITE      ) =     -2.1178

 stepping to zi=  1.5385E+00, delzi=  1.9711E-01, nord= 5
              ncycle=  0
 * note- reactant CLINOPYROXENE(SS)        has saturated but differs        in composition from the corresponding product.  it
must        remain in the reactant subsystem (rsatch)
 * note- reactant GARNET(SS)               has saturated but differs        in composition from the corresponding product.  it
must        remain in the reactant subsystem (rsatch)
 steps completed =    90, iter = 17, ncorr = 0
 most rapidly changing is zvclg1(HEDENBERGITE      ) =     -1.6826

 stepping to zi=  1.7855E+00, delzi=  2.4706E-01, nord= 5
              ncycle=  0
 * note- reactant CLINOPYROXENE(SS)        has saturated but differs        in composition from the corresponding product.  it
must        remain in the reactant subsystem (rsatch)
 * note- reactant GARNET(SS)               has saturated but differs        in composition from the corresponding product.  it
must        remain in the reactant subsystem (rsatch)
 steps completed =    91, iter = 18, ncorr = 0
 most rapidly changing is zvclg1(CALCITE           ) =     -2.3850

 stepping to zi=  1.8971E+00, delzi=  1.1152E-01, nord= 6
                    the phase to be dropped is CALCITE(SS)            (51500)


   attempted species assemblage no.   2

              1      1  H2O
              2      2  NA+
              3      3  K+
```

```
            4      4  CA++
            5      5  MG++
            6      6  AL+++
            7      7  SIO2(AQ)
            8     13  H+
            9     14  CO3--
           10     16  CL-
           11     17  SO4--
           12     21  FE++
           13     29  O2(G)
           14      2  DIAMOND
           15      1  CLINOPYRDIOPSIDE
           16      2  CLINOPYRHEDENBERGITE
           17      3  CLINOPYRJADEITE
           18      1  GARNET(SPYROPE
           19      2  GARNET(SALMANDINE
           20      3  GARNET(SGROSSULAR

 * note- reactant CLINOPYROXENE(SS)        has saturated but differs        in composition from the corresponding product.  it
must        remain in the reactant subsystem (rsatch)
 * note- reactant GARNET(SS)               has saturated but differs        in composition from the corresponding product.  it
must        remain in the reactant subsystem (rsatch)
 steps completed =    92, iter = 16, ncorr = 0
 - - - - - - - - - - - - - - - - - - - - - - - - - - - - - - - -



                    reaction progress        =  1.89705255825827E+00
                    log of reaction progress =       0.2780794

                    temperature    =    900.000 degrees c
                    total pressure =  50000.000 bars

                    computing units remaining =      0.000


 change in the product phase assemblage


                              --- reactant summary ---



        reactant            moles       delta moles      grams        delta grams

   CLINOPYROXENE(SS)       1.81029E+01    1.89705E+00    3.79469E+03    3.97654E+02
   GARNET(SS)              1.81029E+01    1.89705E+00    2.63277E+03    2.75895E+02
   COESITE                 1.81029E+01    1.89705E+00    1.08770E+03    1.13983E+02


               current total mass =  7.51516E+03 grams
               delta total mass   =  7.87532E+02 grams
               delta total volume =    377.83216 cc


        reactant           affinity     rel. rate

   CLINOPYROXENE(SS)          0.1067    0.00000E+00
   GARNET(SS)                 0.0670    0.00000E+00
   COESITE                    0.2888    1.00000E+00

         affinity of the overall irreversible reaction=     0.289 kcal
         contributions from irreversible reactions
         with no thermodynamic data are not included



      --- element totals for the aqueous phase ---

   element         mg/kg soln.         molal conc.          moles

      O         7.054462E+05        1.385820E+02        1.387546E+02
      NA        7.876512E+03        1.076827E+00        1.078168E+00
      K         2.112125E+04        1.697885E+00        1.700000E+00
      CA        4.179702E+04        3.277662E+00        3.281745E+00
      MG        1.742077E+03        2.252777E-01        2.255584E-01
```

AL	5.794725E+03	6.750138E-01	6.758546E-01
SI	4.178083E+04	4.675644E+00	4.681469E+00
H	3.919374E+04	1.222210E+02	1.223733E+02
C	1.301222E+05	3.405013E+01	3.409255E+01
CL	1.126590E+03	9.987559E-02	1.000000E-01
S	1.891133E+03	1.853980E-01	1.856290E-01
FE	2.107720E+03	1.186204E-01	1.187682E-01
co3--		0.000000E+00	0.000000E+00
so4--		0.000000E+00	0.000000E+00
s--		0.000000E+00	0.000000E+00

warning-- co3--, so4--, and s-- totals require that routine comp1
have the names of non-carbonate carbon, sulfide sulfur,
and non-sulfate sulfur aqueous species

single ion activities and activity coefficients are here defined
with respect to the internal ph scale

	ph	eh	pe
internal ph scale	4.6865	1.3585	5.8361E+00
modified nbs ph scale	4.3590	1.4347	6.1635E+00
rational ph scale	4.3590	1.4347	6.1635E+00

phcl = 6.0987

oxygen fugacity = 2.52565E-10
log oxygen fugacity = -9.59763

activity of water = 0.99665
log activity of water = -0.00146
alkalinity = 0.000000E+00 equiv/kg solvent
(not def. for t.gt.50 c)

ionic strength = 6.247025E+00 molal
sum of molalities = 40.9961244572130
osmotic coefficient = 0.00454
equiv. stoich. ionic strength = 9.987559E-02 molal

mass of solution = 3.146931 kg
mass of solvent = 1.001246 kg
mass of solutes = 2.145685 kg
conc of solutes = 68.183423 per cent (w/w)

species	moles	grams	conc	log conc	log g	log act
H2O	5.55778E+01	1.00125E+03				
NA+	9.49492E-01	2.18286E+01	9.48311E-01	-0.02305	-0.32742	-0.35047
K+	1.49493E+00	5.84492E+01	1.49307E+00	0.17408	-0.32742	-0.15334
CA++	1.76450E-04	7.07213E-03	1.76231E-04	-3.75392	-1.30968	-5.06359
MG++	1.50586E-05	3.66000E-04	1.50399E-05	-4.82276	-1.30968	-6.13243
AL+++	1.57223E-15	4.24211E-14	1.57027E-15	-14.80403	-2.94677	-17.75080
SIO2(AQ)	1.59165E+00	9.56334E+01	1.58967E+00	0.20131	0.00000	0.20131
H+	4.38020E-05	4.41480E-05	4.37475E-05	-4.35905	-0.32742	-4.68647
CO3--	1.88568E-01	1.13158E+01	1.88333E-01	-0.72507	-1.30968	-2.03475
CL-	8.23694E-02	2.92024E+00	8.22669E-02	-1.08477	-0.32742	-1.41219
SO4--	2.59474E-04	2.49245E-02	2.59151E-04	-3.58645	-1.30968	-4.89612
FE++	1.99686E-07	1.11519E-05	1.99438E-07	-6.70019	-1.30968	-8.00987
O2(AQ)	1.89546E-16	6.06524E-15	1.89310E-16	-15.72283	0.00000	-15.72283
H2(AQ)	1.29613E-02	2.61273E-02	1.29452E-02	-1.88789	0.00000	-1.88789
CH4(AQ)	4.98144E-02	7.99153E-01	4.97525E-02	-1.30319	0.00000	-1.30319
HS-	3.68488E-02	1.21851E+00	3.68029E-02	-1.43412	-0.32742	-1.76154
FE+++	4.02622E-14	2.24852E-12	4.02121E-14	-13.39564	-2.94677	-16.34241
HCO3-	4.58294E-01	2.79638E+01	4.57724E-01	-0.33940	-0.32742	-0.66682
CLO4-	1.65811E-42	1.64900E-40	1.65605E-42	-41.78093	-0.32742	-42.10835
OH-	4.81249E-01	8.18475E+00	4.80650E-01	-0.31817	-0.32742	-0.64559
HCOO-	2.29539E+00	1.03333E+02	2.29254E+00	0.36032	-0.32742	0.03290

CH3COO-	2.65775E-02	1.56926E+00	2.65444E-02	-1.57603	-0.32742	-1.90345
CH3CH2COO-	4.41643E-01	3.22714E+01	4.41094E-01	-0.35547	-0.32742	-0.68289
CO(AQ)	6.44359E-02	1.80487E+00	6.43557E-02	-1.19141	0.00000	-1.19141
ETHANE(AQ)	2.10287E-04	6.32320E-03	2.10025E-04	-3.67773	0.00000	-3.67773
ETHYLENE(AQ)	4.59785E-07	1.28986E-05	4.59213E-07	-6.33799	0.00000	-6.33799
PROPANE(AQ)	1.19171E-06	5.25498E-05	1.19022E-06	-5.92437	0.00000	-5.92437
HEXANE(AQ)	2.81865E-13	2.42902E-11	2.81515E-13	-12.55050	0.00000	-12.55050
BENZENE(AQ)	9.68529E-13	7.56551E-11	9.67324E-13	-12.01443	0.00000	-12.01443
TOLUENE(AQ)	3.87409E-15	3.56959E-13	3.86927E-15	-14.41237	0.00000	-14.41237
SI2O4(AQ)	3.42418E-01	4.11479E+01	3.41992E-01	-0.46598	0.00000	-0.46598
ALO2-	1.30638E-02	7.70506E-01	1.30475E-02	-1.88447	-0.32742	-2.21189
ALO2(SIO2)-	6.62791E-01	7.89150E+01	6.61966E-01	-0.17916	-0.32742	-0.50658
CACL+	2.43944E-04	1.84258E-02	2.43640E-04	-3.61325	-0.32742	-3.94067
CACL2(AQ)	3.95670E-07	4.39138E-05	3.95178E-07	-6.40321	0.00000	-6.40321
CACO3(AQ)	4.85669E-03	4.86102E-01	4.85065E-03	-2.31420	0.00000	-2.31420
CA(HCO3)+	3.06682E+00	3.10046E+02	3.06300E+00	0.48615	-0.24897	0.23718
CA(OH)+	3.99103E-04	2.27837E-02	3.98606E-04	-3.39946	-0.32742	-3.72687
CA(HSIO3)+	2.09250E-01	2.45182E+01	2.08990E-01	-0.67987	-0.32742	-1.00729
FECL+	1.07188E-07	9.78625E-06	1.07054E-07	-6.97040	-0.32742	-7.29781
FECL2(AQ)	8.39531E-05	1.06413E-02	8.38487E-05	-4.07650	0.00000	-4.07650
FE(HSIO3)+	1.18684E-01	1.57777E+01	1.18536E-01	-0.92615	-0.32742	-1.25357
KCL(AQ)	1.40731E-02	1.04917E+00	1.40556E-02	-1.85215	0.00000	-1.85215
KOH	1.90987E-01	1.07154E+01	1.90749E-01	-0.71954	0.00000	-0.71954
KSO4-	1.07403E-05	1.45161E-03	1.07269E-05	-4.96953	-0.32742	-5.29694
MGCL+	5.89263E-06	3.52132E-04	5.88529E-06	-5.23023	-0.32742	-5.55765
MG(HCO3)+	7.20270E-04	6.14550E-02	7.19374E-04	-3.14305	-0.32742	-3.47046
MG(HSIO3)+	2.24817E-01	2.27957E+01	2.24537E-01	-0.64871	-0.32742	-0.97613
MGSO4(AQ)	9.35163E-09	1.12559E-06	9.33999E-09	-8.02965	0.00000	-8.02965
NACL(AQ)	2.84486E-03	1.66262E-01	2.84132E-03	-2.54648	0.00000	-2.54648
NACO3-	1.57674E-03	1.30868E-01	1.57478E-03	-2.80278	-0.32742	-3.13020
NAHCO3(AQ)	2.27369E-02	1.91005E+00	2.27086E-02	-1.64381	0.00000	-1.64381
NAHSIO3(AQ)	2.91591E-03	2.91828E-01	2.91228E-03	-2.53577	0.00000	-2.53577
NAOH(AQ)	9.86014E-02	3.94377E+00	9.84787E-02	-1.00666	0.00000	-1.00666
HSIO3-	1.18652E+00	9.14709E+01	1.18505E+00	0.07374	-0.32742	-0.25368
HSO4-	1.79558E-03	1.74289E-01	1.79335E-03	-2.74633	-0.32742	-3.07375
H2S(AQ)	1.46714E-01	4.99941E+00	1.46532E-01	-0.83407	0.00000	-0.83407
CO2(AQ)	2.65541E+01	1.16864E+03	2.65211E+01	1.42359	0.00000	1.42359
HCL(AQ)	2.93967E-04	1.07183E-02	2.93601E-04	-3.53224	0.00000	-3.53224
CH3CH2COOH	5.13832E-05	3.80643E-03	5.13193E-05	-4.28972	0.00000	-4.28972
CH3COOH	1.69062E-03	1.01526E-01	1.68852E-03	-2.77249	0.00000	-2.77249
HCOOH	3.16537E-03	1.45688E-01	3.16143E-03	-2.50012	0.00000	-2.50012

--- activity ratios of cations ---

log (NA+ /h+**0)	4.3359981
log (K+ /h+**0)	4.5331272
log (CA++ /h+**0)	4.3093383
log (MG++ /h+**0)	3.2405013
log (AL+++ /h+**0)	-3.6913976
log (FE++ /h+**0)	1.3630639
log (FE+++ /h+**0)	-2.2830148

--- summary of solid product phases---

product	log moles	moles	grams	volume, cc
DIAMOND	-0.4823471	3.29346E-01	3.95578E+00	1.12636E+00
CLINOPYROXENE(SS)	-0.0565246	8.77961E-01	1.83695E+02	5.48994E+01
DIOPSIDE	-0.5515810	2.80814E-01	6.08110E+01	1.85899E+01
HEDENBERGITE	-1.3244184	4.73785E-02	1.17543E+01	3.13646E+00
JADEITE	-0.2598201	5.49769E-01	1.11130E+02	3.31730E+01
GARNET(SS)	0.3119273	2.05082E+00	3.00762E+02	2.36669E+02
PYROPE	0.0275946	1.06560E+00	1.43198E+02	1.20583E+02
ALMANDINE	-0.2154203	6.08947E-01	1.01058E+02	6.89085E+01
GROSSULAR	-0.4244996	3.76271E-01	5.65057E+01	4.71768E+01

--- grand summary of solid phases (e.s.+p.r.s.+reactants) ---

phase/end-member	log moles	moles	grams	volume, cc

DIAMOND	-0.4823471	3.29346E-01	3.95578E+00	1.12636E+00
COESITE	1.2577493	1.81029E+01	1.08770E+03	4.10720E+02
CLINOPYROXENE(SS)	1.2783170	1.89809E+01		
DIOPSIDE	0.5912209	3.90140E+00	8.44858E+02	2.58273E+02
HEDENBERGITE	0.2689693	1.85767E+00	4.60878E+02	1.22978E+02
JADEITE	1.1212916	1.32218E+01	2.67264E+03	7.97805E+02
GARNET(SS)	1.3043562	2.01538E+01		
PYROPE	1.0765447	1.19274E+01	1.60283E+03	1.34970E+03
ALMANDINE	0.7810248	6.03983E+00	1.00234E+03	6.83467E+02
GROSSULAR	0.3397625	2.18657E+00	3.28363E+02	2.74152E+02

	mass, grams	volume, cc
created	4.884122E+02	2.926945E+02
destroyed	7.875321E+02	3.778322E+02
net	-2.991199E+02	-8.513771E+01

warning-- these volume totals may be incomplete because
of missing partial molar volume data in the data base

--- mineral saturation state summary ---

mineral	affinity, kcal	state	mineral	affinity, kcal	state
DIAMOND	0.0000	satd	CORUNDUM	-6.0475	
BRUCITE	-7.4145		DIASPORE	-3.3009	
CALCITE	-4.4719		ARAGONITE	-2.7476	
MAGNESITE	-0.9458		DOLOMITE	-4.1814	
KYANITE	-3.6141		SILLIMANITE	-8.2168	
GROSSULAR	-3.9532		LAWSONITE	-4.4012	
FORSTERITE	-6.9861		ENSTATITE-CL	-1.5694	
ENSTATITE-OR	-1.4743		ENSTATITE-PR	-2.6741	
DIOPSIDE	-2.6575		HEDENBERGITE	-6.8062	
JADEITE	-1.0913		FERROSILITE	-5.2698	
K-FELDSPAR	-7.4733		TALC	-7.8610	
MUSCOVITE	-5.1077		PHLOGOPITE	-2.7757	
PYROPE	-1.5263		ALMANDINE	-2.8309	
COESITE	-0.2888		GRAPHITE	-0.3430	
PYRRHOTITE	-2.8357		PYRITE	-3.5915	
FERROUS_OXIDE	-5.6242		GIBBSITE	-7.4631	
SEPIOLITE	1044.1857	ssatd	SIDERITE	-3.9140	
QUARTZ-ALPHA	-0.9270		QUARTZ-BETA	-2.0050	
CRISTOBALITE-ALPHA	-5.7294		CRISTOBALITE-BETA	-7.7988	
CHALCEDONY	-1.8815		AMORPHOUS_SILICA	-3.2203	

--- summary of solid solutions ---

mineral	aff. kcal/mol		mole frac.	lambda	state
ORTHOPYROXENE(SS)	-1.0564				
FERROSILITE		-1.05645	0.1641026	1.00000	
ENSTATITE-OR		-1.05645	0.8358974	1.00000	
OLIVINE	-6.6884				
FAYALITE		-6.68839	0.1198849	1.00000	
FORSTERITE		-6.68839	0.8801151	1.00000	
BIOTITE	-2.7661				
PHLOGOPITE		-2.76607	0.9958601	1.00000	
ANNITE		-2.76607	0.0041399	1.00000	
CLINOPYROXENE(SS)	0.0000				saturated
DIOPSIDE		0.00000	0.3198480	1.00000	
HEDENBERGITE		0.00000	0.0539643	1.00000	
JADEITE		0.00000	0.6261877	1.00000	

```
 GARNET(SS)               0.0000                              saturated
    PYROPE                      0.00000    0.5195978     1.00000
    ALMANDINE                   0.00000    0.2969288     1.00000
    GROSSULAR                   0.00000    0.1834734     1.00000

 CALCITE(SS)              0.0000
    CALCITE                    -0.00001    0.1468787     1.00000
    MAGNESITE                  -0.00001    0.6665331     1.00000
    SIDERITE                   -0.00001    0.1865882     1.00000


 solid solution product phases

           xbar     lambda   activity  log xbar  log lambda log activity

   CLINOPYROXENE(SS)
 ideal solution

    DIOPSIDE
            0.3198    1.0000    0.3198   -0.4951    0.0000   -0.4951
    HEDENBERGITE
            0.0540    1.0000    0.0540   -1.2679    0.0000   -1.2679
    JADEITE
            0.6262    1.0000    0.6262   -0.2033    0.0000   -0.2033

   GARNET(SS)
 ideal solution

    PYROPE
            0.5196    1.0000    0.5196   -0.2843    0.0000   -0.2843
    ALMANDINE
            0.2969    1.0000    0.2969   -0.5273    0.0000   -0.5273
    GROSSULAR
            0.1835    1.0000    0.1835   -0.7364    0.0000   -0.7364


          --- summary of gas species ---

          gas               log fugacity         fugacity         partial pressure

   CO2(G)                        9.11217       1.29471E+09
   O2(G)                        -9.59763       2.52565E-10
   S2(G)                         4.02828       1.06728E+04
   CH4(G)                        6.96681       9.26434E+06
   H2(G)                         3.80616       6.39967E+03
   H2S(G)                        7.28400       1.92308E+07
   H2O(G)                        6.90034       7.94958E+06



 - - - - - - - - - - - - - - - - - - - - - - - - - - - - - - - -




 stepping to zi=  1.8971E+00, delzi=  1.0000E-08, nord= 0
               ncycle=  0
 * note- reactant CLINOPYROXENE(SS)        has saturated but differs        in composition from the corresponding product.  it
must        remain in the reactant subsystem (rsatch)
 * note- reactant GARNET(SS)               has saturated but differs        in composition from the corresponding product.  it
must        remain in the reactant subsystem (rsatch)
 steps completed =    93, iter =  3, ncorr = 0
 most rapidly changing is zvclg1(HEDENBERGITE      ) =     -1.3244

 stepping to zi=  1.8971E+00, delzi=  1.0000E-08, nord= 0
               ncycle=  0
 * note- reactant CLINOPYROXENE(SS)        has saturated but differs        in composition from the corresponding product.  it
must        remain in the reactant subsystem (rsatch)
 * note- reactant GARNET(SS)               has saturated but differs        in composition from the corresponding product.  it
must        remain in the reactant subsystem (rsatch)
 steps completed =    94, iter =  3, ncorr = 0
 most rapidly changing is zvclg1(HEDENBERGITE      ) =     -1.3244

 stepping to zi=  1.8971E+00, delzi=  1.0000E-08, nord= 0
               ncycle=  0
 * note- reactant CLINOPYROXENE(SS)        has saturated but differs        in composition from the corresponding product.  it
```

```
must        remain in the reactant subsystem (rsatch)
 * note- reactant GARNET(SS)               has saturated but differs        in composition from the corresponding product.  it
must        remain in the reactant subsystem (rsatch)
 steps completed =    95, iter =  3, ncorr = 0
 most rapidly changing is zvclg1(HEDENBERGITE      ) =     -1.3244

 stepping to zi=  1.8971E+00, delzi=  1.0000E-07, nord= 1
              ncycle=  0
 * note- reactant CLINOPYROXENE(SS)        has saturated but differs        in composition from the corresponding product.  it
must        remain in the reactant subsystem (rsatch)
 * note- reactant GARNET(SS)               has saturated but differs        in composition from the corresponding product.  it
must        remain in the reactant subsystem (rsatch)
 steps completed =    96, iter =  3, ncorr = 0
 most rapidly changing is zvclg1(HEDENBERGITE      ) =     -1.3244

 stepping to zi=  1.8971E+00, delzi=  1.0000E-06, nord= 1
              ncycle=  0
 * note- reactant CLINOPYROXENE(SS)        has saturated but differs        in composition from the corresponding product.  it
must        remain in the reactant subsystem (rsatch)
 * note- reactant GARNET(SS)               has saturated but differs        in composition from the corresponding product.  it
must        remain in the reactant subsystem (rsatch)
 steps completed =    97, iter =  5, ncorr = 0
 most rapidly changing is zvclg1(HEDENBERGITE      ) =     -1.3244

 stepping to zi=  1.8971E+00, delzi=  1.0000E-05, nord= 1
              ncycle=  0
 * note- reactant CLINOPYROXENE(SS)        has saturated but differs        in composition from the corresponding product.  it
must        remain in the reactant subsystem (rsatch)
 * note- reactant GARNET(SS)               has saturated but differs        in composition from the corresponding product.  it
must        remain in the reactant subsystem (rsatch)
 steps completed =    98, iter =  7, ncorr = 0
 most rapidly changing is zvclg1(HEDENBERGITE      ) =     -1.3244

 stepping to zi=  1.8972E+00, delzi=  1.0000E-04, nord= 2
              ncycle=  0
 * note- reactant CLINOPYROXENE(SS)        has saturated but differs        in composition from the corresponding product.  it
must        remain in the reactant subsystem (rsatch)
 * note- reactant GARNET(SS)               has saturated but differs        in composition from the corresponding product.  it
must        remain in the reactant subsystem (rsatch)
 steps completed =    99, iter =  9, ncorr = 0
 most rapidly changing is zvclg1(HEDENBERGITE      ) =     -1.3243

 stepping to zi=  1.8982E+00, delzi=  1.0000E-03, nord= 2
              ncycle=  0
 * note- reactant CLINOPYROXENE(SS)        has saturated but differs        in composition from the corresponding product.  it
must        remain in the reactant subsystem (rsatch)
 * note- reactant GARNET(SS)               has saturated but differs        in composition from the corresponding product.  it
must        remain in the reactant subsystem (rsatch)
 steps completed =   100, iter = 11, ncorr = 0
 most rapidly changing is zvclg1(HEDENBERGITE      ) =     -1.3236

 stepping to zi=  1.9082E+00, delzi=  1.0000E-02, nord= 2
              ncycle=  0
 * note- reactant CLINOPYROXENE(SS)        has saturated but differs        in composition from the corresponding product.  it
must        remain in the reactant subsystem (rsatch)
 * note- reactant GARNET(SS)               has saturated but differs        in composition from the corresponding product.  it
must        remain in the reactant subsystem (rsatch)
 steps completed =   101, iter = 13, ncorr = 0
 most rapidly changing is zvclg1(HEDENBERGITE      ) =     -1.3166

 stepping to zi=  2.0082E+00, delzi=  1.0000E-01, nord= 3
              ncycle=  0
 * note- reactant CLINOPYROXENE(SS)        has saturated but differs        in composition from the corresponding product.  it
must        remain in the reactant subsystem (rsatch)
 * note- reactant GARNET(SS)               has saturated but differs        in composition from the corresponding product.  it
must        remain in the reactant subsystem (rsatch)
 steps completed =   102, iter = 15, ncorr = 0
 most rapidly changing is zvclg1(HEDENBERGITE      ) =     -1.2511

 stepping to zi=  2.2189E+00, delzi=  2.1070E-01, nord= 4
              ncycle=  0
 * note- reactant CLINOPYROXENE(SS)        has saturated but differs        in composition from the corresponding product.  it
must        remain in the reactant subsystem (rsatch)
 * note- reactant GARNET(SS)               has saturated but differs        in composition from the corresponding product.  it
must        remain in the reactant subsystem (rsatch)
 steps completed =   103, iter = 16, ncorr = 0
```

```
 most rapidly changing is zvclg1(HEDENBERGITE      ) =     -1.1376

 stepping to zi=  2.5222E+00, delzi=  3.0333E-01, nord= 4
              ncycle=  0
 * note- reactant CLINOPYROXENE(SS)        has saturated but differs        in composition from the corresponding product.  it
must        remain in the reactant subsystem (rsatch)
 * note- reactant GARNET(SS)               has saturated but differs        in composition from the corresponding product.  it
must        remain in the reactant subsystem (rsatch)
 steps completed =   104, iter = 16, ncorr = 0
 most rapidly changing is zvclg1(HEDENBERGITE      ) =     -1.0117

 stepping to zi=  2.8746E+00, delzi=  3.5243E-01, nord= 5
              ncycle=  0
 iter =   14
       1 supersaturated pure minerals
       0 supersaturated solid solutions

                         the most supersaturated phases     affinity, kcal

                           1      60   COESITE                 0.00814108


   attempted species assemblage no.   2

             1      1  H2O
             2      2  NA+
             3      3  K+
             4      4  CA++
             5      5  MG++
             6      6  AL+++
             7      7  SIO2(AQ)
             8     13  H+
             9     14  CO3--
            10     16  CL-
            11     17  SO4--
            12     21  FE++
            13     29  O2(G)
            14      2  DIAMOND
            15     60  COESITE
            16      1  CLINOPYRDIOPSIDE
            17      2  CLINOPYRHEDENBERGITE
            18      3  CLINOPYRJADEITE
            19      1  GARNET(SPYROPE
            20      2  GARNET(SALMANDINE
            21      3  GARNET(SGROSSULAR

 --- reactant COESITE                  has saturated and been        transferred to the equilibrium subsystem ---
 * note- reactant CLINOPYROXENE(SS)        has saturated but differs        in composition from the corresponding product.  it
must        remain in the reactant subsystem (rsatch)
 * note- reactant GARNET(SS)               has saturated but differs        in composition from the corresponding product.  it
must        remain in the reactant subsystem (rsatch)
 steps completed =   105, iter = 19, ncorr = 0
 - - - - - - - - - - - - - - - - - - - - - - - - - - - - - - - -



                    reaction progress        =  2.87461979564810E+00
                    log of reaction progress =       0.4585804

                    temperature    =    900.000 degrees c
                    total pressure =  50000.000 bars

                    computing units remaining =      0.000


 change in the product phase assemblage


                              --- reactant summary ---



        reactant            moles       delta moles      grams        delta grams

   CLINOPYROXENE(SS)       1.71254E+01    2.87462E+00    3.58977E+03    6.02569E+02
   GARNET(SS)              1.71254E+01    2.87462E+00    2.49060E+03    4.18065E+02
```

```
COESITE                 0.00000E+00    2.87462E+00    0.00000E+00    1.72720E+02


             current total mass =  6.08037E+03 grams
             delta total mass   =  1.19335E+03 grams
             delta total volume =    572.53228 cc


      reactant           affinity     rel. rate

CLINOPYROXENE(SS)          0.0917    0.00000E+00
GARNET(SS)                 0.0623    0.00000E+00
COESITE                    0.0000    0.00000E+00

       affinity of the overall irreversible reaction=     0.000 kcal
       contributions from irreversible reactions
       with no thermodynamic data are not included



   --- element totals for the aqueous phase ---

element         mg/kg soln.         molal conc.          moles

   O         7.059368E+05       1.387779E+02       1.396019E+02
   NA        6.865677E+03       9.393056E-01       9.448827E-01
   K         2.100766E+04       1.689966E+00       1.700000E+00
   CA        3.910574E+04       3.068817E+00       3.087038E+00
   MG        1.877471E+03       2.429605E-01       2.444031E-01
   AL        5.797669E+03       6.758415E-01       6.798544E-01
   SI        4.626630E+04       5.181324E+00       5.212088E+00
   H         3.898296E+04       1.216510E+02       1.223733E+02
   C         1.286637E+05       3.369263E+01       3.389269E+01
   CL        1.120531E+03       9.940975E-02       1.000000E-01
   S         1.880962E+03       1.845333E-01       1.856290E-01
   FE        2.494540E+03       1.404911E-01       1.413253E-01

   co3--                          0.000000E+00       0.000000E+00
   so4--                          0.000000E+00       0.000000E+00
   s--                            0.000000E+00       0.000000E+00

 warning-- co3--, so4--, and s-- totals require that routine comp1
 have the names of non-carbonate carbon, sulfide sulfur,
 and non-sulfate sulfur aqueous species


single ion activities and activity coefficients are here defined
with respect to the internal ph scale



                                   ph           eh           pe

  internal ph scale              4.6537       1.3663    5.8697E+00
  modified nbs ph scale          4.3281       1.4421    6.1953E+00
  rational ph scale              4.3281       1.4421    6.1953E+00

phcl =      6.0655



       oxygen fugacity =  2.54492E-10
       log oxygen fugacity =  -9.59433



                         activity of water =   0.99667
            log activity of water =  -0.00145
            alkalinity =  0.000000E+00 equiv/kg solvent
                                      (not def. for t.gt.50 c)

                 ionic strength =  5.940217E+00 molal
              sum of molalities =   40.9405171533063
            osmotic coefficient =    0.00452
                 equiv. stoich. ionic strength =  9.940975E-02 molal
```

mass of solution = 3.163947 kg
mass of solvent = 1.005938 kg
mass of solutes = 2.158009 kg
conc of solutes = 68.206245 per cent (w/w)

species	moles	grams	conc	log conc	log g	log act
H2O	5.58383E+01	1.00594E+03				
NA+	8.38512E-01	1.92772E+01	8.33563E-01	-0.07906	-0.32557	-0.40463
K+	1.50651E+00	5.89021E+01	1.49762E+00	0.17540	-0.32557	-0.15017
CA++	1.73755E-04	6.96411E-03	1.72730E-04	-3.76263	-1.30228	-5.06491
MG++	1.53552E-05	3.73209E-04	1.52646E-05	-4.81631	-1.30228	-6.11859
AL+++	1.83113E-15	4.94066E-14	1.82032E-15	-14.73985	-2.93013	-17.66998
SIO2(AQ)	1.80997E+00	1.08751E+02	1.79929E+00	0.25510	0.00000	0.25510
H+	4.72595E-05	4.76329E-05	4.69806E-05	-4.32808	-0.32557	-4.65365
CO3--	1.61354E-01	9.68272E+00	1.60402E-01	-0.79479	-1.30228	-2.09707
CL-	8.24618E-02	2.92352E+00	8.19750E-02	-1.08632	-0.32557	-1.41189
SO4--	2.26304E-04	2.17382E-02	2.24968E-04	-3.64788	-1.30228	-4.95016
FE++	2.23537E-07	1.24839E-05	2.22218E-07	-6.65322	-1.30228	-7.95550
O2(AQ)	1.91887E-16	6.14015E-15	1.90754E-16	-15.71953	0.00000	-15.71953
H2(AQ)	1.29728E-02	2.61506E-02	1.28963E-02	-1.88954	0.00000	-1.88954
CH4(AQ)	4.96705E-02	7.96844E-01	4.93773E-02	-1.30647	0.00000	-1.30647
HS-	3.45765E-02	1.14337E+00	3.43725E-02	-1.46379	-0.32557	-1.78936
FE+++	4.76750E-14	2.66251E-12	4.73936E-14	-13.32428	-2.93013	-16.25441
HCO3-	4.28369E-01	2.61378E+01	4.25841E-01	-0.37075	-0.32557	-0.69632
CLO4-	1.68540E-42	1.67614E-40	1.67545E-42	-41.77587	-0.32557	-42.10144
OH-	4.46420E-01	7.59240E+00	4.43785E-01	-0.35283	-0.32557	-0.67840
HCOO-	2.13738E+00	9.62197E+01	2.12476E+00	0.32731	-0.32557	0.00174
CH3COO-	2.46544E-02	1.45571E+00	2.45089E-02	-1.61068	-0.32557	-1.93625
CH3CH2COO-	4.08139E-01	2.98233E+01	4.05730E-01	-0.39176	-0.32557	-0.71733
CO(AQ)	6.49843E-02	1.82024E+00	6.46007E-02	-1.18976	0.00000	-1.18976
ETHANE(AQ)	2.08887E-04	6.28112E-03	2.07654E-04	-3.68266	0.00000	-3.68266
ETHYLENE(AQ)	4.58457E-07	1.28614E-05	4.55751E-07	-6.34127	0.00000	-6.34127
PROPANE(AQ)	1.17930E-06	5.20028E-05	1.17234E-06	-5.93095	0.00000	-5.93095
HEXANE(AQ)	2.75783E-13	2.37660E-11	2.74155E-13	-12.56200	0.00000	-12.56200
BENZENE(AQ)	9.62082E-13	7.51515E-11	9.56403E-13	-12.01936	0.00000	-12.01936
TOLUENE(AQ)	3.83377E-15	3.53244E-13	3.81114E-15	-14.41895	0.00000	-14.41895
SI2O4(AQ)	4.40728E-01	5.29617E+01	4.38127E-01	-0.35840	0.00000	-0.35840
ALO2-	1.16364E-02	6.86318E-01	1.15677E-02	-1.93675	-0.32557	-2.26232
ALO2(SIO2)-	6.68218E-01	7.95611E+01	6.64274E-01	-0.17765	-0.32557	-0.50322
CACL+	2.43477E-04	1.83905E-02	2.42039E-04	-3.61611	-0.32557	-3.94168
CACL2(AQ)	3.96876E-07	4.40477E-05	3.94534E-07	-6.40392	0.00000	-6.40392
CACO3(AQ)	4.21435E-03	4.21810E-01	4.18947E-03	-2.37784	0.00000	-2.37784
CA(HCO3)+	2.86300E+00	2.89441E+02	2.84610E+00	0.45425	-0.24790	0.20636
CA(OH)+	3.69096E-04	2.10707E-02	3.66917E-04	-3.43543	-0.32557	-3.76100
CA(HSIO3)+	2.19035E-01	2.56647E+01	2.17742E-01	-0.66206	-0.32557	-0.98763
FECL+	1.21618E-07	1.11038E-05	1.20900E-07	-6.91757	-0.32557	-7.24314
FECL2(AQ)	9.57293E-05	1.21340E-02	9.51642E-05	-4.02153	0.00000	-4.02153
FE(HSIO3)+	1.41229E-01	1.87748E+01	1.40396E-01	-0.85265	-0.32557	-1.17822
KCL(AQ)	1.42527E-02	1.06256E+00	1.41686E-02	-1.84867	0.00000	-1.84867
KOH	1.79224E-01	1.00555E+01	1.78166E-01	-0.74917	0.00000	-0.74917
KSO4-	9.55722E-06	1.29171E-03	9.50081E-06	-5.02224	-0.32557	-5.34781
MGCL+	6.09021E-06	3.63939E-04	6.05426E-06	-5.21794	-0.32557	-5.54351
MG(HCO3)+	6.95034E-04	5.93018E-02	6.90932E-04	-3.16056	-0.32557	-3.48613
MG(HSIO3)+	2.43687E-01	2.47090E+01	2.42248E-01	-0.61574	-0.32557	-0.94131
MGSO4(AQ)	8.56481E-09	1.03088E-06	8.51426E-09	-8.06985	0.00000	-8.06985
NACL(AQ)	2.52484E-03	1.47559E-01	2.50994E-03	-2.60034	0.00000	-2.60034
NACO3-	1.20630E-03	1.00122E-01	1.19918E-03	-2.92112	-0.32557	-3.24669
NAHCO3(AQ)	1.88404E-02	1.58272E+00	1.87292E-02	-1.72748	0.00000	-1.72748
NAHSIO3(AQ)	2.71409E-03	2.71630E-01	2.69807E-03	-2.56895	0.00000	-2.56895
NAOH(AQ)	8.10852E-02	3.24317E+00	8.06066E-02	-1.09363	0.00000	-1.09363
HSIO3-	1.24578E+00	9.60392E+01	1.23843E+00	0.09287	-0.32557	-0.23270
HSO4-	1.71067E-03	1.66047E-01	1.70057E-03	-2.76941	-0.32557	-3.09498
H2S(AQ)	1.49106E-01	5.08090E+00	1.48226E-01	-0.82908	0.00000	-0.82908
CO2(AQ)	2.68821E+01	1.18308E+03	2.67234E+01	1.42689	0.00000	1.42689
HCL(AQ)	3.18749E-04	1.16219E-02	3.16867E-04	-3.49912	0.00000	-3.49912
CH3CH2COOH	5.14306E-05	3.80994E-03	5.11270E-05	-4.29135	0.00000	-4.29135
CH3COOH	1.69860E-03	1.02005E-01	1.68857E-03	-2.77248	0.00000	-2.77248
HCOOH	3.19236E-03	1.46930E-01	3.17351E-03	-2.49846	0.00000	-2.49846

--- activity ratios of cations ---

log (NA+ /h+**0) 4.2490198
log (K+ /h+**0) 4.5034836

log (CA++ /h+**0)	4.2423902
log (MG++ /h+**0)	3.1887087
log (AL+++ /h+**0)	-3.7090284
log (FE++ /h+**0)	1.3518014
log (FE+++ /h+**0)	-2.2934554

--- summary of solid product phases---

product	log moles	moles	grams	volume, cc
DIAMOND	-0.2763718	5.29210E-01	6.35634E+00	1.80990E+00
COESITE	1.2343210	1.71522E+01	1.03058E+03	3.89150E+02
CLINOPYROXENE(SS)	0.3361868	2.16864E+00	4.53865E+02	1.35551E+02
DIOPSIDE	-0.1700124	6.76064E-01	1.46403E+02	4.47554E+01
HEDENBERGITE	-0.9023197	1.25222E-01	3.10669E+01	8.28969E+00
JADEITE	0.1358800	1.36735E+00	2.76395E+02	8.25060E+01
GARNET(SS)	0.4505958	2.82225E+00	4.15383E+02	3.25340E+02
PYROPE	0.1564822	1.43378E+00	1.92674E+02	1.62246E+02
ALMANDINE	-0.0459620	8.99576E-01	1.49290E+02	1.01796E+02
GROSSULAR	-0.3107826	4.88897E-01	7.34191E+01	6.12979E+01

--- grand summary of solid phases (e.s.+p.r.s.+reactants) ---

phase/end-member	log moles	moles	grams	volume, cc
DIAMOND	-0.2763718	5.29210E-01	6.35634E+00	1.80990E+00
COESITE	1.2343210	1.71522E+01	1.03058E+03	3.89150E+02
CLINOPYROXENE(SS)	1.2854227	1.92940E+01		
DIOPSIDE	0.6129046	4.10114E+00	8.88112E+02	2.71495E+02
HEDENBERGITE	0.2642888	1.83776E+00	4.55938E+02	1.21660E+02
JADEITE	1.1256477	1.33551E+01	2.69959E+03	8.05848E+02
GARNET(SS)	1.2998914	1.99476E+01		
PYROPE	1.0685201	1.17090E+01	1.57348E+03	1.32499E+03
ALMANDINE	0.7808349	6.03719E+00	1.00191E+03	6.83168E+02
GROSSULAR	0.3427059	2.20144E+00	3.30596E+02	2.76016E+02

	mass, grams	volume, cc
created	1.906185E+03	8.518515E+02
destroyed	1.193354E+03	5.725323E+02
net	7.128307E+02	2.793192E+02

warning-- these volume totals may be incomplete because
of missing partial molar volume data in the data base

--- mineral saturation state summary ---

mineral	affinity, kcal	state	mineral	affinity, kcal	state
DIAMOND	0.0000	satd	CORUNDUM	-6.2367	
BRUCITE	-7.6925		DIASPORE	-3.3955	
CALCITE	-4.8135		ARAGONITE	-3.0893	
MAGNESITE	-1.2060		DOLOMITE	-4.7833	
KYANITE	-3.5145		SILLIMANITE	-8.1172	
GROSSULAR	-4.0872		LAWSONITE	-4.3721	
FORSTERITE	-7.2533		ENSTATITE-CL	-1.5586	
ENSTATITE-OR	-1.4636		ENSTATITE-PR	-2.6634	
DIOPSIDE	-2.7173		HEDENBERGITE	-6.6485	
JADEITE	-1.0753		FERROSILITE	-5.0415	
K-FELDSPAR	-6.8607		TALC	-7.5399	
MUSCOVITE	-4.6842		PHLOGOPITE	-2.9971	

```
PYROPE                  -1.5788               ALMANDINE              -2.6656
COESITE                  0.0000  satd         GRAPHITE               -0.3430
PYRRHOTITE              -2.8693               PYRITE                 -3.5896
FERROUS_OXIDE           -5.6846               GIBBSITE               -7.5577
SEPIOLITE             1044.4962  ssatd        SIDERITE               -3.9567
QUARTZ-ALPHA            -0.6383               QUARTZ-BETA            -1.7162
CRISTOBALITE-ALPHA      -5.4406               CRISTOBALITE-BETA      -7.5100
CHALCEDONY              -1.5927               AMORPHOUS_SILICA       -2.9315


                    --- summary of solid solutions  ---

mineral               aff. kcal/mol  mole frac.    lambda      state

ORTHOPYROXENE(SS)        -1.0086
    FERROSILITE                -1.00855    0.1773086     1.00000
    ENSTATITE-OR               -1.00855    0.8226914     1.00000

OLIVINE                  -6.8990
    FAYALITE                   -6.89895    0.1410166     1.00000
    FORSTERITE                 -6.89895    0.8589834     1.00000

BIOTITE                  -2.9843
    PHLOGOPITE                 -2.98432    0.9945299     1.00000
    ANNITE                     -2.98432    0.0054701     1.00000

CLINOPYROXENE(SS)         0.0000                             saturated
    DIOPSIDE                    0.00000    0.3117459     1.00000
    HEDENBERGITE                0.00000    0.0577422     1.00000
    JADEITE                     0.00000    0.6305118     1.00000

GARNET(SS)                0.0000                             saturated
    PYROPE                      0.00000    0.5080265     1.00000
    ALMANDINE                   0.00000    0.3187441     1.00000
    GROSSULAR                   0.00000    0.1732294     1.00000

CALCITE(SS)              -0.2297
    CALCITE                    -0.22968    0.1399916     1.00000
    MAGNESITE                  -0.22968    0.6578401     1.00000
    SIDERITE                   -0.22968    0.2021683     1.00000


solid solution product phases

           xbar     lambda   activity  log xbar  log lambda log activity

  CLINOPYROXENE(SS)
ideal solution

    DIOPSIDE
            0.3117    1.0000    0.3117   -0.5062    0.0000   -0.5062
    HEDENBERGITE
            0.0577    1.0000    0.0577   -1.2385    0.0000   -1.2385
    JADEITE
            0.6305    1.0000    0.6305   -0.2003    0.0000   -0.2003

  GARNET(SS)
ideal solution

    PYROPE
            0.5080    1.0000    0.5080   -0.2941    0.0000   -0.2941
    ALMANDINE
            0.3187    1.0000    0.3187   -0.4966    0.0000   -0.4966
    GROSSULAR
            0.1732    1.0000    0.1732   -0.7614    0.0000   -0.7614


          --- summary of gas species ---

          gas               log fugacity         fugacity         partial pressure

  CO2(G)                         9.11547       1.30459E+09
  O2(G)                         -9.59433       2.54492E-10
  S2(G)                          4.04155       1.10040E+04
  CH4(G)                         6.96353       9.19448E+06
  H2(G)                          3.80451       6.37549E+03
```

```
  H2S(G)                        7.28899       1.94531E+07
  H2O(G)                        6.90035       7.94970E+06



- - - - - - - - - - - - - - - - - - - - - - - - - - - - - - - -



                    --- all rates are zero ---

                    --- each reactant is saturated or exhausted ---

---  the reaction path has terminated normally ---


        105 steps were taken
      zi increased from
              0.00000E+00 to  2.87462E+00
      the average value of delzi was  2.73773E-02
      the average matrix dimension was   18


         start time =
           end time =

         user time =      0.000
          cpu time =      0.000

--- no further input found ---
```

Supplementary References

1 Kessel, R., Ulmer, P., Pettke, T., Schmidt, M. W. & Thompson, A. B. The water-basalt system at 4 to 6 GPa: Phase relations and second critical endpoint in a K-free eclogite at 700 to 1400 °C. *Earth Planet. Sci. Lett.* **237**, 873-892, (2005).
